# The complete chloroplast genome of *Cinnamomum camphora* and its comparison with related *Lauraceae* species

**DOI:** 10.7717/peerj.3820

**Published:** 2017-09-18

**Authors:** Caihui Chen, Yongjie Zheng, Sian Liu, Yongda Zhong, Yanfang Wu, Jiang Li, Li-An Xu, Meng Xu

**Affiliations:** 1 Co-Innovation Center for Sustainable Forestry in Southern China, Nanjing Forestry University, Nanjing, Jiangsu, China; 2 Camphor Engineering Technology Research Center for State Forestry Administration, Jiangxi Academy of Forestry, Nanchang, Jiangxi, China; 3 Institute of Biological Resources, Jiangxi Academy of Science, Nanchang, Jiangxi, China

**Keywords:** Chloroplast genome, *Cinnamomum camphora*, *Lauraceae*, Illumina sequencing, Phylogeny

## Abstract

*Cinnamomum camphora*, a member of the *Lauraceae* family, is a valuable aromatic and timber tree that is indigenous to the south of China and Japan. All parts of *Cinnamomum camphora* have secretory cells containing different volatile chemical compounds that are utilized as herbal medicines and essential oils. Here, we reported the complete sequencing of the chloroplast genome of *Cinnamomum camphora* using illumina technology. The chloroplast genome of *Cinnamomum camphora* is 152,570 bp in length and characterized by a relatively conserved quadripartite structure containing a large single copy region of 93,705 bp, a small single copy region of 19,093 bp and two inverted repeat (IR) regions of 19,886 bp. Overall, the genome contained 123 coding regions, of which 15 were repeated in the IR regions. An analysis of chloroplast sequence divergence revealed that the small single copy region was highly variable among the different genera in the *Lauraceae* family. A total of 40 repeat structures and 83 simple sequence repeats were detected in both the coding and non-coding regions. A phylogenetic analysis indicated that *Calycanthus* is most closely related to *Lauraceae*, both being members of *Laurales*, which forms a sister group to *Magnoliids*. The complete sequence of the chloroplast of *Cinnamomum camphora* will aid in in-depth taxonomical studies of the *Lauraceae* family in the future. The genetic sequence information will also have valuable applications for chloroplast genetic engineering.

## Introduction

*Cinnamomum*, contains 250–300, or more, species worldwide and is a species-rich genus of evergreen aromatic tree and shrub belonging to the *Lauraceae* family. As the representative species of *Cinanamomum*, the camphor tree (*Cinnamomum camphora*) is a broad-leaved evergreen characterized by aromatic oils in all of the plant parts. This species originated in the southern parts of China and Japan, and has a widespread naturalized distribution in many other countries. Camphor trees can reach up to 40 m in height and live to be over 1,000 years old. They have oval or elliptical leaves with wavy margins that give off a distinctive aromatic smell when crushed. In addition to providing sources for spices and essential oils, the species also has a notable commodity value as lumber and is ecologically significant in garden construction (Babu et al., 2003).

Chloroplast serves as the metabolic center of plant life by converting solar energy to carbohydrates through photosynthesis and oxygen release (Neuhaus & Emes, 2000). The advent of high-throughput sequencing technology has facilitated rapid progress in the field of chloroplast genetics and genomics. Approximately 800 complete chloroplast genomes from a variety of land plants have been retained in the National Center for Biotechnology Information (NCBI) organelle genome database since the first chloroplast genome of tobacco (*Nicotiana tabacum*) (Shinozaki et al., 1986) and liverwort (*Marchantia polymorpha*) (Ohyama et al., 1986), which were sequenced simultaneously in 1986. The sequenced chloroplast genomes have improved our understanding of plant biology and evolutionary relationships. Chloroplast genomes of land plants have a high degree of conservation in size, structure, gene content and the gene’s linear order. They comprise a single circular chromosome, typically ranging in size from 107 kb (*Cathaya argyrophylla*) to 218 kb (*Pelargonium*) (Chumley et al., 2006; Lin et al., 2010). Chloroplast genomes have a quadripartite structure, with a pair of inverted repeats (IRs) separated by one large and one small single copy region (Yurina & Odintsova, 1998). Several plant chloroplast genomes also show significant structural rearrangements, with evidence of the loss of IR regions or entire gene families (Hirao et al., 2008; Yi et al., 2013). Additionally, the presence of IRs might stabilize the chloroplast genomes organization (Chang et al., 2006; Wicke et al., 2011). The chloroplast genome consists of 120–130 genes divided into three functional categories, protein-coding genes, introns and intergenic spacers. Most genes primarily participate in photosynthesis, transcription and translation.

While morphological and palynological studies of the phylogeny of *Lauraceae* family have been performed (Rohwer, 1993; Shang & Tang, 1994), the classification systems have not been widely accepted or approved. The use of morphological and palynological characteristics generally resolves the majority plant classifications but there is currently insufficient information on the *Lauraceae* family to provide the high-resolution necessary to differentiate some within-species taxa, whose taxonomic relationships are controversial. Therefore, research has been conducted on the relationships among the members of the *Lauraceae* family using multiple short sequences inferred from the chloroplast (Chanderbali, van der Werff & Renner, 2001; Lee, Lee & Choi, 2013; Li et al., 2004; Rohwer, 2000), which provides important molecular information that can be applied to deciphering evolutionary relationships between closely related taxa with phylogenetic clades.

In this study, we sequenced and analyzed the complete chloroplast genome of *Cinnamomum camphora* based on illumina high-throughput sequencing technology. In addition to describing the plastic features of the chloroplast genome, we compared the gene content, repeat structures and sequence divergence with other reported species in the *Lauraceae* family. We also presented results of a phylogenetic analysis of protein sequences from *Cinnamomum camphora* and 25 other plant species. The complete chloroplast genome of *Cinnamomum camphora*, in conjunction with previously reported chloroplast genome sequences, will improve our understanding of the evolution relationships of genera in the *Lauraceae* family, especially regarding the position of *Cinnamomum camphora* in evolution and plant systematics. Moreover, the complete genome sequence of *Cinnamomum camphora* provides valuable data for that can be used in chloroplast genetic engineering.

## Materials and Methods

### Samples and genome sequencing

Fresh young leaves of *Cinnamomum camphora* were obtained from the campus of Nanjing Forestry University. The chloroplasts of *Cinnamomum camphora* were isolated using the Sigma Chloroplast DNA Isolation kit (Sigma, St. Louis, MO, USA), and chloroplast DNA was extracted using DNaesy Plant Mini Kit (QIAGEN, Hilden, Germany). The purified DNA was subjected to hydroshearing, end repair and then interrupted randomly to construct 350 bp libraries. The complete library, with an average read length of 150 bp, was sequenced using Illumina Hiseq2000 platform.

### Chloroplast genome assemble and annotation

To ensure accurate and reliable analyses, raw reads were proofread and assembled with SOAP denovo (Li et al., 2008). The generated contigs were assembled using the chloroplast genome sequence of *Cinnamomum micranthum* (KT348516.1) as a reference. The chloroplast genome sequences of *Cinnamomum camphora* were annotated through the online program Dual Organellar Genome Annotator (Solovyev et al., 2006). The annotation results were manually checked, including the start and stop codons, and adjusted by comparison to homologous genes from other sequenced chloroplast genomes. Transfer RNA (tRNA) genes were verified using tRNA scan-SE in organellar search mode with default parameters (Schattner, Brooks & Lowe, 2005). The circular chloroplast genome map of *Cinnamomum camphora* was drawn using the OGDRAW program (Lohse, Drechsel & Bock, 2007). Nucleotide frequency and relative synonymous codon usage (RSCU) (Sharp, Tuohy & Mosurski, 1986) were analyzed using DAMBE (Xia, 2013) on the protein-coding genes and only genes in inverted repeat region A (IRA) were used to represent repeated genes.

### Repeat structures and simple sequence repeats (SSRs) analysis

REPuter was used to identify forward and palindromic repeats with a minimal size of 30 bp, hamming distance of three and over 90% identity (Kurtz et al., 2001). Tandem repeat sequences were identified in *Cinnamomum camphora* using Tandem Repeats Finder with default parameters (Benson, 1999). Simple sequence repeats (SSRs) were detected using the microsatellite identification tool MISA (http://pgrc.ipk-gatersleben.de/misa/) with the following thresholds: minimum SSR motif length of 10 bp, and 10 repeat units for mononucleotide SSRs, five repeat units for dinucleotide SSRs, four repeat units for trinucleotide SSRs, and three repeat units for tetra-, penta- and hexanucleotide SSRs. The maximum size of interruption allowed between two different SSRs in a compound SSR was 100 bp (von Stackelberg, Rensing & Reski, 2006). All of the repeats identified with the above programs were manually verified to remove redundant results.

### Comparative analysis of different *Lauraceae* plastomes

To encompass the complete nucleotide diversity among *Lauraceae* species, the complete chloroplast genome sequences were aligned using MAFFT 7.222 software (Katoh & Standley, 2013), and manually adjusted with BioEdit software (Hall, 1999). DnaSP 5.0 software was used to conduct a sliding window analysis to calculate the nucleotide variability (Pi) values (Librado & Rozas, 2009). Pi is defined as the average number of nucleotide differences per site between two DNA sequences in all possible pairs in the sample population (Nei & Li, 1979). The window length was set to 600 bp, and the step size was set to 200 bp.

### Phylogenetic analysis

A molecular phylogenetic tree was constructed using 26 different plant species with TreeBeST (http://treesoft.sourceforge.net/treebest.shtml). Among these 26 taxa, *Abies koreana*, *Picea sitchensis*, *Pinus taiwanensis*, *Podocarpus lambertii*, *Cycas revolute* and *Ginkgo biloba* were set as the outgroup. The 26 completed chloroplast genome sequences representing the lineages of angiosperms were downloaded from the NCBI Organelle Genome Resource database. The protein-coding sequences of related *Lauraceae* species were determined by MUSCLE (Edgar, 2004). A phylogenetic tree was constructed based on a neighbor joining analysis. The bootstrap probability of each branch was calculated by 1,000 replications.

## Results and Discussion

### Genome organization and gene features

**Figure 1:**
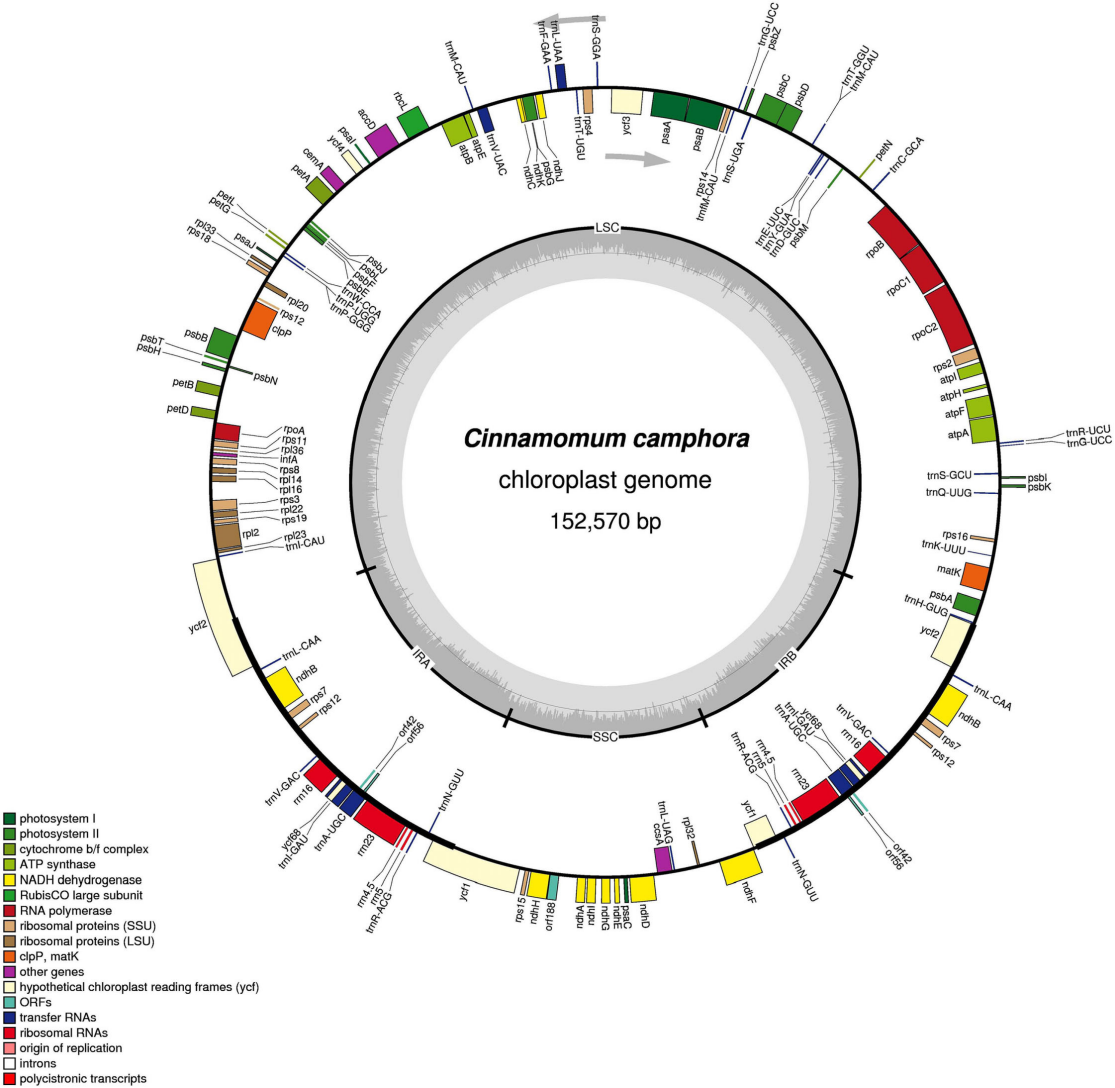
Gene map of the *Cinnamomum camphora* chloroplast genome. Genes lying inside of the molecule are transcribed in the clockwise direction, where as those outside are transcribed in the counterclockwise direction. Genes are color-coded based on their functional category. The innermost circle denotes the GC content across the genome. The dark gray inner circle corresponds to the GC content, and the light gray corresponds to the AT content of the genome. Large single copy (LSC), small single copy (SSC) and inverted repeat (IRA and IRB) regions are indicated.

The complete chloroplast genome size of *Cinnamomum camphora* was 152,570 bp, the same as *Cinnamomum micranthum* (Wu, Ho & Chang, 2016). The chloroplast genome had a single circular chromosome with a quadripartite structure, which included a pair of IR regions (19,886 bp) that were separated by a large single copy (LSC, 93,705 bp) and a small single copy (SSC, 19,093 bp) regions (Fig. 1). Coding regions (83,429 bp), comprising protein-coding genes (72,222 bp), tRNA genes (2,271 bp) and rRNA genes (8,936 bp) accounted for 54.68% of the genome, whereas non-coding regions (69,141 bp) accounted for the remaining 45.32% of the genome. The overall GC content of the *Cinnamomum camphora* was 39.13%. The IR regions had a higher GC content of 44.42%, while the GC contents of the LSC and SSC were 37.96% and 33.92%, respectively. The high GC percentage in the IR regions was similar to most reported chloroplast genomes, which could be the result of ribosomal RNA in this region (Asaf et al., 2016; Qian et al., 2013).

A total of 123 coding regions were identified in the chloroplast genome of *Cinnamomum camphora*, of which 107 were unique (Fig. 1 and Table 1). In total, 79 (73 unique) protein-coding genes were involved in the processes related to photosynthesis, the genetic system and several currently unknown functions. Additionally, 36 (30 unique) genes encoded tRNAs and eight (four unique) rRNA genes. Like the genes, the introns of chloroplast genomes were basically conserved. In *Cinnamomum camphora*, seven protein-coding genes (*rps16, petD, ndhB* (2×), *atpF*, *rpoC1* and *rpl2*) contain one intron, and four protein-coding genes (*ycf3, clpP* and *rps12* (2×)) contain two introns (Table S1). The loss of introns within the protein-coding genes reported in other plant species has not been found in the chloroplast genome of *Cinnamomum camphora* (Daniell et al., 2008; Jansen et al., 2008; Wu et al., 2009). As in many other land plants, *rps12* was located with a single 5′ end in the LSC region and a repeated 3′ end in both of the IR regions (Raman & Park, 2016; Redwan, Saidin & Kumar, 2015; Yang et al., 2013). There were 15 genes duplicated in the IR regions, including three protein-coding genes, eight tRNAs and four rRNAs. Overall, the gene content, number and structure in chloroplast genomes were generally similar to those of other reported *Lauraceae* species (Hinsinger & Strijk, 2016; Song et al., 2015, 2016; Wu, Ho & Chang, 2016).

**Table 1:** List of genes in the chloroplast genome of *Cinnamomum camphora*.

	Groups of genes	Names of genes
Protein synthesis and DNA replication	Transfer RNAs	*trnC-GCA, trnD-GUC, trnE-UUC, trnF-GAA, trnG-UCC, trnfM-CAU, trnH-GUG, trnI-CAU, trnK-UUU, trnL-UAA, trnL-UAG, trnA-UGC, trnP-GGG, trnP-UGG, trnQ-UUG, trnR-UCU, trnS-GCU, trnS-GGA, trnS-UGA, trnT-GGU, trnT-UGU, trnV-UAC, trnW-CCA, trnY-GUA, trnI-GAU* (2*×*)*, trnL-CAA* (2×), *trnM-CAU* (2×), *trnN-GUU* (2×), *trnR-ACG* (2×), *trnV-GAC* (2×)
	Ribosomal RNAs	*rrn16* (2*×*)*, rrn23* (2*×*)*, rrn4.5* (2*×*)*, rrn5* (2*×*)
	Ribosomal protein small subunit	*rps11, rps12* (2*×*)*, rps14, rps15, rps16, rps18, rps19, rps2, rps3, rps4, rps7* (2*×*)*, rps8*
	Ribosomal protein large subunit	*rpl14, rpl16, rpl2* (2*×*)*, rpl20, rpl22, rpl23, rpl32, rpl33, rpl36*
	Subunits of RNA polymerase	*rpoA, rpoB, rpoC1, rpoC2*
Photosynthesis	Photosystem I	*psaA, psaB, psaC, psaI, psaJ*
	Photosystem II	*psbA, psbB, psbC, psbD, psbE, psbF, psbH, psbI, psbJ, psbK, psbL, psbM, psbN, psbT, psbZ*
	Cytochrome b/f complex	*petA, petB, petD, petG, petL, petN*
	ATP synthase	*atpA, atpB, atpE, atpF, atpH, atpI*
	NADH-dehydrogenase	*ndhA, ndhB* (2×)*, ndhC, ndhD, ndhE, ndhF, ndhG, ndhH, ndhI, ndhJ, ndhK*
	Large subunit Rubisco	*rbcL*
Miscellaneous group	Translation initiation factor IF-1	*infA*
	Acetyl-CoA carboxylase	*accD*
	Cytochrome c biogenesis	*ccsA*
	Maturase	*matK*
	ATP-dependent protease	*clpP*
	Inner membrane protein	*cemA*
Pseudogenes of unknown function	Conserved hypothetical chloroplast open reading frame	*ycf1* (2×)*, ycf2* (2×)*, ycf3, ycf4*

The sequence analysis indicates 79 protein-coding genes in this genome represented 63,654 bp and 21,218 codons. On the basis of the sequences of protein-coding genes, the frequency of codon usage was calculated (Table 2). Among these codons 2,203 (10.87%) encode leucine, while 255 (1.25%) encode cysteine, which are the most and least used amino acids, respectively. The overall codon bias pattern in of the *Cinnamomum camphora* genome tended to use A/U—ending codons, and among the 29 preferred codons in the *Cinnamomum camphora* chloroplast genome (RSCU > 1.0), 27 ended with A/U. This phenomenon is similarly observed in many other chloroplast genomes (Nie et al., 2012; Zhang et al., 2016).

**Table 2:** Relative synonymous codon usage (RSCU) for protein coding in the chloroplast genome of *Cinnamomum camphora*.

AA	Codon	ObsFreq	RSCU	AA	Codon	ObsFreq	RSCU
	**UAA**	31	1.26	Trp	UGG	370	1
	UAG	20	0.81	Ala	**GCU**	576	1.81
	UGA	23	0.93		GCC	207	0.65
Leu	**UUA**	658	1.79		**GCA**	352	1.1
	**UUG**	463	1.26		GCG	140	0.44
	**CUU**	445	1.21	Tyr	**UAU**	613	1.56
	CUC	162	0.44		UAC	171	0.44
	CUA	315	0.86	His	**CAU**	420	1.51
	CUG	160	0.44		CAC	137	0.49
Ile	**AUU**	867	1.44	Gln	**CAA**	546	1.44
	AUC	397	0.66		CAG	210	0.56
	AUA	540	0.9	Asn	**AAU**	717	1.54
	**GUU**	461	1.46		AAC	213	0.46
	GUC	165	0.52	Lys	**AAA**	704	1.46
	**GUA**	442	1.4		AAG	261	0.54
	GUG	193	0.61	Asp	**GAU**	658	1.56
Ser	**UCU**	444	1.58		GAC	184	0.44
	**UCC**	288	1.02	Glu	**GAA**	789	1.46
	**UCA**	341	1.21		GAG	189	0.54
	UCG	176	0.63	Cys	**UGU**	190	1.49
	**AGU**	344	1.22		UGC	65	0.51
	AGC	96	0.34	Arg	**CGU**	302	1.39
Pro	**CCU**	346	1.47		CGC	75	0.35
	CCC	221	0.94		**CGA**	297	1.37
	**CCA**	250	1.06		CGG	99	0.46
	CCG	122	0.52		**AGA**	379	1.75
Thr	**ACU**	430	1.55		AGG	18	0.68
	ACC	227	0.82	Gly	**GGU**	499	1.26
	**ACA**	324	1.17		GGC	189	0.48
	ACG	126	0.46		**GGA**	602	1.53
Met	AUG	513	1		GGG	288	0.73

**Notes:**

The preferred codons are in bold (RSCU > 1.0).

AA: amino acids.

### Comparative analysis of different *Lauraceae* plastomes

**Figure 2:**
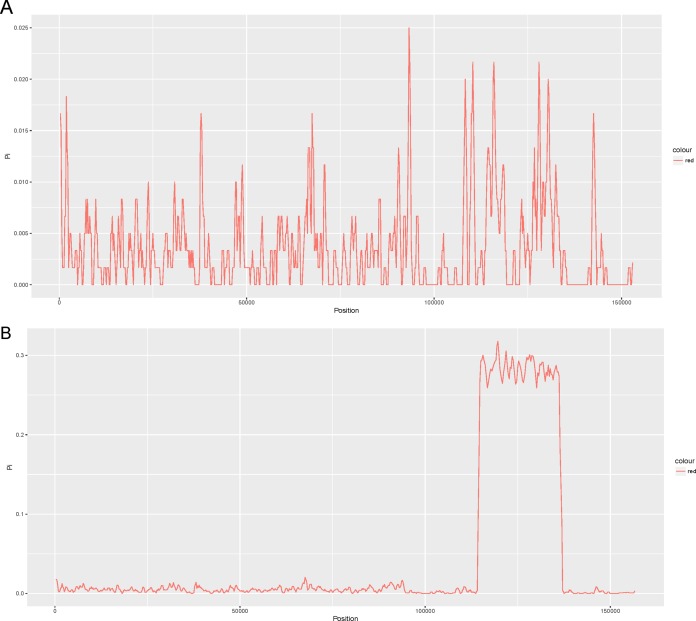
Comparison of the nucleotide variability (Pi) values of the whole plastomes in the (A) *Cinnamomum* and (B) *Lauraceae* (window length: 600 bp, step size: 200 bp). *X*-axis: positions of the midpoints of a window, *Y*-axis: nucleotide diversity in each window.

We compared four other reported chloroplast genomes of representative taxa in the *Lauraceae* family with that of *Cinnamomum camphora* (Table S2). These taxa included *Cinnamomum micranthum* (152,570 bp, KR014245.1), *Persea americana* (152,723 bp, KX437771.1), *Machilus yunnanensis* (152,721 bp, KT348516.1) and *Litsea glutinosa* (152,618 bp, KU382356.1). To examine the level of sequence divergence of the *Lauraceae* family, the Pi values within 600 bp were calculated with DnaSP 5.0 software among the five chloroplast genomes of *Cinnamomum camphora*, *Cinnamomum micranthum*, *M. yunnanensis*, *L. glutinosa* and *P. americana*. Between the two *Cinnamomum* species, Pi values varied from 0 to 0.025 (*ycf2*) with a mean of 0.00362, indicating that the differences between the two *Cinnamomum* genomes were small (Fig. 2A). However, five of the genes (*ycf2*, *rrn23*, *ycf1*, *trnL-UAG* and *ndhF*) showed high levels of variation, which were much higher than the values of other regions (Pi > 0.02). Among the five *Lauraceae* species, the Pi values ranged from 0 to 0.3178 (*ndhH*) with a mean of 0.03361, indicating that the differences among different species of the genera in the *Lauraceae* family were greater than those between congeneric species (Fig. 2B). Particularly, the entire SSC regions were highly variable among the different genera in the *Lauraceae* family. Coding regions, including those of *ycf1*, *psaC*, *ccsA*, *rpl32* and a set of genes named *ndh*, have been identified as highly variable regions in the SSC region. The SSCs often have a higher nucleotide substitution rates relative to the IRs in land plants (Perry & Wolfe, 2002; Zhang, Ma & Li, 2011). These comparatively highly variable loci are good for exploiting molecular markers and evaluating interspecies phylogenetic relationships (Chen et al., 2015; Dong et al., 2012; Timme et al., 2007).

### Repeat and SSR analyses

**Figure 3:**
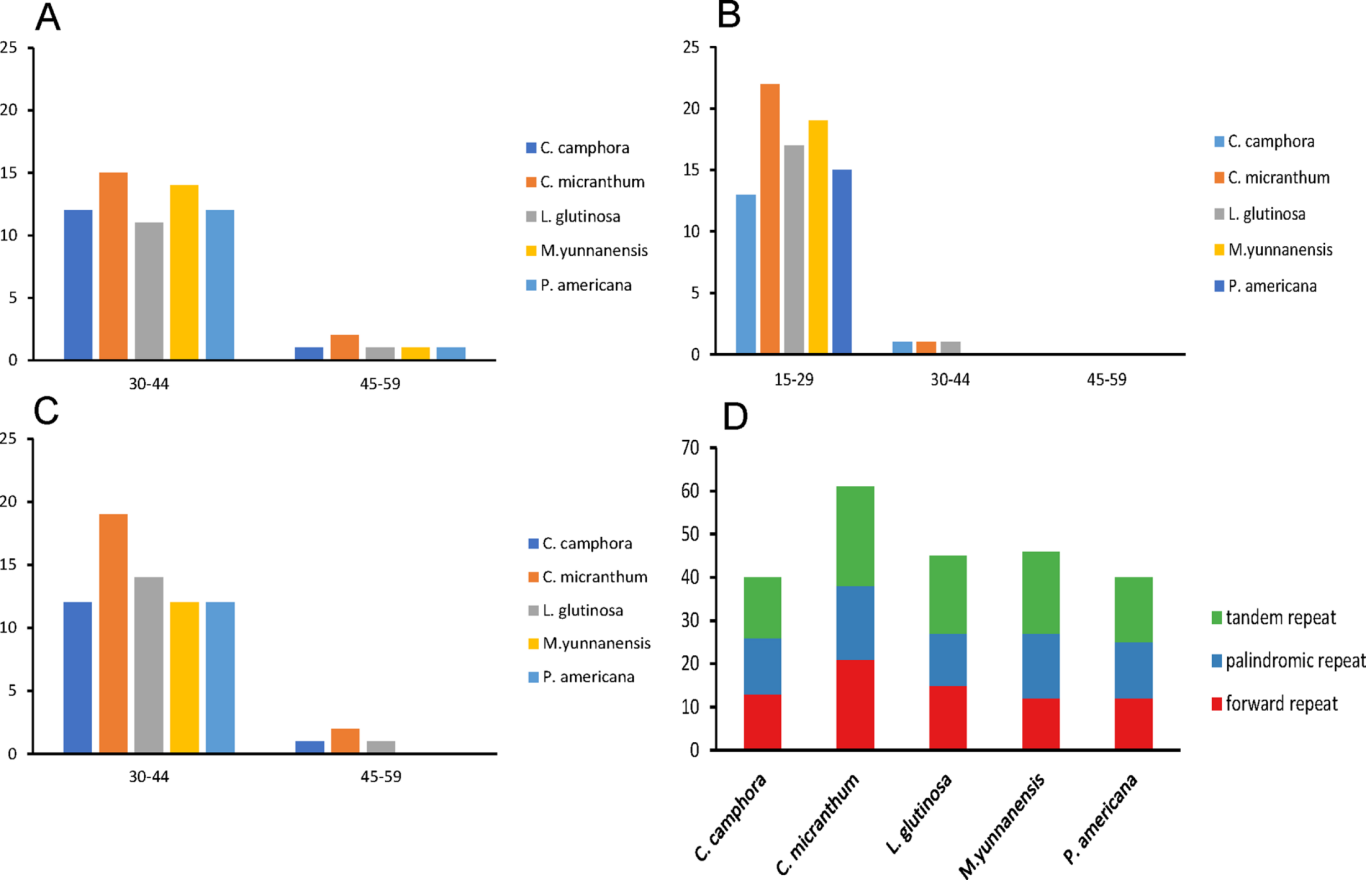
Number and type of repeated sequences in five plastomes of *Lauraceae*. (A) Frequencies of the palindromic repeats by length; (B) frequencies of the tandem repeats by length; (C) frequencies of the forward repeats by length; (D) total of the three repeat types.

Repeat motifs are very useful in the analysis of genome rearrangements and play an important role in phylogenetic analyses (Cavalier-Smith, 2002). A total of 40 repeat structures with a length at least 30 bp were detected in the chloroplast genome of *Cinnamomum camphora*. Similarly, 61, 45, 46 and 40 repeat structures were detected in *Cinnamomum micranthum*, *L. glutinosa*, *M. yunnanensis* and *P. americana* (Fig. 3D). *Cinnamomum micranthum* had the greatest total number of repeats, and the other four *Lauraceae* species showed similar numbers and patterns of repeats. For repeat analysis of *Cinnamomum camphora*, 12 forward and 12 palindromic repeats were found with a size of 30–44 bp, whereas only one forward and one palindromic repeats were 15–29 bp in length. Similarly, 13 tandem repeats were 15–29 bp, and one tandem repeat was 30–44 bp in length (Figs. 3A–3C). The presence of these repeats indicated that the region is a potential hotspot for genome reconfiguration (Asano et al., 2004; Gao et al., 2009). Additionally, these repeats were informative resources for developing genetic markers for phylogenetic and population genetic studies (Nie et al., 2012).

**Figure 4:**
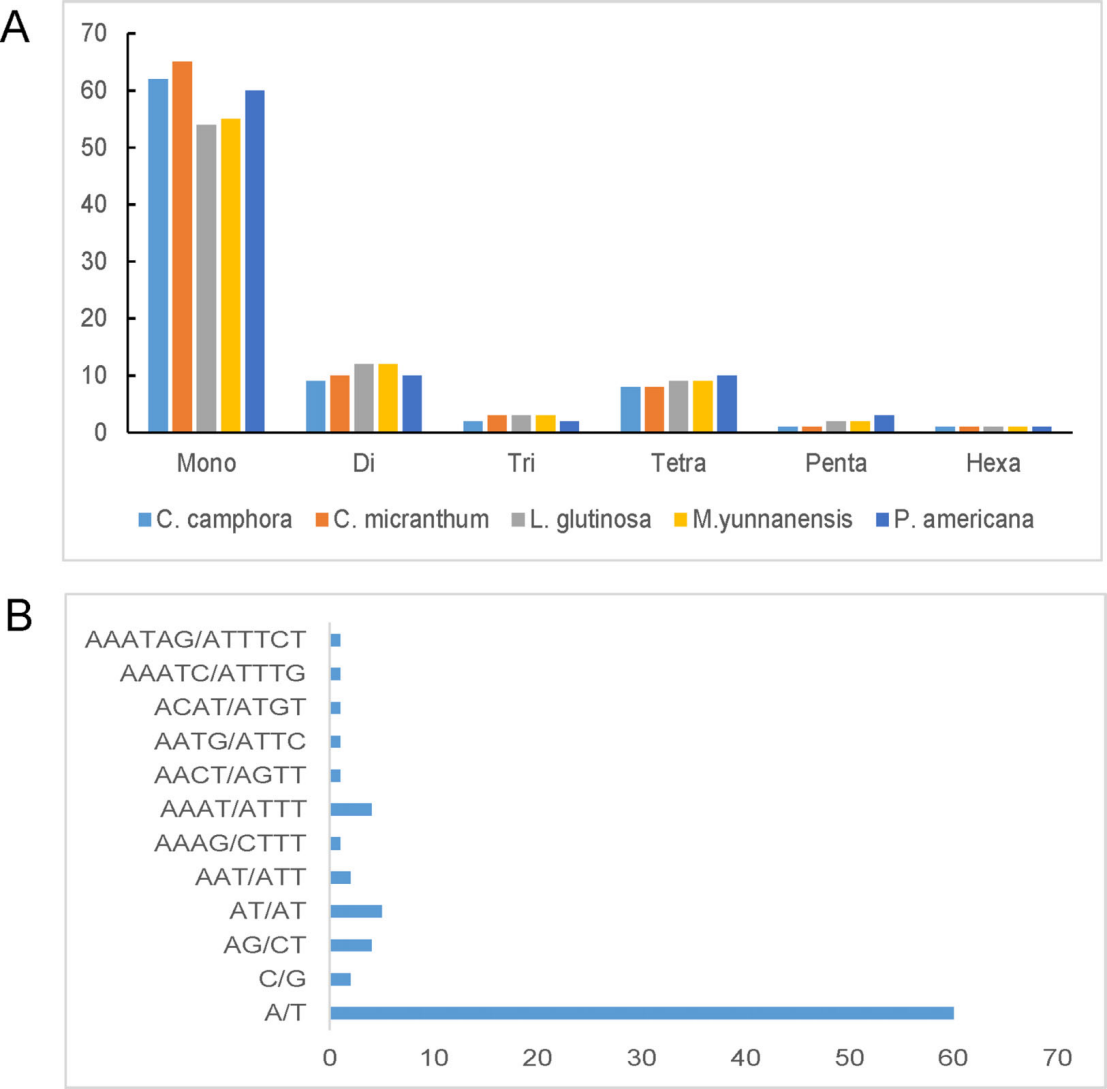
Number and type of simple sequence repeats (SSRs) in five plastomes of *Lauraceae*. (A) Numbers of different SSR types identified in the five *Lauraceae* chloroplast genomes and (B) frequencies of identified SSR motifs in different repeat class types. SSRs were classified as the number of repeat unit lengths.

Simple sequence repeats, also known as microsatellites, are 1–6 bp repeating sequences that are widely distributed throughout the chloroplast genome. SSRs are typically co-dominant and have a higher degree of polymorphism. Here, we detected perfect SSRs with a minimum size of 10 bp in *Cinnamomum camphora* and four other *Lauraceae* species. The SSRs were interrupted by a maximum distance of 100 bp. Based on the analysis, 83 perfect SSRs were detected in the chloroplast genome of *Cinnamomum camphora*. Similarly, 88, 81, 82 and 86 were identified in *Cinnamomum micranthum*, *L. glutinosa*, *M. yunnanensis* and *P. americana* (Fig. 4A). The majority of the SSRs in these chloroplast genomes were mononucleotides, ranging from 54 in *L. glutinosa* to 65 in *Cinnamomum micranthum*. Additionally, only one hexanucleotide was present in all of the *Lauraceae* species. In *Cinnamomum camphora*, the most abundant motif, at 74.50%, was a run of mononucleotide A/T (Fig. 4B). This result confirmed that the chloroplast SSRs found in most plants are generally composed of polythymine or polyadenine repeats, and infrequently contain cytosine and guanine repeats (Choi, Chung & Park, 2016; Kuang et al., 2011). The total output consisted of 83 SSRs: 76% (63 SSRs) in the LSC region, 19% (16 SSRs) in the SSC region, and 5% (four SSRs) in the IR regions (Table S3). In comparison with the IR region, the SSRs were more prevalent in the LSC and SSC regions. SSRs in coding regions are prone to mutation and cause frame-shifts to occur, which renders the gene non-functional (Wang, Barkley & Jenkins, 2009). Chloroplast SSRs have been used to evaluate genetic variations among plant genotypes (Vendramin et al., 1999) and to investigate the genetic diversity of *Lauraceae* species (Santos, Spironello & Sampaio, 2008; Zhai et al., 2010). The SSRs in this analysis are good resource for developing molecular markers and will be applied to molecular marker-assisted breeding, population genetics and genetic linkage map construction (Deguilloux, Pemonge & Petit, 2004; Huang et al., 2014).

### Phylogenetic analysis

**Figure 5:**
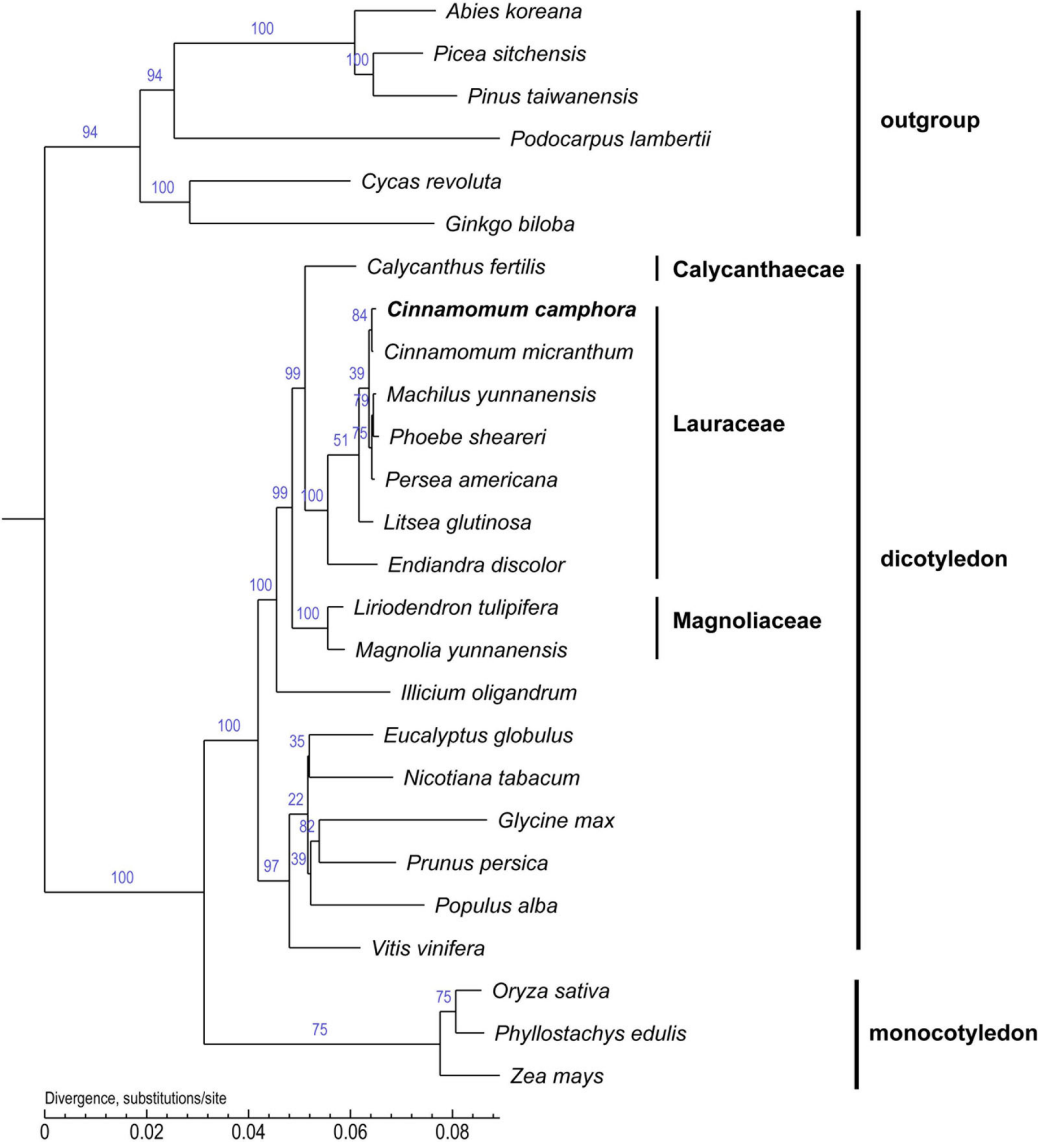
Molecular phylogenetic tree of 26 species based on a neighbor joining analysis. Numbers above and below nodes are bootstrap support values ≥50%.

Chloroplast genome sequences are useful for deciphering phylogenetic relationships among closely related taxa and for clarifying the evolutionary patterns of plant species (Jansen et al., 2007; Kyunghee et al., 2015). To examine the phylogenetic position of *Cinnamomum camphora* in the *Lauraceae* family, the core protein-coding genes that are common to all 26 chloroplasts were used to infer their phylogenetic relationships (Fig. 5). Several species in the gymnosperm were set as outgroups. The alignment analysis was conducted by MUSCLE. A neighbor joining analysis was performed with TreeBeST using 1,000 bootstrap replicates. The long branches indicated faster rates of plastid sequence evolution compared with other members in the tree. The tree suggested the correct phylogeny inference, followed by the latest angiosperm phylogeny group III (Bremer et al., 2009). The phylogenetic tree indicated that *Calycanthus* is most closely related to *Lauraceae*, which are both members of *Laurales* and *Laurales* forms a sister group to *Magnoliids*. In corroboration with other studies, *Endiandra discolor* were first separated from the other *Lauraceae* species (Hinsinger & Strijk, 2016; Rohwer & Rudolph, 2005). Furthermore, the position of *Cinnamomum camphora* was clustered with *Cinnamomum micranthum*, both of which are members of *Cinnamomum*. The chloroplast genome of *Cinnamomum camphora* will provide valuable and essential genetic information to further the phylogenetic resolution among angiosperms (Leebens-Mack et al., 2005; Moore et al., 2007; Ruhfel et al., 2014).

## Conclusion

We successfully assembled, annotated and analyzed the complete chloroplast sequence of *Cinnamomum camphora*. As an ancient tree species, the chloroplast genome of *Cinnamomum camphora*is was still conserved and found to be very similar to its sister taxon, *Cinnamomum micranthum*. The repeat sequences identified in *Cinnamomum camphora* could be selected for developing markers, population studies and phylogenetic analyses. A phylogenetic analysis suggested that *Calycanthus* is most related to *Lauraceae*, with both of them being members of *Laurales*, which forms a sister group to *Magnoliids*. The availability of the *Cinnamomum camphora* chloroplast genome will aid in for further investigations of this woody plant and will also, in conjunction with previously published chloroplast genome sequences, help to expand our understanding of the evolutionary history of *Lauraceae* chloroplast genomes, including the position of *Cinnamomum camphora* in plant systematics and evolution. In addition, it will assist in making other molecular biology applications, such as chloroplast gene transformation, feasible.

## Supplemental Information

10.7717/peerj.3820/supp-1 Supplemental Information 1 Table S1. The lengths of introns and exons for genes in the *C. camphora* chloroplast genome.

10.7717/peerj.3820/supp-2 Supplemental Information 2 Table S2. Comparison of chloroplast genome characteristics of *C. camphora* and four species of *Lauraceae*.

10.7717/peerj.3820/supp-3 Supplemental Information 3 Table S3. SSRs for *Cinnamomum camphora* chloroplast genome.

10.7717/peerj.3820/supp-4 Supplemental Information 4 MF156716-sequences.

10.7717/peerj.3820/supp-5 Supplemental Information 5 MF156716-sequences.

## Additional Information and Declarations

### DNA Deposition

The following information was supplied regarding the deposition of DNA sequences: NCBI: MF156716 The sequences have been provided as Supplemental Dataset Files.
