# Supplementary material for: The complete chloroplast genome of Cinnamomum camphora and its comparison with related Lauraceae species
Source: PeerJ. 2017 Sep 18;5:e3820. doi: 10.7717/peerj.3820 (PMC5609524; doi:10.7717/peerj.3820)
Supplement: Supplemental Information 1 — The lengths of introns and exons for genes in the C. camphora chloroplast genome. [file peerj-05-3820-s001.docx]

**Table S1**. The lengths of introns and exons for genes in the *C. camphora* chloroplast genome

| **Gene** | **Start** | **End** | **Exon I** | **Exon II** | **Exon III** |
| --- | --- | --- | --- | --- | --- |
| *rps16* | 5170 | 6314 | 40 | 255 |  |
| *atpF* | 13055 | 15335 | 143 | 410 |  |
| *rpoC1* | 21860 | 24630 | 446 | 1619 |  |
| *ycf3* | 44705 | 46677 | 125 | 227 | 167 |
| *clpP* | 73557 | 75592 | 68 | 290 | 245 |
| *petD* | 80102 | 81300 | 6 | 481 |  |
| *rpl2* | 107533 | 109021 | 389 | 431 |  |
| *ndhB* | 97847 | 98602 | 755 | 773 |  |
| *ndhA* | 119919 | 122137 | 563 | 539 |  |
| *ndhB* | 146413 | 148597 | 773 | 755 |  |
| *rps12* | 73307 | 101724 | 113 | 230 | 26 |
| *rps12* | 73307 | 145560 | 113 | 211 | 26 |
