# Supplementary material for: The complete chloroplast genome of Cinnamomum camphora and its comparison with related Lauraceae species
Source: PeerJ. 2017 Sep 18;5:e3820. doi: 10.7717/peerj.3820 (PMC5609524; doi:10.7717/peerj.3820)
Supplement: Supplemental Information 2 — Comparison of chloroplast genome characteristics of C. camphora and four species of Lauraceae. [file peerj-05-3820-s002.docx]

**Table S2*.*** Comparison of chloroplast genome characteristics of *C.camphora* and four species of L*auraceae*

|  | **C. camphora** | **C. micranthum** | **M. yunnanensis** | **P. americana** | **L. glutinosa** |
| --- | --- | --- | --- | --- | --- |
| Genome size | 152570 | 152700 | 152622 | 152723 | 152618 |
| Large single copy | 93705 | 93642 | 93676 | 93795 | 93690 |
| Small single copy | 19093 | 18844 | 18897 | 18824 | 18802 |
| Inverted repeat | 19886 | 20107 | 20074 | 20052 | 20063 |
| GC content | 39.13% | 39.10% | 39.15% | 39.00% | 39.20% |
| SSRs | 83 | 88 | 82 | 86 | 81 |
| protein-coding genes | 79 | 79 | 79 | 79 | 83 |
| tRNAs | 36(30) | 36 | 36 | 36 | 36 |
| rRNAs | 8(4) | 8 | 8 | 8 | 8 |
