# Supplementary material for: The complete chloroplast genome of Cinnamomum camphora and its comparison with related Lauraceae species
Source: PeerJ. 2017 Sep 18;5:e3820. doi: 10.7717/peerj.3820 (PMC5609524; doi:10.7717/peerj.3820)
Supplement: Supplemental Information 3 — SSRs for Cinnamomum camphora chloroplast genome. [file peerj-05-3820-s003.docx]

**Table S3.** SSRs for *Cinnamomum camphora* chloroplast genome

| **SSR type** | **SSR** | **size** | **start** | **end** | **Region** |
| --- | --- | --- | --- | --- | --- |
| mono- | (A)13 | 13 | 224 | 236 | LSC |
| mono- | (T)12 | 12 | 4607 | 4618 | LSC |
| mono- | (T)10 | 10 | 5069 | 5078 | LSC |
| mono- | (T)10 | 10 | 5189 | 5198 | LSC |
| mono- | (A)20 | 20 | 5596 | 5615 | LSC |
| mono- | (T)11 | 11 | 5977 | 5987 | LSC |
| mono- | (T)11 | 11 | 10568 | 10578 | LSC |
| mono- | (T)12 | 11 | 10674 | 10685 | LSC |
| mono- | (A)17 | 17 | 13018 | 13034 | LSC |
| mono- | (A)14 | 14 | 14125 | 14138 | LSC |
| mono- | (A)11 | 11 | 15127 | 15137 | LSC |
| mono- | (T)10 | 10 | 15616 | 15625 | LSC |
| mono- | (T)11 | 11 | 19565 | 19575 | LSC |
| di- | (AT)5 | 10 | 20938 | 20947 | LSC |
| mono- | (T)10 | 10 | 23984 | 23993 | LSC |
| mono- | (T)10 | 10 | 29810 | 29819 | LSC |
| mono- | (T)12 | 12 | 30500 | 30511 | LSC |
| mono- | (A)10 | 10 | 31903 | 31912 | LSC |
| mono- | (G)10 | 10 | 31913 | 31922 | LSC |
| tetra- | (CATA)3 | 12 | 32421 | 32432 | LSC |
| mono- | (A)11 | 11 | 32624 | 32634 | LSC |
| mono- | (T)13 | 13 | 33293 | 33305 | LSC |
| di- | (TA)7 | 14 | 34696 | 34709 | LSC |
| mono- | (A)14 | 14 | 38901 | 38914 | LSC |
| mono- | (A)11 | 11 | 39571 | 39581 | LSC |
| mono- | (T)12 | 12 | 44178 | 44189 | LSC |
| mono- | (T)12 | 12 | 44995 | 45006 | LSC |
| tetra- | (TAAA)3 | 12 | 46518 | 46530 | LSC |
| mono- | (A)10 | 10 | 46533 | 46542 | LSC |
| mono- | (A)11 | 11 | 46784 | 46794 | LSC |
| mono- | (A)11 | 23 | 47796 | 47806 | LSC |
| mono- | (G)10 | 23 | 47809 | 47818 | LSC |
| tetra- | (AACT)3 | 12 | 48898 | 48909 | LSC |
| mono- | (A)11 | 11 | 49100 | 49110 | LSC |
| di- | (TC)6 | 12 | 53054 | 53065 | LSC |
| mono- | (T)15 | 15 | 53066 | 53080 | LSC |
| mono- | (T)10 | 10 | 54561 | 54570 | LSC |
| mono- | (T)10 | 10 | 55437 | 55446 | LSC |
| mono- | (T)10 | 10 | 55500 | 55509 | LSC |
| hexa- | (TTCTAT)3 | 18 | 64307 | 64324 | LSC |
| mono- | (A)10 | 10 | 64356 | 64365 | LSC |
| di- | (TC)5 | 10 | 64545 | 64554 | LSC |
| tetra- | (AATG)3 | 12 | 65155 | 65166 | LSC |
| mono- | (T)12 | 12 | 66555 | 66566 | LSC |
| mono- | (T)11 | 11 | 68717 | 68727 | LSC |
| di- | (AT)6 | 12 | 71542 | 71553 | LSC |
| mono- | (T)10 | 10 | 72063 | 72072 | LSC |
| mono- | (A)14 | 14 | 72741 | 72754 | LSC |
| mono- | (T)14 | 14 | 74150 | 74163 | LSC |
| tri- | (TAT)4 | 12 | 74377 | 74388 | LSC |
| mono- | (A)11 | 11 | 74826 | 74836 | LSC |
| mono- | (T)13 | 13 | 75141 | 75153 | LSC |
| tetra- | (TTTC)3 | 12 | 81449 | 81460 | LSC |
| mono- | (T)12 | 12 | 84111 | 84122 | LSC |
| tetra- | (TTAT)3 | 12 | 84132 | 84143 | LSC |
| mono- | (T)13 | 13 | 84611 | 84623 | LSC |
| mono- | (T)11 | 11 | 86180 | 86190 | LSC |
| di- | (TC)7 | 14 | 86935 | 86948 | LSC |
| mono- | (T)10 | 10 | 87781 | 87790 | LSC |
| di- | (TA)5 | 10 | 88350 | 88359 | LSC |
| mono- | (T)10 | 10 | 89353 | 89362 | LSC |
| mono- | (A)10 | 10 | 92027 | 92036 | LSC |
| di- | (GA)6 | 12 | 93226 | 93237 | LSC |
| tetra- | (TTTA)3 | 12 | 102116 | 102127 | IRA |
| mono- | (A)10 | 10 | 113318 | 113327 | IRA |
| mono- | (T)11 | 11 | 114666 | 114676 | SSC |
| mono- | (A)10 | 10 | 115286 | 115295 | SSC |
| mono- | (A)10 | 10 | 115339 | 115348 | SSC |
| mono- | (A)10 | 10 | 115595 | 115604 | SSC |
| mono- | (A)10 | 10 | 116873 | 116882 | SSC |
| penta- | (TTTGA)3 | 15 | 118199 | 118213 | SSC |
| tri- | (ATT)4 | 12 | 118285 | 118296 | SSC |
| mono- | (A)16 | 16 | 118322 | 118337 | SSC |
| mono- | (T)14 | 14 | 121384 | 121397 | SSC |
| mono- | (A)10 | 10 | 126380 | 126389 | SSC |
| mono- | (T)12 | 12 | 128451 | 128462 | SSC |
| mono- | (T)16 | 16 | 128853 | 128868 | SSC |
| mono- | (T)12 | 12 | 129466 | 129477 | SSC |
| di- | (AT)7 | 14 | 129644 | 129657 | SSC |
| mono- | (T)11 | 11 | 130235 | 130245 | SSC |
| mono- | (T)10 | 10 | 130322 | 130331 | SSC |
| mono- | (T)10 | 10 | 133337 | 133346 | IRB |
| tetra- | (AAAT)3 | 12 | 144418 | 144429 | IRB |
