# Supplementary material for: The complete chloroplast genome of Cinnamomum camphora and its comparison with related Lauraceae species
Source: PeerJ. 2017 Sep 18;5:e3820. doi: 10.7717/peerj.3820 (PMC5609524; doi:10.7717/peerj.3820)
Supplement: Supplemental Information 4 [file peerj-05-3820-s004.docx]

LOCUS Cinnamomum 152570 bp DNA circular 16-MAY-2017

DEFINITION Cinnamomum camphora chloroplast, complete genome.

ACCESSION

VERSION

KEYWORDS .

SOURCE chloroplast Cinnamomum camphora

ORGANISM Cinnamomum camphora

Eukaryota; Viridiplantae; Streptophyta; Embryophyta;

Tracheophyta;Spermatophyta; Magnoliophyta; Magnoliidae; Laurales;

Lauraceae;Cinnamomum.

REFERENCE 1 (bases 1 to 152570)

AUTHORS Chen,C.

TITLE direct submission

JOURNAL Unpublished

REFERENCE 2 (bases 1 to 152570)

AUTHORS Chen,C.

TITLE Direct Submission

JOURNAL Submitted (16-MAY-2017) Nanjing Forestry University, Co-Innovation

Center for Sustainable Forestry in Southern China, Longpan Street

No.159, Nanjing, Jiangsu 210037, China

FEATURES Location/Qualifiers

source 1..152570

/organism="Cinnamomum camphora"

/organelle="plastid:chloroplast"

/mol_type="genomic DNA"

CDS complement(join(100931..100957,101494..101724,

73307..73420))

/gene="rps12"

/trans_splicing

/codon_start=1

/transl_table=11

/product="ribosomal protein S12"

/translation="MPTIKQLIRNTRQPIRNVTKSPALRGCPQRRGTCTRVYTITPKK

PNSALRKVARVRLTSGFEITAYIPGIGHNSQEHSVVLVRGGRVKDLPGVRYHIVRGTL

DAVGVKDRQQGRSQYGVKKPK"

misc_feature 1..93705

/note="LSC"

gene complement(30..104)

/gene="trnH-GUG"

tRNA complement(30..104)

/gene="trnH-GUG"

/product="tRNA-His"

/note="anticodon:GUG"

gene complement(499..1560)

/gene="psbA"

CDS complement(499..1560)

/gene="psbA"

/codon_start=1

/transl_table=11

/product="photosystem II protein D1"

/translation="MTAILERRESTSLWGRFCNWITSTENRLYIGWFGVLMIPTLLTA

TSVFIIAFIAAPPVDIDGIREPVSGSLLYGNNIISGAIIPTSAAIGLHFYPIWEAASV

DEWLYNGGPYELIVLHFLLGVACYMGREWELSFRLGMRPWIAVAYSAPVAAATAVFLI

YPIGQGSFSDGMPLGISGTFNFMIVFQAEHNILMHPFHMLGVAGVFGGSLFSAMHGSL

VTSSLIRETTENESANAGYRFGQEEETYNIVAAHGYFGRLIFQYASFNNSRSLHFFLA

AWPVVGIWFTALGISTMAFNLNGFNFNQSVVDSQGRVINTWADIINRANLGMEVMHER

NAHNFPLDLAAVEVPSTNG"

gene complement(2082..3626)

/gene="matK"

CDS complement(2082..3626)

/gene="matK"

/codon_start=1

/transl_table=11

/product="maturase K"

/translation="MEELQGYLEMDGFRQQYFLYPFLFQEYIYALAHGHALNGSILYE

PVENLDHDNKSSSLIVKRLITRMHQQNRLIISVNDSNQNRFVGHNNHFDSQMISEGFA

VVVEIPFSLRLVSSLEEKEIAKSHNLRSIHSIFPFFEDKLSHLNHVSDILIPHPIHLE

ILVQTLHSWIQDTPSLHLLRFSLYEYWNSNSLITPKNSISLFSKENQRFFLFLSNSHV

YECEFIFIFLRKQPFHLRSKSFGSFLERTHFYAKIEYLVVVLCNDFQKTLWLFKDPFM

HYVRYQGKSILASRGARLLIKKWKSHLVNFWQCHFYLWSQPARIHIKQLYNHPFYFLG

YLSSVRLNSSVIRSQMLENSFRIDTAIKKFETVVPIIPLIGSLAKAKFCNVSGHPISK

PFRADLSDSEILNRFGRICRNLSHYHSGSSKKQSLYRIKYILRLSCARTLSRKHKSTI

RAFLKRLGSEFLEEFFTEEEQALSLIFPTTSSPSHRSHRERIWYLDIIRINDLVSHLM

IGHEVM"

gene complement(4344..4380)

/gene="trnK-UUU"

tRNA complement(4344..4380)

/gene="trnK-UUU"

/product="tRNA-Lys"

/note="anticodon:UUU"

gene complement(5170..6314)

/gene="rps16"

exon complement(5170..5425)

/gene="rps16"

/number=2

CDS complement(join(5197..5425,6274..6314))

/gene="rps16"

/codon_start=1

/transl_table=11

/product="ribosomal protein S16"

/translation="MVKLRLKRCGRKQRVIYRIVAIDVRSRREGRDLRKVGFYDPIKN

QTYSNVSAILYFLEKGAQPTGTVHDISKKAEVFKEFRINQMKLMK"

exon complement(6274..6314)

/gene="rps16"

/number=1

gene complement(8207..8279)

/gene="trnQ-UUG"

tRNA complement(8207..8279)

/gene="trnQ-UUG"

/product="tRNA-Gln"

/note="anticodon:UUG"

gene 8615..8794

/gene="psbK"

CDS 8615..8794

/gene="psbK"

/codon_start=1

/transl_table=11

/product="photosystem II protein K"

/translation="MLNIFSLICLNSALHSSSFFFAKLPEAYAFFNPIVDVMPVIPVL

FFLLALVWQAAVSFR"

gene 9178..9288

/gene="psbI"

CDS 9178..9288

/gene="psbI"

/codon_start=1

/transl_table=11

/product="photosystem II protein I"

/translation="MLTLKLFVYTVVIFFVSLFIFGFLSNDPGRNPGRDE"

gene complement(9427..9514)

/gene="trnS-GCU"

tRNA complement(9427..9514)

/gene="trnS-GCU"

/product="tRNA-Ser"

/note="anticodon:GCU"

gene 11116..11152

/gene="trnG-UCC"

tRNA 11116..11152

/gene="trnG-UCC"

/product="tRNA-Gly"

/note="anticodon:UCC"

gene 11278..11349

/gene="trnR-UCU"

tRNA 11278..11349

/gene="trnR-UCU"

/product="tRNA-Arg"

/note="anticodon:UCU"

gene complement(11462..12985)

/gene="atpA"

CDS complement(11462..12985)

/gene="atpA"

/codon_start=1

/transl_table=11

/product="ATP synthase CF1 alpha subunit"

/translation="MVTIRADEISNIIRERIEQYNREVKIVNTGTVLQVGDGIARIHG

LDEVMAGELVEFEEGTIGIALNLESNNVGVVLMGDGLMIQEGSSVKATGRIAQIPVSE

AYLGRVINALAKPIDGRGEISASESRLIESPAPGIISRRSVYEPLQTGLIAIDSMIPI

GRGQRELIIGDRQTGKTAVATDTILNQKGQNVICVYVAIGQKASSVAQVVTTFQERGA

MEYTIVVAETADSPATLQYLAPYTGAALAEYFMYRERHTSIIYDDLSKQAQAYRQMSL

LLRRPPGREAYPGDVFYLHSRLLERAAKSSSRLGEGSMTALPIVETQSGDVSAYIPTN

VISITDGQIFLSADLFNAGIRPAINVGISVSRVGSAAQIKAMKQVAGKSKLELAQFAE

LEAFAQFASDLDKATQNQLARGQRLRELLKQSQSAPLTVEEQIVTIYTGANGYLDPLE

IGQVKKFLVQLRTYLKTNKPQVQEIISSTKTFTAQAEALLKEAIPEQIELFLLQEQK"

gene complement(13055..14335)

/gene="atpF"

CDS complement(join(13055..13465,14192..14335))

/gene="atpF"

/codon_start=1

/transl_table=11

/product="ATP synthase CF0 subunit I"

/translation="MRDVTDSFVSFGHWPSAGSFGFNTDILATNLINLSVVLGVLIFF

GKGVLSDLLDNRKQRILSTIRNSEELREGAIEQLEEARARLRKVEIEADEFRVNGYSE

IEREKWNLINATYENLERLENYKNETIHFEQQRAINQVRQRVFQQALQGALGTLNSCS

NSELHLRTIGANIGMLGAMKEVTD"

exon complement(13055..13465)

/gene="atpF"

/number=2

exon complement(14192..14335)

/gene="atpF"

/number=1

gene complement(14788..15033)

/gene="atpH"

CDS complement(14788..15033)

/gene="atpH"

/codon_start=1

/transl_table=11

/product="ATP synthase CF0 subunit III"

/translation="MNPLISAASVIAAGLAVGLASIGPGVGQGTAAGQAVEGIARQPE

AEGKIRGTLLLSLAFMEALTIYGLVVALALLFANPFV"

gene complement(15698..16441)

/gene="atpI"

CDS complement(15698..16441)

/gene="atpI"

/codon_start=1

/transl_table=11

/product="ATP synthaseCF0 subunit IV"

/translation="MNVLPCSINSLKALYDISDVEVGQHFYWQIGGFQVHAQVLITSW

VVIAILLGSATIAVRNPQTIPTDGQNFFEYVLEFIRDLSKTQIGEEYGPWVPFIGTMF

LFIFVSNWSGALLPRKIIQLPHGELAAPTNDINTTVALALPTSVAYFYAGLTKKGLGY

FGKYIQPTPILLPINILEDFTKPLSLSFRLFGNILADELVVVVLVSLVPSVVPIPVMF

LGLFTSGIQALIFATLAAAYIGESMEGHH"

gene complement(16649..17368)

/gene="rps2"

CDS complement(16658..17368)

/gene="rps2"

/codon_start=1

/transl_table=11

/product="ribosomal protein S2"

/translation="MPRRYWNMNFEEMVKAGVHFGHGTRKWNPRMAPYISSKRKGIHI

TNLTRTARFLSEACDLVFDAASIGKHFLIVGTKNKAANLVASAAIRARCHYVNKKWLG

GMLTNWSTTEMRLHRFRNLRSEQNTGKLNCLPKRDVAMLKRQLSHLQTYLGGIKYMTG

LPDIVIIVGQQEEYTALRECLTLRIPTICLIDTNCDPDLANIPIPANDDAMASIRWIL

NKLVSAICEGSSSYIRNC"

gene complement(17541..21707)

/gene="rpoC2"

CDS complement(17541..21698)

/gene="rpoC2"

/codon_start=1

/transl_table=11

/product="RNA polymerase beta' subunit"

/translation="MAEWADLFFYNKAIDGSAMKRLISRLIDHFGMAYTSHTLDQVKT

LGFQQATATSISLGIDDLLTVPSKGWLVQDAEQQSFILEKHHHYGNVHTVEKLRQSIE

IWYATSEYLRQEMHPNFRMTDPSNSVHIMSFSGARGNASQVHQLVGMRGLMSDPQGQM

IDLPIQSNLREGLSLTEYIISCYGARKGVVDTAVRTSDAGYLTRRLVEVVQHIVVRRT

DCGTTRGISVSPRNGMTERIWVQTLIGRVLAYNIYMGPRCIAAQNKDIGVGLVTRFIT

FRTQPIYIRTPFLCRSISWICRLCYGQSPTHGDLVELGEAVGIIAGQSIGEPGTQLTL

RTFHTGGVFTGGTAEHVRAPFKGKIKFNEDLVHPTRTRHGHPAFLCYIDLYVTIESHD

ILHNVNIPPKSFLLVQNDQYVESEQVIAEIRAGTSTFNFNKVKEKVRKHIYSDSEGEM

HWSTDVYHAPEYKYGNVHLLPKTSHLWILSGALCRSSIVPFSLHKDQDQMNVHSVERR

SISDLSVTNDQVRHKLFSSNPYGKKGGVLDYSGPDRIISNGHWNFIYPTILHENSDLL

AKRRRNRFIIPFQSDQEREKELMPRSGISIEIPINGVLPRNSILAYFDDPLYRRSSSG

ITKYGTIGVGSIVKKEDLIEYRRAKEFRPKYQMKVDRFFFIPEEVHILPGSSPIMVRN

NSIIGVDTRIALNTRSQVGGLVRVERKKKRIELKIFSGDIYFPGATDKISRHCGILIP

PGTGKKNSKESKKLKNWIYVQRITPTKKKYFVSVRPVVTYEIADGINLATLFPQDPLQ

ERDNVQLRIVNYILYGNGKPIRGIYHTSLQLVRTCLVLNWNQDRDGSIEEVHASFVEV

RANDLIRDFIRIDLVKSSIFYIGKRNDMASSGLIANNGSDRTNINPFYFKARIQSFTQ

HQGTIRTLLNRNKECPSFLILLSSDCSRIGLFNGSKSHKELIKLIKEDPAIPIRNSLG

PLGIVPQITNFYSFYYFYLITHNQILLKKYFLLDNFKHTFQGLKYYLMDENGRIYNPD

SCSNIIFNPFDLNWCFLPHDYCEETSTIISLGQFICENVCISKCGPHIKSSQVLIVHV

DSLVIRSAKPHLATPGATVHGHCGEILYEGDTLVTFIYEKSRSGDITQGLPKVEQVLE

ARSIDSISMNLEKRVEGWNERITRILGIPWGFLIGAELTIAQSRISLVNKIQKVYRSQ

GVQIHNRHIEIIVRQITSKVLVSEDGMSNVFSPGELIGLLRAERTGRALEEAICYRAI

LLGITRASLNTQSFISEASFQETARVLAKAALRGRIDWLKGLKENVVLGGMMPVGTGF

KGLVHRSRQHSNIPLEIKKKNPFEEEMRDLLFHHRELFGSCIPNNFPDTSERSFTGFN

DRFILFF"

gene complement(21860..24630)

/gene="rpoC1"

CDS complement(join(21860..23479,24199..24630))

/gene="rpoC1"

/codon_start=1

/transl_table=11

/product="RNA polymerase beta"

/translation="MIDRYKHQQLRVGSVSPQQISAWATKILPNGEMVGEVTKPYTFH

YKSNKPEKDGLFCERIFGPIKSGICACGNYRVIGEEKEDPKFCEQCGVEFVDSRIRRY

QMGYIKLACPATHVWYLKRLPSYIANLLDKPLKELEGLVYCDFSFARPIAKKPTFLRL

RGSFESEIQSRKYSIPLFFTTPGFDTFRNREISTGAGAIREQLADPDLRIIIDHSSVE

WKELGEEGFTGNEWEDRKIGRRKDFLVRRMELAKHFIRTNVEPERMVLCLLPVLPPEL

RPIIQIDGGKLMSSDINELYRRVIYRNNTLTDSLTTSRSTPGELVMCQEKLVQEAVDT

LLDNGIRGQPMRDGHNKVYKSFSDVIEGKEGRFRETLLGKRVDYSGRSVIVVGPSLSL

HRCGLPREIAIELFQTFVIRGLIRRHIASNIGIAKSQIREKEPIVWEILQEVMQGHPV

LLNRAPTLHRLGIQAFQPILGGGRAICLHPLVRKGFNADFDGDQMAVHVPLSLEAQAE

ARLLMFSHMNLFSPAIGDPISVPTQDMLIGLYVLTIGNRRGICANRYNPWNRINYQNE

TVNDYKYKYTTKEKEPYFCSSYDVLIVYQQKRINLDSPLWLRWRLDQRVIASREVPVE

VQYESLGTYHEIYGHYLIVRRIKKQTLCIYTRTTVGPISFSREIEEAIQGFCQAYSYG

T"

exon complement(21860..23488)

/gene="rpoC1"

/number=2

exon complement(24184..24630)

/gene="rpoC1"

/number=1

gene complement(24657..27839)

/gene="rpoB"

CDS complement(24657..27839)

/gene="rpoB"

/codon_start=1

/transl_table=11

/product="RNA polymerase beta subunit"

/translation="MYTIPGFSQIQFDGFCRFIDQGLMEELHKFPKIEDTDQEIEFQL

FVATYQLAEPLIKERDAVYESLTYSSELYVSAGLIWKTGRDMQEQTVFIGNIPLMNSL

GTSLVSGIYRIVINQILQSPGIYYRSELDHSGISVYTGTIISDWGGRSELEIDRKARI

WARVSRKQKISILVPSSAMGSNLREILDNVCYPEIFLSFPNHKEKKKIGSRENAILEF

YQQFACVGGDPVFSESLCKELQKKFFQQRCELGRIGRRNMNRRLNLDIPPNNTFLLPQ

DVLAAVDHLIGMKFGMGTLDDMNHLKNKRIRSVADLLQDQFGLALVRLENAVRGTICG

AIRHKLIPTPHNLVTPTPLTTTYESFFGLHPLSQVLDRTNPLTQIVHGRKLSYLGPGG

LTGRTASFRIRDIHPSHYGRICPIDTSEGINVGLIGSLAIHARIGHWGSIESPFYEIS

ERSKKMVYLSPSRDEYYMVAAGNSLALNRGIQEEQVVPAGYRQEFLTIAWEQIHLRSI

FPFQYFSIGASLIPFIEHNDANRALMSSNMQRQAVPLSRSEKCIVGTGLECQAALDSG

VSAIAEHEGKIIYTDTDKIVLSGNGDTISIPLVMYQRSNKNTCMHQKPQAPRGKCIKK

GQILADGAATVGGELTLGKNVLVAHMSWEGYNSEDAVLISERLVYGDIYTSFHIRKYE

IQTHVTSQGPERITNKIPHLEAHLLRNLDKNGIVMLGSWIERGDILVGKLTPQAAKES

SYAPEDRLLRAILGIQVSTAKETCLKLPIGGRGRVIDVRWIQKKGGSSYNPETIRVYI

LQKREIKVGDKIAGRHGNKGIVSKILPRQDMPYLQAGTPVDMVFNPLGVPSRMNVGQI

FECSLGLAGYLLDRHYRIAPFDERYEQGASRKLVFPELYSASKQTANPWVFEPEYPGK

SRILDGRTGDPFEQPVIIGKSYILKLIHQVDDKIHGRSSGHYALVTQQPLRGRAKQGG

QRVGEMEVWALEGFGVAHISQEMLTYKSDHIRARQEVLGTTIIGGTILKPEDAPESFR

LLVRELRSLALELNHFLVSEKNFQINRKEV"

gene 28984..29064

/gene="trnC-GCA"

tRNA 28984..29064

/gene="trnC-GCA"

/product="tRNA-Cys"

/note="anticodon:GCA"

gene 30111..30200

/gene="petN"

CDS 30111..30200

/gene="petN"

/codon_start=1

/transl_table=11

/product="cytochrome b6/f complex subunit VIII"

/translation="MDIVSLAWAALMVVFTFSLSLVVWGRSGL"

gene complement(31265..31369)

/gene="psbM"

CDS complement(31265..31369)

/gene="psbM"

/codon_start=1

/transl_table=11

/product="photosystem II protein M"

/translation="MEVNILAFIAIALFILVPTAFLLIIYVKTVSQSD"

gene complement(32329..32402)

/gene="trnD-GUC"

tRNA complement(32329..32402)

/gene="trnD-GUC"

/product="tRNA-Asp"

/note="anticodon:GUC"

gene complement(32716..32799)

/gene="trnY-GUA"

tRNA complement(32716..32799)

/gene="trnY-GUA"

/product="tRNA-Tyr"

/note="anticodon:GUA"

gene complement(32854..32926)

/gene="trnE-UUC"

tRNA complement(32854..32926)

/gene="trnE-UUC"

/product="tRNA-Glu"

/note="anticodon:UUC"

gene 33650..33721

/gene="trnT-GGU"

tRNA 33650..33721

/gene="trnT-GGU"

/product="tRNA-Thr"

/note="anticodon:GGU"

gene 35168..36229

/gene="psbD"

CDS 35168..36229

/gene="psbD"

/codon_start=1

/transl_table=11

/product="photosystem II protein D2"

/translation="MTIALGRFTKEENDLFDIMDDWLRRDRFVFVGWSGLLLFPCAYF

ALGGWFTGTTFVTSWYTHGLASSYLEGCNFLTAAVSTPANSLAHSLLLLWGPEAQGDF

TRWCQLGGLWTFVALHGAFGLIGFMLRQFELARSVQLRPYNAIAFSAPIAVFVSVFLI

YPLGQSGWFFAPSFGVAAIFRFILFFQGFHNWTLNPFHMMGVAGVLGAALLCAIHGAT

VENTLFEDGDGANTFRAFNPTQAEETYSMVTANRFWSQIFGVAFSNKRWLHFFMLFVP

VTGLWMSAIGVVGLALNLRAYDFVSQEIRAAEDPEFETFYTKNILLNEGIRAWMAAQD

QPHENLIFPEEVLPRGNAL"

gene 36177..37598

/gene="psbC"

CDS 36177..37598

/gene="psbC"

/codon_start=1

/transl_table=11

/product="photosystemII CP43 chlorophyll apoprotein"

/translation="MKILYSLRRFYPVETLFNGTLALAGRDQETTGFAWWAGNARLIN

LSGKLLGAHVAHAGLIVFWAGAMNLFEVAHFVPEKPMYEQGLILLPHLATLGWGVGPG

GEVIDTFPYFVSGVLHLISSAVLGFGGIYHALLGPETLEESFPFFGYVWKDRNKMTTI

LGIHLILLGIGAFLLVLKALYFGGVYDTWAPGGGDVRKISNLTLSPSVIFGYLLKSPF

GGEGWIVSVDDLEDIIGGHVWLGSICILGGIWHILTKPFAWARRAFVWSGEAYLSYSL

GALSVFGFIACCFVWFNNTAYPSEFYGPTGPEASQAQAFTFLVRDQRLGANVGSAQGP

TGLGKYLMRSPTGEVIFGGETMRFWDLRAPWLEPLRGPNGLDLSRLKKDIQPWQERRS

AEYMTHAPLGSLNSVGGVATEINAVNYVSPRSWLATSHFVLGFFLFVGHLWHAGRARA

AAAGFEKGIDRDFEPVLSMTPLN"

gene complement(37813..37905)

/gene="trnS-UGA"

tRNA complement(37813..37905)

/gene="trnS-UGA"

/product="tRNA-Ser"

/note="anticodon:UGA"

gene 38227..38415

/gene="psbZ"

CDS 38227..38415

/gene="psbZ"

/codon_start=1

/transl_table=11

/product="photosystem II protein Z"

/translation="MTIAFQLAVFALIATSSILLISVPVVFASSDGWSSNKNVVFSGT

SLWIGLVFLVAILNSLIS"

gene 38691..38761

/gene="trnG-UCC"

tRNA 38691..38761

/gene="trnG-UCC"

/product="tRNA-Gly"

/note="anticodon:UCC"

gene complement(38921..38994)

/gene="trnfM-CAU"

tRNA complement(38921..38994)

/gene="trnfM-CAU"

gene complement(39149..39451)

/gene="rps14"

CDS complement(39149..39451)

/gene="rps14"

/codon_start=1

/transl_table=11

/product="ribosomal protein S14"

/translation="MARKSLIQRERKKQKLEQKYHLIRRSSKKEIGKVSSLSDKWEIH

GKLQSPPRNSTPTRLHRRCFLTGRPRASYRDFRLSGHILHEKVQACLLPGATRSSW"

gene complement(39582..41786)

/gene="psaB"

CDS complement(39582..41786)

/gene="psaB"

/codon_start=1

/transl_table=11

/product="photosystem I P700 apoprotein A2"

/translation="MALRFPKFSQGLAQDPTTRRIWFGIATAHDFESHDDITEERLYQ

NIFASHFGQLAIIFLWTSGNLFHVAWQGNFESWVQDPLHVRPIAHAIWDPHFGQPAVE

AFTRGGALGPVNIAYSGVYQWWYTIGLRTNEDLYTGALFLLFLSAISLIAGWLHLQPK

WKPSVSWFKNAESRLNHHLSGLFGVSSLAWTGHLVHVAIPGSRGQYVRWNNFLDVLPH

PQGLGPLFTGQWNLYAQNPDSSRHLFGTSQGAGTAILTLLGGFHPQTQSLWLTDIAHH

HLAIAFIFLVAGHMYRTNFGIGHSMKDLLEAHIPPGGRLGRGHKGLYDTINNSIHFQL

GLALASLGVITSLVAQHMYSLPAYAFIAQDFTTQAALYTHHQYIAGFIMTGAFAHGAI

FFIRDYNPEQNEDNVLARMLDHKEAIKSHLSWASLFLGFHTLGLYVHNDVMLAFGTPE

KQILIEPIFAQWIQSAHGKTSYGFDVLLSSTNGPAFNAGRSIWLPGWLNAVNENSNSL

FLTIGPGDFLVHHAIALGLHTTTLILVKGALDARGSKLMPDKKDFGYSFPCDGPGRGG

TCDISAWDAFYLAVFWMLNTIGWVTFYWHWKHITLWQGNVSQFNESSTYLMGWLRDYL

WLNSSQLINGYNPFGMNSLSVWAWMFLFGHLVWAIGFMFLISWRGYWQELIETLAWAH

ERTPLANLIRWRDKPVALSIVQARLVGLAHFSVGYIFTYAAFLIASTSGKFG"

gene complement(41812..44064)

/gene="psaA"

CDS complement(41812..44064)

/gene="psaA"

/codon_start=1

/transl_table=11

/product="photosystem I P700 apoprotein A1"

/translation="MIIRSPEPEVKILVDRDPIKTSFEEWARPGHFSRTIAKGPDTTT

WIWNLHADAHDFDSHTSDLEEISRKVFSAHFGQLSIIFLWLSGMYFHGARFSNYEAWL

SDPTHIGPSAQVVWPIVGQEILNGDVGGGFRGIQITSGFFQLWRASGITNELQLYCTA

IGALVFAALMLFAGWFHYHKAAPKLAWFQDVESMLNHHLAGLLGLGSLSWAGHQVHVS

LPINQFLDAGVDPKEIPLPHEFILNRDLLAQLYPSFAEGSTPFFTLNWSKYAEFLSFR

GGLDPVTGGLWLTDIAHHHLAIAILFLVAGHMYRTNWGIGHGLKDILEAHKGPFTGQG

HKGLYEILTTSWHAQLSLNLAMLGSSTIVVAHHMYSMPPYPYLAIDYGTQLSLFTHHM

WIGGFLIVGAAAHAAIFMVRDYDPTTRYNDLLDRVLRHRDAIISHLNWVCIFLGFHSF

GLYIHNDTMSALGRPRDMFSDTAIQLQPIFAQWVQNTHALAPGATAPGATTSTSLTWG

GGDLVAVGGKVALLPIPLGTADFLVHHIHAFTIHVTVLILLKGVLFARSSRLIPDKAN

LGFRFPCDGPGRGGTCQVSAWDHVFLGLFWMYNAISVVIFHFSWKMQSDVWGSISDQG

VVTHITGGNFAQSSITINGWLRDFLWAQASQVIQSYGSSLSAYGLFFLGAHFVWAFSL

MFLFSGRGYWQELIESIVWAHNKLKVAPATQPRALSIVQGRAVGVTHYLLGGIATTWA

FFLARIIAVG"

gene complement(44705..46677)

/gene="ycf3"

CDS complement(join(44705..44857,45589..45816,46552..46677))

/gene="ycf3"

/codon_start=1

/transl_table=11

/product="photosystem I assembly protein ycf3"

/translation="MPRSRINGNFIDKTSSIVANILLRIIPTTSGEKEAFTYYRDGMS

AQSEGNYAEALQNYYEATRLEIDPYDRSYILYNIGLIHTSNGEHTKALEYYFRAIERN

PFLPQAFNNMAVICHYRGEQAIRQGDSEIAEAWSDQAAEYWKQAIALTPGNYIEAHNW

LKIARRFE"

exon complement(44708..44857)

/gene="ycf3"

/number=3

exon complement(45589..45816)

/gene="ycf3"

/number=2

exon complement(46552..46677)

/gene="ycf3"

/number=1

gene 47488..47574

/gene="trnS-GGA"

tRNA 47488..47574

/gene="trnS-GGA"

/product="tRNA-Ser"

/note="anticodon:GGA"

gene complement(47854..48459)

/gene="rps4"

CDS complement(47854..48459)

/gene="rps4"

/codon_start=1

/transl_table=11

/product="ribosomal protein S4"

/translation="MSRYRGPRFKKIRCLGALPGLTSKRPRSGSDLRNQSRFGKRSQY

RIRLEEKQKLRFHYGLTERQLLRYVRIAGKAKGSTGQVLLQLLEMRLDNILFRLGMAS

TIPGARQLVNHRHILVNGRVVDIPSYRCKPRDIITARDEQRSRALIQNYLDSSPREDL

AKHLTFDSSQYKGLVNQIIDIKWIGLKINEFLVVEYYSRQT"

gene complement(48799..48871)

/gene="trnT-UGU"

tRNA complement(48799..48871)

/gene="trnT-UGU"

/product="tRNA-Thr"

/note="anticodon:UGU"

gene 49420..49454

/gene="trnL-UAA"

tRNA 49420..49454

/gene="trnL-UAA"

/product="tRNA-Leu"

/note="anticodon:UAA"

gene 49934..49983

/gene="trnL-UAA"

tRNA 49934..49983

/gene="trnL-UAA"

/product="tRNA-Leu"

/note="anticodon:UAA"

gene 50331..50403

/gene="trnF-GAA"

tRNA 50331..50403

/gene="trnF-GAA"

/product="tRNA-Phe"

/note="anticodon:GAA"

gene complement(50935..51411)

/gene="ndhJ"

CDS complement(50935..51411)

/gene="ndhJ"

/codon_start=1

/transl_table=11

/product="NADH-plastoquinone oxidoreductase subunit J"

/translation="MQGRSSAWLVKHELVHRSLGFDYQGRETLQIKPEDWYSIAVISY

VYGYNYLRFQCAYDVAPGGFLASVYHLTRIQYGVDQPEEVCIKVFVPRRNPRIPSVFW

IWKSADFQERESYDMLGISYENHPRLKRILMPESWVGWPLRKDYIAPNFYEIQDAH"

gene 51511..52368

/gene="ndhK"

CDS complement(51511..52368)

/codon_start=1

/transl_table=11

/product="NADH-plastoquinone oxidoreductase subunit K"

/translation="MGNEFRCIGCICVYRSFNFRAYPNCWFSLCMAKRSIGMVLAPEY

SDNQNQKEGKDYIETVMNSIEFPLLDRTAQNSVISTTSNDLSNWSRLSSLWPLLYGTS

CCFIEFASLIGSRFDFDRYGLVPRSSPRQADLILTAGTVTMKMAPSLVRLYEQMPEPK

YVIAMGACTITGGMFSTDSYSTVRGVDKLIPVDVYLPGCPPKPEAVIDAITKLRKKVS

REIYEDRIGSQQENRYFTTNHKFHVGHSTHTGNYDQGLLYQSPSTSEIPSETFFKYKS

SVSSPKLMN"

gene complement(52248..52610)

/gene="ndhC"

CDS complement(52248..52610)

/gene="ndhC"

/codon_start=1

/transl_table=11

/product="NADH-plastoquinone oxidoreductase subunit 3"

/translation="MFLLHEYDIFWAFLIISSVIPILAFLISGVLAPISEGPEKLSSY

ESGIEPMGDAWLQFRIRYYMFALVFVVFDVETVFLYPWAMSFDVLGVSVFIEALIFVL

IPIVGSVYAWRKGALEWS"

gene complement(52256..52257)

CDS complement(52261..52275)

/gene="ndhC"

/pseudo

/codon_start=1

/transl_table=11

/product="noproductstringinfile"

gene complement(54463..54499)

/gene="trnV-UAC"

tRNA complement(54463..54499)

/gene="trnV-UAC"

/product="tRNA-Val"

/note="anticodon:UAC"

gene complement(55087..55125)

/gene="trnV-UAC"

tRNA complement(55087..55125)

/gene="trnV-UAC"

/product="tRNA-Val"

/note="anticodon:UAC"

gene 55305..55376

/gene="trnM-CAU"

tRNA 55305..55376

/gene="trnM-CAU"

/product="tRNA-Met"

/note="anticodon:CAU; tRNA-Met2"

gene complement(55605..56009)

/gene="atpE"

CDS complement(55605..56009)

/gene="atpE"

/codon_start=1

/transl_table=11

/product="ATP synthase CF1 epsilon subunit"

/translation="MTLNLCVLTPNRIIWDSEVKEIILSTNSGQIGVLPNHAPIATAV

DIGILRIRLNDQWLTMAVMGGFARIGNNEITILVNDAEKGSDIDPQEAQRTLEIAEAN

LSRAEGKRQAIEANLALRRARTRVEAINVISY"

gene complement(56006..57502)

/gene="atpB"

CDS complement(56006..57502)

/gene="atpB"

/codon_start=1

/transl_table=11

/product="ATP synthase CF1 beta subunit"

/translation="MRINPTNSGPGVSTLEEKNLGRIAQIIGPVLDVAFPPGKMPNIY

NALVVKGRDTVGQQINVTCEVQQLLGNNRVRAVAMSATDGLMRGMEVIDTGAPLSVPV

GGATLGRIFNVLGEPVDNLGPVDTRTTSPIHRSAPAFIQLDTKLSIFETGIKVVDLLA

PYRRGGKIGLFGGAGVGKTVLIMELINNIAKAHGGVSVFGGVGERTREGNDLYMEMKE

SGVINEQNIAESKVALVHGQMNEPPGARMRVGLTALTMAEYFRDVNEQDVLLFIDNIF

RFVQAGSEVSALLGRMPSAVGYQPTLSTEMGSLQERITSTKEGSITSIQAVYVPADDL

TDPAPATTFAHLDATTVLSRGLAAKGIYPAVDPLDSTSTMLQPRIVGEEHYETAQRVK

QTSQRYKELQDIIAILGLDELSEEDRLTVARARKIERFLSQPFFVAEVFTGSPGKYVG

LTETIRGFQLILSGELDGLPEQAFYLVGNIDEATAKAMNLEVESKLKK"

gene 58257..59684

/gene="rbcL"

CDS 58257..59684

/gene="rbcL"

/codon_start=1

/transl_table=11

/product="ribulose-15-bisphosphate carboxylase/oxygenase

large subunit"

/translation="MSPKTETKASVGFKAGVKDYKLTYYTPDYETKDTDILAAFRVTP

QPGVPPEEAGAAVAAESSTGTWTTVWTDGLTSLDRYKGRCYHIEPVPGEETQFIAYVA

YPLDLFEEGSVTNMFTSIVGNVFGFKALRALRLEDLRIPPAYSKTFQGPPHGIQVERD

KLNKYGRPLLGCTIKPKLGLSAKNYGRAVYECLRGGLDFTKDDENVNSQPFMRWRDRF

VFCAEAIYKAQAETGEIKGHYLNATAGTCEEMIKRAVFARELGVPIVMHDYLTGGFTA

NTTLAHYCRDNGLLLHIHRAMHAVIDRQKNHGMHFRVLAKALRMSGGDHVHAGTVVGK

LEGERDITLGFVDLLRDDFIEKDRSRGIYFTQDWVSMPGVLPVASGGIHVWHMPALTE

IFGDDSVLQFGGGTLGHPWGNAPGAVANRVAVEACVQARNEGRDLAREGNEIIREAAK

WSPELAAACEVWKEIKFEFAAMDTL"

gene 60368..61879

/gene="accD"

CDS 60368..61879

/gene="accD"

/codon_start=1

/transl_table=11

/product="Acetyl-CoA carboxylase carboxyltransferase beta

subunit"

/translation="MGKWWFNSMLSNEELEHRCGLGKSMDSLGRPVGNTSGSEDPILN

DTNKNNHNHGWRESNSCSNVDHFFGVRDIWSFISDDTFLVRDSNGNSYSVYFDIENRV

FEIDNDSSFLSELETAFSSYLNSGSKSDNRYYDHYMYDTTYSWNNHINSCIDSYLRSE

ISINKYISSGSDNPIYSYIYSYICSGESVSDSDRGSSSIRTGGNGSDFNIRGRSNDFD

GNKKYRHLWVQCENCYGLNYKKFFRSKMNICEQCGYHLKMGSSDRIELSVDSGTWDPM

DEDMVSIDPIEFHSEEEPYRDRINSYQRKTGLTEAVQTGIGQLNGIPIAIGVMDFQFM

GGSMGSVVGEKITRLIEYAANRSLPVIMVCASGGARMQEGSLSLMQMAKISSALYDYQ

FNKKLFYVSILTSPTTGGVTASFGMLGDIIIAEPNAYIAFAGKRVIEQTLNKTVPDGS

QAAEYLFHKGLFDPIVPRNPLKGVLSELFQLHGFFPLNQNSSRALGSVICSEL"

gene 62465..62575

/gene="psaI"

CDS 62465..62575

/gene="psaI"

/codon_start=1

/transl_table=11

/product="photosystem I subunit VIII"

/translation="MTDFNLPSIFVPLVGLVFPAIAMASLSLHVQKNKIV"

gene 63013..63567

/gene="ycf4"

CDS 63013..63567

/gene="ycf4"

/codon_start=1

/transl_table=11

/product="photosystem I assembly protein ycf4"

/translation="MNYRSERIWIELITGSRKTSNFCWACILFLGSLGFLLVGTSSYL

GRNLISLFPSQQIIFFPQGIVMSFYGIAGLFISSYLWCTISWNVGSGYDRFDRKEGIV

CIFRWGFPGINRRIFLRFLMREIQSIRMEVKEGLYPRRVLYMEIRGQGAIPLTRTDEN

LTPREIEQKAAELAYFLRVPIEVF"

gene 64478..65167

/gene="cemA"

CDS 64478..65167

/gene="cemA"

/codon_start=1

/transl_table=11

/product="chloroplast envelope membrane protein"

/translation="MSKKKALTPLPYLASIVFLPWWISLSFNKSLEPWVTNWWNTGQS

ETFLNDIQEKNVLERFVELEQLFLLDEMIKEYPETQIQKLRIGIHKETMQLVKMHNED

HIHIILDFSTNIICFAILSGYSILGNEELVILNSWVQEFLYNLSDTIKAFSILLLTDL

CIGFHSPRGWELMIGSVYKDFGFAHNDQIISGLVSTFPVILDTILKYWIFHYLNRVSP

SLVVIYHSMNE"

gene 65390..66352

/gene="petA"

CDS 65390..66352

/gene="petA"

/codon_start=1

/transl_table=11

/product="cytochrome f"

/translation="MQNRNTFSWVKKEMTRFISVLIMIYVITRTSISNAYPIFAQQGY

ENPREATGRIVCANCHLANKPVDIEVPQAVLPDTVFEAVVRIPYDMQMKQVLANGKKG

ALNVGAVLILPEGFELAPPDRISPELKEKMGNLSFQSYRPTKRNILVVGPVPGQKYSE

IVFPILSPDPSTKKDVHFLKYPIYVGGNRGRGQIYPDGSKSNNTVYNATAAGIVSRIV

RKEKGGYEISIADASDGHQVVDIIPPGPELLVSEGESIKLDQPLTSNPNVGGFGQGDA

EIVLQDPLRVQGLLFFLASVILAQIFLVLKKKQFEKVQLSEMNF"

gene complement(67516..67638)

/gene="psbJ"

CDS complement(67516..67638)

/gene="psbJ"

/codon_start=1

/transl_table=11

/product="photosystem II protein J"

/translation="MADTTGRIPLWLIGTVTGIPVIGSIGIFFYGSYSGLGSSL"

gene complement(67765..67881)

/gene="psbL"

CDS complement(67765..67881)

/gene="psbL"

/exception="RNA editing"

/codon_start=1

/transl_except=(pos:complement(67879..67881),aa:Met)

/transl_table=11

/product="photosystem II protein L"

/translation="MTQSNPNEQNVELNRTSLYWGLLLIFVLAVLFSNYFFN"

gene complement(67904..68023)

/gene="psbF"

CDS complement(67904..68023)

/gene="psbF"

/codon_start=1

/transl_table=11

/product="photosystem II cytochrome b559 beta subunit"

/translation="MTIDRTYPIFTVRWLAVHGLAVPTVSFLGSISAMQFIQR"

gene complement(68033..68284)

/gene="psbE"

CDS complement(68033..68284)

/gene="psbE"

/codon_start=1

/transl_table=11

/product="photosystem II cytochrome b559 alpha subunit"

/translation="MSGSTGERSFADIITSIRYWVIHSITIPSLFIAGWLFVSTGLAY

DVFGSPRPNEYFTESRQGIPLITGRFDPLAQLDEFSRSF"

gene 69552..69647

/gene="petL"

CDS 69552..69647

/gene="petL"

/codon_start=1

/transl_table=11

/product="cytochrome b6/f complex subunit VI"

/translation="MTTITSYFGFLLAASTITPALLISLSKIRLI"

gene 69822..69935

/gene="petG"

CDS 69822..69935

/gene="petG"

/codon_start=1

/transl_table=11

/product="cytochrome b6/f complex subunit V"

/translation="MIEVFLFGIVLGLIPITLAGLFVTAYLQYRRGDQLDL"

gene complement(70055..70128)

/gene="trnW-CCA"

tRNA complement(70055..70128)

/gene="trnW-CCA"

/product="tRNA-Trp"

/note="anticodon:CCA"

gene complement(70282..70355)

/gene="trnP-UGG"

tRNA complement(70282..70355)

/gene="trnP-UGG"

/product="tRNA-Pro"

/note="anticodon:UGG"

gene complement(70284..70354)

/gene="trnP-GGG"

tRNA complement(70284..70354)

/gene="trnP-GGG"

/product="tRNA-Pro"

/note="anticodon:GGG"

gene 70731..70865

/gene="psaJ"

CDS 70731..70865

/gene="psaJ"

/codon_start=1

/transl_table=11

/product="photosystem I subunit IX"

/translation="MRDIKTYLSTAPVLTTLWFGSLAGLLIEINRLFPDALTFPFFSF

"

gene 71292..71492

/gene="rpl33"

CDS 71292..71492

/gene="rpl33"

/codon_start=1

/transl_table=11

/product="ribosomal protein L33"

/translation="MAKGKDVRVVVILECTSCVRNGLNKESRGISRYITQKNRHNTPS

QLDLKKFCPYCYKHTIHGEIKK"

gene 71633..71938

/gene="rps18"

CDS 71633..71938

/gene="rps18"

/codon_start=1

/transl_table=11

/product="ribosomal protein S18"

/translation="MDKSKRPFHKSKRSFHRRLPPIGSGDRIDYRNMSLINQFISEQG

KILSRRVNRLTLKQQRLITIAIKQARILSSLPFLNNEKQFERTGSIPRTTGPRTRNK"

gene complement(72196..72549)

/gene="rpl20"

CDS complement(72196..72549)

/gene="rpl20"

/codon_start=1

/transl_table=11

/product="ribosomal protein L20"

/translation="MTRVRRRYIARRRRTKIRLFAATFRGAHSRLTRTTTQQKMRALV

STHRDRGRRKRSFRRLWITRINAVTRENRGSHSYSRLILDLYKRQLLLNRKIPAQIAI

SNRNCLDTISNAIIK"

CDS join(complement(73307..73420),144824..145035,

145534..145564)

/trans_splicing

/codon_start=1

/transl_table=11

/product="ribosomal protein S12"

/translation="MPTIKQLIRNTRQPIRNVTKSPALRGCPQRRGTCTRVYTITPKK

PNSALRKVARVRLTSGFEITAYIPGIGHNSQEHSVVLVRGGRVKDLPGVRYHIVRGTL

DAVGVKDNMGSKSQNK"

misc_feature join(complement(73307..73420),144824..145035,

145534..145564)

/note="trans-splicing"

gene complement(73307..145560)

/gene="rps12"

gene complement(73557..75592)

/gene="clpP"

CDS complement(join(73557..73802,74458..74748,75524..75592))

/gene="clpP"

/codon_start=1

/transl_table=11

/product="clp protease proteolytic subunit"

/translation="MPIGVPKVPFRSPGEEDAVWVDVNRLHRERLLFLGQEVDSEISN

QLVGLMVYLSIEDDTRDLYLFINSPGGWVIPGIAIYDTMQFVPPDVHTICMGLAASMG

SFILVGGEITKRLAFPHARVMIHQPASSFYEAPTGEFILEAEELLKLRETLTRVYVQR

TGNPLWVVSEDMERDVFMSATEAQDYGIVDLVAIENTGDFA"

exon complement(73557..73802)

/gene="clpP"

/number=3

exon complement(74458..74748)

/gene="clpP"

/number=2

exon complement(75524..75592)

/gene="clpP"

/number=1

gene 76021..77547

/gene="psbB"

CDS 76021..77547

/gene="psbB"

/codon_start=1

/transl_table=11

/product="photosystem II CP47 chlorophyll apoprotein"

/translation="MGLPWYRVHTVVLNDPGRLLSVHIMHTALVSGWAGSMALYELAV

FDPSDPVLDPMWRQGMFVIPFMTRLGINNSWGGWSITGGTITNPGIWSYEGVAGAHIV

FSGLCFLAAIWHWVYWDLEIFCDERTGKPSLDLPKIFGIHLFLSGVACFGFGAFHVTG

LYGPGIWVSDPYGLTGKVQSVNPAWGAEGFDPFVPGGIASHHIAAGTLGILAGLFHLS

VRPPQRLYKGLRMGNIETVLSSSIAAVFFAAFVVAGTMWYGSATTPIELFGPTRYQWD

QGYFQQEIYRRVGASLAENLSLSEAWSKIPEKLAFYDYIGNNPAKGGLFRAGSMDNGD

GIAVGWLGHPIFRDKEGHELFVRRMPTFFETFPVVLVDGDGIVRADVPFRRAESKYSV

EQVGVTVEFYGGELNGVSYSDPATVKKYARRAQLGEIFELDRATLKSDGVFRSSPRGW

FTFGHATFALLFFFGHIWHGARTLFRDVFAGIDPDLDAQVEFGAFQKLGDPTTRRQVV

"

gene 77725..77832

/gene="psbT"

CDS 77725..77832

/gene="psbT"

/codon_start=1

/transl_table=11

/product="photosystem II protein T"

/translation="MEALVYTFLLVSTLGIIFFAIFFRDPPKVPTKKTK"

gene complement(77891..78022)

/gene="psbN"

CDS complement(77891..78022)

/gene="psbN"

/codon_start=1

/transl_table=11

/product="photosystem II protein N"

/translation="METATLVAISISGSLVSFTGYALYTAFGQPSQQLRDPFEEHGD"

gene 78130..78351

/gene="psbH"

CDS 78130..78351

/gene="psbH"

/codon_start=1

/transl_table=11

/product="photosystemIIphosphoprotein"

/translation="MATQTVEGSARSGPRRTITGDLLKPLNSEYGKVAPGWGTTPFMG

VAMALFAIFLSIILEIYNSSVLLDGISMN"

gene 79257..79904

/gene="petB"

CDS 79257..79904

/gene="petB"

/codon_start=1

/transl_table=11

/product="cytochrome b6"

/translation="MSKVYDWFEERLEIQAIADDITSKYVPPHVNIFYCLGGITLTCF

LVQVATGFAMTFYYRPTVTEAFASVQYIMTEANFGWLIRSVHRWSASMMVLMMILHVF

RVYLTGGFKKPRELTWVTGVVLAVLTASFGVTGYSLPRDQIGYWAVKIVTGVPEAIPV

IGSPLVELLRGSASVGQSTLTRFYSLHTFVLPLLTAVFMLMHFPMIRKQGISGPL"

gene 80102..81300

/gene="petD"

CDS join(80102..80108,80819..81300)

/gene="petD"

/codon_start=1

/transl_table=11

/product="cytochromeb 6/f complex subunit IV"

/translation="MGVPITKKPDLNDPVLRAKLAKGMGHNYYGEPAWPNDLLYIFPV

VILGTIACNVGLAVLEPSMIGEPADPFATPLEILPEWYFFPVFQILRTVPNKLLGVLL

MVLVPTGLLTVPFLENVNKFQNPFRRPVATTVFLIGTAAALWLGIGATLPIDKSLTLG

LF"

exon 80102..80108

/gene="petD"

/number=1

exon 80819..81300

/gene="petD"

/number=2

gene complement(81488..82507)

/gene="rpoA"

CDS complement(81488..82507)

/gene="rpoA"

/codon_start=1

/transl_table=11

/product="RNA polymerase alpha subunit"

/translation="MVREEVAVSTRTLQWKCVESRTDSKRLYYGRFVLSPLMKGQADT

IGIAMRKALLGEIEGTCITRAKSEKVSHEYSTIVGIEESVHEILMNLKEIVLRSNLYG

TRDASICVRGPKYVTAQDIISPPSVELVDTTQHIANLTEPIHLCIEFKIERDRGYRMK

SPNNYQDGSYPIDAVSMPVRNANHSIHSYGSENEKQEILFLEIWTNGSLTPKEALREA

SRTLIDLFIPFLHAEEEDIHFYLEDNQNRFTVSFFTFHDRLANIRKNKKGIALKCIFI

DQSELPSRTYNCLKRSNIHTLLDLLNNSQEDLMRIEHLRIEDVKQILDILQKHFAIYL

PKNKF"

gene complement(82576..82968)

/gene="rps11"

CDS complement(82576..82968)

/gene="rps11"

/codon_start=1

/transl_table=11

/product="ribosomal protein S11"

/translation="MTKAIPRIGSRKNGRRIQKGVIHVQASFNNTIVTVTDVIGRVVS

WSSAGTCGFRGTRRGTPFAAQTAAGNAIRKAVDQGLQRAEVMIKGPGLGRDAALRAIR

RSGILLSFVRDVTPMPHNGCRPPKKRRV"

gene complement(83081..83194)

/gene="rpl36"

CDS complement(83081..83194)

/gene="rpl36"

/codon_start=1

/transl_table=11

/product="ribosomal protein L36"

/translation="MKIRASIRKICEKCRLIRRRGRIIVICSNPKHKQRQG"

gene complement(83310..83543)

/gene="infA"

CDS complement(83310..83543)

/gene="infA"

/codon_start=1

/transl_table=11

/product="translational initiation factor 1"

/translation="MKEQKLIHEGLITESLPNGMFRVRLDNEDLILGYVSGRIRRSFI

RILPGDRVKIEVSRYDSTRGRIIYRLRNKESND"

gene complement(83660..84058)

/gene="rps8"

CDS complement(83660..84058)

/gene="rps8"

/codon_start=1

/transl_table=11

/product="ribosomal protein S8"

/translation="MGRDTIADIITSIRNANMDKKGTVRVASTNIAENIVKILLQEGF

IENVRKHRENNKYFLVSTLRHRRNRKGTYRNILKRISRPGLRIYSNHQRIPRILGGMG

VVILSTSRGIMTDREARLERIGGEILYYIW"

gene complement(84238..84606)

/gene="rpl14"

CDS complement(84238..84606)

/gene="rpl14"

/codon_start=1

/transl_table=11

/product="ribosomal protein L14"

/translation="MIQPQTHLNVADNSGARELMCIRIIGASNQRYAHIGDVIVAVIK

EAVPNMPLERSEVIRAVIVRTCKELKRDNGMIIRYDDNAAVVIDQEGNPKGTRVFGAI

ARELRQLNFTKIVSLAPEVL"

gene complement(84736..85140)

/gene="rpl16"

CDS complement(84736..85140)

/gene="rpl16"

/codon_start=1

/transl_table=11

/product="ribosomal protein L16"

/translation="MLSPKRTRFRKQHRGRMKGISYRGNHICFGRYALQALEPAWITS

RQIEAGRRAMTRYARRGGKIWVRIFPDKPVTVRPTETRMGSGKGSPEYWVSVVKPGRI

LYEMGGVSETVARAAISIAACKMPIRTQFIIA"

gene complement(86264..86926)

/gene="rps3"

CDS complement(86264..86926)

/gene="rps3"

/codon_start=1

/transl_table=11

/product="ribosomal protein S3"

/translation="MGQKINPLGFRLGENQSHRSLWFAQPKSYYIGLQEDEKIRDWIK

IYVQKNIRVSSSFEGIGIAHIEIQKKMDLIQVIIYIGFPNLLIEGQTRGIEELQINVQ

KGLHSVNRRLNIAITRVAKPYGQPNILAEYIALQLKNRVSFRKAMKKAIELTEQADTK

GIQVEIAGRIDGKEIARVEWIREGRVPLQTIRAKIDHCSYTVRTAYGALGIKIWIFVD

EQ"

gene complement(86817..87380)

/gene="rpl22"

CDS complement(86817..87380)

/gene="rpl22"

/codon_start=1

/transl_table=11

/product="ribosomal protein L22"

/translation="MKRSSSTQVQALAQRICMSAHKARRVIDQIRGHSYEKTLMLLEL

MPYRAFYPIFKLVYSAAANASHNKSFNEADSVISKAEVNGGTIVKKLKPRARGRSYPI

ERPACHIIIVLKDSSKKKTDQDIFLETKNVWRDPIIERYIEKEREREKKDGSKNKSTW

FPPWRKPKSSFPLVRTTKKLLHRSPGR"

gene complement(87482..87760)

/gene="rps19"

CDS complement(87482..87760)

/gene="rps19"

/codon_start=1

/transl_table=11

/product="ribosomal protein S19"

/translation="MTRSLKKNPFVANHLLEKIKKLNMREEKEIIVTWSRASTIIPTM

IGNTIAIHNGKAHLPIYITDRMVGQKLGEFAPTLTFQGHARNDTRSLR"

gene complement(87835..89329)

/gene="rpl2"

CDS complement(join(87835..88266,88940..89329))

/gene="rpl2"

/exception="RNA editing"

/codon_start=1

/transl_except=(pos:complement(89327..89329),aa:Met)

/transl_table=11

/product="ribosomal protein L2"

/translation="MIHLYKTSTPSIRKGSIDSQAKSNPRNNLIYGQHRCGKGRNSRG

IITAGHRGGGHKRLYRKIDFRRNEKDISARIVTIEYDPNRNAYICLIHYGDGEKRYIL

HPRGAIIGDTIVSGTEVPISMGNALPLSTHMPLGTAVHNIEITLGKGGQLTRAAGAVA

KLIAKEGKSVTLRFPSGEVRLISKNCSATVGQVGNVGANQKSLGRAGSKCWLGRRPVV

RGVVMNPVDHPHGGGEGRAPIGRKKPTTPWGYPALGRKSRKRNKYSNSFIIRRRK"

exon complement(87835..88266)

/gene="rpl2"

/number=2

exon complement(88940..89329)

/gene="rpl2"

/number=1

gene complement(89361..89588)

/gene="rpl23"

CDS complement(89361..89588)

/gene="rpl23"

/codon_start=1

/transl_table=11

/product="similar to ribosomal protein L23"

/translation="MGKKVESGSTRTEIKHWVELFFGVKVIAINSHQLPGKGRRTGPI

MGHTMHYRRMIITLQPGYSILPLIEKRKEFK"

gene complement(89738..89811)

/gene="trnI-CAU"

tRNA complement(89738..89811)

/gene="trnI-CAU"

/product="tRNA-Ile"

/note="anticodon:CAU"

gene 89895..96866

/gene="ycf2"

CDS 89895..96866

/gene="ycf2"

/codon_start=1

/transl_table=11

/product="hypothetical chloroplast RF2"

/translation="MRGHQFKSWIFELREIKNSHDFLDSWIQPDSVKSFTSFFFHQER

FMKLFDSRIWSVLISRDSQGSIRRHCMIKGVVLLVLVVAVLIYNRNRVERKNIYLMGL

LPKPLRSIGPPNYTLKESFWSSNLNRLIVSLLYLPKGKNIYESCFMDPKESTWVLPIT

KKCIMSESNWGSQRWRNAIVKKRNSSCKISNEIAAGIEISFKEKDIKYLEFFFVSYTN

DPIRKDHDWKLFDRLSPSKKRNIINLNSGQLFEILVKHLICYLMSAFREKRPIDEGGF

FKQQGAEATIQSNEIEHVSHLLSRNKGGIFLKNCAQFHMWQFRQDLFVIGGKNRHKSD

FLRNVSRENLIWLDNAWLVNRNRVFSKVRNVSSNIQYDSIRSIFFQVTDSSQSKGFSD

QSIDPFNSISNEGSEYHTLINQTEIQQLKKRSILLDTSFLQTERTEIKSDRFSKYLSG

YSSMARLFPEREKQMNNHLLPEEIEEFLGNPTRSIRSFFSDRWSELHLGLNPTERSTI

DQKLLKKQQGVSFVPSRRSENKEIVDIFKIITYLQNTSSVHSIAADPGWDMVPKDEPD

MDSSNKISFLNENAFFDLFHLFHDRNKGGYRLHHEFELEETFQEMADLFTLSITEPGL

AYHNKEFGLSIDSYGKLLNEVFNSGDESKKKSLLVLPSIFYDLFLLVLSSIFYDLFLL

VLLSIFYDLFLLVLLSIFYEENESFYRKIKKKSVRISCGNDLEDPKPKIAVFAHNNIM

EAIHQYRLIRNQIQIQYSTYGYIRNVLNRFFLMNRPDCNFAYGIQKHPIGIQKHPIGN

DILNHLTIIIDKINQHLSNLKKIKKKWFDPLISRTERSTNLDPNVYRYKCSNGSKNFQ

EHLEHFVSEQKHRFQVMFDRLRINQYSIDWSEVIDKQDLSKSLRFFLSKSLLLLSKSL

LFLSKSLPFFVVSLGNISIHRAEIHIYELKGLNDQPGNQLLESIGVQIVYLNKLKPFL

LYDHDTSQRSKFLINTGTILPFLFNKIQKCMIDSFRTRKNRKKSFENTDSYFSMISHD

RNNWLNPQKSSLISSFYRANRLQFLNHPHCFWFYCNKGFHFYGEKTRIHNYDFTYAQF

PNILCIRNKKFSLCFGKKKHVLGERETISPIESQVSGIFIPNNVSQSGNKTYNLYKSF

HFSIGSDPSVPIYSIADISGTPVIEEQIVNFERTYCQLLSDMNLSDSEGKNLHHYLRF

NSNMGLIHTPCFEKYVPSGKRKELSLCLKKNVEKGEVGRTLQRDSAFSNLSKWNLFQT

YMPWFLTWTGCKYLYFTLKNNIYLILNIPFQYSLSGSQNFVSVFHDMMHGSDISWPIP

QKKWWSILPQRNLISESSSKCLQNLLLSEEMIHRNNESPIPLIWTHLRSPNAWEFLYS

ILFLLLVAGYLVRTHLLFVFRASSELQTELEKIKSLMIPSYMIELRKLLDRYPTSELN

SFWLKNLFLVALEQLGDSLEEIRDSASGGNMLLGGGPAYGVKSIRSKKKYLNINLIDL

ISIIPNPINRITFSRNTRHLSRTSKEIYSLIRKRKNVNGDWIDDKIESWVANSDSIDD

EEREFLVQFSTLTTEKRIDQILLSLTHSDRLSKNDSGYQMIEQPGSIYLRYLVDIHKK

YLMNYEFNRSCLAERRIFLAHYQTITYSQTSCGANSSHFPSHGKPFSLRLALSPSRGI

LVIGSIGTGRSYLVKYLATNSYVPFITVFPNKFLDDKPKGYLIDDIDIDDSDDIDIDD

SDDIDDDLDTELLTMTNVLTMYMTPKIDRFDTTLQLELAKAMSPCIIWIPNIHDLYVN

ESNYLSLGLLVNYLSRDSERCSTRNILVIASTHIPQKVDPTLIAPNKLKKCMKIRRLL

IPQQRKHFFILSYTRGFNLEKKMFHTNSNRFGSITMGSNARDLVALTNEALSISITQK

KSIIDTNTIRSALHRQTWDLRSQVRSVQDHGILFYQIGRAVAQNVLLSNCPIDPISIY

MKKKSCKEGDSYLYKWYFELGTSMKKLTILLYLLSCSAGSVAQDLWSPPGPDEKNWIT

SYGFVENDSDLVHGLLEVEGALLGSSRTEKDCSQFDNDRVTLLLRSEPRNQLDMMQNG

SCSIVDQRFLYEKYESEFEEGEREGALDPQQIEEDLFNHIVWAPRIWRPCGNLFDCIE

RTNELGFPYWARSFRGKRIIYHKEDELQENDSEFLQSGTMQYQTRDRSSKEQGFFRIS

QFIWDPADPFFFLFKDQPFVSVFSRREFFADEEMSKGLITSQTNPPTSIYKRWFIKNT

QEKHFELLIHRQRWLRTNSSLSNGSFRSNTPSESYQYLSNLFLSNGTLLDQMTKALLR

KRWLFPDEMKHLIHVTG"

repeat_region 93706..113784

/rpt_type=inverted

gene complement(97206..97286)

/gene="trnL-CAA"

tRNA complement(97206..97286)

/gene="trnL-CAA"

/product="tRNA-Leu"

/note="anticodon:CAA"

gene complement(97847..100078)

/gene="ndhB"

CDS complement(join(97847..98602,99305..100078))

/gene="ndhB"

/codon_start=1

/transl_table=11

/product="NADH-plastoquinone oxidoreductase subunit 2"

/translation="MWHVQNENFILDSTRIFMKAFHLLLFHGSFIFPECILIFGLILL

LMIDSTSDQKDIPWLYFISSTSLVMSITALLFRWREEPMISFSGNFQTNNFNEIFQFL

ILLCSTLCIPLSVEYIECTEMAITEFLLFVLTATLGGMFLCGANDLITIFVAPESFSL

CSYLLSGYTKRDVRSNEATTKYLLMGGASSSILVHGFSWLYGSSGGEIELQEIVNGLI

NTQMYNSPGISIALISITVGIGFKLSPAPSHQWTPDVYEGSPTPVVAFLSVTSKVAAS

ASATRIFDIPFYFSSNEWHLLLEILAILSMILGNLIAITQTSMKRMLAYSSIGQIGYV

IIGIIVGDSNDGYASMITYMLFYISMNLGTFARIVSFGLRTGTDNIRDYAGLYTKDPF

LALSSALCLLSLGGLPPLAGFFGKLHLFWCGWQAGLYFLVSIGLLTSVVSIYYYLKII

KLLMTGRNQEITPHVRNYRRSPLRSNNSIELSMIVCVIASTIPGISMNPIIAIAQDTL

F"

exon complement(97847..98602)

/gene="ndhB"

/number=2

exon complement(99305..100078)

/gene="ndhB"

/number=1

gene complement(100405..100872)

/gene="rps7"

CDS complement(100405..100872)

/gene="rps7"

/codon_start=1

/transl_table=11

/product="ribosomal protein S7"

/translation="MSRRGTAEEKTAKSDPIYRNRLVNMLVNRILKHGKKSLAYQIIY

QAVKKIQQKTETNPLSVLRQAIRGVTPDIAVKARRVGGSTHQVPIEIESTQGKALAIR

WLLGASRKRPGRNMAFKLSSELVDAAKGSGDAIRKKEETHRMAEANRAFAHFR"

gene complement(100927..100956)

/gene="rps12"

/pseudogene="unknown"

gene complement(101482..101724)

/gene="rps12"

gene 103559..103630

/gene="trnV-GAC"

tRNA 103559..103630

/gene="trnV-GAC"

/product="tRNA-Val"

/note="anticodon:GAC"

gene 103859..105349

/gene="rrn16"

rRNA 103859..105349

/gene="rrn16"

/product="16S ribosomal RNA"

gene 105599..105640

/gene="trnI-GAU"

tRNA 105599..105640

/gene="trnI-GAU"

/product="tRNA-Ile"

/note="anticodon:GAU"

gene 105740..106118

/gene="ycf68"

CDS join(105740..105781,105810..106118)

/gene="ycf68"

/codon_start=1

/transl_table=11

/product="hypothetical chloroplast RF68"

/translation="MAYSSCSNRSLKPNSGEIQCRSNFLFTRGIRAVRGGPPWLLSSR

ESIHPLSVYGQLSLEHRFRFGLNGKMEHLTTHLHRPRTTRSPLSFWGDGGIVPFEPFF

FMLFPRRSGESSNQ"

exon 105740..105781

/gene="ycf68"

/number=1

exon 105810..106118

/gene="ycf68"

/number=2

gene 106580..106614

/gene="trnI-GAU"

tRNA 106580..106614

/gene="trnI-GAU"

/product="tRNA-Ile"

/note="anticodon:GAU"

gene 106679..106716

/gene="trnA-UGC"

tRNA 106679..106716

/gene="trnA-UGC"

/product="tRNA-Ala"

/note="anticodon:UGC"

gene 107702..110505

/gene="rrn23"

rRNA 107702..110505

/gene="rrn23"

/product="23S ribosomal RNA"

gene 110624..110726

/gene="rrn4.5"

rRNA 110624..110726

/gene="rrn4.5"

/product="4.5S ribosomal RNA"

gene 110947..111067

/gene="rrn5"

rRNA 110947..111067

/gene="rrn5"

/product="5S ribosomal RNA"

gene 111300..111373

/gene="trnR-ACG"

tRNA 111300..111373

/gene="trnR-ACG"

/product="tRNA-Arg"

/note="anticodon:ACG"

gene complement(111965..112036)

/gene="trnN-GUU"

tRNA complement(111965..112036)

/gene="trnN-GUU"

/product="tRNA-Asn"

/note="anticodon:GUU"

gene 112414..118035

/gene="ycf1"

CDS 112414..118035

/gene="ycf1"

/codon_start=1

/transl_table=11

/product="hypothetical chloroplast RF1"

/translation="MILKSFLLGNLLSLYMKIINSVVVVGLYYGFLTTFSIGPSYLFL

LRARIMEEGTEKEVSATTGFITGQLMMFISIYYAPLHLALGRPHTITVLVLPYLLFHF

FWNNHKHFLDYGSTTRNSMRNLSIQCVFLNNLIFQLFNHFILPSSTLVRLVNIYMFRC

NNKMLFVTSSFVGWLIGHIFFMKWVGLVLFWIRQNHSIRSNVLIRSNKYLVSELRNSM

ARIFTILLFITCVYYLGRIPSPIVTKKLKETSKTEERGESEEETDVEIEKTSETKGTK

QEQEGSTEEDPSLCSEEREDPKKLHEKKKRQEILKLEILKEKEDKDLFWFEKPLVNLL

FDYKRCNRPLRYIKKNLFQNAVRNEMSQYFFHVCPVDGKQIISFTYPPSLSIFLEMMQ

RKMSLCTTEKLSPEDLYNHWVYTNEQKRYSLSNEFINRIEVLNKGSLTMDVLEKRTRL

YNDKNNQEDKNNQEDKNNQEDKNNQEDKNNQECLPRVYDPFLNGPYRGTIKKVYSRSM

VDDSITSTEDSIGTEDSIGMVWINKIHDRLPTDYQKLEHKTDTFNGEPLSTDIGPFLT

SISELARKSTTGFSLNFKKLVLISEQRRFDSENKKKCLKFLFDVITTDQNNQTIQNKS

IGIEEIGKKIPRRSYKLINSFEEREEENEEESEESTENHGIRSRKAKRVVIYTDKADP

DQNTNTHTSTSTNSDQAEELALVRYSQQSDFRRDIVKGSIRAQRRKMVIWEMFQANAH

SLLFLDRIDKTFFFSFDISRTMNLIFRNWIDTGPKLKTSDSEEEEAKEEAKKMEDKKN

ENERIAIAETWDTFIFAQAIRGTMLVTQSILRKYIILPSLIIAKNLGRMLLFQFPEWY

EDLKEWNREMHVKCTYNGVQLSETEFPKNWLTDGIQIKILFPFCLKPWRKSKLRSHHR

DPIQKKGKTENFCFLTIWGKETELPFGSARQQPSFFEPIYNEFEKKKIKVKKKCFLVL

RVFKKKTKQFRKVSKEKTRWIIKTILFLKRKIKEFANVNPIFLFVLKKVYEPNENGKD

SIIISSNKIVPKSTSTIRIRFMDWANYSLTEKKKKDLSDRTTLIRNQIERGAKDKRKI

FLTPDINISPNDTSCGDKRSESQKHIWQISKGKSNRFIFIRKWHYFLTFLDERIYIHI

FLYTVNVSRVNVQLFLESTKKIIDKYIHKEGIDEINQKKMHFISTIKKSISNISKNKS

KISGDLYSFSQASVFYKLSQIQAINKKYHLRSLLQYREAYLILKDRIRNFFGTRRILD

SKSRHKKLPNSGMNEWKNWLRGHYQYNLSQARWSKLVPQKWRTRVNWRRTIQNKDSKK

NSYEKAQFIHYEKKNDYEVNSLTIKKAKLKKNYRYDLFSYKYINYGDRKDSYIYPSSL

QVNEDREIPYNYNTPKIEPFYVLGDICISDYLGEESIIGTGKSTDRKYLEWKIFDLFL

RKNIDIESWTDTDTGTNINKMTKTETDYYQMIDKKDLFYLTIHQEINPPNQKKKFFLM

GMNKEMLYRPILNTKSWFFSEFVPLYDAYKIKPWIIPIKLLLLIFNGNENISENKNIN

ENQKKDLRISSNQKEYLELKNRNQEEKEQLGHGNIGSDARKRQKDFEKDYTESDIQKR

EKKGQPESNKKAKQELFLKKYLLFQLRWDDLLNNRIFNNVKVYCFLLRLINAKEIAIS

SIQGGEMHLDVMLIQTNPTLPELIKKGILILEPVRLSIKWDRQFIMYQTIGISLVHNN

KCQTNGRYREKRYVDENYFNGSIVQHKKMLVNRDENHYDLLVPENILSPRRRRELRIL

ICFNSGNRNAMDRNPVFFNDNNVRNWVQFLDEDKHIDTDINKFIQFKLFLWPNYRLED

LACMNRYWFDTNNGSRFSMSRIHMYPRFGIS"

misc_feature 113785..132877

/note="SSC"

gene 118362..118628

/gene="rps15"

CDS 118362..118628

/gene="rps15"

/codon_start=1

/transl_table=11

/product="ribosomal protein S15"

/translation="MVKNSFISVIPKEEKNKGSVEFQVISFTNKIQRLTSHFELHRKD

YLSQIGLRKILGKRQRLLAYLSKKNRVRYKKLIDQLDIREPKTR"

gene 118736..119917

/gene="ndhH"

CDS 118736..119917

/gene="ndhH"

/codon_start=1

/transl_table=11

/product="NADH-plastoquinone oxidoreductase subunit 7"

/translation="MNVPATRKDLMIVNMGPHHPSMHGVLRLIVTLDGEDVIDCEPVL

GYLHRGMEKIAENRTIIQYLPYVTRWDYLATMFTEAITVNAPEQLGNIQVPKRASYIR

VIMLELSRIASHLLWLGPFMADIGSQTPFFYIFRERELLYDLFEAATGMRMMHNYFRI

GGVAADLPHGWIDKCLDFCDYSLTGIVEYQKLITQNPIFLERVEGVGIIGGEEAINWG

LSGPMLRASGIQWDLRKVDHYECYDEFDWEVQWQKEGDSLARYLVRINEMTESIKIIQ

QALEGIPGGPYENLEVRRFDRASDSEWNGFEYRFISKKPSPTFELSKQELYVRVEAPK

GELGIFLIGDNSVFPWRWKIRPPGFINLQILPQLVKRMKLADIMTILGSIDIIMGEVD

R"

gene 119919..122137

/gene="ndhA"

CDS join(119919..120482,121598..122137)

/gene="ndhA"

/codon_start=1

/transl_table=11

/product="NADH-plastoquinone oxidoreductase subunit 1"

/translation="MIIDTTEVQAINSFSRSESLKEVYDLLWLLVPIFTPVSGITIGV

LVIVWLEREISAGIQQRIGPEYAGPLGILQALADGTKLLLKEDLLPSRGDVRLFSMGP

SIAVISILLSYLVIPFGYRLVLADLSIGVFLWIAISSIAPIGLLMSGYGSNNKYSFSG

GLRAAAQSISYEIPLTPCVLSISLRVIRLSNSSSTVDIVEAQSKYGFWGWNLWRQPIG

FIVFLISSLAECERLPFDLPEAEEELVAGYQTEYSGIKSGLFYVASYLNLLVSSLFVT

VLYLGGWNFSIPYIFISEPFGINKTGGVFGMTIGILITLAKAYLFLFIPITTRWTLPR

MRMDQLLNLGWKFLLPISLGNLLLTTSSQLVSL"

exon 119919..120482

/gene="ndhA"

/number=1

exon 121598..122137

/gene="ndhA"

/number=2

gene 122216..122758

/gene="ndhI"

CDS 122216..122758

/gene="ndhI"

/codon_start=1

/transl_table=11

/product="NADH-plastoquinone oxidoreductase subunit I"

/translation="MFPMVTGFMNYGQQTIRAARYIGQSFMITLSHVNRLPVTIQYPY

EKSITSERFRGRIHFEFDKCIACEVCVRVCPIDLPVVHWRLETDIRKKRLLNYSIDFG

ICIFCGNCVEYCPTNCLSMTEEYELSTYDRHELNYNQIALGRLPMSVIGDYTIRTITN

STPIKIIRGKPLDSKTITNY"

gene 123090..123620

/gene="ndhG"

CDS 123090..123620

/gene="ndhG"

/codon_start=1

/transl_table=11

/product="NADH-plastoquinone oxidoreductase subunit 6"

/translation="MDLPGPIHDILLVFLGSGLILGGLGVVLLTNPIYSAFSLGLVLV

CISLFHIPSNSYFVAAAQLLIYVGAVNVLILFAVMFMNGSEYYKDFYLWTVGDGVTSL

VCTSILFSLITTISDTSWYGIVWTTRSNQIIEQDLTSNVQQIGIHLSTDFYLPFELIS

IILLVALIGAIAMARQ"

gene 123868..124173

/gene="ndhE"

CDS 123868..124173

/gene="ndhE"

/codon_start=1

/transl_table=11

/product="NADH-plastoquinone oxidoreductase subunit 4L"

/translation="MMTEHVLILSAYLFSIGIYGLITSRNMVRALMCLELILNAVNIN

LVTFSDLFDSRQLKGDIFSIFVIAIAAAEAAIGPAIVSSIHRNRKSTRINQSNLLNK"

gene 124441..124686

/gene="psaC"

CDS 124441..124686

/gene="psaC"

/codon_start=1

/transl_table=11

/product="photosystem I subunit VII"

/translation="MSHSVKIYDTCIGCTQCVRACPTDVLEMIPWDGCKAKQIASAPR

TEDCVGCKRCESACPTDFLSVRVYLWHETTRSMGLAY"

gene 124807..126312

/gene="ndhD"

CDS 124807..126312

/gene="ndhD"

/exception="alternative start codon"

/codon_start=1

/transl_except=(pos:124807..124809,aa:Met)

/transl_table=11

/product="NADH-plastoquinone oxidoreductase subunit 4"

/translation="MYFPWLTIIVVLPISAGSSIFFLPRRGNKNKVVRWYTICICLLE

LLLTTYAFCYHFQLDDPLIQLEEAYKWINTFDFHWRPGIDGLSIGPILLTGFITTLAT

LAARPVTRDSRLFHFLMLAMYSGQIGSFSSRDLLLFFLMWELELIPVYLLVSIWGGKK

RLYSATKFILYTAGGSIFLLMGVPGMGLYGSNEPTLNFETLANQSYPLGLEIIFYIGF

LIAYAVKSPIIPLHTWLPDTHGEAHYSTCMLLAGILLKMGAYGLVRINMELLPHAHSI

FSPWLMIVGAIQIIYAASTSFGQRNLKKRIAYSSVSHMGFTLIGIGSITDTGINGAIL

QIISHGFIGAALFFLAGTSYDRIRLVYLDEMGGIAIPMPKIFTMFSSFSMASLALPGM

SGFVAESVVFFGIITSPKYLLMPKILITFVMAIGMILTPIYSLSMSRRMFYGYKLFNV

PNSYFFDSGPRELFVSVCILLPVIGIGIYPDFVLSLSIDRIEAILSIYFHK"

gene complement(126535..127485)

/gene="ccsA"

CDS complement(126535..127485)

/gene="ccsA"

/codon_start=1

/transl_table=11

/product="CcsA"

/translation="MIFATLEHILTHISFSIISIVITIHLMTLLIHETVVLFDLSEKA

MMATFFCITGLLVTRWIYSRHLPLSDLYESLMFLSWSFSIIHMFPKRRNQKSYLSAIT

APSAIFTQGFATSGLSTEMHQSAILVPALQSQWLMMHVSMMLLSYAALLCGSLLSIAL

LVITFRKNLDIPRKSNHLLIGSFSFVNEKRSVLQNTSFLSFRNYHRYQLTQQLDQCSY

RVISLGFTFLTIGILSGAVWANEAWGSYWNWDPKETWAFITWTIFAIYLHSRTNQSFQ

GVDSAIVASIGFLIIWICYFGVNLLGIGLHSYGSFTLTTN"

gene complement(127573..127652)

/gene="trnL-UAG"

tRNA complement(127573..127652)

/gene="trnL-UAG"

/product="tRNA-Leu"

/note="anticodon:UAG"

gene complement(129044..129217)

/gene="rpl32"

CDS complement(129044..129217)

/gene="rpl32"

/codon_start=1

/transl_table=11

/product="ribosomal protein L32"

/translation="MAVPKKRTSLSKKHIRRNIWKGRGYQAAAKALSLAKSISTGHSK

SFFVRQTSNKALE"

gene 130423..132657

/gene="ndhF"

CDS 130423..132657

/gene="ndhF"

/codon_start=1

/transl_table=11

/product="NADH-plastoquinonevoxidoreductase subunit 5"

/translation="MEHTYQYAWIIPFALLPVTMSIGLGLLLVPTATKNLRRMWTFPS

VSLLSIVMVFSSDLSIQQINGSSIYQHLWSWTINTDFSLEFGYLIDPLTSIMSILITT

VGIMVLIYSDNYMSHDQGYLRFFAYMSFSNTSMLGLVTSSNLIQIHIFWELVGMCSYL

LIGFWFTRPAAANACQKAFVTNRVGDFGLLLGILGFYWITGSFEFRDLFEIFNNLIRN

NGVNSLFATLCASLLFVGAVAKSAQFPLHVWLPDAMEGPTPISALIHAATMVAAGIFL

VARFLPLFTVIPYIMNLISLIGVITVLLGATLALAQRDIKRSLAYSTMSQLGYIMLAP

GIGSYRAALFHLITHAYSKALLFLGSGSIIHSMEPIVGYSPDKSQNMVLMGGLTKYVP

ITKNTFLLGTLSLCGIPPLACFWSKDEILNDSWLYSPIFAIIACFSAGLTAFYMFRMY

LLTFDGHLHAHFQNYSGTQNSSFYSISIWGKEGTKPVNRNLFLSTMNNNEKVSFFSRK

IYKMNGNVRNLIRSCRIYFENKDTSTYPHESDNTMLLPLLILVLFTLFVGSIGIPFDQ

GVIDFDILSKWLTPSINLLHQNSNYSVDWYEFVTNAIYSVSIACFGIFIASILYGSVN

SSFQNLDLINSFVKKTGSKKILLDRIINVIYNWSYNRGYIDLFYATCLTTSIRGLAEV

THFLDRRVIDGITNGVGVASFFVGEGIKYVGGGRISSYLFVYLSYVSGFLLIYYIYYL

FFLF"

gene complement(132781..134250)

/gene="ycf1"

CDS complement(132781..134250)

/gene="ycf1"

/codon_start=1

/transl_table=11

/product="hypothetical chloroplast RF19"

/translation="MILKSFLLGNLLSLYMKIINSVVVVGLYYGFLTTFSIGPSYLFL

LRARIMEEGTEKEVSATTGFITGQLMMFISIYYAPLHLALGRPHTITVLVLPYLLFHF

FWNNHKHFLDYGSTTRNSMRNLSIQCVFLNNLIFQLFNHFILPSSTLVRLVNIYMFRC

NNKMLFVTSSFVGWLIGHIFFMKWVGLVLFWIRQNHSIRSNVLIRSNKYLVSELRNSM

ARIFTILLFITCVYYLGRIPSPIVTKKLKETSKTEERGESEEETDVEIEKTSETKGTK

QEQEGSTEEDPSLCSEEREDPKKLHEKKKRQEILKLEILKEKEDKDLFWFEKPLVNLL

FDYKRCNRPLRYIKKNLFQNAVRNEMSQYFFHVCPVDGKQIISFTYPPSLSIFLEMMQ

RKMSLCTTEKLSPEDLYNHWVYTNEQKRYSLSNEFINRIEVLNKGSLTMDVLEKRTRL

YNDKNNQGKERCLCVRPKNYPPKICIIIGFIPMNKKGTA"

repeat_region 132878..152570

/rpt_type=inverted

gene 134628..134699

/gene="trnN-GUU"

tRNA 134628..134699

/gene="trnN-GUU"

/product="tRNA-Asn"

/note="anticodon:GUU"

gene complement(135291..135364)

/gene="trnR-ACG"

tRNA complement(135291..135364)

/gene="trnR-ACG"

/product="tRNA-Arg"

/note="anticodon:ACG"

gene complement(135596..135716)

/gene="rrn5"

rRNA complement(135596..135716)

/gene="rrn5"

/product="5S ribosomal RNA"

gene 135938..136040

/gene="rrn4.5"

rRNA 135938..136040

/gene="rrn4.5"

/product="4.5S ribosomal RNA"

gene complement(136139..138895)

/gene="rrn23"

rRNA complement(136139..138895)

/gene="rrn23"

/product="23S ribosomal RNA"

gene complement(139048..139082)

/gene="trnA-UGC"

tRNA complement(139048..139082)

/gene="trnA-UGC"

/product="tRNA-Ala"

/note="anticodon:UGC"

gene complement(139881..139918)

/gene="trnA-UGC"

tRNA complement(139881..139918)

/gene="trnA-UGC"

/product="tRNA-Ala"

/note="anticodon:UGC"

gene complement(139983..140017)

/gene="trnI-GAU"

tRNA complement(139983..140017)

/gene="trnI-GAU"

/product="tRNA-Ile"

/note="anticodon:GAU"

gene complement(140479..140787)

/gene="ycf68"

CDS complement(140479..140787)

/gene="ycf68"

/exception="alternative start codon"

/codon_start=1

/transl_except=(pos:complement(140785..140787),aa:Met)

/transl_table=11

/product="hypothetical chloroplast RF68"

/translation="MGEIQCRSNFLFTRGIRAVRGGPPWLLSSRESIHPLSVYGQLSL

EHRFRFGLNGKMEHLTTHLHRPRTTRSPLSFWGDGGIVPFEPFFFMLFPRRSGESSNQ

"

gene complement(141248..142689)

/gene="rrn16"

rRNA complement(141248..142689)

/gene="rrn16"

/product="16S ribosomal RNA"

gene complement(142918..142989)

/gene="trnV-GAC"

tRNA complement(142918..142989)

/gene="trnV-GAC"

/product="tRNA-Val"

/note="anticodon:GAC"

gene 145619..146086

/gene="rps7"

CDS 145619..146086

/gene="rps7"

/codon_start=1

/transl_table=11

/product="ribosomal protein S7"

/translation="MSRRGTAEEKTAKSDPIYRNRLVNMLVNRILKHGKKSLAYQIIY

QAVKKIQQKTETNPLSVLRQAIRGVTPDIAVKARRVGGSTHQVPIEIESTQGKALAIR

WLLGASRKRPGRNMAFKLSSELVDAAKGSGDAIRKKEETHRMAEANRAFAHFR"

gene 146413..148597

/gene="ndhB"

CDS join(146413..147186,147842..148597)

/gene="ndhB"

/codon_start=1

/transl_table=11

/product="NADH-plastoquinone oxidoreductase subunit 2"

/translation="MWHVQNENFILDSTRIFMKAFHLLLFHGSFIFPECILIFGLILL

LMIDSTSDQKDIPWLYFISSTSLVMSITALLFRWREEPMISFSGNFQTNNFNEIFQFL

ILLCSTLCIPLSVEYIECTEMAITEFLLFVLTATLGGMFLCGANDLITIFVAPESFSL

CSYLLSGYTKRDVRSNEATTKYLLMGGASSSILVHGFSWLYGSSGGEIELQEIVNGLI

NTQMYNSPGISIALISITVGIGFKLSPAPSHQWTPDVYEGSPTPVVAFLSVTSKVAAS

ASATRIFDIPFYFSSNEWHLLLEILAILSMILGNLIAITQTSMKRMLAYSSIGQIGYV

IIGIIVGDSNDGYASMITYMLFYISMNLGTFARIVSFGLRTGTDNIRDYAGLYTKDPF

LALSSALCLLSLGGLPPLAGFFGKLHLFWCGWQAGLYFLVSIGLLTSVVSIYYYLKII

KLLMTGRNQEITPHVRNYRRSPLRSNNSIELSMIVCVIASTIPGISMNPIIAIAQDTL

F"

exon 146413..147186

/gene="ndhB"

/number=1

exon 147842..148597

/gene="ndhB"

/number=2

gene 149158..149238

/gene="trnL-CAA"

tRNA 149158..149238

/gene="trnL-CAA"

/product="tRNA-Leu"

/note="anticodon:CAA"

gene complement(149555..152570)

/gene="ycf2"

CDS complement(149555..152570)

/gene="ycf2"

/pseudogene="unprocessed"

/codon_start=1

/transl_table=11

/product="hypothetical chloroplast RF21"

BASE COUNT 45810 a 30038 c 29677 g 47045 t

ORIGIN

1 gtccaagttt atagggatag cgaatgctgg gcgaacgacg ggaattgaac ccgcgcatgg

61 tggattcaca atccactgcc ttgatccact tggctacatc cgcccctcct ctctcaaaag

121 gattccattt tcaccattca ttattttttg atttagtctt tattacttca ctctccttcc

181 tgctgaaata cagatattgt acataagaca aaatgttgta cgtaaaaaaa aaaaaagaaa

241 aatgctttga ttttttcaaa aaatcaaatt attttgaaga ataagaatat ataaaatgca

301 ggttggtaca gaagaaacta cgatattcga tcatgaaata accagcggtt ttcataagtt

361 gaataaaaga aatgaaaatg aaaaacgatt atgtgaataa aacactactg aaccaaatgg

421 atcaatacca aacttcttaa tagaacaaga agtttggtat tgatccttca acgactcgta

481 tacactaata ccaaagtatt atccgtttgt agatggaact tcgacagcag ctaggtctag

541 agggaagttg tgagcattac gttcatgcat aacttccata ccaaggttag cacgattgat

601 gatatcagcc caagtgttaa taacacgacc ttgactgtca actacagatt ggttgaaatt

661 gaaaccattt aggttgaaag ccatggtgct gatacctaaa gcagtaaacc agatacctac

721 tacaggccaa gctgctagga agaaatgtaa ggaacgggag ttgttaaaac tagcatattg

781 gaagatcaat cggccaaaat aaccatgagc agctacgata ttgtaagttt cttcctcttg

841 accgaatctg taacctgcat tagcagattc attttcagtg gtttccctga tcaaactaga

901 ggttaccaag gaaccatgca tagcactgaa tagggagccg ccgaatacac cagctacgcc

961 taacatgtga aatggatgca taaggatatt gtgctctgcc tggaatacaa tcatgaagtt

1021 gaaagtacca gatattccta aaggcatacc atcagaaaaa cttccttgac caatagggta

1081 gatcaagaaa acagcagtag ctgctgcaac aggagctgaa tatgcaacag caatccaagg

1141 gcgcataccc agacggaaac taagttccca ttcacgaccc atgtaacaag ctacaccaag

1201 taagaagtgt agaacaatta gctcataagg accaccattg tataaccatt catcaacgga

1261 tgctgcttcc catattgggt aaaaatgcaa acctatagct gcagaagtgg gaataatggc

1321 accggaaata atattgtttc cataaagtag agacccagaa acaggttcac gaataccatc

1381 aatatctact gggggagcag caatgaaggc aataataaat acagaagttg cggtcaataa

1441 ggtagggatc atcaaaacac cgaaccatcc aatataaaga cggttttcag tgctggttat

1501 ccagttacag aagcgacccc ataggcttgt gctttcgcgt ctctctaaaa ttgcagtcat

1561 ggtaaaatct tggtttattc aattctcagg gactcccaag cacacagatt atctataaat

1621 agaaatagac aacggaaggc ttgttattca acagtataac atgacttata tgtccgtgtc

1681 aaccaataag agagatatct atctggatag atccatccga acgatttgta aattaaatga

1741 gtagggattt atccaataac aaatattttt tcgtattttc cgtacgattg gtaatgggtt

1801 gcccgggact cgaacccgga actagtcgga tggagtagat aatttccttc ttgcaatata

1861 atagatatag agtaaaaaga ccccccaaaa aagccgtgct tgcatttttc agtgcacagg

1921 gctttaccta tgtatacatc caaaactaag ttccctaaaa ggggacctaa gaaacttgaa

1981 gactcagttg attcaaccac tactgtatga acatttcaga attcaaatga ataaaatgat

2041 tttgtgattt tatctcttca tcatttaggg atcctttcta tttacatgac ctcatgacca

2101 atcattaaat gactgactag gtcattgata cggataatat ccaaatacca aatccgttct

2161 ctatgtgacc tatgagaagg agaagaggtt gttgggaaga tcaaagaaag agcttgttct

2221 tcctccgtaa agaattcttc caagaactcc gaacctaatc ttttcaaaaa agcacgtatc

2281 gtacttttat gtttacgaga caaagttcta gcacatgaaa gtcgaagtat atactttata

2341 cgatacaaac tctgtttttt tgaggatcca ctgtgataat gagaaagatt tctgcatatc

2401 cgcccaaatc gattgagaat ctcagaatct gacaaatcgg cccgaaacgg cttactaatg

2461 ggatgccctg atacattaca aaattttgct ttagccaatg atccaatcag aggaataatt

2521 gggactacgg tctcgaattt cttaatagca gtatctattc gaaacgaatt ctctagcatt

2581 tgactcctta tcaccgaaga gtttagtcgt acacttgaaa gatagcccag aaaatagaag

2641 ggatgattat ataattgctt tatatggatc ctggccggtt gagaccacaa gtaaaaatga

2701 cattgccaaa agttgacaag gtgagatttc catttcttta tcagaagacg agcccccctt

2761 gaagccagaa tcgattttcc ttgatatctg acataatgca taaaagggtc tttgaacaac

2821 catagggttt tctgaaaatc gttacaaagc actactacaa gatattctat ttttgcatag

2881 aaatgtgttc gctcaagaaa ggatccaaaa gattttgatc gtaaatgaaa gggttgttta

2941 cggagaaaaa tgaatatgaa ttcacattca tatacatgag aattagagag gaacaagaag

3001 aatctttgat tctcttttga aaaaagggaa atggaatttt ttggagtaat gagactattt

3061 gaattccaat actcgtagag agagaatcgc aataaatgca acgaaggagt atcttgtatc

3121 caagagtgaa gggtttgaac caagatttcc agatggatgg ggtggggtat tagtatatct

3181 gacacatgat ttaaatgtga taacttgtcc tcgaaaaagg gaaatattga atgaatagat

3241 cgtaaattat gagattttgc tatttctttt tcttctaggg aagataccaa tcgcagcgag

3301 aatggaattt ccacaacgac tgcaaaaccc tccgatatca tttgagaatc aaaatgattg

3361 ttgtgcccaa cgaatcgatt ttgattagaa tcattaaccg aaataatcaa acgattctgt

3421 tgatgcattc gagtaattaa acgtttcaca attagtgaac tggatttatt gtcatgatct

3481 aaattttcca ccggttcata aagaatcgat ccatttaaag catgaccatg agcaagcgcg

3541 tagatatatt cctgaaatag aaacggatat aggaagtatt gttgccgaaa tccatccatt

3601 tctaaatatc cttgtagttc ctccatttcc atttgaaatt acacttgaac caaatggggg

3661 atttcttgag ttatcaaata atacatagta cgatacggtc agaacaaggt atatagtaag

3721 aaaagaatag ataccccgga gccagaaagg acaatcaacg gatcctattt ccatccaatt

3781 tatttatgtt cgttatagtt acaagagatg gttagaaatc ctttattttt acaacccgat

3841 cactcttttg actttggaat aatgaatttt gatcagtata ccgtttcttc tacacattcg

3901 tctccactac ataatagaga acataataga gaatagttag gattcattaa aaaaaaggaa

3961 ttgatgatcc actcacaaga gaaccctttc ccacatcagg cactaatata tttttaacgt

4021 ctaattagat cgggtaatca ttcgaattaa gaacaaacag aagctcgttg cttttggttt

4081 ccctataatt ggagctatag ggctctatcc atttattcac tcgacccaac ttgaattgat

4141 ttgacccctt tccaagaaaa gaatcaaaac aagattttgt atcgatccgt taaggatgaa

4201 gtattctaag agttctccat tgatacgaca tgctgttttt tcctttcatt ccctttcagg

4261 atcagtcgtg gtcttacaaa ctccaccaat ggtatggacg aatccgttgc ttcatcaaaa

4321 tgtgtaaaag accatagccg cacttaaaag ccgagtactc taccgttgag ttagcaaccc

4381 atataaatag ggtgtgtaga tacgatcgga ataaaaaata aataaagaga ttcgattgcc

4441 cgacctcgtc aaaacattga actagcaaca gatcaaaaag aaagatttga tgatcaattg

4501 tgaacataaa aatgaacaga gatcagatga aaatacaaca gattctggga taaattatag

4561 agaaaatcta aatagatgta aaaattgata gactacccat actctatttt ttttttttca

4621 ttatattaac taagatactt cttgtgtcac aacgaaattg acgaacccat cgtttgagtg

4681 aaataaagaa acaaacttat gaaatgtgga taaatagatc tatttatcca cgatcgaatt

4741 atatttgttc gatacaccat tgtcaatatg aattgaatgt tgagaaaacc aattcaataa

4801 ataaaacaag gacttgtgtt ggagtgacac tacaacatag ataagggatg aagtatgagt

4861 ggggaaataa ataaggaatt ccggtaggaa aaaaatgtcg ggtttattca atatttattc

4921 aatagaggta caataagcaa gattgacctt ttgtttgttg gtggagtccc aacgaaaacc

4981 atctgattga tggtaatccc ataatttccc agttatttca tctattcact caatcttttt

5041 tctatctgtc tcatatcaag agaagatatt ttttttttat agttctatga tacggggtca

5101 tgtgagagca acaatgaata gagaatagag aaaaaaaata aggaatggtg gagaaacaat

5161 tagacaaagc tagatactgg cccccccctt ttttttttat ttcattaatt tcatttgatt

5221 aattcgaaat tccttaaata cctccgcctt ctttgaaata tcatgaacag ttcctgtagg

5281 ttgagcgcct ttttcaagga aatatagaat agcggaaaca tttgaataag tttggttctt

5341 tatcggatcg taaaaaccca ctttacgaag atctcttccc tctcttcggg atcgaacatc

5401 aattgcaacg attcgataga tgactcattg ggatagacat aaatgaacaa ccccccctag

5461 aaacgtataa gaggttttct cctcgtacgg ctcgaaaaag aatgattcga atttatgtat

5521 tgtagtggca aatagatcca caaaatcatc aattagacta tgatttgagt catttttttt

5581 tgttcttcct tcctgaaaaa aaaaaaaaaa aaaaataaat ctcattcgta ctcataactc

5641 aagttgggta attctcaaag agctcgaagg gaaatcctta gacatttatt gagccgtctc

5701 taacctcttt tgtttgtctc gcctagaatc gatttgattt cttccccatt ctgatctagt

5761 tgttgagaca attgaaaacg gtgtttcctt gttccggtat cctttatttt atctttgctt

5821 tgaatccttg ggtttagaca ttacttcggt gatccttaat tgtttcaaaa tggtagcaac

5881 ataccttttg ttatttcgtt ctatggaaac gattgattcc cctgtgatac acttttgatc

5941 ggaattggta aaactatttc gacaaattca ttttactttt tttttttact tgttcgaact

6001 tgatcctttc aatttctata ccgaagatat acttacgaag ttgttccaac ttattgattg

6061 gcattaaccc tagatccttc tctctgctaa atgaaccaat tctttatgct cgagctccat

6121 catgtgctat attataatta tattatttta ttacaacccc aaaattgggg tcctagtgga

6181 atagaacaaa ctatgtcgag ccgagagcat cttctttgat atataaaatg gtgggtacaa

6241 gaatccacag ccaataatgt ccttcaagtc gcacgttgct ttctaccaca tcgtttcaaa

6301 cgaagtttta ccataacatt cctctaattt tggaccggta tggaattgat tcaatatgga

6361 atcatgaata gtcattggct caatcggtat atagtatatg aagtcctcca tactttcatt

6421 ttcatatatg gatctggaga agtctcagca agaatataat ttaaccccat attttattag

6481 aagaatgaaa cacatttata aaaaacacga agaaatgcgt tgcttaacac ttctttacat

6541 cttcaacaga ttgttaggga tgatgaatca tgaaagatcc aattctttct caaacaacta

6601 aaactaaaga agggtctaga ttaccgatac cggaacagaa tgagtcatta accaaataag

6661 ctattccaat gaccgggaaa gatcgcaagt gactcgatag gatagattca ccaatgtcac

6721 acttcttcga ggagaaagaa tttataagga atcataaaaa acaatttcaa gaatttccta

6781 cttcgacatc attactggaa ataagttttc cagtaaagac tgaattgaat ctctaaatcc

6841 atcaacaagt cggtacaacg aggatctaaa aatgtttagc agatatctga ttaattaaat

6901 cagacgcgaa tttgatcatc ataagagacc tctatgtttc ctttccgatt gaatcatcaa

6961 taatttaaac cagataaatg ggtcaagaaa caaatccaat tgttttgttt tcttggggat

7021 ggatagaagg ggctcatgaa agaaaagatt aaggtcggta ttctttcagg tattctttca

7081 tctgattgat aaaatcagaa tcgtaggagt ctgattttat cgtattcatc agatacacca

7141 aacaagtgtt accgggggta atcgtgggta tttaagggat cgcagatctt tttcgccaaa

7201 actgaaacac cggtgtcact gatcactgaa atagaacaat aacaatatat ataggcatgg

7261 ttcattttct acagattttt ccttacttca gattcaggaa tctttccata aagtaaagtg

7321 agtgtatgaa tctcccccct cccccaattg atcgctacat tgatttccaa ttattcattg

7381 gagccaaatc aaatacaaaa taaaagaaat taggtccaaa gaaaataatc cgttccgtat

7441 tgaattttct tgtttgttaa taagatccgg atgacaaggg tcttgaaatt gataccttcc

7501 tttcctttgc ggattaactc atatcgacac gaattccatg gggatcatga atcataccca

7561 aagccaattt aaaaggatct tcttatggga tgctatcctg tcttgtataa gtacaaagca

7621 aaatgggttc atatcaattc gttgttacta ttttgtttga ttttgtccca ccccagtctc

7681 aggagcttta attccagcga tggaattaat accaagaacc cccccgtttt ttaggattca

7741 ggatccacat agaattagtc attttgtcta ccctatcttt atttatattt actttaggga

7801 aagtagagac ttctctttta tttcgcattt cgactcagaa tgcatcatgt gagaatccaa

7861 atatcataga tatggggcat aaagtttatc caaatgacta actaaccttc aatatgaata

7921 tgggcgaggg ggcgaacata cgctgaatgg cccacccagt ttttttttga atttcacttt

7981 gatctttgtt cccatcctac ctatatcaaa aaagatattt atttccatcc acatgtcatt

8041 gatactcgat ccgtttgttt gtgagaaaca aaatcgaaag ggaagatatg attctataga

8101 agaatcatta gaaatcacaa agaaagattg gatcacattg tatccaacat tacaccctta

8161 aacagaagat tgttaaaaag aacaatcttt gttatgatag aattggtctg ggacggaagg

8221 attcgaacct ccgagtaacg ggaccaaaac ccgttgcctt accacttggc cacgccccat

8281 ttttatttct attcggcacc aataaacact aatatcggta ttggttgttc gtcaattcca

8341 gccccaatat ctattgaatt ggttgttgct atgattctac acatgtagat gtagaatcaa

8401 aatgaattta ttgatcatta catataattc aattaagata ttgtatgtaa ggtatgattc

8461 cttctattct catttgagaa ttgaaggatt tttgattgag ggagttcaaa gaaaaagaaa

8521 gattttgcgg cctactttcc cttctttctt cattttcccc ttatatcaat aacccaataa

8581 taatgaaatt ttctccaaga acaaaatgtt tgttatgctt aatatcttta gtttgatctg

8641 tcttaattct gcccttcatt cgagtagctt tttcttcgcc aaattgcccg aagcttatgc

8701 ttttttcaat ccaatcgtag atgttatgcc agtcatacct gtgctctttt ttctcttagc

8761 ccttgtttgg caagctgctg taagttttcg atgagatctt taatactgtc ttagaaacat

8821 tcatgattta ttcgataaaa aaaaattcta ttcttaagaa ttgataagat cagataatga

8881 accctcgact caaacatgga aattcttttg gataaccgag atgaatcgga atcacctcat

8941 ttcttcattc cttctggggg tcgaagaccc tatgtatggt ccctacaata cctaattgta

9001 ggtatgagag atcattttgt taacgaaaga atcagaatct tattacaaat gcattcctgc

9061 aaattcctta tgttttctag aaaccgcttc ttttcttggt gtcaaaacgg aatatgtggt

9121 acaaaaatgg agaatctatt cccctatttc ccccaaaatg atcttggaga ttgtgtaatg

9181 cttactctca aactcttcgt ttacacagta gtgatattct ttgtttctct cttcatcttc

9241 ggattcctat ctaatgatcc aggacgtaat cctggacgtg atgaataaaa aaatcagggg

9301 tttttccttg ctcgatttct gaattttctt aggattttct ttctccattc catacattta

9361 actatgagaa agggggttag agattttttc gaattcgaaa gggaaatatc aagtgatcag

9421 aagaaacgga gagaggggga ttcgaaccct cggtacaaat aattcgtaca acggattagc

9481 aatccgccgc tttagtccac tcagccatct ctccccattt taaatggata attcatatgt

9541 gacgcgtgaa gtaaaggttg ataaaagttt ttccttatct ttctttattc tataaatata

9601 gacgaatttg atcaatcatc aattcccttt agataatgat tcgaaacaga tatctccaat

9661 agaaagagta cctctttgat ttcgtccgaa aagttctttc ttttattccc ccggcctggc

9721 cagtacctag ccaggccatt ccttgttcca atgaatcata gatcaaatga tttatttgat

9781 ttgaaaacga aaatgcttgt tattgaagca gcaacaaggc tatttccatt cctatgatag

9841 gagtgtcatt tgttattatg tttttccttt tctcgattta cttaaatgga ataaaaaaaa

9901 catatttttt tctattataa tagaactcat atatttcttc aaagaacatg tttgaacctg

9961 aacccttgag tccacaacca aaacaatagg atttttcact cgatccaatc gacccgaatt

10021 cataagattt ggcagttgaa tgaataggaa aaggagtagc ttcgaaaaag aaaaatggag

10081 ctctggattc ttgtacaact caactcattt ttatgttccg acttcaatgg ctctttcggg

10141 ccgggactat cagtaacggc tccccgataa aagcttgtta ttgaaatgaa cctccttctc

10201 ctattttatc aagtctcccc gtcagagcac aacatgtcag caccccaatt ttcatgattc

10261 tgatcctatc ttgattacgt ttcacgccct tgttcgacaa atggcccgct cgtatacaat

10321 aattatattg tagcgggtat agtttagtgg taaaagtgtg attcgttcta ttaacaagtg

10381 aaatagataa gggatctttc gtttgattcc tattctgatc aaaaacttta tttattaaaa

10441 gggcattaat cccttacctc tcaatgccac atttgaggaa gaatatacat tctcgtgatt

10501 tgtatccaaa agtcaagtca attagaaatt gactaacaaa attggattat ggaattgcga

10561 agcataattt ttttttttga agttggatca accattccaa ttgaatgagt ataagtaagg

10621 gatccatgta tgaagataca aaagtctatt tctaatcgta actagatctt ccattttttt

10681 tttttagggg gagattgaag ccaaatagct attaaacgat gactttggtt tactagagcc

10741 atcgacatat tgtttcagct cggtggaaca aaaaaattct tttcttcagg attcttgcaa

10801 gtacaaatag ggaacgaagt aactagaaag atttgtgaga atcctcctct ttctagaggg

10861 atcatctaaa aagcaagtca tttggggtgc attcagacga aaaggctgac atagatgtta

10921 tgggccaaat tgatttcttt gaattcagat ttgctatgac tcccttttcc catacatcgt

10981 aaatttttta gtttttttta tgtcttagat ttgggaatcc cataaaggag ccgaatgaaa

11041 ccaaaatttc atgttcggtt ttgaattaga gacgttaaaa atgatgaatc gacgtctact

11101 ataaccccta gccttccaag ctaacgatgc gggttcgatt cccgctatcc gcttcatatt

11161 aattattata atacatgctt ttgatatgtc cctaaaatct ttctttcaca tacaatccta

11221 ttcctttttt ttataggaga taggaaagtc agaacgtgaa agaaatcgga atgaaaagcg

11281 tccattgtct aatggatagg acagaggtct tctaaacctt tggtataggt tcaaatccta

11341 ttggacgcaa tttatttcca tctatttttg tagattgcta tgtcaagaaa catattttga

11401 atgattcgaa tcggggccat ttctcaacga ttcgtcttgt acttaagagt gatcaatttc

11461 tttatttttg ttcctgaagt agaaacagtt ctatctgttc cggaatagct tccttcaaaa

11521 gggcttccgc ttgcgcggta aatgtcttgg tagaagatat gatttcttgg acctgaggtt

11581 tatttgtttt taagtaggta cgtaactgaa cgagaaattt ctttacctgt ccaatttcta

11641 acggatcaag atacccattc gctccagtat aaatagtaac tatctgttct tccaccgtga

11701 gaggggctga ttgggattgt ttgagcaact cgcgcaatcg ttgacctctt gccaattgat

11761 tctgagtggc tttatcgaga tcagaagcga attgtgcaaa ggcttctaac tctgcgaatt

11821 gagccagttc cagtttcgat ttgccggcta cttgtttcat ggctttaatt tgagctgcag

11881 atcctactct cgagacagaa atacccacat taatggcagg acggattccg gcattgaata

11941 gatcagcgga taagaatatt tgtccatctg taatggaaat cacattagta gggatataag

12001 ccgaaacgtc cccagattga gtctcaacta ttggtaaagc ggtcatactt ccttcaccta

12061 aacgagaact tgatttagcg gctctttcca aaaggcgtga atgcaaataa aaaacatctc

12121 ctggataagc ttcgcggccg ggcggtcttc ttaatagaag agacatttga cgataagctt

12181 gtgcttgttt ggagagatca tcataaatta ttgaagtatg tcgttcacgg tacataaaat

12241 attcagccag agccgctcct gtataaggag cgaggtattg taatgtagca ggtgaatccg

12301 ccgtttcggc taccacaata gtgtattcca ttgcccctcg ttcctggaaa gtggtcacta

12361 cctgggccac ggaggatgct ttttgaccaa tagctacata aacacatatt acattttgcc

12421 ccttttggtt gagaatagta tctgtggcta ctgctgtttt tccggtctgt ctgtccccaa

12481 taattaattc tcgctgaccg cgtcctatag ggatcatcga atcaatagca ataagtcccg

12541 tttgaagagg ctcatatacg gaacgtctcg aaataatacc tggagcagga gattcgatta

12601 accgagattc agaagctgaa atttcacctc tcccatcaat gggtttagcc agagcattta

12661 taacacgacc caaataagcc tcactaacgg gtatctgagc aattcttcct gttgctttta

12721 cagaacttcc ctcttgtatc atcaaaccgt cacccattaa tacaacgcca acattatttg

12781 attccaaatt cagagcaatg cctattgtac cctcttcaaa ttctactaat tcccctgcca

12841 ttacttcatc aagaccatga atacgagcaa tgccgtcgcc tacctgaagt actgtgccgg

12901 tattcacaat cttgacttct ctattatatt gttcaatacg ttcacggata atattactaa

12961 tttcgtcggc tcgaatggtt accattagtg tctcttaatt ctttttcgga aacaaagaaa

13021 aaaaaaaaaa aaaataatgc cttcagtaga agggctaatc agttacttct ttcatggccc

13081 cgagcatgcc aatattagca ccgatggtgc gtaaatgtaa ctcgctgttt gaacaactat

13141 tcagagttcc tagagctcct tgtaaggctt gttggaaaac tcgttgtcgc acctgattaa

13201 ttgctctttg ttgttcaaaa tgaatggttt catttttgta attttctaat cgttccaaat

13261 tctcataagt ggcattaatc aaattccatt tttctcgttc tatctcagag tatccattca

13321 ctcgaaactc atctgcttct atttccactt tccgtaagcg agcccgggct tcttcgagct

13381 gctcaatggc tccttcacgt agttcttctg aatttcgaat agtactcaag atcctctgtt

13441 ttcgattatc taataaatca cttaatgaaa gtagattatc ttcccattca tttcacaact

13501 tcatttccat gatctcttcc cgaaccaaac atgaatcttt cgattcattt ggctctcaca

13561 ctcagttact taatgggtca ttccatctat tttaatgtaa tgagcctacc ctctcttctc

13621 tgttcgtatt ccaagatatc aaaactgata cgagaccaga atattcggag gactcttccg

13681 acccgacaaa aaatctgtca ttgtcagcaa agttgtttct ttttttttgt tttcttcaaa

13741 tccaaaaaat tcttcttatt ttagacatag gtcatcgatt caacattgga taaaaaaggg

13801 cgagacacct atttttacag taaatggttc aaatcatttt atcgatatga gtgttctata

13861 tcggataaat tgccaactat tcattttttc gaaaccatct ccgtactaac gtagtggtag

13921 aaagagtacc atgttgtgcc tggacttcaa acggtttcgc tttaaccatg ttaaaggtcc

13981 cacattattg gctgatagag aatcaaagtt gatttaccaa taaattacga aatgctatgg

14041 ttcttacata tgatttctta atttattcag aagtaattcg tcgagatcgt gcacctttct

14101 ttcctattta taactttccc attcaaaaaa aaaaaaaagg aaagtgcagc cggttggatc

14161 cagcctattc ttgaaataca caactcgcac acactccctt tccaaaaaag atcaatacac

14221 caagcactac acttagattt attagatttg ttgctaaaat atcggtatta aacccgaaac

14281 tcccagcgga tggccagtga ccaaaggaaa cgaaagaatc ggttacatct ctcatatgct

14341 tttctcttat agataggacc aacaaaatgg aacagagttc tttttgtatc acttcgcccc

14401 ctttgttttg gattgatttc tttttattaa tttacttatt ataaatatga atagattcat

14461 tttaagaaat atttttcttt attattattt ccatggaaat tctcaataat ctatttattt

14521 agtcccaggt ttcatgtcaa ttacgaaata cctcgtttgt tgcaacactt cctaaaagtc

14581 aaaaagagtt tccattaaga acggaaggga agaaagcaag tgggtctgct atgctaattc

14641 ctcatcctca aatcactcca tccccggggg tattgtctca acgaagaagt gattgtagga

14701 gtgaagtttg gatataattc ggcaaggcaa gcccgcggca ataaaatagg aaaagaaaat

14761 aagtatttat ttttcacatt tataggatta aacaaaagga ttcgcaaata aaagcgctaa

14821 tgccacgacc agtccgtaaa ttgttaaagc ttccataaaa gccagactaa gcaataaagt

14881 acctcgtatt ttaccctctg cttctggctg tctcgcgata ccttctacgg cttggcccgc

14941 agcagtacct tgaccaactc caggtccaat agaagcaagt cccacagcca atccagcagc

15001 aataacggaa gcggcagaaa tcaatggatt catattaagt tcctcgcacc aaaaaaaaga

15061 aatggttaat gatacaatca accgatgaat tattacttca ttattccatc acttaagatc

15121 tatccgaaaa aaaaaaagaa ctaagaactc tgaattgaaa taataatatt actgaatcat

15181 cagagctact tcgatatctc gtttttagtt cctatccgtg gagtctttgt aaatctatac

15241 ggttccagtt cttccatttc tttgttccga accattccat tctttcaatt cttcgctctt

15301 tctcttctat atgcatgctt gacttcattt gtttattcat tcaatccaca gtcacagata

15361 aaacggaagg gcttgcattg gaatccgtct aaattcagtg ggatgggaaa taatatatat

15421 ggatagccca tatataacta gtgaatatct aatatcacat atacattgtt tctttaataa

15481 cgtaaaccat ccgtaccttc tattgaaccg gattctagaa tcattcttcg aaacatatac

15541 agggattggc ttagagccct tacatatacc cagctcgacc ccccttactt ttttttaatt

15601 ctctaatatc atacattttt tttttgctcc tattctagat cgtatatacc ggtatgagtt

15661 gggataagct tttttttaag accatttcgg aagctagtca atgatgaccc tccatggatt

15721 cacctatata agctgcggct aaagttgcaa aaataagagc ctgaatcccg cttgtgaata

15781 atccaaggaa catgacaggt ataggaacca ccgaaggtac taaagaaaca agaacaacaa

15841 ctactaattc atccgctaat atattcccga aaagtcgaaa actaagtgat aagggttttg

15901 tgaaatcttc tagaatgtta attggtaaaa gtattggagt tggttgaatg tatttaccga

15961 aataacccaa tccttttttg gtaagacccg catagaaata tgccactgac gtaggtaaag

16021 ctaaagcaac agtagtattt atatcattcg tgggtgcagc taactctcca tgcggtaact

16081 gtatgatttt ccggggtaaa agggcacctg accagttaga aacaaaaata aataggaaca

16141 tagttccaat aaagggaacc caaggaccat attcttctcc aatctgagtt ttgctcaagt

16201 ctcgaatgaa ttcgaggaca tattcgaaga aattctgacc gtcggttgga atggtttgtg

16261 gattccgaac agctatagtg gctgaaccta ataagatagc aattacaacc caagaagtga

16321 taagtacttg ggcatggact tggaaacccc ctatttgcca ataaaaatgt tggcctactt

16381 ccacatcgga tatatcgtat aacgctttta gtgagttgat ggaacagggt aaaacattca

16441 tattgccctc tgacataaat agaacttaaa aaggaattat tttgattcag ccatctcgta

16501 tctctttctc aactcgtcta ctttgaatca atcgtatatt tcggatccca agtgatcaca

16561 taatatcccc agtgattttg atctcttttt tgagactcag ggatagtaac cgattcaatc

16621 aatttatgga gtttccaaag tgattgactt atcctaatca acaatttctt atatagctag

16681 aactgccctc acaaattgcg gatactaatt tgttaagaat ccatcggatt gaagccatag

16741 cgtcatcgtt cgctggaatc ggaatatttg cgagatccgg gtcacaattt gtatcgatta

16801 aacaaattgt tggaatcctc aaagtgagac attctcgaag agccgtatat tcttcttgct

16861 gaccaacgat gattacaata tcgggtaacc ccgtcatata tttgatcccg cccagatagg

16921 tttgcaagtg agataattgc ctcttcaaca ttgctacatc tcttttcggg agacagttga

16981 gtttccccgt attttgttcc gatctcaagt tcctgaacct atgaagtctc atttctgtag

17041 tggaccaatt cgttaacata ccaccgagcc attttttatt aacataatga caccgagccc

17101 ttattgcagc tgatgctact aaattggctg ctttattttt ggtaccaact attaagaagt

17161 gttttcctat acttgctgca tcaaaaacta aatcacaggc ttctgacaaa aaacgagcag

17221 ttcgagtaag atttgtaata tgaatacctt tacgctttga agagatgtaa ggtgccattc

17281 taggattcca tttcctagta ccatggccaa aatgaactcc tgctttcacc atctcttcaa

17341 aattcatgtt ccaatatctt cttggcattt ctccccacat tttctctctt tttttttatt

17401 taagaggtac ccctgaaata aataattgtt ccgacggaac cttctaccgg agattgaccg

17461 ttaataccca gtccaagtca ttaattcctt tctattcgtt attatcttta ttaccaaatc

17521 aaatgaccag gaccctatag ttaaaagaaa agaatgaatc tgtcattaaa tcccgtaaat

17581 gatcgttctg atgtatcagg gaaattattt gggatgcaag aaccaaataa ttctctgtgg

17641 tggaacaaaa gatctctcat ttcctcctcg aatggattct tctttttgat ttccaaagga

17701 atgttgctgt gttgccttga gcggtgcact aatcctttga atccggtacc aacaggcatc

17761 atccccccca gaacaacgtt ttctttcagg cctttcaacc aatcaatacg acctcgtaga

17821 gcagcttttg ctaaaactcg agcggtttct tgaaaactcg cttcggatat gaaactttga

17881 gtattcagag atgccctcgt tattcccaat aagatggctc ggtaacagat cgcttcttcc

17941 aaagcacgcc ctgttcgttc cgctcgcaac aatccgatta gttctccagg tgaaaaaaca

18001 ttagacattc catcttctga aaccaacact tttgatgtta tttgacgtac aataatctct

18061 atatgcctat tatggatctg caccccttgg gatcgataaa ccttttggat cttattaacc

18121 aaagagatac gactttgcgc tatggttagt tcagcgccaa tcaagaatcc ccaaggaatt

18181 ccaaggattc ttgttatacg ttcgttccaa ccctcaaccc tcttttctaa gttcatcgat

18241 attgaatcaa tcgaacgcgc ttctaacact tgttccactt ttggaagacc ttgtgttata

18301 tcacccgatc tcgatttttc atatataaat gtaactaatg tatctccttc gtaaaggatt

18361 tctccacaat ggccatgaac agttgcccct ggcgtagcca aatggggctt agcggatctt

18421 atgactaagg agtcaacatg aacaattaga acttgactcg attttatgtg tggtccgcat

18481 ttggatatac atacattttc acaaataaac tgtccaaggc taattattgt ggatgtctcc

18541 tcacaataat cgtgagggag aaagcaccaa ttcaaatcga atggattaaa aatgatgtta

18601 ctgcatgaat cgggattata aattctccca ttttcatcca ttaaataata tttaagccct

18661 tggaaagtgt gtttgaaatt gtcaagtaga aaatatttct ttaacaagat ctgattatga

18721 gttattaaat agaaatagta aaatgaataa aaattcgtaa tttgaggtac aatccctaaa

18781 ggacccaacg aattcctaat gggaatcgcg ggatcctctt taattaattt aattaattct

18841 ttgtgagatt tcgaaccatt gaatagacca attcgagaac aatcggatga taacaaaatt

18901 agaaaagatg gacattcctt atttctattc agcaacgtac gaatagtccc ttgatgttgg

18961 gtaaatgatt gaatcctcgc cttgaaataa aaaggattaa tattggtgcg atctgatcca

19021 ttattggcaa tcaatcctga acttgccata tcattccttt ttccgatata aaaaatagag

19081 gacttcacta aatcaattct tatgaaatct cgaatcagat catttgccct tacttcaaca

19141 aaggaagcat gaacctcttc tatagaacca tctcggtctt ggttccaatt caatactaaa

19201 caagtccgaa ctaattgaag acttgtgtga taaattcccc gaatcggttt gccatttcca

19261 taaaggatat aattgacaat tcgtagttgc acattatccc tttcctgcaa cggatcctgg

19321 gggaaaagcg ttgctaaatt tatcccatca gctatttcat atgtgactac gggtcgaact

19381 gaaacaaaat actttttctt ggtaggtgta atccgttgga cataaatcca atttttcaat

19441 ttttttgatt ccttagaatt tttttttccc gttcctggtg gtatcaagat gccgcagtgc

19501 cgggatatct tatctgtcgc tccaggaaaa tagatatccc cagaaaagat tttgagttca

19561 atcctttttt tttttctctc cacccggacc aatccgccta cttggcttct tgtatttaaa

19621 gcgattcgtg tatctactcc aatgatactg ttgttccgta ccattatggg cgaagaaccg

19681 ggtaagatat gcacttcttc gggaatgaaa aaaaatcgat ctactttcat ttggtatttt

19741 ggcctaaatt cttttgctct tcgatactca atcaaatcct ctttttttac gattgaaccc

19801 acccctatag tcccatattt agtaattcct gaactgcttc ttctgtatag gggatcgtcg

19861 aaataagcaa gaatactatt tctaggtaaa acaccattta tgggtatttc aatcgagata

19921 ccggaacggg gcattagttc tttttctcgt tcttgatcag attggaatgg gatgataaat

19981 ctatttcttc gcctcttagc caataaatca gaattctcgt ggagaatggt aggatatatg

20041 aaattccaat gaccgttgga tatgattcga tcaggtcctg aataatcaag aacccccccc

20101 tttttaccgt aaggattcga actaaacaat ttgtgtctca cttgatcatt agtcacggag

20161 aggtcagaaa tagatctccg ttcaacagaa tgaacattca tttgatcttg atccttgtgg

20221 agcgaaaaag gcactatact ggatctgcac agagctcctg ataatatcca taaatgactt

20281 gttttgggta agagatgaac attaccatat ttatattcag gtgcatggta cacatcggta

20341 ctccagtgca tttctccctc tgagtcagaa taaatatgtt ttcgaacttt ctctttaact

20401 ttattaaaat tgaaagtgga tgttccagcg cgaatctcag caatcacttg ttctgattct

20461 acatattgat cattttgaac taaaagaaaa cttttgggtg gaatattcac attatgtaga

20521 atatcgtgac tctcaatagt tacatacagg tctatataac atagaaaagc aggatgccca

20581 tgacgtgtac gtgtgggatg aaccaaatcc tcattgaatt tgatttttcc cttaaaagga

20641 gctcgtacat gttctgcagt accacctgtg aatactccac cggtatgaaa agttcttaat

20701 gttagttgag tccccggttc gccgattgat tgacccgcaa taatacctac tgcttctcct

20761 aattcgacca ggtcgccatg agtgggactc tgaccataac ataatcgaca gatccaagat

20821 atactcctgc aaagaaaggg ggttcgaata tatattggtt gtgttcgaaa ggttatgaat

20881 cgagtgacaa gtccaacccc aatatcttta ttttgagcgg caatgcaacg tggacccata

20941 tatatattgt atgctaatac acgaccaatt agtgtttgga cccaaattct ttccgtcatc

21001 ccatttctag gactcacgga aatgcctcgg gtagtgccac aatctgttct acgtacaaca

21061 atgtgttgaa ctacttcaac aagtctacgc gtgaggtatc cagcatctga tgttcgtaca

21121 gcagtatcca caactccttt gcgggctccg tagcaggaaa tgatatattc cgttaaagaa

21181 agtccttcgc gtaaattgct ttgaatcggt aaatcaatca tttgtccttg gggatccgac

21241 attaatcctc tcatacctac taattggtgt acctgagatg catttcctct agctcccgaa

21301 aaggacatta tatggactga attagaagga tcagtcatcc taaaattagg atgcatttct

21361 tgtctcaaat attcacttgt agcataccat atctcaatgg attggcgtaa tttttctact

21421 gtgtgtacat tcccataatg atggtgcttt tctaaaatga aactttgttg ttcagcatct

21481 tggactaacc accccttaga aggtactgtt aaaagatcat caattcctaa tgaaatggat

21541 gtagcagtgg cttgctggaa acccagagtc tttacttgat ccagggtgtg cgatgtatat

21601 gccattccga agtgatctat taatctgcta ataagtcgtt tcatggcaga cccatctatc

21661 gctttattgt aaaagaacag atcagcccat tctgccataa gtacttctct attccgctga

21721 gtaggattcg ccaatgggtt tgagtcagtg attcgaagac ctcctttact ggatctcgat

21781 tcatgtagaa attaaggaat tatgattcca gttgaaccgg agagatccaa attcccgcgg

21841 tattacataa ttccttagct taggtaccgt atgagtaggc ctgacaaaac ccctgtatgg

21901 cttcttctat ttctcgagaa aaagaaatag gaccaacagt ggttcgggtg tatatacaaa

21961 gggtttgttt ttttatacgt cttactatta gatagtgccc ataaatttca tgataggtac

22021 ccaaagattc atattgaact tcgacaggaa cttctcttga ggcaatgaca cgttgatcta

22081 gtcgccaccg aagccacaaa ggactatcta aattgattcg tttctgctga taaactataa

22141 gtacatcata ggaactacaa aaatagggct ctttctcttt cgtagtatat ttatacttat

22201 aatcattaac tgtttcattt tgatagttta tgcgattcca tggattatac ctatttgcac

22261 aaatacctcg acgattcccg atcgttaata catagagtcc aataagcata tcttgggttg

22321 gtaccgaaat gggatctcca atagccggag aaaagagatt catatgagaa aacataagta

22381 aacgagcctc cgcttgcgct tccaaagata aaggtacatg aacagccatt tgatccccat

22441 caaagtctgc attgaatcct ttacgaacta atggatgtaa acaaatagca cgtccacccc

22501 ctaaaatggg ctggaacgcc tgtatgccta atctatgcag ggtgggcgct ctattcaaca

22561 atacaggatg cccctgcata acttcttgaa gtatttccca tacaatgggt tctttttccc

22621 gaatttgact tttagcaatt cctatgttag aagcgatatg tcgtctgatt aaaccacgaa

22681 tgacaaatgt ctgaaaaagt tctattgcta tttctcgagg taatccacat cgatgtaatg

22741 aaagcgaagg gcccacgaca atgacggaac gccccgaata atcgacccgt ttaccaagta

22801 gagtctcacg aaatcttcct tctttgcctt caattacatc tgaaaatgac ttgtaaactt

22861 tattatgacc gtctctcatt ggttgtccgc ggatcccatt atcaagaagt gtatccacgg

22921 cttcttgtac caatttctcc tgacacatta ctaattcccc tggcgtagat ctacttgttg

22981 ttaatgaatc ggtaagagta ttgttccgat agataactct tcggtagagt tcattaatat

23041 ccgaactcat tagtttaccc ccatctatct gaatgattgg cctcaactcg ggaggaagaa

23101 ctggtaatag gcacaaaacc atccgttctg gttctacatt tgttcgaata aaatgcttag

23161 ctaattccat gcgtctaacc aaaaaatcct ttcttcttcc aatttttcta tcttcccatt

23221 cattcccagt gaacccttct tcccctaatt ccttccattc taccgatgaa tgatctataa

23281 taattcgcaa atccggatcg gctaattgtt ctctgatagc acctgctcca gtagagattt

23341 ctcgatttcg aaatgtatcg aagcctgggg tagtaaaaaa aagtgggatg ctgtatttcc

23401 gggattggat ttcagattcg aatgaacctc gtaatcgcaa gaaagtcggt tttttagcta

23461 tgggcctggc aaaagaaaaa ttgggatagg ttcctatagg atcccccctt caaaatcgga

23521 cgtgatggtt tcctctcatc cggctcaagt agttacacca aataaagaaa ggggttctcc

23581 actttcaaat tttgttctag aaaatcccag aaagatctac tccttactca agttcccagt

23641 gaggaccaac aaaaatattt catggattca tccttccttt gacttttctg aattacttat

23701 tcaattacga taaaaatgga atgtcaaatt attgagtagt ctacttccct tcgaatgata

23761 aatcccctta aatgaaagga ataccttgga attaataagg gatttacttg tctatgtatt

23821 gtttcattcg atcttttagg tctccacttc acctcgacgg ttatgccata atgtcccttg

23881 aagcatatat gcgatggatg ggctcctgta accgtgtcat atttgtttat ttgaacagaa

23941 tctctttcta aaagaaatag aacgtctaat tccacgaaag aagttttttt tttcacgagg

24001 tacaactgtt atatgttacg gaatcgacca tggatcaatt cccctttcat ttggaagtat

24061 tgaatacaac cataattctg agcttcatat tcctcctccc aagatacatg tcagagtcag

24121 gggcatcccc atcgaattga atgggatgac agtttctcat tccgaatctg taaaatccta

24181 atttcgatca aatcacacat cgcagtatac taggccttct aattccttaa ggggtttatc

24241 taaaagattc gcgatataac taggaagacg tttcaaatac catacatgag tcgctggaca

24301 tgccagtttg atgtatccca tttgatatct tcgtatccga gaatcaacaa attcaactcc

24361 gcattgttca caaaatttcg gatcttcttt ttcctctccg atcactcgat aatttccaca

24421 agcacaaatt ccgcttttta taggtccaaa aattctttca caaaacaatc catccttttc

24481 cggtttattg cttttgtaat gaaaagtata gggttttgtc acctctccaa ccatctctcc

24541 attaggtagg attttggtgg cccaagcact tatttgttga ggagaaactg atccaactcg

24601 aagttgttga tgtttatatc ggtcgatcat agaagaaaaa ttctgattca tttcgatcaa

24661 acttccttcc tgttaatctg gaagttcttc tcagatacaa ggaaatgatt cagttccaga

24721 gccaaagatc gtagttctcg aacgagcaat cgaaaagatt ctggcgcatc ctcgggttta

24781 agtattgttc cgccaatgat tgtagttcca agtacttcct gacgagctct aatatgatcc

24841 gatttataag taagcatctc ttgtgaaata tgagcaacac caaatccctc tagagcccaa

24901 acttccattt ctcctactcg ttgtccccct tgcttggccc ttcccctaag gggttgttgt

24961 gtaacaagtg cataatgccc gctggaacgt ccatggattt tatcatcaac ttgatgaatt

25021 aatttcagga tataggactt tcctattata accggttgtt caaaaggatc tcctgttctt

25081 ccatccaata ttctgctttt tcccggatac tcgggttcaa atacccacgg atttgctgtt

25141 tgcttactgg ctgaatataa ttcaggaaac actagctttc tcgaagcccc ctgctcatat

25201 ctctcatcaa agggtgctat tctataatgt ctgtccaaca ggtaccccgc taaaccgagc

25261 gaacattcaa atatttgtcc cacattcatt cgcgaaggta ctcctaatgg gttgaagacc

25321 atatcgaccg gtgttccagc ttgcaaataa ggcatatcct gtctaggcaa aattttggaa

25381 acgataccct tattcccatg tcttccagct attttatcac ctactttgat ttcacgtttc

25441 tgtaaaatat atacacgaat cgtttctgga ttataactgg aacccccctt tttctggatc

25501 catctcacat caataactcg accccttccg cctataggta gttttagaca agtttccttt

25561 gcggtggata cctgaatgcc aagtatggct cgtaataatc tatcttccgg ggcatacgat

25621 gattctttcg ccgcctgagg cgttaattta cccactaaaa tatcacctct ttctatccaa

25681 gatcccagca ttacaattcc atttttgtct aaattgcgga gtaaatgggc ttctaaatgc

25741 ggtattttat tagtgattct ttcggggcct tggcttgtca catgagtctg aatttcatat

25801 ttccgtatgt gaaaagacgt ataaatatct ccatatacca gacgttcgct aatgagtacc

25861 gcgtcttcag aattgtagcc ttcccatgac atatgagcta ctaatacatt ttttcccaaa

25921 gtaagttcac cgccaactgt agccgcgccg tccgctaaaa tttgtccctt tttaatgcat

25981 ttaccccgcg gagcctgagg tttttgatgc atacaagtat ttttgttgga acgttgatac

26041 ataaccaacg gaatgcttat agtgtctcca ttacccgata aaacgatctt gtccgtatcg

26101 gtataaatga tctttccctc gtgttcggct atagccgaaa cccccgaatc tagagccgct

26161 tggcattcca acccagttcc aacaatgcac ttctcggatc gagaaagcgg aactgcttga

26221 cgctgcatat tagaactcat taaagcccga tttgcatcat tatgctcgat aaagggaatg

26281 agggaagctc caatagaaaa atattggaag ggaaaaatgc ttcgaagatg aatctgttcc

26341 catgcaatag tcaagaattc ttgacggtat ccggctggaa caacctgttc ttcctgaata

26401 ccccgattca aagccaaaga atttcctgcc gctaccatat agtattcatc tctacttggt

26461 gataaataaa ccatcttttt tgatctctca gatatttcat aaaacggact ctctatagac

26521 ccccaatgac caatcctcgc atgaatagct aaggatccaa taagtccaac attgattcct

26581 tcggacgtgt caattgggca aatacgtcca tagtgactag gatggatatc tcgtatccga

26641 aaactagcag ttcgccctgt taatcctcca ggacccaaat aactcaattt tcgcccatga

26701 actatttgtg tcaatggatt agttcgatcc aaaacttgag ataaagggtg taggccgaaa

26761 aacgattcat aagtggttgt taatggagtt ggagttacca aattatgagg agtcggtatc

26821 aatttatgcc tgattgctcc acatatagtt cctcgaaccg cattttctaa acgaaccaga

26881 gccaatccga attgatcctg taacagatct gctacagaac gaatacgttt atttttcaag

26941 tgattcatgt cgtcaagtgt gcccattcca aatttcattc cgatcaaatg atccacagca

27001 gccaatacgt cttgtggtaa caagaatgta ttgttcggag gtatatcaag attcagtctc

27061 cggttcatat ttcgtcgacc aatccttcct aattcacatc tttgttgaaa aaatttcttt

27121 tgtaattcct tacataagga ctcagaaaat accggatccc cgcctacaca agcaaattgt

27181 tgataaaact ccaaaatggc attttccctt gacccaatct tttttttctc cttatgattc

27241 gggaaagaca agaaaatttc agggtagcaa acattatcta gaatttctct tagattcgaa

27301 cccatagccg atgatggaac taggatagat attttttgtt tcctactcac acgggcccat

27361 atccttgctt ttctatcaat ctctaattct gatcttcccc cccaatctga tattatggtg

27421 ccggtataga cagaaattcc gctatggtcc aattctgaac ggtaataaat accggggctt

27481 tgcaatattt gattgattac aattctgtat attccactta ctagagaggt tcccagggaa

27541 ttcattagag gaatatttcc aataaatacg gtttgttctt gcatatctct accggttttc

27601 caaattaatc ccgcggatac atataattca gaagagtatg tgagtgattc atacacagca

27661 tctctttctt ttatcaaggg ctctgccaat tgatatgttg ccacaaataa ttgaaattca

27721 atttcttggt ctgtatcttc aatttttgga aacttatgaa gttcttccat caagccttga

27781 tcaatgaacc tacaaaatcc gtcaaattgt atctgactaa accctggtat tgtatacatt

27841 ccctcatttc catcccggaa catcttaagt tttccgttta tcgaaaaaat ccaactattg

27901 gctcactctt cgttgaacca tatagattga tctagcaacg atggaatgta tattttgctc

27961 atttgaacaa catgaaattt tatccaaccc catatacata tatacatgta ctaaatacgt

28021 atgaacggag gaataaaaaa aaatgtgact caaattcgaa tttgcgacag atacaaatgg

28081 aaatgaattg ataaaacatt cctggaaaca aaattctgcc acttagactt atggagtctt

28141 gtatagaata tcaaaataga tccaatttct accttatgat attacgatca gattgggtac

28201 cataaatgga ttcggaattg aatctgttct ctatgagtga gataaagaca gaataatcag

28261 gaaccgtcta gagttgactt tgattttagc acttaattta tttcatcgat tccgttgttc

28321 aaaaaatgat tcgcagagag aaaagatatt tctacccatt tgttaattag tagaatacga

28381 ttgaagtgcg taagagaagt catatttatt aagtacatgc agatataact atctagctat

28441 ccgtatatcc catcttttat cgaagttccc ttgaggcaac ataggtcgtg ctatatccaa

28501 atttctattt tatattcaat atattcaatg aaaaatgcaa gcacgacgat ttcctaatag

28561 gaatatgtag ataagatacc tgactaggta tccgtgtaag aatttctgtt ctggggttta

28621 catatacaca taattgttgt tataattgaa attgaaaagg attaattatg gaaaagaatt

28681 gagactgatt agtcgtatat caatttgatc tccttatgtc attaaggaaa ccaaattgga

28741 gatcaaaacc caagaaccat tcatgaattc acagtcatta atgcttccaa tttgctctga

28801 attttggatt ctgtgactgg aaatccattt ttctcaatag aaaaaggggg gaaagctttt

28861 atttaggtgt tgtgtgtttt gaaatacaat caatctaaga gaacaacagg atccaatcaa

28921 aaaaaagaaa tggttcagca attccccaga atatttccat ctatatctat tttgtatcgt

28981 tttggcggca tggccgagtg gtaaggcggg ggactgcaaa tcctttctcc ccagttcaaa

29041 tccgggtgtc gcctgattaa caaaagactc ggaatttctt accctactaa actaatagaa

29101 ctcacaaaat tcttgcctgg cagaagcaga ggtaaggggc ggggactgtc gatacccaat

29161 tttaagaatc ggggggttga ctttcaatta tttcttcaaa aatcggggtg tgacccaaac

29221 ctgtaccata ccaatatgaa ttaccaatat aaataaagaa atactcactt aattacggat

29281 tgctgatgcg ttcaggccat tgatttgatt tatcaatcga atcaaatcca tagtaagatt

29341 caattgtgag attcagaact acgaaagtca gggaccgtaa atccgtgtat ccaaaggaag

29401 gttcctaaga gactgtaaat ccttgctcct aggatccaag aaggggttct aggaaggact

29461 ataaatactt cctaattacc ttaccctact agtgtttact gactgagtct tgaagtagat

29521 tgggtaggct ggtggggaat ccaattagga gttgtggaaa gaactgaaga tactttgtat

29581 ccatacaaac tcatgagagt ctctgagtgc tcaggttttc aattaatatt gtatgggtga

29641 ttggctttta tagaataaaa gtggaaaaaa gtgctttcgt tggggtaacc ccgccaagaa

29701 tgtaataggg tgtcttccaa ttgtttcaca tttcacagaa gtagagacag taaggcttag

29761 atgggaaatt aggagtatgt gatagatagt tatatatctt gatggtatat tttttttttc

29821 tgctttttgt ttatgaaagg caacaatagg tcttactatt cctacatatt ccattagtca

29881 cattcccttg agacttccaa ggggcaactg tgtatcttgc ttgtacttag tgctttccga

29941 ttccaccaga aatcatatag ggacttgtta cgggtgtatt cattggattg gttcatcaaa

30001 aacgttaggt caaaatccca ttttgactct gcaccattga ttccactatt attagtgatc

30061 aagaatggaa taattccttc atattcatag agatagggga cacgattcac atggatatag

30121 taagtctcgc ttgggctgct ttaatggtag tctttacatt ttccctttca ctcgtagtat

30181 ggggaagaag tggactctag gggtactact aattgagttg agtaatcgaa tttatcaatt

30241 gtttaataga tcgttctgca aagcgctttt aaatcaaaat atctccacct cataaattct

30301 actggaatcc aatatgaata agaacctttc gatcaaacaa atatttcaac gacttgattc

30361 ccatattcgt atttcgaaac tcaaagggat acacatgatg ggaaattttt tccaaccgaa

30421 ttctttctaa atattctatt tcgacaaatc ggcccttact agaattatgc atattacaat

30481 gaggagcaac caacccctat tttttttttt tatttgtttc ccttttctct ttgctgttca

30541 aagagggaac cgttcttcta ttacgtacgt ggatatgtac tttctactga ggcgacatag

30601 acatagtcgt tgtccaaaga ggtactacgc ctaataagat cttactttcg ttgggtatgc

30661 gtacttacct ttttatactc ctaggaatct tatttatgct ttatcgactc gtctcatgtc

30721 atggttcaag catgaaaaat cggtggggtc tactacatcc ttttcaaaat ccgaagaagt

30781 tactatagaa ctttttggat catccgttaa cggatcaatc aattacttct tcgtaatgct

30841 aaaaaaaggc ttggttttct tttatataat atatgcccat actagtcttt ctccattgat

30901 tctttcaatg gatccccgga tccatattga aaataatcag aaacccagga attagaaaag

30961 ttgacgttcg attatttcag attgatcggg atcaatacaa attgacgtaa caaagaaata

31021 gaattggagt gctatttaca tgtacatata taaatgtggg tacatattgt ggattgatct

31081 atatcaagct catacctttc tacaataata gatagtgtgg tagaaagaac tataatgaac

31141 ctttctacca tactatctat tatactgctg actccaaccc gaccatttaa gacttggaat

31201 ttgaatccct ttctttcatt tcttcaatcg ttgataagaa ctaataagtc aagtttcagt

31261 caaattaatc actttgactg actgttttta cgtagattat aagtaaaaaa gcagtaggaa

31321 ctagaatgaa caacgcaata gcaataaatg ctagaatatt gacttccata atctcatcgt

31381 ttttttttgc ttcgcaataa ctcgggatct aatcccatag agatgataag tctttctcct

31441 gtaaattcaa taggatagat tgtatcctga tgatacttaa tcgtatcaat atcatgaata

31501 acaatatctg atctatcaaa tcgattcatc gtcgagaatt gaatagtata acataggaag

31561 atcttttatc cataccgaat ccaaaattgg attcctggtc caatcaagaa tcccattgaa

31621 tttctcattt ccactctttc ttttttataa cctgccgtct tccttataca atcatctgac

31681 cggcgttcca ttggtcacaa acccaaacgg tagggatgaa atggaaaaag gaatgagtta

31741 agttctaaac gaagtttttg tgaagatcta ctctttttgg aagacagaga agtgtgataa

31801 agattggtcc ggtagaaaag atctaacaga atattctatt ctgacaaatt catttattta

31861 ttgattttgt tttttcttcg atggggccat taaaatagga agaaaaaaaa aagggggggg

31921 ggtaggtttc atctgaaaag tactctgtcg ctgtcggggt aactagtaac tatactatat

31981 taattaaatt aattgcgtat cgtacaataa acgaatacaa tttgtgtatg tgctcccggg

32041 aaacgtatga gtactctatt acatggacca ggagcaatcg aaaaagacag gcccgtaggg

32101 tctctcttga aatctgaata gggcgatacc gccgatcaat ctacatatgt ctctccccat

32161 caatcggtac tagttgaagt aattgaaagt cccatatttg tacgatgaga aatgcgaaac

32221 gaaaaacacg aaagaaataa ggatcccccg gggattaaat cctgctcctt gtcccccctc

32281 ttcgcagaaa atggggagat gagttgatgg attcatcgga ttctaggtcg ggactgacgg

32341 ggctcgaacc cgcagcttcc gccttgacag ggcggtgctc tgaccgattg aactacaatc

32401 ccgggaaatg gggtgtacag catacataca tattcttata atttcattcg aaccctttct

32461 ttctattcta tattagattg aaaatcgaca tctttctgtt acaagaaaga cgagtgatat

32521 actgatatac acatggatat ggactatagt gggagtgaca cggattacta gtaatcctgt

32581 ggttatttta ttacccaatc aattgataat ccatttttca atgaaaaaaa aaaaggactc

32641 tttatttcta tctatcaggc atttcattta tagaggacaa actggttata tcatcctcat

32701 ggatcggcga attgttgggc cgagctggat ttgaaccagc gtagacatat cgccaacgaa

32761 tttacagtcc gtccccatta accgctcggg catcgaccca ggaagaatca attctaggct

32821 tattgataat ccatgatcaa ccccctttcg tcttaccccc aggggaagtc gaatccccgc

32881 tgcctccttg aaagagagat gtcctgaacc actagacgat aggggcatac ccgcccgatc

32941 gccatcatac tatctatgct catagtatga gcagtttttt gaaattgtca atatacaata

33001 tatatgacta gatccgaaga atctttcttg cttacaagat tccatagaat ggaattttgg

33061 gattgttgat tcatgaacca tcctatatat aagagaggat aggatccttc agggagtgat

33121 ttgtccgaca gaaaaagggc aaaccccatt ccatttcttt cattttcact cgttgattcg

33181 ttcgtcgtta aggtgagata tgcctatctc acactaacac taaactaagc caggaaattc

33241 agaaacgata gaatttcttt ttttgaggat cgacgaataa tcgaaaagat tctttttttt

33301 tttttctaat aatttaattt agggtacgaa tcgaatccct tcatcacatg attcgatgaa

33361 ataccttgga tctatatcgg attggtacat gtatcaatca accaagcgaa tctcgtccgg

33421 atgaatcaat aaaagcaaag caattaggag cgtccctgaa acaattcatt gcattgatat

33481 ttctcaaata tcaataacta aaacttccta ggtaaatcaa atttattgtt cctgaatgag

33541 cccctatgta tacatgtaca ttatatacat atacattgta gtacatacat agatatatgt

33601 agtagactct atagtagcta gtgattaatt cattttttga agaaaatggg cccttttaac

33661 tcagtggtag agtaacgcca tggtaaggcg taagtcatcg gttcaaatcc gataaagggc

33721 tttttctacg aagctccagt cttcgtcttc atttttcatt ggagaataga gatattgttg

33781 atatttgtaa taaaagtaac ccataatgag ttatcattct aatgagttat aggtataaag

33841 tgaaacagtt gtttattatg attatgataa gtaatcgtac ttagtaggag gactactatg

33901 taattcacta caagctatac ccctcctcat attattccta tttttggtcc tgggacatag

33961 atattctaga tacccaatcc aaattgtgaa tcgccaaacc aaagtattcc catttctcta

34021 ttgttccaat caaatccctc ggaaaaatta gaaatcaaga aaatcaaaaa gtaagtggac

34081 ctgagccatt gaatcatgac tatatcagct attctgatat tcaaattcga tagagatgaa

34141 attgtagaag cgaacttttt ctttccttgg accacgcaat aatttgtcga tatttcagat

34201 tgaatcttct tgttcctgga tgctccatag gaataaattg ctcttccttt cctccacaga

34261 gatacgttta ttccaagtca caagagcaat ctctttttca atacctttct ttgattccag

34321 aaaaaaagaa gtttctatct atataggatt tagatataga tatcaaatca tggcttcagg

34381 tacaaaatat ttccatattg atgcatcaga tatttttgtt cctccaatgc aacggaaaac

34441 gagtgcgata aaggagggat tttgatttcc agtctcccta tttaatttag ggggcaggga

34501 caaaaaatag ggtccttttt tttctgccgg atatagggta ataaaaaaaa gtaaagaggg

34561 aaatattcga agtttatttt ttttggttcg acccgcgaaa agatatactc tggaatttta

34621 gattcattcg aaggaaatat aacaaagaag acaataacaa acaaaaagca atcaaaaaag

34681 gaaggagtaa gaaattatat atatatatag gatactgtag tagtttagta tacacataaa

34741 ttacgagaat ccataaagat atttattgat cttttctcaa taagatccaa gaacaagaat

34801 acgattagct tatggaatgg cgagctagat ctggggagca actgataacg agagaaagga

34861 tcgcttgttt cctcacagtt atttcaaaaa atggatctga ttgatgggtc ataagacaat

34921 tcagggttcg gatggttatt aagaataaga aggaataagg aataggaagg aataattgaa

34981 tcgaactcat ggatttacct aggttggttt ctggcccaat agaaaggaag gatttgtatc

35041 ttcgaaaccc attggatgga aggcgcagtg gacgaggaat cgttcataga tgaccgaacc

35101 atcgtatgcc ctgagaatga tatgaggtgt tcggaaatgg ttgaagtagt tgaataggag

35161 gatcgatatg actatagccc ttggcagatt taccaaagaa gaaaatgatt tatttgatat

35221 tatggatgac tggttaagga gggaccgttt cgtttttgta ggttggtctg gtctattact

35281 ctttccttgt gcttatttcg ctttaggcgg ttggttcaca ggtacaacct ttgtaacttc

35341 atggtatacc catggattgg ccagttccta tttggaaggc tgcaatttct taaccgctgc

35401 agtttctact cctgctaata gtttagcaca ttctttgttg ttactatggg gacctgaagc

35461 acaaggagat tttactcgtt ggtgtcaatt aggcggtctg tggacttttg ttgctctcca

35521 tggagctttc gggctaatag gtttcatgtt acgtcaattt gaacttgctc gatctgttca

35581 attgcgacct tataatgcaa tcgcattttc tgctccaatt gctgtttttg tttctgtatt

35641 cttgatttat ccactaggtc agtctggttg gttcttcgcg cctagttttg gtgtagcagc

35701 tatatttcga ttcatcctct tcttccaagg gtttcataat tggacgttga acccatttca

35761 tatgatgggg gttgctggag tattgggcgc tgccctgcta tgcgctattc atggtgctac

35821 tgtagaaaac actttattcg aggatggtga cggtgcaaat acattccgtg cttttaaccc

35881 aactcaagct gaagagactt attcgatggt cactgctaac cgcttttggt ctcaaatctt

35941 tggggttgct ttttccaata aacgttggtt acatttcttt atgttatttg taccagtaac

36001 cggtttatgg atgagtgcta ttggagtagt cggtctggcc ctgaacctac gcgcctatga

36061 cttcgtttcc caggaaatcc gtgcagcgga agatcctgaa tttgagactt tctacaccaa

36121 aaatattctc ttaaacgaag gtattcgtgc ttggatggcg gctcaggatc agcctcatga

36181 aaatcttata ttccctgagg aggttctacc ccgtggaaac gctctttaat ggaactttag

36241 ctttagccgg tcgtgaccaa gaaaccactg ggttcgcttg gtgggccggg aatgcacgac

36301 ttatcaattt gtccggtaaa ctactcgggg ctcacgtagc ccatgccgga ttaattgtat

36361 tctgggccgg agcaatgaac ctattcgaag tggctcattt cgtaccagag aaacctatgt

36421 atgaacaagg attgatttta cttccccatc tagctactct aggttgggga gtaggtccgg

36481 gtggggaagt tatagacacc tttccatact ttgtatctgg agtacttcac ttaatttcct

36541 ctgcagtctt aggctttggc ggcatttatc atgcacttct aggacctgag actctcgaag

36601 aatcctttcc attcttcggt tatgtatgga aagatagaaa taaaatgact acaattctgg

36661 gtattcactt aatcttgtta ggtataggtg cttttcttct agtactcaag gctctttatt

36721 ttgggggcgt atatgatacc tgggctcccg gggggggaga tgtaagaaaa attagcaact

36781 tgacccttag cccgagtgtt atatttggtt atttactaaa atcgcctttt gggggagaag

36841 gatggattgt tagtgtggac gatttagaag atataattgg aggacacgta tggttaggtt

36901 ccatttgtat acttggtgga atttggcata tcttaaccaa accctttgca tgggctcgcc

36961 gagcatttgt atggtctgga gaggcctact tatcttatag tttaggtgct ttatctgtct

37021 ttggtttcat tgcttgttgt tttgtttggt tcaataatac cgcttatcct agcgagtttt

37081 acgggcccac cgggccggaa gcttctcaag ctcaagcatt tacttttcta gttagggacc

37141 aacgtcttgg ggctaacgtg ggatccgctc aaggacccac tggtttaggt aaatatctaa

37201 tgcgttcccc gaccggagag gtcatttttg gaggagaaac tatgcgtttt tgggatctcc

37261 gtgctccctg gttggaacct ctaagaggtc ccaatggttt ggacttgagt aggctgaaaa

37321 aagacataca accttggcaa gaacgacgtt cggcagaata tatgactcat gcccctttag

37381 gttctttaaa ttccgtgggt ggcgtagcta ccgagatcaa tgcagttaat tatgtctctc

37441 ctagaagttg gttagctacc tctcattttg ttctaggatt cttcctattc gtgggtcatt

37501 tgtggcatgc gggaagggcc cgtgcagctg cagcaggatt tgaaaaagga atcgatcgtg

37561 attttgaacc tgttctttcc atgacccctc ttaattgaga caggagatca aatgcatgaa

37621 gtaggaatcc atttgattcc attatacata ttaggttaag atcaggtcat atttaaaaag

37681 tattccttgt tttttccttt tcattctatc tatttttttc tggctcggct atcccaccta

37741 gccgagccat ttccctttat gacaccgggc caggccatac caataaagaa acaaatcgat

37801 tcaacgagca aaaggagaga gagggattcg aaccctcgat agttctttgt tcggaactat

37861 accggttttc aagaccagag ctatcaacca ctcagccatc tctccaagag acaatctcca

37921 ttttattcct ccgaatagaa catggccata tgggttgata ctctaactat ctgtagaaac

37981 atcccaagtg cgaatctata tttcgacata tctatctgtc tatagatgca tgatccaaca

38041 tgcccatttg ggaagtcaaa aaaaaattcc ctgattccat gtccgaataa aataaagtgg

38101 gactaagttc gaaaggatca ataaattcat ggtcaaatcc cgtcatgatg cattatttca

38161 attttgactc tgagagaggg atcaaatggt atagttcatt tgttggtagc ttggaggatt

38221 acaagcatga ctattgcttt ccaattggct gtttttgcat taattgctac ttcatcaatc

38281 ttactgatta gtgtacccgt tgtatttgct tcttctgatg gttggtcaag taacaaaaat

38341 gttgtatttt ctggtacatc gttatggatt ggattagtct ttctggtagc tattcttaat

38401 tctctcatct cttgaaccta ttcggtcttt cccggatcaa aaaactgacc cctccccaaa

38461 ttctttcgga ttgtaagaca cattaaaatg aaatatgagt ccaaaaataa aaataaaaaa

38521 attggaggga ggggtcaaaa atcacttctt gaataaaaaa aacgaagaat ctaataataa

38581 ttggaatctt cctaagtatc tgaccctgtc tgtacaaatg ggatccagac acatatatga

38641 tatatcatat atgtgtggac atatacgtgt gtatcaggaa cgaagaaagt gcggatatgg

38701 tcgaatggta aaatttctct ttgccaagga gaagatgcgg gttcgatccc cgctatccgc

38761 ccatggtaaa gtaaggtaat atgataaatg atttaggtat agttgaccac gataggggag

38821 tggttctatt cttcccatcc caaaaccaaa atagccattg gttactaggt aacggaatcg

38881 cacctaaaaa tgtttttttg aaaaaaaaaa aaaagagatg ttgcggagac aggatttgaa

38941 cccgtgacct caaggttatg agccttgcga gctaccaaac tgctctaccc cgcgctgaaa

39001 actaatggac gaacaagaat tggatgtgcc cccataccat attctatata aatagaatag

39061 cccatttata cagaatggta aagggggccc ctctatgatc atagctcata gagataaata

39121 gaaataggaa gaaggggtat ttttatcctt accaacttga tcttgtggcc cccggcaaca

39181 aacatgcctg aaccttttcg tgaagtatgt gtccggatag ccgaaagtct cgatagctag

39241 ctctaggtct tccggtcaaa aaacaacgtc gatgaagacg tgtaggtgta ctattacgtg

39301 gtggggattg caattttcca tgaatttccc atttgtcact caacgatgaa actttgccta

39361 tttctttttt tgaggatcga cgaatcaaat gatatttctg ttccaatttc tgcttctttc

39421 tctccctctg aatcaaactt ttccttgcca taaaggttca gttcctatta ttatcaatga

39481 tacgggtcgg atcctagatg tagaaataga agaaggtgga ttctcccttc tccatcgaat

39541 caaatgaaat tgtcgatgat acagcacatt aaaaaaaaaa attaaccaaa tttgcctgat

39601 gtagaggcaa tcaagaaagc tgcataagtg aatatataac ctacagaaaa gtgggctaat

39661 ccaaccaatc ttgcttgcac aatggaaaga gccactggtt tatccctcca tcgaatcaaa

39721 ttagccaaag gcgtgcgttc atgagcccat gctaaggttt caatcaattc ctgccaatat

39781 ccacgccagg aaattaagaa cataaatcca atagcccaaa caagatgccc aaataggaac

39841 atccatgccc agaccgataa actattcata ccaaaggggt tatagccatt gataagttgt

39901 gaagagttta accatagata atctcttaac catcccatca aataagtgga ggattcgtta

39961 aattgtgaaa cgttaccctg ccataatgtg atgtgcttcc aatgccaata aaaagtaacc

40021 catccgatag tatttaacat ccagaaaacc gccaaataaa atgcgtccca ggccgaaata

40081 tcacaagtac cgcctcgtcc cggaccgtcg caaggaaaac tataaccgaa atctttttta

40141 tctggcatta acttggaacc acgtgcatct aaagcacctt ttactaagat caatgtagtt

40201 gtatgcaaac ctagagcaat agcatgatga accaagaagt ctccaggacc tattgttaag

40261 aatagtgaat tactattctc attaacggca ttcaaccagc ccggtaacca tatacttcga

40321 ccagcattga atgccgggcc attcgttgaa gataaaagta catcgaaccc atatgaagtc

40381 ttaccgtgag cggattgtat ccattgggca aatatgggtt cgatcaagat ttgtttctcc

40441 ggagtaccaa aagcgagcat gacgtcgtta tgaacataga gtcctaaggt atggaaccct

40501 agaaagagac tggcccaact taaatgagat ttgatagctt ctttatggtc taacattctt

40561 gccaatacat tatcctcatt ctgttccgga ttgtaatctc gaatgaagaa tatagctcca

40621 tgagcaaagg cccctgtcat gatgaatcct gcgatgtatt ggtgatgagt atataacgca

40681 gcttgagtag taaagtcttg tgctatgaat gcataagcag gtaaagagta catgtgttga

40741 gctaccaagg aagtaataac ccctaaagag gctagagcaa ggcctaattg aaaatgaatc

40801 gaattattga ttgtgtcata aagaccctta tgcccacgtc ccaatcgacc ccccggagga

40861 atatgtgctt ctaaaagatc tttcatactg tgcccaatcc cgaagttagt tctatacata

40921 tgaccagcaa cgagaaaaat aaatgcaata gctaaatgat gatgagcaat atcggtcagc

40981 cataaacttt gcgtttgtgg atggaatccc ccgagaaggg ttagaatggc agttcccgcc

41041 ccttgggagg taccaaataa atgacgactg gaatcggggt tttgggcata aagattccac

41101 tgacctgtaa aaagtgggcc caacccttga ggatggggta atacatctaa gaaattattc

41161 catctgacgt actgccccct tgatcctgga atagcgacat gaactaaatg ccctgtccaa

41221 gccaaggaac tgactccgaa gagtcctgac aaatgatgat tgagacgaga ttcggcattt

41281 ttgaaccacg aaacgcttgg tttccatttg ggttgtagat gtaaccaacc cgcaattaaa

41341 gatatggcag aaagaaataa tagaaaaaga gctccagtat aaagatcttc attggtgcgt

41401 aagccaattg tgtaccacca ctgataaaca ccggagtaag cgatattcac tgggccgaga

41461 gcccctcctc gagtaaaggc ttctacagcc ggttgaccaa aatggggatc ccaaattgca

41521 tgagcaatag gtcttacatg taaagggtcc tgtacccatg actcaaaatt tccttgccaa

41581 gctacatgaa acagatttcc ggaagtccac agaaagatta ttgctaactg cccgaagtga

41641 gaagcaaaaa tgttctgata aagacgttcc tcagtaatat catcatgact ctcaaaatca

41701 tgtgcggtag caataccaaa ccaaatacga cgagtagtgg ggtcctgagc taagccttgg

41761 ctaaactttg gaaatcttaa tgccataatg cctttcaaat cctcctagcc attatcctac

41821 tgcaataatt cttgctaaga agaatgccca tgttgtggca attccaccca gaaggtaatg

41881 ggttactcct acagcgcgtc cttgtacaat gctcaaggct ctaggctgag tagcaggagc

41941 aacttttaat ttgttatgag cccaaacgat ggattcaatg agttcttgcc aataaccacg

42001 gccgctgaat agaaacatta aactgaaagc ccagacaaaa tgagcaccta ggaaaaaaag

42061 gccatatgcc gataatgaag aaccataaga ctgaattacc tgagatgcct gtgcccataa

42121 aaaatcccgg agccacccat taatagtaat ggaactctgt gcaaagtttc ctcccgtaat

42181 atgagttacc accccttgat cacttatact accccaaaca tctgactgca ttttccaact

42241 gaaatggaat attactaccg aaatagcatt gtacatccag aatagaccta agaagacatg

42301 atcccaggca gatacttgac atgtcccccc tcttccaggt ccatcacaag ggaaacgaaa

42361 accaagattt gctttatcag gtatcaaacg tgagctacga gcaaatagaa cacctttcag

42421 tagtatcaat acggtcacat ggatcgtaaa tgcatgaatg tgatgtacca aaaaatctgc

42481 ggttcctaat ggaataggta acaaagcgac tttgccgcct actgctacta aatcaccacc

42541 cccccaagtc aagctggtgc ttgttgttgc accaggagct gttgcaccag gtgctaaagc

42601 gtgggtgttt tgtacccatt gagcaaagat gggttgtaat tgtatagcgg tatctgaaaa

42661 catatctcgg ggacgcccta aagcgctcat ggtatcatta tgaatataca agccaaaact

42721 gtgaaagcct agaaatatac atacccagtt gagatgtgat atgattgcat ctcggtgtct

42781 aaggacacga tctaatagat cgttgtatcg agtagttgga tcatagtctc ttaccataaa

42841 aatggctgca tgtgcagcag caccaactat gagaaatcca ccgatccaca tgtgatgtgt

42901 gaacaacgaa agttgtgtac catagtcaat agctaggtat ggataggggg gcatggaata

42961 catatggtga gctacaacaa tggttgaaga gcctaacata gctagattaa gagataattg

43021 agcatgccat gacgttgtta ggatctcata gaggccttta tgaccctggc ccgtaaatgg

43081 gcctttatgc gcctctaaaa tatctttcag gccatgacca atgccccagt tggtcctata

43141 catgtgacca gctaccagga aaagaattgc aatagctaaa tgatggtgtg caatatctgt

43201 cagccataga ccccctgtta ctggatctaa tcctccacga aaactaagaa attccgcgta

43261 ttttgaccaa ttcaaggtga aaaatggggt tgatccctcg gcaaaactgg gataaagttg

43321 agccaaaaga tcccgattca agataaattc atgaggaagt ggtatctctt taggatccac

43381 tccagcgtct agaaattggt taatcggtaa agatacatgt acttggtgtc ccgcccaaga

43441 aagagaccca agccctagta accccgctaa gtggtgattc aacatggatt ccacatcttg

43501 gaaccaagcc aattttgggg cagctttgtg ataatggaac caaccggcaa aaagcattaa

43561 ggctgcaaag accaatgcac cgattgcggt acaatagagt tgtaattcat tagttattcc

43621 agatgctcgc caaagctgaa aaaaaccaga ggttatttgt attcctcgga aacccccgcc

43681 cacatcacca ttcaatattt cttgacctac tattggccaa acaacctggg cgctgggtcc

43741 aatgtgagta ggatcactta gccatgcttc ataattagaa aaacgggcgc catggaaata

43801 cataccactc agccaaagaa aaatgatgga gagttgaccg aaatgagcac taaatacttt

43861 tcgagagatc tcctccaaat cactggtatg gctatcgaaa tcgtgagcat cagcatgaag

43921 gttccagatc caagtggtag tatcagggcc cttagctatt gttcttgaga aatggccggg

43981 tctggcccat tcctcgaaag aagtttttat gggatcccta tccaccaaaa tcttcacttc

44041 tggttccggc gaacgaataa tcattgagtc ctcctctttc cggacaacac atacaaagaa

44101 acccgccaac agtcaagtaa ttagtgaacg atgggtattt atgattagtt ccttatcttt

44161 cctatccccc atctatcttt ttttttttta gttatttact agagcaatta tgatatggaa

44221 gtcgatccgg ggcaagtgtt cggatctatt atgacataac catggggcgc tcaacggacc

44281 tttataatat tttataaccc cctccggcgt gacacaaaaa cggatttttt gatacaagct

44341 agttagtgta ttcatatctc aatgtatagt atctagatgg atctacttca tatcttacac

44401 ggaacatatt acttacaata caaatcaaag gatcattcat tagtcattaa taagagacat

44461 cttgatatct atatttagtc attcgaggtc tgtctttttt actggcttag ctttattagc

44521 atagcagaag ggaatatttt ctgtactgta tccgtatcgt ttatccctat gacagacgaa

44581 atagaacaat cttagactta gaagggatat aatgaaattc cctgattggc tcttcctaga

44641 ggaacgatct attttatttg attgatggat cccatattat aatgaattca aaaagagaat

44701 gttcttattc aaacctcctt gcgatcttca accaattatg tgcttcaata taattccccg

44761 gagtaagcgc tatagcttgt ttccaatatt cagcagcttg atcggaccaa gcctctgcaa

44821 tttcagaatc tccttgtcga atggcctgtt ctccccggtc ggaataggtg ggtcaattcc

44881 ttcccttaga accgtacttg agagtttcct acctcatacg gctcgacatt cttttggtgt

44941 tccatcttaa tctaccatat ctaactgaat gagatttctc ataaatctat cccatttttt

45001 ttttttcggg ttaaccagaa gaggttaatt acacgagttt caaactctaa ttttgatcaa

45061 taatcagttt tctcttttct cccaccttca gaagaaccaa gcataggtat tttcctctat

45121 cgttcgaatt ttctgaaagg taactatctc ggtttcatat agaaattcat atagaatctt

45181 tgaaaaagac tttcctccat aagaaagaaa ggacttacta tctttgggat ctgatgctac

45241 accgctgctc aataccttag tagatcgact ctattacata agttgattcc taacttttat

45301 ctcatatcat gacattaagt aagcagtcct tattgtatcg gtccccgaac ctcactaatt

45361 gatctttacg gtgcttcctc tatcaattag atcctttatc catagaataa agtatatagg

45421 ccatacctat ttcttcatat ttcggctctt atgaagtctc tttctttgct acagctgata

45481 aaaatcgttg ctttggacga tgcatatgta gaaagcctat tttgtttcta gtattgacta

45541 gcggatttgg tctttccttc cttctttcta tagtgtagat agtcgcacgt aatgacagat

45601 cacggccata ttattaaaag cttgtggtaa gaatggattt cgttctattg cccggaaata

45661 atattccaaa gccttcgtgt gttctccgtt acttgtgtgg ataaggccta tgttatagag

45721 tatataactt cgatcatagg gatcaatttc tagtcgcgta gcttcataat aattttgtaa

45781 agcttccgca taatttcctt cggattgagc tgacatccgt tacggtcgtt cattctattc

45841 aaagaatctc cgttccagaa ccgtacgtga gattttcatc tcatacggct cctcccttct

45901 gtgcatagta ataaggggaa taatccatgg aatcaaaaaa gattgaaata ttctcattat

45961 gaactgacag gggctggtgt ttttacaaga aatctctagc cagccttcct gcaagaggtc

46021 tgtcttttct taacaccaag cgcgtttgtg ctagatagaa atggtaactc caacaatttc

46081 tttgtcctca acgccccctg tttccaggaa ttagtcactt caacgacctt tgatggttat

46141 acgggtatcc aaagtacgaa cgagatggat gtttgttgtc ccaaccattc ttgttagttc

46201 cgatcccgat aaggaaaagg gttaatttat aacaaagttt tcgtgttgtt gatttctaga

46261 tgtagtgctt cttcccctat gcggcctatt ggtactagtg gagtaggatt gacccgcaat

46321 acagaaccta taggtgtaac ctttcgctca atactagaat cgacagttga agcatctaag

46381 gctgcatcaa tcggggatac acgacagaag gaattgttct atctccaaac ttcaccttca

46441 tcaagcgtag gtttatttca agaatctttt ttctttgtat cccgaatcat gtctctttct

46501 cgtaagactg agggcggtaa ataaataaat tcaaaaaaaa aagcaaatcg caccatctct

46561 gtaataggta aatgcctctt tttctcctga ggttgtcgga attattcgta ataagatatt

46621 ggctacaatt gaagaggtct tatcaataaa atttccattt atccgagatc taggcatagt

46681 taacaatcca ttctagaatt cttctcatta cccctcaggg gaaaatgatt ccacaaacaa

46741 aggaattgta cagtacgaaa tcacataaaa acagactcat tctaaaaaaa aaaatgtgga

46801 ccttccactc aaattatccc tttttgagag gtatagatag gaaatatttg aatcggattg

46861 gatttcattg aaattgagta gtataccaat gaatggaact cttttatttt atcgaagtta

46921 agaaatccag gaatttttac taccgattct tataatttaa ttcgataaat ttggatttga

46981 ttatgatcca aagaggaaaa agaatcaaat aatcattcca tgatgaaaat agaataacca

47041 tccattttgt gtgcatagtg tggatacacc atccaatcga aagataaaaa tctatagaac

47101 gattcatgaa tttgtaatag atctatggag tagctcatga gaggagttgt tgttgagaaa

47161 tctgaaactg gaagggggga attttgtaat tcctatggaa tcgtagttta aatataaaca

47221 tagtctaaaa tagggtcagt tgactcgttc caattcattg gcttaatccg aaatattaga

47281 ataagatagg atcgtcgtat tgacaaacaa gacatttttg tttttaacaa gaaaaaagtg

47341 tgtttttttt atccctcgag cctcgaagga aaatcgttct ttagcgaaaa gttttctatt

47401 tctaatagat tggtcgtacc tgtattgcaa taatatgaat gactcgctat ttactcggtt

47461 tctggggcat aataataaga ttatgtagga gagatggccg agtggttcaa ggcgtagcat

47521 tggaactgct atgtagactt ttgtttaccg agggttcgaa tccctctctt tccgtacctt

47581 catctaattc accaaccgac cacaatgtat caaatcaaat aacaattgat accattattc

47641 caacagtaag acccttattt gatagagatt ctctattcct aattactgca gtacggaaaa

47701 taccggaaag agtggaaagg aatgaaaatc tcactgctga tccatttgtg atacgtgaat

47761 gggagaaaaa tccggatcaa accccttctt cggtgaaaaa aaaaaagagg ggggggggca

47821 aaatggtccg aagctttgtt attttagtta ggttcaagtc tgacgggaat aatattctac

47881 gactagaaac tcattgattt tcaaaccgat ccatttaata tctattattt gatttactaa

47941 tcctttatat tgggatgagt caaaagtcaa atgttttgcc aaatcctcgc ggggcgatga

48001 atcaagataa ttttgaatca gagctctgga tctttgttca tcccttgcag taataatatc

48061 tcggggtttg cagcgataac ttgggatatc tactacacga ccattaacta aaatatgtct

48121 atggttaact aattgcctgg ctccaggaat ggtcgaagcc atacctaatc gaaaaaggat

48181 gttatccaaa cgcatctcaa gtagttgtag taaaacctga cctgttgacc ctttggcttt

48241 tccggcaata cgaacatatc taagcaattg tcgctctgtc agaccataat gaaaacgcaa

48301 tttttgtttt tcttctagac gaatacgata ttgagatctt ttcccgaaac gtgattggtt

48361 tctaagatca cttccggatc taggtctttt actagttagt cccggtaaag cccccagaca

48421 gcgtattttt ttgaaacgag gccctcggta acgagacata aagactcctt gttaaaattg

48481 tattttacag aataaactta aattaagact gaactaaacg ataaacgaaa ctaaatctat

48541 tgaagtacta caaaagaaga ctacaaaaga agaatgagat gaattgtatc aatatccgga

48601 ttattttgta tatataggaa gtgaaggacc cctttcttga tttgttctgt agtgtagaga

48661 tttaactgct ccaatcaaat aagtttttta ttcatagttg gaagttgcta cgacataata

48721 gatcggtgac ccgacatttt taaaagaaaa aaagagagga gtcttttcaa tattccttga

48781 gatcaaggaa tattgaaaag ccggctatcg gaatcgaacc gatgaccatc gcattacaaa

48841 tgcgatgctc taacctctga gctaagcggg ctcacataac agaaataagt gcaatagaac

48901 taactaacta tatctatata gaatgttttt tttaattctt aatttatata ttatacttaa

48961 tttatagcag ttagttatag cagattagat taatcatatt agagcagatc ggtactaagg

49021 aaaggataag ataaggatgc aatccagatc ataatgagac atttcgccgg tttcattcag

49081 aaagggggga ggtagaacga aaaaaaaaaa tgaatatcga ccgttccagt attaaaaatc

49141 gagcgggaaa aatgagaggg ggggagggta tgtatatgtg ggatatctct atccatattg

49201 aattgcagat acatcaatga tagaatcatt tctgatggga ccaaatacgg gtcttccgat

49261 agagaatatg gacaagaaat caaaataaaa taaataaaat aataaaatag gagtagactt

49321 tttttcgata ttaggaatca gtatctaatg aattcaacgg ttccgacata aataaatgaa

49381 agagggggat gggatcacaa tgagatctcg gtctcataag gggatatggc gaaattggta

49441 gacgctacgg acttggttgg attgagcctt ggtatggaaa cctactaagt gataacttcc

49501 aaattcagag aaaccctgga attaaaaatg ggcaatcctg agccaaatcc tgttttcaga

49561 aaacaagggt tcagaaagcg agaaccaaaa aaaggatagg tgcagagact caaaggaagc

49621 tgttctaacg aatggagttg attaacattg gtataggaat ccttctatcg aaattccaga

49681 aaggatgacc ctatcctata tacgtactga aatatcaaac aattaatcac gatccgattc

49741 tgtatttttt ttatatgaaa aatggaagaa ttcttgtgaa tcgattccaa attgaaggaa

49801 gaatcgaata ttcagtgatc aaatcattca ctcctcggat agatcttttg aagaactgat

49861 taatcggacg agaataaaga tagagtccat tctacatgtc aataccgaca acaatgaaat

49921 ttatagtaag gggaaaatcc gtcgacttta gaaatcgtga gggttcaagt ccctctatcc

49981 ccaataaaaa gaaaagagcc cgttttacta cctaacctct ttatttcgtc atcggttcca

50041 aattagttat gtttcttatt cactctactc tttcacaaac ggatccggac agaaaccttt

50101 ctctcttatc acaagtctat agatacgata tacttacaaa tgaacatata taggcaagga

50161 atttccatta ttaaataatt cacagtccat atcattactc ttacactgac aaagtcttct

50221 ttttgaagat ccaagaaact ccaaggccta ggtaagattt tgtaagactt tttgggtttc

50281 tttaattgac atagacccca gtcctctaat agggcgatgc atccggaatg gtcgggatag

50341 ctcagctggt agagcagagg actgaaaatc ctcgtgtcac cagttcaaat ctggttcctg

50401 acacgcggtt aatgtatcga atggatactc atccaaatga atgggtaaag gaaagaagta

50461 gattttgttc ctttttttta tactgtaccc cctctcgctc aaaaagaatg ttaatacttc

50521 atacatatcc aaagttaggt ggctgaaacc aaaaagtcta gcctagggga gttgaaggat

50581 aggaatagac aggattcatt tcagatacag tacaaagaaa atacgatccc ttttcatttc

50641 tgaatttcat attttcttgc gtattctatt tcctcactcc ctcttacgcg acttccagga

50701 gcccatccaa gtgatatgcg cggtacaaag ttcatggtac agaactcttt tgattcatac

50761 tattggcttt actcatccga aatagatata tttaaaattg gggaatatca acgaagccta

50821 tttattagct catccataat acgaattaga gcccagttac tctgtttcat ctagaacgta

50881 aaaagattcc ttgaatatct ggagtcgtag aagtgaagat tagtttctta tcattcaatg

50941 agcatcttgt atttcataga aattgggggc aatataatcc ttacgtaggg gccatcccac

51001 ccaactttcg ggcatcaaga tacgtttcag gcgtggatga ttttcataag agatccccaa

51061 catatcataa gattcccgtt cttgaaaatc agcacttttc caaatccaga aaacagacgg

51121 gattctagga ttcctccttg gaacaaatac ttttatgcac acctcttcgg gttgatccac

51181 cccatactgt attctcgtaa gatgatacac actagctaaa aatccgccag gtgctacatc

51241 ataggcacac tggaaacgta gataattgta accatataca tatgaaataa cagcaatgga

51301 gtaccaatcc tcgggcttta tttgtaaagt ctctcttcct tggtaatcga agcccaacga

51361 tctatgaact agctcatgct tgactagcca agcagatgaa cgaccctgca tcttcttgat

51421 ctctcccaca tttgtatgaa tattttacat ttacgatgaa ttttatgaaa attgactcgc

51481 cgtttgttat tccgcacaaa aacaccctgc ctaattcatt aatttggggg aagatactga

51541 acttttgtat ttgaaaaatg tttcagaagg tatctctgaa gtagatggag attggtagag

51601 taatccttga tcgtaatttc cagtatgagt actgtgtcca acatgaaact tgtgattggt

51661 agtaaaatat cgattttcct gttgagaccc aattctatct tcatagattt ctcgagagac

51721 tttcttacga agtttcgtta tagcatctat aactgcctct ggtttagggg ggcagcccgg

51781 caaatagaca tccacaggaa ttagcttatc gactccccga acagtactat aagaatcggt

51841 actgaacatc cctcctgtaa tagtacaggc tcccatagca atgacatatt ttggttcggg

51901 catttgttca tataatctca ctaacgaagg agccattttc attgttactg tgccggctgt

51961 taaaattagg tccgcttgcc taggacttga tcttggtacc agtccataac gatcaaaatc

52021 gaatcgcgag cctattaatg aagcaaactc aatgaagcaa caactggtac cataaagaag

52081 cggccataaa ctagagagtc ttgaccaatt cgaaagatca ttcgatgtag ttgaaataac

52141 tgaattttgg gcggttcggt caagtaacgg aaactcaata gaattcataa ctgtttcaat

52201 gtaatctttt ccttcttttt gattttgatt gtctgaatat tcaggagcta agaccattcc

52261 aatgctcctt ttcgccatgc ataaactgaa ccaacaattg ggataagcac gaaaattaaa

52321 gcttctataa acacagatac acccaataca tcgaaactca ttgcccatgg ataaagaaag

52381 actgtttcaa catcaaaaac aacaaaaact agagcaaaca tgtaatagcg gattcggaat

52441 tgtaaccaag catcccccat cggttctatg cccgattcat aactagagag cttctctggt

52501 ccttcactaa tcggggccaa aactccggaa attagaaatg ccaaaatagg aataacactt

52561 gatattatta gaaatgccca gaaaatatca tattcgtgaa gcagaaacat agaagcactc

52621 ctattaatgt ggaatatacc gaattagttg attcaaattg gaattctcaa ttcatccata

52681 actgcattag tcgaaacaac aattttgatc aaaccacata gtttcgtttg tttacttgtt

52741 gtgggtcatg tatcgtctca agattcatcc aacggaatcc cacttacact tacttcgatt

52801 ctatttagat atggtgtaga catataatgc tattatacaa atcaaactct ctcctacctt

52861 gcctcgggtt ttctatcaaa caaaaaaagg aattaaggaa ttttttttaa agaatatttt

52921 aaataatatg aattgaaatt gaaataatat tcaaacaata ttattcaaat aagattaaat

52981 gaaatattaa acataaataa tttcaatatt ctattaatat aatagagcca aagaggaggt

53041 ctggcccatt ttttctctct ctctcttttt tttttttttt cttagtgatt tagaatatag

53101 tcagtagtca gatgtaatag aatttctagg aatttccatc tcgggattta tggtatattc

53161 tacgtggctg tgttggtgta ttcttttcat taagatccgg atagaggatt attgtttcta

53221 ttgatttgat acggatacga aaacggaatg catcgaccga ttcgattctc tctccctgtc

53281 ccagatttat acttaattga tttgattcaa tccattgaat tgtgaggaac ccttacatat

53341 aaaaactcat gggttcctat acccaaatta ggaaactttg gacctgtact accccggccc

53401 cggctttacc cccgagttag aagtcttgaa agaatcattt cagacccatt tctaggacta

53461 aagatcgtga tttggaatga ctcgaaatac ttatttattt aatgtaataa taacacctag

53521 caggaccaac caacgagtta ggtttcgtga caacaaaaaa cgtttctttt gaagcaaacc

53581 tacaaaatgg ggcatagttt aatggtagag tcggctgaat cgtaaatgat ttacagaaga

53641 tacttcgaat ggaatcatgc gttgtcgaac gattcgatag acaaaatctc cccatcccaa

53701 aaccaactca tagaacatag aaatagaaga gggtgcgttg atacatttag gaccaattca

53761 ttgtctctaa aacaatgaat tgggattgtt gttctgccaa aaggcgatac gtcgggggat

53821 tcctaactca tccaagttca aatgggccct aatcttttta gataaagtct gcattggtag

53881 aattggaatg atgaacaaat tggattgggt agattggaat aaaaaaaaga agttattaag

53941 tattgtacag aaaaatgact acttgctttg ctaagccggt tatacgaaga aaagcctatt

54001 gtacaatgaa acttaccaaa gagcttcgtt tttgaaactc tggcttttct acaaatacaa

54061 gaacaagaat aggttctaga taatgtgact tactattaga ttgaatttgg atttgatttg

54121 gttgggtcag gttggagttt ttcttgagcc aggctcatgt tatgattttg acttcataaa

54181 ttggcttggg tataccaaag caaaggtgta tcactaaatc ttggatcatg gacaaataaa

54241 agaggaaaaa ggccgtatgt cattcacaga cgaagattaa tgaagaagaa tgggtttgtt

54301 tatccgagat ttgaaaatac cgatccgatt ggatccattg gaataaatta ttgttttcaa

54361 gccccgaggg atctccgtga tcctgtggga atgattccat ttctatggaa caatcaaccg

54421 gccggtcaca cgcactaatt aggaaatgaa tacaaaaatg tatagggcta tacggactcg

54481 aaccgtagac cttctcggta aaacagatca aacttattat tatcgaaatg attcgaactg

54541 tttcaaagac ccaacatgcg tttttttttt gcattgggct ctttcattaa ctgataaaaa

54601 gatcggctag tccaccatat tttttcttga caggaagata acgagatggc tccatgcgct

54661 cggattcatt atttgaattc tgatccggga gcaataccaa agtgtttcaa agaagggtta

54721 ccctgacgta ggtctgcctc cggcctagat caacctaagt taaatggagt ctctatcaat

54781 ccgccccaag agtcaaatat gatacttaat acaccttaaa gttcatagga cgaaaagagg

54841 ttattttgag gtccttatcc tcattatgcc tagcattgaa gggactgggt attcacctta

54901 tcaatgatca aaccaatgat gggttctatt tggtacctga attggcacct gaatcggacc

54961 gaacaaaata tttgtcaggc tattgttctc ttgttccctc gaatccatgg agtaagacat

55021 cgatttctca ataagatcaa ttctgttgat tgcatgatgg actcctctga aaaagcattg

55081 gcgcgcgtgt aaacgaggtg ctctacctaa ctgagctata gcccttgtca tagacatatt

55141 aacatctaga taatttcttg tcaagatgga tattccataa tcccacatga taactctccg

55201 atccgtttcc tgccaaggat tggtattgct gagaagtaat attccgtcta taatccccga

55261 tgtgatgggt cccatttttt ctttctcttt gtgatgataa atgacctact taacccagtg

55321 gttagagtat tgctttcata cggcgggagt cattggttca aatccaatag taggtagaac

55381 ttattagata ccggagtcga tggtatctaa taagtttttc tacccacctt cttctctttt

55441 ttttttatgg attttgtacc ctttccctat tataccccca ctactcatat ttgtatttgt

55501 tttttttttg ttcgttacat cagattacaa ttgattgtat ccaattggcg gaatccaaat

55561 atggtgtata aacagaactt cttttgatta ttctgataca ttgactagta cgaaataaca

55621 ttgatagcct ctactcgtgt cctagctcgt ctaagagcta gattcgcctc aattgcttgt

55681 ctcttgcctt cagctctact caagttagct tcagctattt caagagttcg ctgagcttct

55741 tgtggatcaa tgtcactacc cttctctgca tcatttacta aaatggtgat ctcattattg

55801 cctattctag cgaaaccgcc catcacagcc atcgttaacc attggtcgtt gaggcgtatt

55861 ctcaaaatac ctatatctac ggctgtggca ataggggcgt gatttggtaa tacgccaatt

55921 tggccactat tagtagataa aatgatttct ttcacttccg aatcccaaat aattcgattc

55981 ggagtcagta cacaaagatt taaggtcatt tcttcaattt gctctccact tctaagttca

56041 tagccttcgc agtagcttca tcaatgttac ctaccaaata aaaggcctgc tcgggaagac

56101 catctaattc tccggaaagg atcagttgaa accccctaat tgtttctgta agaccaacat

56161 atttccctgg agaaccagta aatacttctg ctacgaagaa gggttgtgat aagaaacgtt

56221 caatttttcg tgctcttgct acggttaaac gatcctcttc agataattcg tccaacccaa

56281 ggatagctat aatgtcctga agttctttgt aacgttgtga agtttgctta actctttgcg

56341 cagtttcata atgttcctca ccaacgatcc taggttgtag catagttgac gttgaatcta

56401 acggatctac tgctggatag atacctttgg cagctaatcc tcttgatagt acggtagtag

56461 catctaagtg cgcaaatgtc gtagcaggag cagggtcggt caaatcgtcc gcaggtacat

56521 aaactgcttg aatggaagtt atagacccct ctttggtaga agtaattctt tcttgcaaag

56581 aacccatttc tgtactaagg gtaggttgat aacccacagc ggaaggcatt ctacctaata

56641 aggcggatac ttctgatcct gcttggacga aacggaaaat attgtcgata aatagaagta

56701 cgtcttgttc attaacatcc cgaaaatatt ccgccatggt tagggcagtc aaaccaactc

56761 tcatacgagc tcccggcggt tcattcatct gtccatggac tagagctact ttggattctg

56821 caatattttg ttcattaatc actccggatt ctttcatttc catgtaaaga tcatttcctt

56881 cacgagtacg ttcgcctact ccgccaaata cagatacacc tccatgagct ttggcaatgt

56941 tgttgatcaa ttccatgatg agtactgttt tacccacccc agctcccccg aatagtccga

57001 tttttcctcc acgacgataa ggggctaaaa gatctaccac tttaatccct gtttcaaaga

57061 ttgataattt ggtatctaac tggataaaag caggcgcaga tctatgaata ggagatgttg

57121 tgcgagtatc tacaggacct aaattatcaa caggctctcc aagaacgttg aaaattcgtc

57181 ctagagtagc tccacccact ggaacgctta gaggagctcc cgtgtcaatc acctccattc

57241 ctctcatcag accatctgta gcactcatag ctacagctct aactcgatta tttcctaata

57301 attgctggac ctcacaagtc acattaattt gctgaccgac agtatctcga cccttaacta

57361 ccaaagcgtt gtaaatatta ggcatcttgc cggggggaaa agctacatcc agtaccggac

57421 caatgatttg agcaatacgc cccaggtttt tttcttcaag tgtggaaacc ccaggcccag

57481 aattagtagg attgattctc ataataatga aagtgaaata tgtcaaaatt ttttgcgaat

57541 attaccgaat cgaaaataaa tgtccgatag caagttgatc ggttaattca ataaataaga

57601 aatgggagtt agcgcttgat ttcgttggta ccattcaact gaatccaact caatcgttta

57661 ctcattcact aaatgaattt tcaagttcaa ccaacccttt ttcaaaatat ctatcaaatc

57721 aagtagatga ataagaatca tggggaagtc tttcattttt ctatcattat agacaatccc

57781 atccatatta tctatggaac tcgaacctga actttattta tgattcagaa tttctatctt

57841 attggccgtt gttccttatt tcagcatatt agtttccgcc tattcttgtt tttatttttt

57901 ataccctttc atggatgaat tctgcctatt ttcacatcta ggatttacat atacaacata

57961 tatcactgtc aagggtgaat ttcttattat ttagattcaa aaaaaaagaa ggagatccaa

58021 acttgcaaaa caaggattgg gttgcgccat acatatgaaa gggtatacaa taatgatgta

58081 tttggatgta tttggcaaat caaataccat gataacgaac cattctaatt agttgataat

58141 attagttgag aattttgtga aagattcctg tgaaaggttt cattcattac taatccatgt

58201 cgagtagacc ttgttgttgt gagaattctt aattcatgag ttgtagggag ggacttatgt

58261 caccaaaaac agagactaaa gcaagtgttg gattcaaagc tggtgttaaa gattacaaat

58321 tgacttatta tactcctgac tatgaaacca aagatactga tattttggca gcatttcgag

58381 taactcctca acccggagtt ccacctgagg aagcaggggc tgcggtagct gccgaatctt

58441 ctactggtac atggacaact gtgtggaccg atggacttac cagccttgat cgttacaaag

58501 gacgatgcta ccacatcgag cccgttcctg gggaggaaac tcaatttatt gcctatgtag

58561 cttacccttt agaccttttt gaagaaggtt ctgttacgaa catgtttact tctattgtgg

58621 gtaatgtatt tgggttcaaa gctctacgag ctctacgtct ggaggatctg cgaattcctc

58681 ctgcttattc caaaactttc caaggcccgc cccatggcat ccaagttgag agagataaat

58741 tgaacaagta tggtcgtccc ctattgggat gtactattaa accaaaattg gggttatccg

58801 ccaagaacta cggtagagcg gtttatgaat gtctccgtgg tggacttgat tttaccaagg

58861 atgatgagaa cgtgaactcc caaccattta tgcgttggag agaccgtttc gtattttgtg

58921 ccgaagcaat ttataaagcg caggccgaaa caggtgaaat caaaggacat tacttgaatg

58981 ctactgcagg tacatgcgaa gaaatgatca aaagggccgt atttgccaga gaattgggag

59041 ttcctatcgt aatgcatgac tatttaacgg ggggattcac tgcaaatact accttggctc

59101 attattgccg ggacaacggc ctacttcttc acatccatcg cgcaatgcat gcagttattg

59161 atagacagaa gaatcatggt atgcactttc gcgtactggc taaagcgtta cgtatgtctg

59221 gtggagatca tgttcacgct ggtaccgtag taggtaaact agaaggggaa cgggacatca

59281 ctttgggttt tgttgattta ctacgtgatg attttattga aaaagaccga agtcgcggta

59341 tttatttcac tcaagattgg gtctctatgc caggtgttct gcccgtggct tcagggggta

59401 ttcacgtttg gcatatgcct gccctgaccg agatctttgg ggatgattcc gtactacagt

59461 tcggtggagg aactttagga cacccttggg gaaacgcacc tggtgcagta gctaatcggg

59521 tggctgtaga agcgtgtgta caagctcgta atgagggacg tgatcttgct cgtgaaggta

59581 atgaaattat ccgtgaagct gccaaatgga gccctgagct agctgccgct tgtgaggtat

59641 ggaaggagat caaattcgaa ttcgccgcaa tggatacctt gtaatccagt aattcccgtt

59701 cgttccccta attgtaatta aactcggccc aatcttttac taaaaggatt gagccgaatt

59761 aaagaatgag gatcctatgt atatggatag atatagatct tgtatctatc aatatgtgcc

59821 tactttacct agatatacaa gatctaaata caagataaga tctaagacta aacaactcaa

59881 tgcttctatt gttggatcca taattaatcc tatggatcct taggattggt ggatcctttt

59941 ctatcccgtt gtttcggacc atagatcgag ccaagggtca caacttcttc tactcatcct

60001 gtatattgtc cttttcattc cgtgttgcat tagaaactta ttattatacg agattatacg

60061 aaaatgaatc cttcctagga gggaacaaat atttctcttt tcgatgagag tttgtacaca

60121 acatgggaga aacctatctt ctatttataa taattgaaga aaaggttcca tcatatcata

60181 tatagtgaat tgatactccc gattcccaca aaatcatttc tttcgttcaa tagttactcg

60241 ttattagtta ataatcctag tgattggatc tatatgcgta ttccgatagg aaatgaaata

60301 gtaaaatgat ttttcgtcga atgactattc atttattgta ttttcaaata gggggcagga

60361 aggatctatg ggaaaatggt ggttcaattc aatgttgtct aacgaggagt tagaacacag

60421 gtgtgggcta ggtaaatcaa tggacagtct tggtcgtcct gttggaaata ccagtggaag

60481 tgaagatccc attctaaatg atacgaataa aaacaatcat aatcatggtt ggcgcgaaag

60541 taatagttgc agtaatgttg atcatttttt cggtgtcaga gacatttgga gtttcatctc

60601 tgatgacact tttttagtta gggatagtaa tggtaacagt tattccgtat attttgatat

60661 tgaaaatcgg gtttttgaga ttgacaatga tagttctttt ctgagtgaac tagaaactgc

60721 tttttctagt tatctgaata gcgggtctaa gagtgacaat cgctactatg atcattatat

60781 gtatgatact acgtatagtt ggaataatca cattaatagt tgcattgata gttatcttcg

60841 ttctgaaatc agtattaata agtacatttc gagtggtagc gacaatccca tttacagtta

60901 tatttatagt tacatttgta gtggtgaaag tgtaagtgat agtgacaggg ggagttctag

60961 tataagaact ggcggtaatg gcagtgattt caatataaga ggaagatcta atgatttcga

61021 tggaaataaa aaatacagac atttatgggt tcaatgcgaa aattgttatg gattaaatta

61081 taagaaattt tttaggtcaa aaatgaatat ttgtgaacaa tgtggatatc atttgaaaat

61141 gggtagttca gatagaatcg aactttcggt tgattcgggc acttgggatc ctatggacga

61201 agacatggtc tctattgacc ccattgaatt tcactcggaa gaggaacctt atagagatcg

61261 tatcaattcg tatcaaagaa agacaggttt aactgaggct gttcaaacag gcataggtca

61321 actaaatggg attcccatag ccattggggt tatggatttt cagttcatgg ggggtagtat

61381 gggatccgta gtaggcgaga aaatcacccg gttgatcgaa tatgctgcta atagatctct

61441 acctgttatt atggtgtgtg cttctggagg agcacgcatg caagaaggaa gtttgagttt

61501 gatgcaaatg gctaaaatat cttccgcttt atatgattat caattcaata aaaagttatt

61561 ctatgtatca atccttacat ctcctacaac cggcggagta acggccagtt ttggtatgtt

61621 gggagatatt attattgctg aacccaatgc ctacattgca tttgcgggga aaagagtaat

61681 tgaacaaaca ttgaataaga cagtacctga cggttcacaa gcagctgagt atttattcca

61741 taagggctta tttgatccaa tcgtaccacg taatccttta aaaggtgttc tgagtgaatt

61801 atttcagcta cacggtttct ttcccttgaa tcaaaattca agtagagcgc taggctcagt

61861 tatttgtagc gaactttagt tcatcggaat caaagtcaaa ataagaagag tggagttttc

61921 tttggtaaca taacttctat aggaagtttc ggataattac tttttttgat gcagattttt

61981 tatcctaccc ctattcatga ttagtaatca ggaaccccct atcaggagga aaagagtgaa

62041 ttcttccttc cgcggaatgg aattgggaaa aaaaatcaaa agaatttcat gttcccttct

62101 ttcatattaa tatatatcgt attaatatat atagaataat tcaaatctat aagggaagtg

62161 ttgcatattt ttatatctcc cgaggaccta cacttttgac tatgaattcc tgttggatcg

62221 gattctaaca aatccttagg aaactcgtaa gaaactcttt attagaagat aagggcggta

62281 gaacaagaat aataaagcgg attatcatcc atctatttct tgtagaaagg tgaatagata

62341 cacttattta gctctacatt ccttgcactt attatatact taataatact tataatagat

62401 atacttatca taagataaga tatctttata acaggtacaa atattaaatc gaggcaccca

62461 ttctatgaca gatttcaatt taccctctat ttttgtgcct ttagtgggct tagtgtttcc

62521 agcaattgca atggcttctt tatctcttca tgttcaaaaa aacaagattg tttagacctg

62581 ataggacaaa atttcatcaa tttatttcaa cacttggact tggatcataa tagggatatc

62641 catttagtgg aatatgatac gacatgtggc cccctccggg cacaaataaa aaagtgatta

62701 tacatgcgga tacattatat atggataaat gcatgtatat ggggggatag ctgttttaaa

62761 atggatcaga gcggatatct gaaatagaaa gttaacgtat ctatattatg tagatataca

62821 tagtggtggt attatacgaa ggggatgtta ttattttata tctaaccaat tcgatgaatt

62881 actcctaata gttcgcgtca taatagtgct agttgatgag agttacttcg ggagcaaaat

62941 aaaaaaagta aaatcaaatt catttggctt attctcttct ctcaattcca ataggatgca

63001 actgaatcta gtatgaacta tcgatcagaa cgtatatgga tagaacttat aacggggtct

63061 cgaaaaacca gtaatttctg ctgggcctgt atcctttttt taggttcact aggattctta

63121 ttggttggaa cttccagtta tcttggtagg aatctgatat ctttattccc gtctcagcaa

63181 atcatttttt ttccccaagg aatcgtgatg tctttctatg ggatcgcagg tctgttcatt

63241 agttcctatt tgtggtgcac aatttcgtgg aatgtaggta gcggttatga tcgattcgat

63301 agaaaagaag gaatagtgtg tatttttcgt tggggatttc ctggaataaa tcgtcgcatc

63361 ttccttcgat tccttatgag agagattcaa tcgatcagaa tggaagttaa agagggtctt

63421 tatcctcgac gtgtccttta tatggaaatc agaggccagg gcgccattcc cttgacccgt

63481 actgacgaaa atttgactcc acgagaaatt gaacaaaaag ctgctgaatt ggcctatttc

63541 ttgcgcgtgc caattgaagt attttgaaat gaagggaatg aatgctttct cagcatgagg

63601 gaagggaccc aggaaccccc ttttaaatat aactgaagct tcttcggaac gttcattcga

63661 gcaaaacatg ttagattcta tttcccccgt tccgttggta atccttctgt ggcccataga

63721 ataaagcagg cggacgtata cggaacaacc ataataagaa gcaattttga tcgaccaaaa

63781 ctcctttttc tgcatataga actcaattct actagtaaca agtctaataa gtatgtattc

63841 atcacacata tcagagcatt tcggaataca taatttatct ttttaggacc aatactttgg

63901 attaatacat tagatacaga tgtatcatat cccgttaatt atctttcttt tgtcaatcga

63961 tgtttctttt ttgatccttt ctttagctcc tggataacca aacgtttaga tctctcataa

64021 ctatccaatt tctctctcgt tttgtcacct atttcggatt tcatcattaa tatttttcag

64081 aaatatcccc tcaattattc ccgggtcgtg ggtagccagt gaaaatttcg aaaaaataat

64141 tgaggggagt tctttcgtct cgaaatcaaa ataatattca ttatttcaag cggttctttt

64201 gggcattcat cgaaagaaca aatgaagata gaattggttc gaatttgacc aactgagata

64261 tctgggaaaa gtatttgatt atttcttcat tcgaaacggg cccttattct atttctattt

64321 ctatattctt tctagatcca aggactaaac aattcaaaaa aaaaaggaat agatccatag

64381 gttccatacc ttgttataga actcatgctt catagaaata tcggatcaga tagagtcggc

64441 gaatgaagcg ggttcattaa caattcacag atgaaaagtg tcaaaaaaga aagcattgac

64501 tcccctcccg tatcttgcat ctatagtctt tttgccctgg tggatctctc tctcatttaa

64561 taaaagtctg gaaccttggg ttactaattg gtggaatacc ggacaatccg aaactttttt

64621 gaatgatatt caagaaaaga acgttctaga aagattcgta gaattagaac aactattcct

64681 gttggatgaa atgataaaag agtacccgga gacacagata caaaagcttc gtataggaat

64741 ccacaaagag acgatgcaat tggtcaaaat gcacaacgaa gatcatatcc atatcatttt

64801 ggatttctcg acaaatataa tctgttttgc tattctaagt ggttattcta ttctgggtaa

64861 tgaagaactt gtcattctga attcttgggt tcaggaattc ctctataact taagcgacac

64921 aataaaggct ttttctattc ttttattaac tgatttatgt atcggattcc actcaccccg

64981 ggggtgggaa ctaatgattg gttcggtcta caaagatttt ggatttgctc ataacgatca

65041 aattatatct ggtcttgttt ccacttttcc agtcattcta gatacaattt tgaaatattg

65101 gatcttccat tatttaaatc gtgtatctcc ttcacttgta gtgatttatc attcaatgaa

65161 tgaatgaaga actcatttga tctgctgata tcaatcaaat catgatgcta cttcgtacat

65221 aaacaaactg ttttgaagct tactcactct ttatacttct acccgcccag ggggttccta

65281 ctatacttca gtacaattat tccagtacaa tggcagaatc atggataggg aactatgcta

65341 gctacctacc taatttattg tagaaatttc cgggatcaat tattggacca tgcaaaatag

65401 aaataccttt tcctgggtaa agaaagagat gactcgattc atttccgtat tgatcatgat

65461 atatgtaata actcggacat ctatttcaaa tgcatatcct atttttgcgc agcagggtta

65521 tgaaaatcca cgagaagcaa ccggtcgtat tgtatgtgcc aattgccatt tagctaataa

65581 gcccgtggat attgaggttc cacaagctgt gcttcctgat actgtatttg aagcagttgt

65641 tagaatccct tatgatatgc aaatgaaaca agttcttgct aatggtaaaa agggggcttt

65701 gaatgtgggg gctgtcctta ttttacccga gggattcgaa ttagctcccc ccgatcgtat

65761 ttctcccgag ctgaaagaaa agatgggcaa tctgtctttt cagagctatc gccccactaa

65821 aagaaatatt cttgtagtgg gtcctgttcc tggtcagaaa tatagtgaaa tcgtctttcc

65881 cattctttct ccggaccctt ctactaagaa agacgttcac ttcttaaaat atcccatata

65941 cgtaggcggg aacaggggaa ggggtcagat ttatcccgac gggagcaaaa gtaacaatac

66001 agtctataat gctacagcag caggtatagt aagcagaata gtacgtaaag aaaaaggggg

66061 atatgaaata agcatagccg atgcatcgga tggacaccaa gtggttgata ttatacctcc

66121 aggaccagaa cttcttgttt cagagggtga atccatcaag cttgatcagc cattaacgag

66181 taatcccaat gtgggtggat ttggtcaggg agatgcagaa atagtacttc aagatccatt

66241 acgtgtccaa ggtttgttgt tcttcttggc atctgttatc ctagcacaaa tctttttggt

66301 tctcaaaaag aaacagtttg agaaggttca attgtccgaa atgaatttct agatccacgg

66361 attcatcaag ttgataaaaa gggccgaatt attgttgatc aatgcaatta tgtatgatcc

66421 aaaaaaatat ggaaagcccc ttgtcttgct ttgtttatcc tgcttttctg cgagatgccg

66481 ggaattgctt gtatcccatt cccagtaata gtatgtatat tgcgaagaag actacttgac

66541 cccccccctt tttatttttt ttttttcaat ccaaattgga gcggtgtgac gcttcttatt

66601 gccagattgt aaatgccatg gaatgcatca atagtttttt ctatctaata gaatcgaatt

66661 ctaatagaca ataggcggca taacattaag taggaagaga atacgcggca agggataaat

66721 gaaagaatga ttctgggagg gattacttgt cttcctaatt ttcgacacaa gaaaaggaat

66781 tttccgccct tttcttgtgt cgaaataata atgattcttg atcttgttcg tcaaagatta

66841 ctgttttctt ttccaggtct atcggaacct ctttctttag attcataaga agtggcggac

66901 aaacaaaaaa gggggatggc ttagtaaaca aatagaactt cttcaacgaa cttataaaat

66961 ttcaagtaaa aaaaaataaa attttaagat gagataataa ataaggattt ggatatgtgc

67021 aaaaatccaa gattttcccc tttcaaccgg aacattaaga gtcttttttt acttgatgaa

67081 attttatttt tatagaattt ttatagaata gagtagagta aggttcaatt aaattagtat

67141 agaaatggtt tgcaggatgt ctcatctgta gaaatcctgt gtcatccaaa aaatcaattg

67201 attccttctt tcttcttgtt tcggaagggg ccctcatact atggcggaac agatactatg

67261 aatcaatcaa gggattccat ttttcaaaaa catcatcaga aacaaagcat ccttttatca

67321 tttctatgaa tctaatatta tgattatgtt gactggatga atttccaact ttttttatgt

67381 tatggaatag atcaaacaaa accttacccg aagagtaaga actcaatagg accttacccc

67441 tctttgtctg attcgggggg taaggtccta ttgagttctt actttttcat gtctacaatc

67501 cggctcatcc gattactata gggatgatcc caatccggaa tatgagccgt aaaagaaaat

67561 acctattgaa ccgatcacag gaataccagt tacagtacct ataagccaaa gaggaatcct

67621 tccagtagta tcggccattt acccacttcc ctccacattt catcaagtgg tcgtgctaga

67681 gacataaaca gtcatggata attatgagga tgatatcctt ccgaatggga taagagaatt

67741 cctactcttt ttctttattt tttctcaatt gaagaaataa ttggaaaata aaacagcaag

67801 tacaaaaatg agtaataacc cccagtagag actggtacga ttcaattcaa cattttgttc

67861 gttcgggttt gattgtgtcg tagctctata attcggatta ggtttatcgt tggatgaact

67921 gcattgctga tattgacccc aaaaaagaaa cagtaggtac agctagtccg tgaacagcca

67981 accatcgcac tgtaaaaatt ggataggttc gatctatggt cattgaggcc tcctaaaagg

68041 atctactaaa ttcatcgagt tgtgccaaag gatcaaaacg accagttatt aatggaattc

68101 cttgccggct ctctgtgaaa tattcgtttg gccgagggct tccaaacaca tcgtaagcta

68161 aacctgtgct gacgaataac caacccgcaa tgaataggga aggtatagta atactatgaa

68221 tgacccagta tcgaatactg gtaataatat cagcaaaaga acgttctccc gtgcttccag

68281 acatgctgag ctccacatat tcttgtacag tcaaacagag gatcgattcc gtgaaagatg

68341 ggatcagtaa atggaaaact actgatattt catccttgtg agatcgtcaa tagtgtaccg

68401 aaggtgtatt tagagtatac cgaatcagta tagctatcct tcctctgaca cagcaatgca

68461 ttttcaatca gtatcgaaaa gaaatggaat tcctttcttc ctttcttgtt ccttgtctat

68521 gcaaaaccgc gtgtcattca atagaaaatt cttaaatacc tgtaatatag ggtttccttt

68581 actggcttcg gaatagaaac tgaagatctt ggtaaagtat gagtcgacgg gttctaataa

68641 ttcatgatta ttatatttcc acaattcaat tagatgcaaa atttagaaac cccttttcat

68701 ggttgtcgaa aaggtatttt tttttttcta atcctttcat ttaaagtaat tggttggtcg

68761 tacagtagta gacagtagta ggtagtagta gatggaaaaa acagaacaaa cagtagttgg

68821 aacaattatc aatatttgtg atgcaaccat tgctgcatta gacccaaagg ttccttctta

68881 acccagctac aaggatggga ctgaacttta tgatatggaa agacagagtg cgaaacctaa

68941 actaaaagga taatagcaat tgctagtttt agaatgaagt tgggctcgaa ataaaggttt

69001 tatttcttcg agaaatcatg ggatactttt catttttctt ctcgttcgaa atattatgtg

69061 caattaacca acctactact gaatacaatt aagttaaaaa gtcaagtaaa agccgttatc

69121 cggctgtttg ttccaaaatg gattagataa attgaaaaag aaacgaaatg atcaaaaaaa

69181 tggaattttc aaatccaatt ctttatattc tgatagttac tcaaagagaa tttcattttt

69241 gaattgaatg aagttacaag acacagttct tattattagt actttactca cgggttgctc

69301 actgaatctg ttgattcgga atcatgggat ctgtagatgt tacaggcgac gaatccatct

69361 tttttttcta cccctcttac tctctctttg ttagtgccgt ctataatgga tgatgaatca

69421 agagctttca attggaactg attctgtcaa ttggtatttt ttcttgtcat tgtatctcgc

69481 aaaaatggaa acttaggtaa gtgctttaga accctatgta tgaaaaagaa gatatctcat

69541 ttagctcctc catgactact ataactagtt atttcggttt tctactggct gcttcaacta

69601 taaccccagc tctattgatt agtctgagca agatacgact tatttgaaat taattgaatg

69661 aacaattcat aaaaatgaat atttctgtga gattcccggt attctatagt tccttcccgc

69721 gttaattgcc aattcttggt tattgagatt catgggcgat tcggattaat atttagggac

69781 agatattacc tctctttttc tattctttca aagaaattga aatgattgaa gtttttctat

69841 ttggaattgt gttaggtcta attcccatta ctttggccgg attattcgta actgcatatt

69901 tacaatacag acgcggcgat cagttggacc tttaattgag taacatctct tttttttaat

69961 tgacctcctc cttaatctcc aggaggtcaa attcaggttg cagttcaagt tagtgaagtt

70021 attttattgt gattcaacat taaaagaaca gaatcacgct ctgtaggatt tgaacctacg

70081 acatcgggtt ttggagaccc acgttctacc gaactgaact aagagcgctt tattatgatg

70141 ggagatacgg atgtcaagaa aaggattctt tttgtacccc caatacatct tgtatgtata

70201 gtatcataaa atggtagatt gtgtccaatt ttaatcgatc tcaattgacc cctcgttact

70261 gtccatagga gaagtgataa gtagggatga caggatttga acccgtgaca ttttgtaccc

70321 aaaacaaacg cgctaccaag ctgcgctaca tccctttcat ttgttgtaca gtgccattgt

70381 agggaatcca tgttttgttt tccacatcat aatttcctct atctaaatag aatttctttt

70441 gccatttctt ctttttggtt ttttggtttc attctcataa agaattatat acatacctaa

70501 cgtataaacg tataaaggaa tgaatattta tcagtagtgc tcaggaagga gggttcatct

70561 ttttctgttt tagggacagg tagatttcat ctaccggatc gttgtatata tccattttgg

70621 ttagagattc cccgtacaaa tgatctttaa ctacatatgc atctgatcat atatgtatta

70681 caatatacaa taaagtcaat aaagttaact ttaaagaagg aggattttca atgcgagata

70741 taaaaacata tctctccacg gcacctgtgc taactactct atggttcggg tctttggcgg

70801 gtctattgat agagatcaat cgtttattcc cagatgcgtt gacattcccc tttttttcat

70861 tctagttatt gacatgggaa gggatcaaga agattagaga tacaataaat tctctgtgac

70921 taaccccccc ctttttcagt tctttaggat aggaaagaaa gagtaaagaa taaaagtgga

70981 ttgaatctca tcgaaactcg ggttcgggtt aatataggga gaacagaaat ggaaatgtgg

71041 gtcgagggca ggctgttcaa gatcatacaa gatactaaat gaaatactgg gattgggaat

71101 aattgatagt tagaaatatt tgtattactt aataatttga ttactctatt gattgcaacg

71161 aaatctttca taattgaatt ggatttcgag ttagcaactt ctcgtctatt tatttttcat

71221 tcctttcttc ttcgcttcgg ttcgaatcga aaatagaaga attgagtgaa ttcaaaatcc

71281 aaaggaggtt catggctaag ggtaaagatg tcagagtagt agttattttg gaatgtacca

71341 gttgtgtccg aaatggtttg aataaagaat cgcggggcat ttccagatat attactcaaa

71401 agaatcgaca caatacacct agtcaattgg acttgaaaaa attctgccct tattgttaca

71461 aacatacgat tcatggggag ataaagaaat aaatcgaacg gaacgcgtgt gccactcttc

71521 caaggaagag gaagaaatta catatatata tataatatat acaaatccag tcctattttg

71581 gtcggatccg agatgaatga agaaatagga ttttagaaat aagaaataaa ccatggataa

71641 atccaagcgg ccctttcata aatccaagcg atcttttcat aggcgtttgc ccccaattgg

71701 atcgggggat cgaattgatt atagaaacat gagtttaatt aatcaattta ttagtgaaca

71761 aggaaaaata ttatctagac gagtgaatag attaaccttg aaacaacaac gattaattac

71821 tattgctata aaacaagctc gtattttatc ttcgttacct tttcttaata atgagaaaca

71881 gtttgagaga accgggtcga tccctagaac tactggtcct agaaccagaa ataaataagc

71941 ctattcctct caatcgaatc aaaactctaa ttcgaactca gattgaagtt ttgttcgaaa

72001 aagccgagag attgtcgcgc cgtaatgtaa taataaaaaa aagaatgggg gaagaataaa

72061 tctttttttt ttattgaaac gtgttcgttc attcctacta cttatcttat catacgaatt

72121 tctactatac cctcccggag ttcattctcc ggggaactcc gtttaaagta ttccagtgaa

72181 ttccttccaa tctccttatt tgatgatcgc attggaaatc gtgtcaagac aattcctatt

72241 tgatatggct atttgtgcag gtattttacg attaagaagc aactgcctct tgtacagatc

72301 aagtattaat cgactataac tatgggatcc cctattctca cgagttactg catttatccg

72361 agtgatccac aaacgacgaa aacttctctt tcgcctgcct ctatcccgat gagtggaaac

72421 caaagctctc attttctgtt gagtagtagt tcgagtaagt cttgaatgag cccctcgaaa

72481 ggttgctgca aataaacgaa tttttgttct acgtctccga gctatatatc ttcgtctaac

72541 tctggtcatt gaatcaaaag aaactttgat gaataactaa ttgatttctt ttctttcagt

72601 cattcttttc ccctctcctg gtttattaat aacaaaacgg attcttccga tgtataaaat

72661 taaaatacaa attccaatgg cttttgctac tataaccttc ccaaccacga tttttttatt

72721 tcttccaggc atttcacctc aaaaaaaaaa aaaagaaatt gtaccgatat taggtataaa

72781 ataaatcgta aatggacaaa tagtggcttc catcgtttct atggttactt cttaaacggc

72841 gaggtcctct ctatacaccg gagccccttt cctcatttaa tcaatgttat tggtaacttg

72901 tacagttcac gctctttggc tctaccgatg aattatcgag taataggtct tttttcaatg

72961 ggatctatcc atacagtgac ggcatttaat tatgaaggtt gaataggtag ctgaccctgt

73021 tagtccgttc ttgcaagagt aggagcataa tctttctgct tcttaaatat cattcccccg

73081 cttaatggat aaccatttgc taccaatggg aattgctttt catctcaaat cgaggtgatt

73141 ggatttgcac caatggaaac cataaattcc atacaaatag aggtatacga gagatcttta

73201 tttttcgata gtgaatggag ttcttccatt ctattcattg gtacgagtca ttgatactgt

73261 aaaagtcgtc tcatttgttc tagctcatga tctgaacgag tcgcacatac accctagtac

73321 atgttcctcg acgctgagga catcctcgaa gagcggggga tttcgtgaca tttctgattg

73381 gctgtcttgt atttctaata agttgtttaa tagttggcat gctgaatcat atacagaatg

73441 ggctggttta gatcgatcct aaccggatga ttatgaatta cttctttacc caggtaagaa

73501 gataaaagat caaataaggg ttcattcaaa ttatgattcg aaatggaatc aaagatttat

73561 gcgaaatccc cggtattttc gatcgctaca agatcaacaa tgccataatc ttgggcttct

73621 gttgctgaca taaaaacatc cctttccatg tcttcggata caacccataa aggattgccc

73681 gttctttgta cataaaccct tgtgagggtt tcgcggagtt tcagtagttc ttccgcttcc

73741 aggataaatt ctcctgtggg tgcctcataa aaagaactag caggttgatg gatcataacc

73801 ctgatgatat aacataatga acgattcctc tatctcgcat gattgggcga agggaaggga

73861 taaagaataa caagcaggga gaaaaagata gaattgaaca accgtacagg catcttttgt

73921 gcatacggct ctgtaatgga attttttttt ctcttttttc atcgaagaaa gagacaagtc

73981 gaatctatca gacccagatc gttgaatgat ccatttacca tccttccttt cggagtaatc

74041 aaaaaatact atgatggttc cgttgcttta tatatttatc tcgtctgtga ttcagcaatc

74101 ccaaagtttc tttctgatcc gatcaaataa aaataagtaa aaaatgatct tttttttttt

74161 tttattttca cactctttca taacataaat attggaaaga gacttctgat gtggaagcaa

74221 aaaggtttgt gacgctgaaa tggaccccga tacataagat caagtcggaa ataacctttc

74281 tttcatacta ctatctcgat acataatctc atattatgaa aaaataataa tagtttgctc

74341 atatcgaact tgaaatgcca tgctattatt acttaatatt attattattt tattcatatt

74401 ccatatgacg aaggcatagt ctttttttct ctcaaataaa aaaactcatt ggcgccaagc

74461 gtgagggaat gctagacgtt tggtaatttc tcctccaacc aggatgaaag atcccattga

74521 agcggctaat cccatgcata ttgtatgcac atctggtggc acaaattgca tagtatcata

74581 aatagctatt cctgggatta cccatccgcc gggagaattt ataaacaaat acagatccct

74641 ggtatcatcc tctatactga gatataccat gagaccaaca agttgattcg agatctcgct

74701 atcaacttct tggcctaaaa aaagtaatct ttctcgatga agtcggttga ttaggacaaa

74761 attctattcc ttaggaaccg tacacgcacc tttgggtgca tacggttcaa aaaatcaaaa

74821 ttgagaaaaa aaaaaagaaa ttgtcgattc cagccctatt tcttttttcg tagcgggctt

74881 tttcttccat tttttaagaa acatgagttt tgacttgctt ccctataaaa tcaaaaaaga

74941 attcactgaa cttatcgagc taacccctca ttgatgtatt gtttcatcga gatctaaatc

75001 acgatgtaat tttcttgttc ccgaatgggc ctcttccact cttttaggtt tatgctctac

75061 tccgggtaaa gatctgcccg aattcgattt gcacatatag gacaaatgat cccagtacca

75121 cttctttttg ctatgacttc tttttttttt tttcaatttg tttcattttc atgccttcca

75181 caaaatattc gatgtattca tcatattatt ccattaattg gcaatttggg atcactcata

75241 tggtataaag gaatcatttc tgatagggtg gtaatcatac atggattacc ttggtatttt

75301 ctgaacggag cctgtatact tcattttatt ggtccaagcc aaccataaat tcttttaatt

75361 gagaatattg atcctccaac caaataaatt gatctaattg cacttcacgc ttcgaattat

75421 tgatggttca atcaatcttt cttgggcgaa acagaggata tctcgatcgg gggagagaac

75481 ggggaaatcc catatgaccc aatatgtctg acaagtcaca ctatacgtca acccaaactg

75541 catcttcctc tccaggactc cgaaaaggta cttttggaac accaatgggc attaaatgaa

75601 agaaaaatga agtattctat ttcactttga tgtggaaacg taacaaacaa tggtttattg

75661 tcttcataat attgtcgttt atcgtatttt atcgatagat tggaagattc atagaggaag

75721 acggaataaa ggaaaattct tacgaacgga tcgttcgaat gagaaacaag tatctataca

75781 ttcgctcaca aaaaatagga ttaatccccc cattgcgtat tggtacttat tgggtataga

75841 atagatctgc ttctctttgt tcctacgaac agaattgttc aattattact aacggaacag

75901 aataaatatt aacccttgtt tcgagataat ccaatgaaaa ggtgaggtcc atagcatagt

75961 tatttccaat gtgataaagt tacatagtat ctattttatc tttgagaaag gggtatttcc

76021 atgggtttgc cttggtatcg tgttcatacc gtcgtattga atgatcccgg tcggctgctt

76081 tctgtccata taatgcatac agctctagtt tccggttggg ccggttcgat ggctctatac

76141 gaattagcag tttttgatcc ttctgacccc gttcttgatc caatgtggag acagggtatg

76201 ttcgttatac ccttcatgac tcgtttagga ataaacaatt catggggcgg ttggagtatt

76261 acaggaggaa ctataacgaa tccgggtatt tggagttacg aaggtgtggc cggggcacat

76321 attgtgtttt ctggcttgtg cttcttagca gctatctggc attgggtgta ttgggaccta

76381 gaaatattct gtgatgaacg tacgggaaaa ccctccttgg atttgcccaa gatttttgga

76441 attcatttat ttctctcagg ggtggcttgc tttgggtttg gcgcatttca tgtaacaggc

76501 ttgtatggtc ctggaatatg ggtatccgat ccttatggcc taaccggaaa agtacaatct

76561 gtaaatccag cgtggggtgc ggaaggtttt gatccctttg ttccgggagg aatagcctct

76621 catcatattg cagcaggtac attgggtata ttagcaggtc tattccatct tagtgtccgc

76681 ccaccccaac gtctatacaa aggattacgt atgggcaata ttgaaactgt cctttccagt

76741 agtatcgctg ctgtcttttt tgcagctttc gttgttgctg gaactatgtg gtatggttca

76801 gcaactaccc cgatcgaatt atttggtccc actcgttatc agtgggatca gggatacttc

76861 cagcaagaaa tatatcgaag agttggcgcc agtctagccg aaaatctgag tttatcggaa

76921 gcttggtcta aaattcccga aaaattagct ttttatgatt acatcggtaa taatccggcg

76981 aaaggtggat tattccgggc aggctcaatg gacaacgggg atgggatagc tgttggatgg

77041 ttaggacacc ctatctttag agataaagaa gggcatgaac tttttgtacg ccgtatgcct

77101 actttttttg aaacatttcc agtagttttg gtggacggag acggaattgt gagagccgat

77161 gttcctttta gaagggcaga atcgaagtat agtgtcgaac aagtgggtgt aactgttgag

77221 ttctatggtg gcgaactcaa tggagtcagc tatagcgatc ctgctactgt gaaaaaatat

77281 gctagacgtg cccaattggg tgaaattttt gaattagatc gtgctacttt gaaatccgat

77341 ggtgtttttc ggagcagtcc aaggggttgg ttcacttttg gacatgctac gtttgctttg

77401 ctcttctttt tcggacacat ttggcatggc gctcgaacct tgttcagaga tgtttttgct

77461 gggattgacc cagatttgga tgctcaagtg gaatttggag cattccaaaa acttggagat

77521 ccaactacaa ggagacaggt agtctgatac aacattgctc cggtatcttt cgcctctata

77581 tttgattttt ttgatttgac ataaggtacc gtagaaatat tgatttgaat catcgccttt

77641 ctttgctctt gtcctttctt tatctgggaa ataatcctaa atgaacaggt gtggaagcta

77701 taattgtaaa caacgatcga atctatggaa gcattggttt atacattcct cttagtctcg

77761 actttaggga taatcttttt cgctatattt tttcgagacc cgcctaaggt cccgactaaa

77821 aagacgaaat gatttttcat tatcttaatt gaagtaatga gtcccccata tgggggactc

77881 attacttcaa ttagtctccg tgttcctcga atggatctct tagttgttga gagggttgcc

77941 caaaagcggt atataaggcg taccctgtaa agcttacaag tgaaccagat atggagatgg

78001 cgactaaggt tgctgtttcc attattagag aatttcaaga ccacgatgga tctatgctac

78061 gataagatcg tttatttaca acggaatagt atacaaagtc aacagatctc aaccaatgca

78121 atagtattta tggctacaca aaccgttgag ggtagtgcta gatctgggcc aagacgaact

78181 attacagggg atttattgaa accattgaat tcagaatatg gtaaagtggc tcctggatgg

78241 ggaaccaccc catttatggg tgtcgcaatg gctctatttg cgatattcct atctattatt

78301 ttagagattt ataattcttc cgttttactg gatggaattt caatgaatta ggtccataag

78361 aaccagaagc cctagctttt caatcaaaaa tgaatcactt aggactcaga tttatagtcc

78421 attctggtag tttgaccgtg gaattccgtt gtttcggtat ttccggaata tgagtgtgcg

78481 acttgttata attgatccta ttgatagtac agagaatggg tctgtcatct cgacagagat

78541 ggttctgcct cgtcggatat tcatcctagt atctggagca cggaatatat ggaatagatc

78601 aagaaatatt tgaactatga ttcataccta ctattcagac ctcgtgactg gacttccaaa

78661 aattttcaaa caaagaggta tttgataaat tgaacgattt ttcttccttt agaatcatgc

78721 ttattttgac cgaaggacaa atctttctct ggatttttag tcattacatc tatgaataag

78781 tgatgatcaa atagttctta ctcatagaac ccttggtctt agtttttggg ttttattgaa

78841 tcatcgtggt tctagtatga atctgaggtt tcaatcgatt cataggctct caacaagaga

78901 attcctatca aaaaaaaata gtaaacaata gtcaatctgc attacgcaca aacaaaaaca

78961 acaaatcaaa taacaaataa ataggggaat agaagattca agaggcctgt aacgatcaac

79021 ataaagacag atgagctaac ttgatatttt ggcattctca tcacaacaaa gaagagagtt

79081 cggattttgg ttccttcgta tcttcagaga cgattgaatc aagtggataa ataagaaatt

79141 tcaaattttc tattacatat ccattgtaat cagtatttgg gtgtttctgc ttgagccgta

79201 cgagatgaaa ttctcatata cggttctcag agggggagtc cccttggttt acctatatga

79261 gtaaagtata tgattggttc gaggagcgtc tcgagattca ggcgattgca gatgatataa

79321 ctagtaaata tgttcctcct catgtcaata tattttattg tctaggaggg atcacactta

79381 cttgtttttt agtacaagta gctacgggtt ttgctatgac tttttactat cgtccgaccg

79441 ttacggaggc ttttgcctct gttcaataca taatgactga agccaacttt ggttggttaa

79501 tccgatcagt tcatcgatgg tcagcaagta tgatggtcct aatgatgatc ctacacgtat

79561 ttcgtgtgta tctcacgggc ggatttaaaa aacctcgcga attgacttgg gttacgggtg

79621 tggttctggc tgtattgact gcatcgtttg gtgtaactgg ttattcctta ccccgggacc

79681 aaatcggtta ttgggcagta aaaatcgtga caggcgtacc tgaagctatt cccgtaatag

79741 gatcaccttt agtagagtta ttgcgtggaa gtgctagtgt gggtcaatct actttgaccc

79801 gtttttatag tttacacact tttgtattac ctcttcttac tgccgtattt atgttaatgc

79861 attttccaat gatacgtaag caaggtattt caggtccttt atagagaaga cagatcatag

79921 atatttgtaa tcgatcatat ataatttcgg ggaggaacaa tagtgtttta ttgctacaaa

79981 tatggattat tgaaaagaat aagacatctt tttggatatt tctcttcaac taactacgaa

80041 gtattgtatt ctttatttga tacgaatagt tgaagtacat tctccgaaga gaagatggat

80101 tatgggagtg tgtgacttga actattgatt gggccgtgca gatatatgat tttatccgcc

80161 acattggaat tcacaaccaa atgtgtctct gttccaacca ccgcgtaggt ccccctacag

80221 aggataggct ggttcgcttt aggagaatct tttctatgat cagaccaaat catgttatgt

80281 tgtgcatgaa cgggctccgt aagatccaat agaataaaat aaaatgaagt aatgtggcat

80341 gatccagatt atgttttatc tatttactta aagtatggaa atgcattcat ttcctctgca

80401 tcgatcccaa tctatgatac tatcggagtg aaacaaggga tctaaggaag aacataggct

80461 agactttatt agtaacaagg aaatcctttg tattaagaag actcgagata ttgtggggat

80521 aaacactaat cacaaagcat gagaccatcc aaaaagcatt tgatcatgat caaatttgga

80581 agcctacttg ggtattgagc atttacttgt aagaactgaa ttccttgcaa tgggtagttg

80641 caaccccgta aaattgaatc cggtaaatct tttcttacat agagtcatat atgtgtggat

80701 gatatatcta ttttatatgg acccgtttta ttcttttgat tcttgctcga gccggatgat

80761 aaaaaattat catgtccggt tccttcgggg gatggatcta taagaaagaa ttcacctatc

80821 ccaataacaa agaaacctga cttgaatgat cctgtattaa gagctaaatt ggctaaaggg

80881 atggggcata attattacgg agaacccgca tggcccaatg atcttttata tatttttcca

80941 gtagtcattc taggcactat tgcgtgtaac gtaggtctag cggttctaga accgtcaatg

81001 attggtgaac cagcggatcc atttgcaact cctttggaaa tattacccga atggtacttc

81061 tttcccgtat ttcaaatcct ccgtacagta cccaataagt tattgggtgt tctcttaatg

81121 gttttagtac caacgggatt attgacagta ccgtttttgg agaatgttaa taaattccaa

81181 aatccatttc gtcgtccagt agctacaaca gtttttttga tcggtaccgc ggcagccctt

81241 tggttaggta ttggggcaac attacctatt gataaatctc taactttagg ccttttttaa

81301 gttgatttaa ccgtgaaata ctacgcgtat gtatctaggg aatagtcact tctaaagtga

81361 attctcccta gatacatctc ttaaatttca ttatcaatcc attctggata tagagatgat

81421 actaaggatt caaaagccat tttcttcttt tctttctttc aaaaaagatg aaataatacc

81481 aatggattta aaacttattc ttaggtaaat aaattgcaaa atgcttctgt agaatgtcca

81541 atatctgttt tacatcttct atgcgaagat gttcaattct cataagatct tcttgactgt

81601 tattcaaaag gtccaataat gtatgtatat tggacctttt gagacaatta taggtcctgg

81661 agggcaattc tgattggtca ataaaaatac atttcagtgc aattcctttt ttgtttttcc

81721 ttatattagc caatctatca tgaaaagtaa aaaacgatac agtaaacctg ttttgattgt

81781 cttctaaata aaaatgaatg tcctcttcct ccgcatgtag aaaaggaata aataagtcaa

81841 tcaaagtacg ggaagcttcg cgaagtgctt ctttaggagt taaacttcca ttcgtccata

81901 tttcaagaaa aagtatctct tgtttctcat tctcactccc ataagaatga atactatgat

81961 tcgcatttcg aacaggcatg gatacagcat ctataggata acttccatct tgatagttat

82021 tgggggattt catacgatat ccgcgatccc tctcgatttt gaattcaata cacaaatgaa

82081 ttggctccgt cagattagct atatgctgtg tagtatcgac tagttccaca gaaggcggtg

82141 agatgatatc ttgagcagtt acgtatttag gacccctgac gcaaatggat gcgtcacgag

82201 ttccatacag attacttctc aatacaattt ctttcaaatt cattaaaatt tcatgtactg

82261 attcttcaat accaactatc gtagaatatt catgtgatac cttctcagat tttgcacgtg

82321 tgatacatgt tccctctatt tctccgagta aagcctttcg catcgcgata cctatcgtat

82381 ctgcttgacc tttcataagc ggggacagaa cgaaacggcc ataataaaga cgcttactgt

82441 ctgttcttga ttcaacacac ttccactgta gtgttcgagt ggatactgct acttcttctc

82501 gaaccatact aatattattg tttgatcaga tcattgaatc atttatttct attgcaatcc

82561 attccatttt tatttctaca cacgtctttt tttaggaggc ctacatccat tatgtggcat

82621 aggggttacg tcacgtacga aacttaatag tataccactt ctacgaatgg ctcgtaatgc

82681 tgcatctctt ccgagaccag ggccttttat catgacttct gctcgttgca gaccctgatc

82741 cactgcctta cgaatagcat ttcctgctgc ggtttgagca gcaaatggtg tccctcttct

82801 tgtgcctctg aatccacaag taccagcgga ggaccaagaa accacccgac ctattacatc

82861 tgtaacagtc acaatggtat tgttgaaact cgcttgaaca tgaataactc ctttttgtat

82921 tctacgtcca ttcttacgtg aaccaattct tggtatagct tttgtcatat tttatcatct

82981 cataaatatg agtcagagat atacggatat atccatttca tgtcaaaaca gatcctttat

83041 ttgtacatcg gaccgtttag aaagtccctt gttagaaaga ttacccctgt ctctgtttat

83101 gtttcggatt ggaacaaatt actataattc gtccccgcct acggatcagt cgacattttt

83161 cacaaatttt acgaatggaa gcccttattt tcatatttgt tattccttaa ttccaaatat

83221 actccttgga agaaaataag tctcttcaaa ttttgaacct cgaattgtat tcccatgaaa

83281 ggaatgttta aattcaaata aaaagccgcc taatcattcg actctttgtt gcgaagtcta

83341 taaattatac gtcccctagt tgaatcataa cgacttactt cgattttgac tctatctcct

83401 ggtagtatcc ggataaaact ccgtcggatc ctccccgaaa cataacctag aatcagatct

83461 tcattgtcta aacgaactcg gaacatacca ttgggaagtg attcagtaat taaaccttcg

83521 tgaatcaatt tttgttcttt cattccaggt aacccccttg aagtatcaac taatggagga

83581 ggagtaatag tagacaattc gtctttcctc tctttttccc aaatagcaag ttacggatca

83641 aattcggata ccagaaggat caccagatat aatacaaaat ttctccccca attctttcta

83701 gtcgagcttc tcgatctgtc attatacctc gagaagtaga aagaattacg acccccattc

83761 cacctaaaat cctaggaatt cgttgatggt tggaatagat tcgtagaccg ggacgactga

83821 tacgctttaa aatatttcta tatgttcctt tcctattcct tctatgtcgc agggttgaaa

83881 ccaagaaata tttgttgttt tccctatgtt tcctaacatt ttcaataaac ccttcttgta

83941 gaagtatttt aacaatgttt tcggcgatat tagtagatgc tactcgaacc gttccttttt

84001 tatccatgtt agcatttctt atagaagtta ttatatcggc aatagtgtcc ctacccatga

84061 cgaactaaat ttatgggtgc cttccagttt tgatataatc aacatgttcc tttttttttt

84121 ttcatttttt cttatttatt tatgaattat taaaggtata tgcgtgagac acaatctact

84181 aacgtgatct atttcagaga cctgactata ctctatcacg gtctcatcta ctagtattta

84241 taagacttca ggagctaatg agactatttt agtgaaattc aactgtctca attcccgcgc

84301 gatcgctcca aaaactcgag ttccttttgg atttccttct tgatcaatga caactgctgc

84361 attgtcatca tatcgtatta tcataccgtt gtcgcgtttg agttctttac atgtacgtac

84421 aattacagct ctgatcactt ctgatctttc gagaggcata ttgggcactg cttctttgat

84481 tacagcaaca ataacgtcac caatatgagc atatcgttga ttactagctc ctatgattcg

84541 aatacacatc aattctcgag ccccgctgtt gtccgctaca ttcaaatgag tctgaggttg

84601 aatcatatca tttttttttt tttgaatctg ctctttcaat gcaaaaggca aaggaaaaag

84661 agagaaatat tgtctgccca gaaatccaaa aatctgcgat tgtatttttc atcacaaata

84721 cccttcacat acctatcacg cgataatgaa ttgagttcgt ataggcattt tgcacgcagc

84781 tattgaaata gctgctctgg ctacagtttc tgatactccg cccatttcat aaagtattcg

84841 accgggttta acgacagata cccaatattc gggagatcct ttccccgaac ccatacgtgt

84901 ttcggtaggt cttactgtaa cgggtttgtc gggaaatata cgtacccata tttttccacc

84961 acggcgtgca tatcgtgtca ttgctcgtcg tcctgcttct atttgtctag atgtgatcca

85021 agcgggttca agtgcctgaa gagcgtatct gccgaaacaa atatgattgc ctcgataaga

85081 tattcccttc attcttcctc tatgttgttt acggaatctg gttcttttgg gactaagcat

85141 gatggttgtt tctcaatccc atctctactg cagaaccgga catgagagtt tcttctcatc

85201 cagctcctcg cgaatgaaac gattcaataa gattacgtat atgtatttat tgaatgaata

85261 atacactgaa tcatggaatt tcttgatatt taatctgtca cacgggaagc cgtatagtat

85321 atagtatata cggctagacg gatatttcta ttttatttat atgggataat gcctttcttt

85381 tgaaaatgaa tccttgacct ttaccgaatc tgtcaaaata ctacaatcca ataatggttt

85441 cgcgggcgaa tattgactct ttccatattt gcttcattcg tagggtgaac ccatgaccta

85501 tcagaagaaa ttaattggtt cctggttgat tccgccatcc cacccaatga atcattagga

85561 ttcgttttca atagaatctt ccgcagtcac aggtttcgtc gttcccatag cttttccatt

85621 aatggctagg cctgaactat gcaatggagc tcctaattaa attcgttccc gagccaatct

85681 cctcagtctc tattgactcg gggctcttta ttatttgtat ttttcttatg aaccgtattc

85741 atctaattat ggacgaatca gtattgatgc tttatcacac tgccttttat gatatgatgt

85801 gattgataga ccatacatat tggaatcata tatcatggag attctccttc tctctttctc

85861 tcgcccttcc agttacccac atccctctat ttttctttcc aacctataaa tggatttttc

85921 ctttatggaa aaaaaaagat ttcagttgct acaactatat gatcgataca tcatatggcg

85981 actgcttcct tggatctcga taatacaaag caatgagttg gttactagtt cttatagtta

86041 ttagttaggg gctggtctgt tttttgaatc ccaactttaa ataaaaaacc aacgagtcac

86101 acactaagca tagcagttcc accaaaaggt caatcgaatt tttattcaac cttatagaat

86161 tagaattgct catttttcat tttttttttt attgaagtga aaaggaatag tttgtagttt

86221 ttgttctatc actgaataga atggcaagca aaggaagggt ccattattgc tcgtctacaa

86281 atatccaaat tttgatgccc aatgccccat aggcagttcg aactgtatag gaacaatgat

86341 caattttagc tcgaatggtt tggaggggaa ccctaccttc tctgatccat tcgacacgtg

86401 caatttcttt tccgtcgata cgccctgcaa tttccacttg aattcctttt gtgtctgctt

86461 gttcagttaa ttcaatagct tttttcattg cctttcgaaa cgaaacccta ttctttaatt

86521 gtagagctat atattctgca agaatattag gttgtccata aggttttgca actcttgtaa

86581 tagcaatgtt aagtctccga ttcacagaat gaagcccctt ttgtacattg atctgcaatt

86641 cttcgattcc tcgtgtttgg ccttctatta acaaatttgg gaatccaata tagattatga

86701 cctggatcaa gtccattttt ttttgaatct ctatatgtgc aattccaatt ccttcgaaac

86761 ttgaagatac tcttatattt ttttgtacat atatcttgat ccaatcccgt attttttcat

86821 cttcctggag acctatgtaa taactttttg gttgtgcgaa ccaaagggaa cgatgacttt

86881 ggttttcgcc aaggcggaaa ccaagtggat ttattttttg acccatcttt tttttctctc

86941 tctctctcct tctctatata tctttctatt ataggatctc tccatacatt ttttgtttct

87001 aaaaatatat cctgatctgt tttcttttta gagctatcct tcaatacaat aattatatga

87061 caggcgggtc tttctatcgg ataactacgt cctctagccc tgggttttaa ctttttcacg

87121 atagtacccc cattgacttc ggctttacta atgaccgaat cagcttcgtt gaaactttta

87181 ttgtgactag catttgctgc tgcagaataa accagtttaa aaatgggata aaatgctcga

87241 taaggcatga gttccagtaa cataagtgtt ttctcatagg aatgcccacg aatctgatca

87301 atgactcttc gtgctttgtg agcagacata catatacgtt gagctaaagc ttgtacttgt

87361 gtactcgagc tcctcttcat cttctgcttt ttcaaggtct tcttccactt tctccacttt

87421 gacataagat aaggttcgcc tcccgccaat gaacgatgag cacctatttc actttatttt

87481 attaacggag agatctagta tcgtttctcg cgtgtccctg gaaagtaaga gtaggtgcaa

87541 attctcctaa tttttgaccc accatacgat ccgttatata aataggtaaa tgcgcctttc

87601 cattatgaat ggcaattgta ttgccgatca ttgtgggtat aatggtagat gcccgggacc

87661 aagttactat tatctctttt tcctctctca tgttaagctt ctttattttt tccaataaat

87721 gattagctac aaaaggattt ttttttagtg aacgtgtcac ggcctattat tccccccccc

87781 tttttttttt gaaaagacga gtaaaaaaca atatttattt gattttgaat attcctattt

87841 acggcgacga ataataaaac tattactata tttattcctt ttcctacttt ttcttccaag

87901 cgcaggataa ccccaagggg ttgtgggttt ttttctacca atcggggccc tcccttcacc

87961 acccccgtgg ggatggtcta cagggttcat aactacccct cttactacag gacgcctacc

88021 tagccaacat ttagatccgg ctctacccaa acttttctgg ttcgccccaa cattacccac

88081 ttgtccgact gttgctgagc agtttttgga tatcaaacgg acctccccag atggaaatct

88141 taatgtgacc gatttaccct cttttgcaat cagtttcgct acagcacctg ctgctctagt

88201 taattgtcca ccctttccaa gtgtgatttc tatgttatgt acggccgtgc ctaagggcat

88261 atgggttgaa gtagattttt ctttttgatc aataaaaacc ccttcccaaa ccgtacaagc

88321 ttcttccaaa gcatacggct ttccggatgt atatatatat tctatgatga tatctagaca

88381 gatggatttt atatgaatcg tgtgatgaag taccacatga gtggatatat aggaatacaa

88441 atctgccaaa tcactcatgt tatgatctta tacatcctag gtctctccgt ttcgtcatct

88501 ggcttatgtt cttcatgtag cattcagacc gaatgactct atgaaattac gtcgctactt

88561 ccacatatta cgggtaacgt aggagacatc tctatttttc cccgggggaa tttttagaat

88621 taccaccact tagctttcaa ttcacctctg accatcaaat gaaatgtgaa taacccatcc

88681 tcttctcttt gaaacaaggg tcgcttccgc ttctgtccgt gcttcaaaca attttgtctt

88741 ctccatatta ccatatctgg agtgtcaata gttttctatg aggaactact gaactcaatc

88801 acttgctgcc gttactcttc agttttctgt tgaggtctat cccgtagagg tactcaaatt

88861 ggatcagtga tcaatttcta ggtttcgtcg taaacctaat tggttacttc caattacgta

88921 aatccatagt tcaaaccgca ctcaaaggta gggcatttcc cattgatata ggaacttctg

88981 taccggaaac aatggtatct ccaattatag cccctctggg atgtaaaata tatcttttct

89041 caccatctcc atagtgtatg agacaaatgt atgcatttcg attagggtca tattctatgg

89101 ttacgatcct agcagatatg tctttttcat tccgtcgaaa atcgatttta cggtatagac

89161 gcttatggcc tcctcctcta tgccctgcgg taatgattcc cctggaatta cgacctttac

89221 cgcagcgatg ctgtccatag atcaaattat ttcgcggatt ggatttcgct tgactgtcta

89281 tggatccttt gcgtatgctc ggggtagaag ttttgtataa atgtatcgcc gtgttattta

89341 gtattttttt tatttttttt ttacttaaat tcttttctct tctctataag aggtaaaata

89401 gaataacccg gttgaagcgt aatgatcata cgtctgtaat gcattgtatg tcccataatg

89461 ggtcccgttc ttctaccctt tcccgggagt tgatgactat ttatagctat tactttgacg

89521 ccaaagaaga gttcgaccca atgctttatt tctgtcctag ttgatcctga ttcgactttc

89581 ttccccacga gttccagtat cgataagaat tctagttctt actcttcata tgttatggtt

89641 ggtatgaata taccatacca attcgttatg tatggatgat gagattccat tgatacggag

89701 ccagtggaat tagtcttatt gaatgtcccc gttggcctgc atccagcagg aattgaacct

89761 acgaattcgc caattatgag ttgggtgctt taaccattca gccatggatg cttcactggg

89821 gtcattgtac atcgcgagtg acccaaattc aattcactta gattcttttg gattctttag

89881 gaggaatcaa tgaaatgaga ggacatcaat tcaaatcctg gatcttcgaa ttgagagaaa

89941 tcaagaattc tcacgatttc ttggattcat ggatccaacc cgattcggtg aaatctttca

90001 cttccttttt tttccaccaa gagcgcttta tgaaactttt tgattcccga atttggagtg

90061 tcctaatttc acgtgattca cagggttcaa ttcgtcgaca ttgcatgatc aaaggtgtag

90121 tactgcttgt acttgtagta gcggtcctta tatacaatcg aaatagggtc gaaagaaaaa

90181 atatctattt gatggggctt cttcctaaac ctctgcgttc cattggaccc cccaattata

90241 cattgaaaga atccttttgg tcttccaatc tcaataggtt gattgtttcg ctcctgtatc

90301 ttccaaaagg gaaaaatatc tatgagagtt gtttcatgga tccgaaagaa agtacttggg

90361 ttcttccaat aactaaaaag tgtatcatgt ctgaatctaa ctggggttcg cagcgatgga

90421 ggaatgcgat cgtaaaaaag aggaattcca gctgtaagat atcgaatgaa attgcagctg

90481 gaattgagat ctcattcaaa gagaaagata tcaaatatct ggagtttttt tttgtatcct

90541 atacgaatga tccgatccgc aaggaccatg attggaaatt atttgaccgc ctttctccga

90601 gtaagaagcg aaacataatc aacttgaatt cgggacagct attcgaaatt ttagtgaaac

90661 atttgatttg ttatctcatg tctgcttttc gtgaaaaaag accaattgat gaggggggtt

90721 tcttcaaaca acaaggagct gaggcgacta ttcaatcaaa cgagattgaa catgtttccc

90781 atctcctctc gagaaacaag gggggtattt ttttgaaaaa ttgcgctcaa tttcatatgt

90841 ggcaattccg ccaagatctc ttcgttattg gggggaagaa tcggcacaaa tcggattttt

90901 tgaggaacgt ctcgagagag aatttgattt ggttagacaa tgcgtggttg gtaaacagga

90961 atcgggtttt tagcaaggta cggaatgtat cgtcaaatat tcaatatgat tccataagat

91021 ccattttctt tcaagtaacg gattctagcc aatcgaaagg attttctgat caatccatag

91081 atcctttcaa ttccattagt aatgagggtt cggaatatca cacattgatc aatcaaacgg

91141 agattcagca actaaaaaaa agatcaattc ttttagatac ttcctttctt caaacggaac

91201 gaacagagat aaaatcagat cgattctcaa aatacctttc cggatattcc tcaatggctc

91261 ggctattccc ggaacgtgag aagcagatga ataatcatct gcttccagaa gaaatagaag

91321 aatttcttgg gaatcctaca agatcaattc gttctttttt ctctgataga tggtcagaac

91381 ttcatctggg tttgaatcct accgagaggt cgactataga tcagaaattg ttgaagaaac

91441 aacaaggtgt ttcttttgtc ccttcgaggc gatcggaaaa taaagaaata gttgatatat

91501 tcaagataat tacgtattta caaaatacct cctcagttca ttcgattgca gcagatccgg

91561 gatgggatat ggttccgaag gatgaaccgg atatggacag ttccaataag atttcattct

91621 tgaacgaaaa tgcatttttt gatttatttc atctattcca tgatcggaac aaggggggat

91681 acaggttgca ccacgagttt gaattagaag agacatttca agaaatggca gatctattca

91741 ctctatcaat aaccgagccg ggtttagcct atcataataa ggaatttggc ttgtctattg

91801 attcctacgg aaaattattg aatgaggtat tcaactccgg ggatgagtcg aaaaagaaat

91861 ctttattggt tctaccttcc attttttatg atttattttt attggttcta tcttctattt

91921 tttatgattt atttttattg gttctacttt ctatttttta tgatttattt ttattggttc

91981 tactttctat tttttatgaa gagaatgaat ctttttatcg aaagataaaa aaaaaatcgg

92041 tccggatctc ctgcgggaat gatttggaag atccaaaacc aaaaatagcg gtatttgctc

92101 acaacaacat aatggaggcg atccatcaat atagattgat ccgaaatcag attcaaatcc

92161 aatatagtac ctatgggtac ataagaaatg tattgaatcg attcttttta atgaatcgac

92221 ccgattgcaa cttcgcatat ggaattcaaa agcatcccat aggaattcaa aagcacccaa

92281 taggaaatga tattctgaat catctaacta taataataga taagatcaac caacatttat

92341 ccaatttgaa aaagattaag aagaagtggt tcgatcctct tatttctcga accgagagat

92401 ccacgaatct ggatcctaat gtatatagat acaaatgttc caatggaagc aagaatttcc

92461 aggaacattt ggagcatttc gtttctgagc agaaacaccg ttttcaagta atgttcgatc

92521 gattacgtat taatcaatat tcgattgatt ggtccgaggt tatcgacaaa caagatttgt

92581 ccaagtcact tcgtttcttt ttgtccaagt cacttctcct tttgtccaag tcgcttctct

92641 ttttatctaa gtcacttcct tttttcgttg tgagtctcgg gaatatctcc attcataggg

92701 ccgaaatcca catctatgaa ttgaaaggtc tgaatgatca acccggcaat cagttgttag

92761 aatcaatagg tgttcaaatc gtttatttga ataaattgaa acccttctta ttgtatgatc

92821 atgatacttc ccaaagatcg aaatttttaa tcaatacagg aacaatatta ccttttttgt

92881 tcaacaagat acaaaagtgc atgattgact cattccgtac tagaaaaaat cgcaagaaat

92941 cctttgagaa cacggattcc tatttctcaa tgatatccca cgatcgaaac aattggttga

93001 atcctcagaa aagttcattg atatcttctt tttatagagc aaacagactt caattcttga

93061 atcatcccca ttgcttctgg ttctattgta acaaaggatt ccatttttat ggggaaaaga

93121 cccgtatcca taattatgat tttacatatg cacaattccc caatatcttg tgcattcgca

93181 acaaaaaatt ttctttgtgt ttcggtaaaa aaaaacatgt tttgggagag agagagacta

93241 tttcaccaat tgagtcacag gtatctggca tattcatacc taacaatgtt tcacaaagtg

93301 gtaacaaaac gtataacttg tacaaatctt tccatttttc aattggatct gatccatccg

93361 ttcctattta ctcgattgca gacatttcgg gaacacctgt aatagaggaa caaatagtca

93421 attttgaaag aacttattgt cagcttcttt cagatatgaa tctatctgat tcagaaggga

93481 aaaacttgca tcactatctc cgtttcaatt caaacatggg tttgattcac actccatgtt

93541 ttgagaaata tgtgccgtcc ggaaagagga aagaactgag tctatgtcta aagaaaaacg

93601 ttgagaaggg ggaagtaggt agaacccttc aacgagatag tgctttttca aatctctcaa

93661 aatggaattt gttccaaacc tatatgccat ggttccttac ttggacgggg tgtaaatatc

93721 tttatttcac cttaaaaaac aatatttatt tgatattgaa tattcccttt caatattccc

93781 taagtggcag tcaaaatttt gtgtccgttt ttcatgatat gatgcatgga tcagatatat

93841 catggccaat tcctcagaaa aagtggtggt cgattcttcc acaacggaat ctgataagtg

93901 agagttcgag taagtgttta cagaatcttc ttctgtccga agaaatgatt catcgaaata

93961 atgagtcacc cattccattg atatggacac atctgagatc accaaatgct tgggagttcc

94021 tctattcaat tcttttcctt cttcttgttg ctggatatct cgttcgtaca catcttctct

94081 ttgttttccg agcctctagt gagttacaga cagagttaga aaagatcaaa tctttgatga

94141 ttccatcata catgattgag ttgcgaaaac ttctggatag gtatcctaca tctgaactga

94201 attctttctg gttaaagaat ctctttctag ttgctctgga acaattagga gattctctgg

94261 aagaaatacg ggattctgct tctggcggca acatgctatt gggtggtggt cccgcttatg

94321 gggtcaaatc aatacgttct aagaagaaat atttgaatat caatctcatc gatctcatca

94381 gtatcatacc aaatcccatc aatcgaatca ctttttcgag aaatacgaga catctaagtc

94441 gtacaagtaa agagatctat tcattgataa gaaaaagaaa aaacgtgaac ggtgattgga

94501 ttgatgataa aatagaatcc tgggtcgcga acagtgattc gattgatgat gaagaaagag

94561 aattcttggt tcagttctcc accttaacga cggaaaaaag gattgatcaa attctattga

94621 gtctgactca tagtgatcgt ttatcaaaga atgactctgg ttatcaaatg attgaacaac

94681 cgggatccat ttacttacga tacttagttg acattcataa aaagtatcta atgaattatg

94741 agttcaatag atcctgttta gcagaaagac ggatattcct tgctcattat cagacaatca

94801 cttattcaca aacctcgtgt ggggctaata gttctcattt cccatctcat ggaaaaccct

94861 tttcgctccg cttagcccta tccccttcta ggggtatttt agtgataggt tctataggaa

94921 ctggacgatc ctatttggtc aaatacctag cgacaaactc ctatgttcct ttcattacgg

94981 tatttccgaa caagttcctg gatgacaagc ctaaaggtta tcttattgac gatatcgata

95041 ttgatgatag tgacgatatc gatattgatg atagtgacga tattgatgat gaccttgata

95101 cggagctgct aactatgacg aatgtgctaa ctatgtatat gacgccgaaa atagaccgat

95161 ttgataccac ccttcaatta gaattagcaa aagcaatgtc cccttgcata atatggattc

95221 caaacattca tgatctgtat gtgaatgagt cgaattactt atccctcggt ctattagtga

95281 actatctctc cagagatagt gaaagatgtt ccactagaaa tattcttgtt attgcttcga

95341 ctcatattcc ccaaaaagtg gatcccactc taatagctcc gaataaatta aagaaatgca

95401 tgaagatacg aaggcttctt attccacaac aacgaaagca ctttttcatt ctttcatata

95461 ctaggggatt taacttggaa aagaaaatgt tccatactaa cagtaacaga ttcgggtcca

95521 taaccatggg ttccaatgca cgagatcttg tagcacttac caatgaggcc ctatcaatta

95581 gtattacaca gaagaaatca attatagaca ctaatacaat tagatcagct cttcatagac

95641 aaacttggga tttgcgatcc caggtaagat cggttcagga tcatgggatc cttttctatc

95701 agataggaag ggctgttgca caaaatgtac ttctaagtaa ttgccccata gatcctatat

95761 ctatctatat gaagaagaaa tcatgtaagg aaggggattc ttatttgtac aaatggtact

95821 tcgaacttgg aacgagcatg aagaaattaa cgatacttct ttatcttttg agttgttctg

95881 ccggatcggt cgctcaagat ctttggtctc cacccggacc cgatgaaaaa aattggatca

95941 cttcttatgg attcgttgag aatgattctg atctagttca tggcctatta gaagtcgaag

96001 gcgctctgtt gggatcctca cggacagaaa aagattgcag tcagtttgat aatgatcgag

96061 tgacattgct tcttcggtcc gaaccaagga atcagttaga tatgatgcaa aacggatctt

96121 gttctatcgt tgatcagaga tttctatatg aaaaatacga atcggagttt gaagaagggg

96181 aaagagaagg agccctcgac ccgcaacaga tagaggagga tttattcaat cacatagttt

96241 gggctcctag aatatggcgc ccttgtggca atctatttga ttgtatcgaa aggaccaatg

96301 aattgggatt tccctattgg gccaggtcat ttcggggcaa gcggatcatt tatcataaag

96361 aggatgagct tcaagagaat gattcggagt tcttgcagag tggaaccatg cagtaccaga

96421 cacgagatag atcttccaaa gaacaaggct tttttcgaat aagccaattc atttgggacc

96481 ctgcagatcc attctttttc ctattcaaag atcagccctt tgtctctgtg ttttcacgcc

96541 gagaattctt tgcagatgaa gagatgtcaa aagggcttat tacttcccaa acaaatcctc

96601 ctacatctat atataaacgc tggttcatca agaatacgca agaaaagcac ttcgaattgt

96661 tgattcatcg ccagagatgg cttcgaacca atagttcatt atctaatgga tctttccgtt

96721 ctaatactcc atccgagagt tatcagtatt tatcaaatct gttcctatct aacggaacgc

96781 tattggatca aatgacaaag gcattgttga gaaagagatg gcttttcccg gatgaaatga

96841 aacatttgat tcatgtaaca ggctaaaacg gactatgtac tttatctgtt gggttacggg

96901 cgggcatttt accagaggtt tctattgtat caatttaccc ttgtgtgatt cctgttgaag

96961 catatactcg gggggtgggt gcagggcgga cgatttcaaa acggactcct cattcattag

97021 atagagaaga tcgccaagat ttcgtgatcc gctgccgaac ctattccaat tccaacagcc

97081 cggactcgga tcgtggggat cgatggaata cttcgtatca acagatactt ggtatatgta

97141 tatcaatatt gattagatcc gagatctgtt attgaattgc tcattcaatg agcatttcaa

97201 tattatgcct tgaagaggac tcgaacctcc acgctcttta gcacgagatt ttgagtctcg

97261 cgtgtctacc atttcaccat caaggcatct tgaaagtgaa tcgtattcca tgaatatgat

97321 atctatctag tgtgatatat ggaatatatg acaaaggtgg agttttggag tatttctatc

97381 gatcggtcag gtcatatagg cccgagtcgg acatcaaatt gcttcgattt gaattatccg

97441 gaggatacct tatatatatc aaaaagatgt acaatcaaac ctatttctcg attcaatcga

97501 agcccaaaga agttaatatg gtacccaaat aacgatagat atgtaaaaag caggtccgat

97561 tacgcctatt cctaatccta aatggaatgt aacgacgtag ggatccatat gtaaacatag

97621 tctctattta catatgctcg aatgacccct tctcataatg agaatgtaca taaccctatt

97681 ccggtctggt ccggtatgga atgaacttat aatctgatga tcgagtcgat tccatgatta

97741 taagttcata accccagccc attcccattt tgggcggaac agatctacta attcttttat

97801 tccagttagt aaaagggatc ttgaactaag aaatagaccc tagaagctaa aagagggtat

97861 cctgagcaat tgcaataatt ggattcattg atattcctgg tatagtagat gctatcacac

97921 atacaatcat actcaattcg atggaattgt ttgatcttaa gggggatctt ctataatttc

97981 gcacgtgagg ggttatttct tggtttcgtc cagtcattaa taacttgatt atttttagat

98041 aatagtagat agaaacaacg ctcgtaagga gtcctattga aaccaagaaa tataggcctg

98101 cctgccatcc acaccagaat agatggagtt ttccgaaaaa acctgctagt ggaggaagac

98161 ctcctaggga taagagacat agggctgaag agagagccaa aaaaggatct ttcgtgtata

98221 atcctgcata atctcgaatg ttatcagttc cggtacgtag accaaatgag acaatgcgag

98281 caaaagttcc tagattcatg gagatataga acagcatata agttatcatg cttgcatatc

98341 catcatttga gtctccaaca attattccaa taattacata tccgatttga cctatggacg

98401 aatatgcaag catacgtttc atgcttgttt gagtaatagc aatgagattc cccaatatca

98461 tgctaagaat agctaggatt tccagaagaa gatgccattc gtttgatgag aaataaaaag

98521 gaatatcgaa aattcgagtg gctgaagctg aagcagctac tttcgaagta acagaaagaa

98581 aagcaacgac tggagtggga gagtcagagt cgaaaagagg attcctcact tctttctctc

98641 attcaaaacc gtgcatgaga ctttcatctc gcacggctcc taagtgataa aagaaagaag

98701 aactcacctt ctttcttttt tgattacctt cctcgcgtat gtataagacc gaatccattc

98761 gatttctaaa aaggattact aatccttaac ttttcgagga atccttcatc agtggttgtg

98821 aatgactgac tttttcaatc ttttcgaccc cggttccgta ggagcacaag tcagaaagat

98881 tgagaaatag aaccatctga tttgattcgt tctcaatagc catgagatga tcatcttagg

98941 gtgatccttt tgtcgacgga tgctcctatt acactcgtag tctctgaagg atgagaacca

99001 actatgtagc atctacatcg ataattcaag tattgtatac gtcattagtc cgatcctttg

99061 taggaactac ccgtaataac gaacttgcaa aatggatctg tttatcataa agagattcgt

99121 tgttcctgac cctgcttcac cttaattgtt atttgaacaa aaagatcaca ataaactttt

99181 ggtaaaagtt atgtcttggt ccgagtgggg atagcatttc tcttctgcat gtccatggag

99241 ttttgaaaaa tccaaacatc tcagagatag atatagaggt aggaatttgt cgaacgaacc

99301 gcactccttc gtatacgtca ggagtccatt gatgagaagg ggctggggaa agcttgaacc

99361 caattcctac agtgatggat ataagcgcaa tggaaattcc tggggagtta tacatttgtg

99421 tattgataag accattcact atttcttgaa gctcgatctc tcccccggat gaaccatata

99481 gccaagagaa accatgaacc agaatagaag agcttgcccc acccatgagt aaatatttcg

99541 tagtagcctc attagaccgt acatctctct tggtatatcc agataatagg taggagcata

99601 aactgaaaga ttctggagct acaaagatag ttattaaatc gttagcacca cataaaaaca

99661 ttcctcctag agtagctgtt aatacgaata acaaaaactc tgttatagcc atttctgtac

99721 attcaatgta ctctacggat agaggaatac atagagttga acatagtaaa ataagaaatt

99781 gaaagatttc gttgaaattg ttcgtttgga aatttcccga aaagctaatc ataggttctt

99841 ctctccatcg gaacaatagg gccgttatgc tcattactaa acttgttgaa gagatgaaat

99901 ataaccaagg tatatctttt tgatcagagg ttgaatcgat catcagaaga agaattaggc

99961 caaaaatgag gatacattct gggaaaatga aacttccatg gaagagaagc aaatgaaacg

100021 ctttcataaa aattctcgta gaatcgagaa tgaagttttc attctgtaca tgccagatcc

100081 tgaattagta actgcatcca atctccgaaa aagtcccaat tgtttcgaac tttcgatttt

100141 tggaatggga tatttacgga atccccatga acaggatcaa accttattcc atggtatttc

100201 catgagattc ctccttctta ttcttaagaa agcccccgag agggcttagt tgatccatga

100261 tttatgtttc atctttcttt tccttttcgt ttgtttcgag aaagatatcg atccattcca

100321 attctttctt tttctattga ttcttttccg atcgagatgt atggatccac ggatctatgt

100381 gtctatatag atcctgttca tggattaacg aaaatgtgca aaagctctat ttgcctctgc

100441 cattctatga gtctcttcct ttttgcgtat ggcatcgcca ctccctttgg cagcatccac

100501 taattcggaa cttaatttga aagccatatt tcgacccgga cgttttcggg atgcccctaa

100561 taaccaacga atggcaagtg cttttccttg tgtagattct atttcaatag gaacttgatg

100621 agtcgatcca cctacacgtc ttgctttgac tgctatatcg ggagttactc cacgtattgc

100681 ttgacgtaaa acagatagtg gatttgtttc tgtcttttgt tgaatctttt tcacggcttg

100741 atagataatt tgataagcca atgatttttt tccgtgtttc agaatacggt taaccaacat

100801 gttaactaat cgattacgat aaattggatc ggattttgca gttttttctt ctgcagtacc

100861 tcgacgtgac atgagcgtga aagaggttca agaatccgtt ttctttttat acgggctaaa

100921 aacgaatcac ttattttggc tttttgaccc catattgtag ggtggatctc gaaagatatg

100981 aaagatctcc ctccaagccg tacatacgac tttcatcgaa tacggctttc cacagaattt

101041 gatatgtatc tatgaaatcg agtatggaat tctgtttact cactttaaat tgagtagccg

101101 tttccctcct tttcctgcta ggattggaaa tcctgtattt tacatatcca tacgatcgag

101161 tccttgggtt tccgaaatag tgtaaaaaga agtgcttcga atcattgcta tttgactcgg

101221 acctgttctg aaaaagtcga ggtatttcga attgtttgtt gacacggaca aagtaaggga

101281 aaacctctga aatgatttca atattgaacc ttggacatat aatagttccg aatcgaatct

101341 ctttagaaag aagatctttt gtctcatggt agcctgctcc agtcccctta cgaaactttc

101401 gtgattgggt tagccataca cttcacatgt ttctagcgat tcacatggca tcatccaatg

101461 atacaagtct tggataagaa tctacaacgc actagaacgc ccttgttgac gatcctttac

101521 tccgacagca tctaaggttc ctcgaacaat gtgatatctc acaccgggta aatccttaac

101581 ccttcctcct cttactaaga ctacagaatg ttcttgtgaa ttatggccaa taccaggtat

101641 ataagcagtg atttcaaatc cagaggttaa tcgtactctg gcaactttac gtaaggcaga

101701 gtttggtttt ttgggggtga tagtggaaaa gttgacagat aagtcaccct tactgtcact

101761 ctacagaacc gtacatgaga ttttcacctc atacggctcc tcgttcaatt ctttcgaagt

101821 aattgggtcc ttttcctcgt tcgagaatct cctcccttct tccactccgt cccgaagagt

101881 aactaggacc aattcagtca cgttctcatg ttccaattga acactttcca tttttgatta

101941 tgatcaaagg agaagattat tctttttacc aaaatatgcg gatcaaatca cgatcttata

102001 ataagaacaa gagatctttc tcgatcaatc cctttgccct cattcttcga gaatcagaaa

102061 gatccttttt gagtttgaat ttgttcattt ggaatcttta tttatttttg tattttttat

102121 ttatttattt tctttgattc tttatttcga ttttctttcc ctctcttttc tttttattcc

102181 cttccatcat tccttaagtc ccataggttt gatcctgtag aatctgaccc attttctcat

102241 cgaacgaggg gtacgaaata aatccgattg atttttcgat caaaagtact atgtgaaatc

102301 ttcggttttt tcctcttcct ctatcccata ggtacagcgt ttgaatcaat agagaacctt

102361 ttcttctgta tgaatcgata ttattacatt ccaattcctt cccgatacct cccaaggaaa

102421 atcccgaatt ggatcccaaa ttgacgggtt ggtgtgagct tatccatgcg gttatgcact

102481 cttcgaatag gaatccattt tctgaaagat cctggctttc gtgctttggc gggtcgtccg

102541 agatcctttc gatgacctat gttgtgttga agggatatct atatgatccg atcgattgcg

102601 taaggcccgc ggtagcaacg gagccgggaa agtatacaga aaagacagtt cttttctatt

102661 atattagtat tagttagtga tcccggctcg gtgagtcctt tcttccgtga tgaactgttg

102721 gcaccagtcc tacattttgt ctctgtggac cgaggagaaa ggggggctca gcgggaagag

102781 gattgtacca tgagagaagc aaggaggtca acctgtttca aatatacaaa tggattctgg

102841 caatgcaatg tagttggacc ctcatgtcga tccgaatgaa tcagtctttc cacggaggtc

102901 aatctttgcc tgctaggcaa gaggatagca agttacaaat tctgtctcgg taggacatgt

102961 atttctatta ctattaaatt cagaaatgaa gtagttaatg ttggggttac cattatcctt

103021 tttgtagtga cgaatcttgt atgtgttcct aagaacctaa gaaaaggaat ttgtcccttt

103081 ttcgaggtct caaaagggcg tggaaacaca taagaactct tgaatggaaa ttgaaaagag

103141 atgtagctcc agttccttcg gaaatggtaa gatctttggc gcaagaagaa ggggttgatc

103201 cgtatcatct tgacttggtt ctgcttcctc tcttttttta acaataccga gtcgggttct

103261 tctcctacca gtatcgaata gaacatgctg aacaaaatct tcttcctgta aaacctgctc

103321 gatttagatc gggaaaatcg tacggatttt atgaaaccat gtgctatggc tcgaatccgt

103381 agtcaatcct atttccgata ggagcagttg acaattgaat ccaatttttc cattcttttc

103441 gtatccgtaa tagtgcgaaa agaagtcccg gctccgagtt gttcaggaag agtggcgttg

103501 agtttctcga ccctttgcct taggattagt cagttctatt tctcgatggg ggcagggaag

103561 ggatataact cagcggtaga gtgtcacctt gacgtggtgg aagtcatcag ttcgagcctg

103621 attatcccta aacccaacgc aatgtgagtt tttctatttt gacttgctcc cccgccgtga

103681 tcgaatgaga atggataaga ggctcgtggg attgacgtga gagggtaggg atggctatat

103741 tgctgggagc gaactccagg ctaatatgaa gcgcatggat acaagccttg gaatgaaaga

103801 caattccgaa tcagctttgt ctacgaaccg gaagattggt aagtaatgca actatgaatc

103861 tcatggagag ttcgatcccg gctcaggatg aacgctggcg gcatgcctaa cacatgcaag

103921 tcggacggga agtggtgttt ccagtggcgg acgggtgagt aacgcgtaag aacctgccct

103981 tgggagggga acaacaactg gaaacggctg ctaatacccc gtaggctgag gagcaaaagg

104041 aggaatctgc ccgaggaggg gcttgcgtct gattagctag ttggtgaggc aatagcttac

104101 caaggcgatg atcagtagct ggtccgagag gatgatcagc cacactggga ctgagacacg

104161 gcccagactc ctacgggagg cagcagtggg gaattttccg caatgggcga aagcctgacg

104221 gagcaatgcc gcgtggaggt agaaggccca cgggtcgtga acttcttttc ccggagaaga

104281 agcaatgacg gtatctgagg aataagcatc ggctaactct gtgccagcag ccgcggtaag

104341 acagaggatg caagcgttat ctggaatgat tgggcgtaag gcgtctgtag gtggcttttc

104401 aagtccgccg tccaatccca gggctcaacc ctggacaggc ggtggaaact accaagctgg

104461 agtacggtag gggcagaggg aatttccggt ggagcggtga aatgcgtaga gatcggaaag

104521 aacaccaacg gcgaaagcac cctgctgggc cgacactgac actgagagac gaaagctagg

104581 ggagcgaatg ggattagata ccccagtagt cctagccgta aacgatggat actaggcgct

104641 gtgcgtatcg acccgtgcag tgctgtagct aacgcgttaa gtatcccgcc tggggagtac

104701 gttcgcaaga atgaaactca aaggaattga cgggggcccg cacaagcggt ggagcatgtg

104761 gtttaattcg atgcaaagcg aagaacctta ccagggcttg acatgccgtg aatcctcttg

104821 aaagagaggg gtgccttcgg gaacgcggac acaggtggtg catggctgtc gtcagctcgt

104881 gccgtaaggt gttgggttaa gtcccgcaac gagcgcaacc ctcgtgttta gttgccacca

104941 ttgagtttgg aaccctgaac agaccgccgg tgataagccg gaggaaggtg aggatgacgt

105001 caagtcatca tgccccctat gccctgggcg acacacgtgt tacaatggcc gggacaaagg

105061 gtcgcgatcc cgccagggtg agctaactcc aaaaacccgt cctaagttcg gattgcaggc

105121 tgcaactcgc ctgcatgaag ccggaatcgc tagtaatcgc cggtcagcca tacggcggtg

105181 aattcgttcc cgggccttgt acacaccgcc cgtcacactg tgggagctgg ctatgcccga

105241 agtcgttacc ttaaccgcaa ggagggggat gccgaaggcg gggctagtga ctggagtgaa

105301 gtcgtaacaa ggtagccgta ctggaaggtg cggctggatc acctcctttt cagggagagc

105361 taaagaagcg agctacgtct gagctaagct tggagatgga agtcttcttt cgtttctcga

105421 cggtgaagta agacaagctc atgagcttat tatcctaggt cggaacaagt tgataggatc

105481 ccctttttta cgtccccatg cccctcccgt gtggcgacat gggggcgcaa aaaggaaaga

105541 gagggatggg ctttctctcg cttttggcat agcgggcctc cccgtggggg gcccgcaagg

105601 gctattagct cagtggtaga gcgcgcccct gataattgcg tcgttgtgcc tgggctgtga

105661 gggctctcag ccacatggat agttcaatgt gctcatcagc gcctgacccg gggatgtgga

105721 tcatccaagg cacattagca tggcgtactc ctcctgttcg aaccggagtt tgaaaccaaa

105781 cttctcctca ggaggataga tggggcgatt caggtgagat ccaatgtaga tccaactttc

105841 tattcactcg tgggatccgg gcggtccggg ggggaccacc atggctcctc tcttctcgag

105901 aatccataca tcccttatca gtgtatggac agctatctct cgagcacagg tttaggttcg

105961 gcctcaatgg gaaaatggag cacctaacaa cgcatcttca cagaccaaga actacgagat

106021 cacccctttc attctggggt gacggaggga tcgtaccatt cgagcctttt tttttcatgc

106081 ttttcccgcg gaggtctgga gaaagcagca atcaatagga tttccctaat cctcccttcc

106141 cgaaaggaag aacgtgaaat tctttttcct ttccgcaggg accaggagat tggatctagc

106201 cataagaaga atgcttggta taaataactc acttcttggt cttcgacccc ctcagtcact

106261 acgaacgccc cccgatcagt gcaatgggat gtgtctattt atctatctct tgactcaaaa

106321 tgggagcagg tttgaaaaag gatcttagag tgtctagggt tgggccagga gggtctctta

106381 acgccttctt tttcttctca tcggagttat ttcacaaata cttgccatgg taaggaagaa

106441 ggtgggaaca agcacacttg gagagcgcag tacaacggag agttgtatgc tgcgttcggg

106501 aaggatgaat cgcccccgaa aaagaatcta ttgattctct cccaattggt tggatcgtag

106561 gtgcgatgat ttacttcacg ggcgaggtct ctggttcaag tccaggatgg cccagctgcg

106621 ccagggaaaa gaatcgaaga agcatctgac tccttcatgc atgctccact gggctcgggg

106681 ggatatagct cagttggtag agctccgctc ttgcaattgg gtcgttgcga ttacgggttg

106741 gatgtctaat tgtccaggcg gtaatgatag tatcttgtac ctgaaccggt ggctcacttt

106801 ttctaagtaa tggggaagag gaccgaaaca tgccactgaa agactctact gagacaaaga

106861 tgggctgtca agaacgtaga ggaggtagga tgggcagttg gtcagatcta gtatggatcg

106921 tacatggacg atagttggag tcggcggctc tcctagggtt ccctcatctg gatccctggg

106981 gaagaggatc aagttggccc ttgcgaacag cttgatgcac tatctccctt caaccctttg

107041 agcgaaatgt ggcaaaagga aggaaaatcc atggaccgac cccatcgtct ccaccccgta

107101 ggaactacga gatcacccca aggacgcctt cggcatccag gggtcacaga ccgaccatag

107161 accctgttca ataagtggaa cgcattagct gtccgctctc cggttgggca gtaagggtcg

107221 gagaagggca atcactcatt cttaaaacca gcattcttaa gaccaaagag tcgggcggaa

107281 aaaggggaag agctccccgt tcctggttct cgtgtagctg gatcctccgg aaccacaaga

107341 atccttagaa tgggattcca actcagcacc ctttgagatt ttgagaagag ttgctctttg

107401 gagagcacag tacgatgaaa gttgtaagct gtgttcgggg gggagttatt gtctatcgtt

107461 ggcctctatg atagaatcag tcggggaggc ccgagaggcg gtggtttacc ctgtggcgga

107521 tgtcagcggt tcgagtccgc ttatctccag ctcgtgacct tagccgatgc aaaggtatat

107581 gatagcaccc aatttttccg attcggcagt tcgatctatg atttctcatt catggacgtt

107641 gataagatcc ttccatttag cagcacctta ggatggcata gccaacacat taatggcgag

107701 gttcaaacga ggaaaggctt acggtggata cctaggcacc cagagacgag gaagggcgta

107761 gcaagcgacg aaatgcttcg ggaagttgaa aataagcata gatccggaga ttcccaaata

107821 ggtcaacctt tcgaactgcc tgctgaatcc atgagcaggc aagagacaac ctggcgaact

107881 gaaacatctt agtagccaga ggaaaagaaa gcaaaagcga ttcccgtagt agcggcgagc

107941 gaaatgggag cagcctaaac cgtgaaaacg aggttgtggg agagcaatac aagcgtcgtg

108001 ctgctaggcg aagcggtgga gtgccgcacc ctagatggat aaagtccagt agccgaaagc

108061 atcactagct tacgctctga cccgagtagc atggggcacg tggaatcccg tgtgaatcag

108121 caaggaccac cttgcaaggc taaatactcc tgggtgaccg atagcgaagt agtaccgtga

108181 gggaaaggtg aaaagaaccc ccagcgggga gtgaaataga acatgaaacc gtgctgagct

108241 cccaagcagt gggaggagaa agtgatctct gaccgcgtgc ctgttgaaga atgagccggc

108301 gactcatagg cagtggcttg gttaagggaa cggaacccac cggagccgta gcgaaagcga

108361 gtcttcatag ggcaattgtc actgcttatg gacccgaacc tgggtgatct atccatgacc

108421 aggatgaagc ttggatgaaa ctaagcagag gtccgaaccg actgatgttg aagaatcagc

108481 ggatgagttg tggttagggg tgaaatgcca ctcgaaccca gagctagctg gttctccccg

108541 aaatgcgttg aggcgcagca gttgactgga catctagggg taaagcgctg tttcggtgcg

108601 ggccgcgaga gcggtaccaa atcgaggcaa actctgaata ctagatatga ccccaaaata

108661 acaggggtca agttcggaca gtgagacgat gggggataag cttcatcgtc gagagggaaa

108721 cagcccggat caccagctaa ggcccctaaa tgaccgctca gtaataaagg aggtaggggt

108781 gcagagacag ccaggaggtt tgcctagaag cagccaccct tgaaagagtg cgtaatagct

108841 cactgatcga gtgctcttgc gccgaagatg aacggggcta agcgatctgc cgaagctgtg

108901 ggatgtaaaa atgcatcggt aggggagcgt tccgcttaga gggaagctcc cgcgcgagca

108961 ggtgtggacg aagcggaagc gagaatgtcg gcttgagtaa cgcaaacatt ggtgagaatc

109021 caatgccccg aaaacctaag ggttcctccg caaggttcgt ccacggaggg tgagtcaggg

109081 cctaagatca ggccgaaagg cgtagtcgat ggacaacagg tgaatattcc tgtactaccc

109141 cttgttggtc ccgaggtacg gaggaggcta ggttagccga aagatggtta tcggttcaag

109201 gacgcaaggt caccttgctt ttttagggca gggtaagaag gggtagagga aatgccccga

109261 gccaatgtcc gagtaccagg cgctacggcg ctgaagtaac tcatgccata ctcccaggaa

109321 aagctcgaag gaccttcaac aaaagggtac ctgtacccga aaccgacaca ggtgggtagg

109381 tagagaatac ctaggggcac gagacaactc tctctaagga actcggcaaa atagccccgt

109441 aacttcggga gaaggggtgc ctcctcacaa aggaggtcgc agtgaccagg cccgggcgac

109501 tgtttaccaa aaacacaggt ctccgcaaag tcgtaagacc atgtatgggg gctgacgcct

109561 gcccagtgcc ggaaggtcaa ggaagttggt gaactgatga cagggaagcc ggcgaccgaa

109621 gccccggtga acggcggccg taacaataac ggtcctaagg tagcgaaatt ccttgtcggg

109681 taagttccga cccgcacgaa aggcgtaacg atctgggcac tgtctcggag agagactcgg

109741 tgaaatagac atgtctgtga agatgcggac tacctgcacc tggacagaaa gaccctatga

109801 agctttactg ttccctggga ttggctttgg gcctttcctg cgcagcttag gtggaaggcg

109861 aagaaggccc ccttccgggg gggcccgagc catcagtgag ataccactct ggaagagcta

109921 gaattctaac cttgtgtcag gacccacggg ccaagggaca gtctcaggta gacagtttct

109981 atggggcgta ggcctcccaa aaggtaacgg aggcgtgcaa aagtttcctc gggccagacg

110041 gacattggcc ctcgagtgca aaggcagaag ggagcttgac tgcaagaccc acccgtcgag

110101 cagagacgaa agtcggcctt agtgatccga cggtgccgag tggaagggcc gtcgctcaac

110161 ggataaaagt tactctaggg ataacaggct gatcttcccc aagagtccac atcgacggga

110221 aggtttggca cctcgatgtc ggctcttcgc cacctggagc tgtaggaggt tccaagggtt

110281 gggctgttcg cccattaaag cggtacgtga gctgggttca gaacgtcgtg agacagttcg

110341 gtccatatcc ggtgtgggcg ttagagcatt gagaggacct ttccctagta cgagaggacc

110401 gggaaggacg cacctctggt gtaccagtta tcgtgcccac ggtaaacgct gggtagccaa

110461 gtgcggagag gataactgct gaaagcatat aagtagtaag cccaccccaa gatgagtgct

110521 ctcctattcc gacttcccca gagcctccgg tagcacagcc gagacagcga cgggttctct

110581 gcccctgcgg ggatggagcg acagaagtct tgagaatcca agataaggtc acggcgagac

110641 gagccgttta tcattacgat aggtgtcaag cggaagtgca gtgatgtatg cagctgaggc

110701 atcctaacag accgagagat ttgaaccttg ttcctacatg acccgatcaa ttcgatcagg

110761 cactcgccat ctattttcat tgttcaactg tttgacaaca tgaaaaaacc aaaagctctg

110821 ccctccctct ctatctatcc aagggatgga agggcagagg cctttggtgt cccttccagt

110881 caagaatttg ggcttcacaa tcactagcca atatttctct catacctttc ttcgttcatg

110941 gttcgatatt ctggtgtcct aggcgtagag gaaccacacc aatccatccc ggaacttggt

111001 ggttaaactc tactgcggtg acgatactgt aggggaggtc ctgcggaaaa atagctcgac

111061 gccaggatga taaaaagctt aacacctctt attcttatta ctcaaaaaga aaaaaatgaa

111121 aaggtcgtct tattcaaaac ccaattatga catcccttct ctcccacttc acacctcgga

111181 acgcgctgtt cttatagaga gaaaggcgct ttcacgtctt cttaacccga aatggctgag

111241 gggagaaaag gttccttttt gagggtactc cagggaacag atccagtgga gacggggtgg

111301 ggcctgtagc tcagaggatt agagcacgtg gctacgaacc acggtgtcgg gggttcgaat

111361 ccctcctcgc ccacaaccgg cccaaaaggg aaggaccttt cctttacctc tgggggtagg

111421 aaaatcatga tcgggatagc ggacgcaaag ctatggaact ggggtgtggg tcttttgtcg

111481 aaatggcctt attcttttta tttatcgtga aggaaaaaat cgatacatat agtatgcctg

111541 gcccgaatca gcatatttgt gttttactcc ccgtaactct tcctcagcca ggcttgggaa

111601 gaatagcaga gcaaatacaa gtattagtag catagcaaaa atgcgttcct cgtcattaat

111661 atgtttgctc gcggtaattg tggcctctcg ggagaatcga tgactgcatc tttgatgcac

111721 tgctagtaca tcatctgaga attatgaatt ggctagttgt aaatagcccc agggctatgg

111781 aacaaaggat tatcccggat ctacaccgag gtattgacgg cgattctcaa atatcgcaga

111841 acagaatgcg atgagataga gtgcaataga aacaaagaca gggaacgggt tacctactcc

111901 taacggtcaa agcgagccct ttaattctgt aattctgaat tctttaatta agaattcatc

111961 aaatctcccc aagtaggatt cgaacctacg accagtcagt taacagccaa ccgctctacc

112021 actgagctac tgaggaataa cgggagattc gatctcatag agttcaactc ccgctctcaa

112081 cccatgacca atatgagccc gaagcttcct tcgtaactcc cggaacttct tcgtagtggc

112141 tccgttccat gcctcatttc atagggaacc tcaaagtggc tctatttcat tatattccat

112201 ccatatccca atattccatc catatcccaa ttccattcat ttaatatccc tttggtgtca

112261 ttgacataag agatgtcatt caatcgaaga agagggtatc attgacataa aagatgtcat

112321 ttctagtcta tctgtttcta tctatggaaa gtgaagaaat catcatatag taatcgagaa

112381 attgcaatag aaaagaaaaa agggaggttt gtgatgattt tgaaatcttt tctactaggt

112441 aatctattat ccttatacat gaagataata aattcggtcg ttgtggtcgg actctattat

112501 ggatttctga ccacattctc catagggccc tcttatcttt tccttctccg agctaggatt

112561 atggaagaag gaaccgagaa ggaggtatca gcaacaactg gttttattac gggacagctc

112621 atgatgttca tatcgatcta ttatgcgcct ctgcatctag cattgggtag acctcataca

112681 ataactgtcc tagttctacc gtatcttttg tttcatttct tctggaacaa tcacaaacac

112741 tttttggatt atggatctac taccagaaat tcaatgcgta atctcagcat tcaatgtgta

112801 ttcctgaata atctcatttt tcaattattc aaccatttca ttttaccaag ttcaacgtta

112861 gtcagattag tcaacattta tatgtttcga tgcaacaaca agatgttatt tgtaacaagt

112921 agttttgttg gttggttaat tggtcacatt ttcttcatga aatgggttgg attggtatta

112981 ttctggatac ggcaaaatca ttctattaga tcgaatgtac ttattcgatc taataagtac

113041 cttgtgtcag aattgagaaa ttctatggct cgaatcttta ctattctctt atttatcacc

113101 tgtgtctact atttaggcag aataccgtcg cctattgtca ctaagaaact gaaagaaacc

113161 tcaaaaacgg aagaaagggg ggaaagtgag gaagaaacag atgtagaaat agaaaaaact

113221 tccgaaacga aggggactaa acaggaacaa gagggatcca ccgaagaaga cccttccctt

113281 tgttcggaag aaagggagga tccaaaaaaa ctacatgaaa aaaaaaagag gcaagaaatt

113341 ttgaagttag aaatacttaa agagaaagaa gataaagacc tcttctggtt tgaaaaacct

113401 cttgtgaatc ttcttttcga ctataaacga tgtaatcgtc cattgagata tataaaaaaa

113461 aatttatttc aaaatgctgt aagaaatgaa atgtcacaat atttttttca cgtatgtcca

113521 gttgatggaa aacaaataat atcttttaca tatccaccca gtttatcgat ttttttggaa

113581 atgatgcaaa gaaagatgtc tttgtgtacg accgaaaaac tatcccccga agatctgtat

113641 aatcattggg tttataccaa tgaacaaaaa aggtacagct tgagcaatga attcataaac

113701 cgaatagaag ttctaaacaa gggatctctt actatggatg tgcttgaaaa aaggaccaga

113761 ttgtataatg ataaaaataa ccaagaggat aagaataacc aagaagataa gaataaccaa

113821 gaagataaaa ataaccaaga agataagaat aaccaagaat gcttgcctag agtgtatgat

113881 ccttttttaa acggaccata tcgtggaaca ataaaaaaag tgtattcacg ttcaatggtg

113941 gatgactcaa tcacttcgac agaagattct atagggacag aagattctat aggaatggtt

114001 tggataaata agattcatga taggcttcct actgattacc aaaaacttga acataaaacg

114061 gatacattta atggagaacc attatcgaca gacattggtc ctttcttgac ctctatcagt

114121 gaattagcta ggaaatcaac aactggtttt agtctgaatt ttaaaaagct tgttttaata

114181 tccgaacaaa gaagatttga ttcagaaaat aaaaaaaaat gtttgaaatt tctattcgat

114241 gtaattacaa ctgatcaaaa taatcaaaca attcaaaata aatctattgg aatagaagaa

114301 atcggtaaaa agattcctcg acgatcatac aaattgatca attcttttga agagcgggag

114361 gaggaaaatg aggaagaatc agaagaatca acagaaaatc atgggattcg ttcaagaaaa

114421 gccaaacgtg tggtaattta tactgataag gcggatccgg atcagaatac caatactcat

114481 actagtacca gtactaatag tgatcaagca gaagagttgg ctttggtacg ttactcgcaa

114541 caatcagatt ttcgtcggga tatagtaaaa ggatccatac gcgctcaaag acgtaaaatg

114601 gttatttggg aaatgtttca agcgaatgca cattccctgc tttttttgga cagaatagac

114661 aaaacttttt ttttttcttt tgatatctcc cgaacaatga atctcatttt tagaaattgg

114721 atagatacag gaccgaaact caaaacttcg gattctgagg aggaagaggc aaaagaagag

114781 gcaaaaaaaa tggaagataa aaaaaacgag aatgaacgga tagcaatagc agaaacatgg

114841 gatactttta tatttgctca agcaataaga ggtactatgt tagtaaccca atcgattctt

114901 agaaaataca tcatattgcc ttcattgata atagctaaaa acctcggccg tatgctctta

114961 tttcaattcc ccgagtggta cgaggatttg aaggagtgga atagagaaat gcatgttaaa

115021 tgcacctata atggtgttca attatcagaa acagaatttc cgaaaaactg gttaacagat

115081 ggtattcaga taaaaatcct atttcctttc tgtctgaaac cctggcgcaa atccaaacta

115141 cgatcccatc atagagatcc aatccaaaag aaagggaaaa cagaaaattt ttgtttttta

115201 acaatctggg gaaaggaaac cgaactacct tttggttctg cccgacaaca accttccttt

115261 tttgaaccta tttataatga attcgaaaaa aaaaagataa aagtgaaaaa aaaatgtttt

115321 ctagttctaa gagttttcaa aaaaaaaaca aaacagttta gaaaggtctc aaaagaaaaa

115381 acaagatgga ttatcaaaac gattctattt ttaaaaagaa aaattaaaga gtttgcaaac

115441 gtaaatccaa ttttcttatt tgtattgaag aaagtatatg aaccgaatga aaatggaaaa

115501 gattccataa tcataagcag taataaaatt gttcctaaat cgacatcgac cattcgaatt

115561 agattcatgg attgggcaaa ttattcactg acagaaaaaa aaaagaaaga tctgtccgat

115621 agaacaaccc taatcagaaa tcaaatagaa aggggtgcaa aagacaaaag aaaaatattt

115681 ctaactccgg atataaatat tagtcctaac gatacaagtt gtggtgataa aagatcggaa

115741 tcgcagaaac atatttggca gatatcaaaa ggaaaaagta acagattcat attcatacgc

115801 aaatggcact attttttgac atttctcgac gaaagaatat acatacatat ctttctatat

115861 actgttaatg tttctagagt caacgtacaa cttttccttg aatcaacaaa aaagattatc

115921 gataaataca ttcacaaaga agggattgat gaaataaatc aaaaaaaaat gcactttatt

115981 tcgactataa aaaagtctat ttctaatatt agtaaaaata aatcaaagat ttctggtgac

116041 ctatattcct tttcacaagc atctgtattt tacaaattat cgcaaatcca agctattaat

116101 aagaagtatc atttgagatc tctacttcaa tatcgcgaag catatcttat tcttaaggat

116161 agaatccgga atttttttgg aacacgaaga atattagatt ccaaatcaag gcataaaaaa

116221 cttccgaatt ctggaatgaa tgagtggaaa aactggttaa ggggtcatta tcaatacaat

116281 ttatctcagg ctaggtggtc taaattagta ccgcaaaaat ggcgaactag ggtcaattgg

116341 cgtcgtacga ttcaaaataa agactcaaaa aagaattcat atgaaaaagc ccaattcatt

116401 cattacgaga aaaaaaatga ttatgaagtg aattcattga cgataaaaaa agcaaaatta

116461 aaaaaaaact acagatatga tcttttttca tataaatata ttaattatgg ggataggaaa

116521 gactcatata tttatccatc ctcattacaa gtaaacgagg accgagagat tccatataat

116581 tacaacacac ctaaaattga accattttat gtactggggg atatatgtat tagtgattat

116641 ctaggagaag agtctattat tggtacgggt aaaagtacgg atagaaaata tttggagtgg

116701 aaaattttcg atttatttct tagaaagaat atcgatattg agtcctggac cgatacggat

116761 accgggacca acattaataa aatgactaaa accgagactg attattatca aatgattgat

116821 aagaaagatc ttttctatct cacgattcat caagaaatca acccacccaa tcaaaaaaaa

116881 aagttttttt tgatgggaat gaataaagaa atgctatatc gccccatatt aaatacgaaa

116941 tcttggttct tctcagaatt tgtgccactt tatgatgcat ataagatcaa accgtggatt

117001 ataccaatca aattacttct tttgattttt aatggaaatg aaaacattag tgaaaacaaa

117061 aacattaatg aaaatcaaaa aaaggatctt cgtatatcat ctaatcaaaa agaatatctt

117121 gaattaaaga atcgaaatca agaagaaaaa gaacagctcg gccacggaaa tattggctca

117181 gacgcacgaa aacgacaaaa agattttgaa aaggattaca cggaatcgga cattcaaaaa

117241 cgtgaaaaga aaggacaacc cgagagtaac aagaaagcaa aacaagagtt attcctgaaa

117301 aaatatttgc tttttcaatt gagatgggat gatcttttga ataacagaat tttcaataat

117361 gttaaggtat attgtttcct gcttagacta ataaatgcaa aggaaattgc tatatcctct

117421 attcaaggag gagaaatgca cctggatgta atgttaattc agacgaatcc aactcttcca

117481 gaattgataa aaaagggaat attgattctt gaaccagtac gtctgtctat aaaatgggat

117541 agacaattta ttatgtatca aaccataggt atctcattgg tccataataa taaatgccaa

117601 actaatggaa gatatcgaga aaaaagatat gttgatgaga attatttcaa tggatccatt

117661 gtacaacata aaaagatgct tgtgaataga gacgaaaatc attatgattt gcttgttcct

117721 gaaaatattc tatcccctag gcgtcgtaga gaattgagaa ttctaatttg tttcaattcc

117781 ggaaatagga atgctatgga tagaaatccg gtatttttca atgacaacaa tgtaaggaac

117841 tgggtccaat ttttggatga ggacaagcat attgatacag atataaataa attcattcaa

117901 ttcaaattgt ttctttggcc caattatcga ttagaggatt tagcttgtat gaatcgctac

117961 tggtttgata ccaataatgg cagccgtttc agtatgtcaa ggatacatat gtatccacga

118021 ttcggaatta gttgatggtt ggtacatttc ctatatatca ggtgtcggat ttggattgaa

118081 tctagaaatg atttggcaga atcttcttag gtcaaaataa ttttgttttg tgggtacaaa

118141 aattgccctt ctatcgaatc aaattacaaa ttgtacttac aattcatttg aatttaattt

118201 tgatttgatt tgatagaata tagaataaag taatatatga ataaatccag attattgggt

118261 gtatcagatc aaaataactg gcattattat tattcctgat tggtaaaatc catatatgtg

118321 gaaaaaaaaa aaaaaaagag agaaatttta tttttatggt tatggtaaaa aattcattca

118381 tctcagttat tccgaaagaa gaaaaaaaca aagggtctgt tgaatttcaa gtaatcagtt

118441 tcaccaataa gatacagaga cttacttcac attttgaatt gcacagaaaa gattatttat

118501 ctcagatagg tttgcggaaa attctgggaa aacgtcaacg actgctggct tatttgtcaa

118561 agaaaaatag agtgcgttat aaaaaattaa tcgatcaatt agatattcgg gaaccaaaaa

118621 ctcgttaatt tgaagattat ttgaattctt tggtttatta gatcttgaat tttgatgaac

118681 ctcatttatt tccttttcgg caattcatag aataatggat cggagaagaa aacatatgaa

118741 tgtaccggct acaagaaaag acctcatgat agttaatatg ggtcctcacc acccatcaat

118801 gcatggtgtt cttcgactta tcgttactct agatggtgaa gatgttattg actgcgaacc

118861 cgtattgggt tatttacaca gagggatgga aaaaattgcg gaaaaccgaa caattataca

118921 atatcttcct tatgtaacac gttgggatta tttagctact atgttcacag aggcaataac

118981 ggtaaatgca ccagaacaat taggaaatat tcaagtaccc aaaagagcca gctatatcag

119041 agtaattatg ctggagctga gtcgtatagc ttctcattta ttatggcttg gaccttttat

119101 ggcagatatc ggttcacaga ctcccttctt ctatattttc agagaaaggg aattgctata

119161 tgacctattc gaagctgcca caggtatgcg aatgatgcat aattatttcc gtatcggggg

119221 agtcgctgct gatctacctc atggctggat agataaatgt ttggatttct gcgattattc

119281 tttaacagga attgttgaat atcaaaagct tattacgcaa aatcccattt ttttggaacg

119341 agttgaaggg gtgggcatta ttggtgggga ggaagcaata aattggggtt tatcgggacc

119401 aatgctacga gcttccggaa tccaatggga tcttcgtaaa gttgatcatt atgagtgtta

119461 cgatgaattc gattgggaag tccagtggca aaaagaagga gactcattag ctcgttattt

119521 agtacgaatc aatgaaatga cggaatccat aaaaattatt caacaggctc tagaaggaat

119581 tccggggggg ccctatgaga acttagaagt tcgacgcttt gatagagcaa gtgattccga

119641 atggaatggt tttgaatatc gattcattag taaaaagcct tctcccactt ttgaattgtc

119701 gaaacaagaa ctttatgtga gagtcgaagc cccaaaggga gaattgggaa tttttctgat

119761 aggggataat agtgtttttc cctggagatg gaaaattcgt ccacctggtt tcatcaattt

119821 gcaaattctt cctcagctag ttaaaagaat gaaattggcg gatatcatga cgatactagg

119881 tagtatagat atcattatgg gagaagttga tcgttgaaat gataattgat acgacagaag

119941 tacaagctat caattctttt tccagatcgg aatccttaaa agaggtctat gacctcttat

120001 ggctgcttgt ccctattttt actcctgtat cgggaatcac aataggcgta ctcgtgattg

120061 tgtggttaga aagagaaata tctgcaggga tacaacaacg tatcgggcct gaatatgccg

120121 gccccttggg aattcttcaa gctctagcag atgggaccaa actacttttg aaagaggatc

120181 ttctcccatc tagaggagat gttcgtttat tcagtatggg gccatctata gcggtcatat

120241 caattctact aagctattta gtaattcctt ttggctatcg ccttgttcta gccgatctca

120301 gtataggcgt ttttttatgg atcgctattt ccagtattgc tcctattgga cttcttatgt

120361 caggatatgg atcgaataat aaatattcct tttcaggtgg tctacgagct gctgctcaat

120421 ctattagtta tgaaatacca ttaactccgt gtgtgttatc aatatctcta cgtgtgattc

120481 gttggaacat gaacctttac ccctttcctg gaaataaagg aaaggggttg gatgtgttga

120541 atagatacct ttctcttttt attcatcatt cgggtcgatg agttaaacca gatagttata

120601 tgagtgaaac aaaacagctt atgaatttgc agtaagaaga ttgattctca ttccctatgt

120661 acgagagtaa agtggaagta aacataagcg gtcgaaactg tttaccccaa gattggttga

120721 ttagtcatca tgacttgaag cgggtgcaaa agatcaactg tatggagttt ctactattgt

120781 atagtatgtt gttgtatgtt gtgtatgttg tatgtattac cataccgggg atcaatcaaa

120841 aatgagtgga cggttaggaa cacaaaggta cacaaaggat tagtgatgaa gataatgtaa

120901 ggtatccaaa gggatatttc tgcataacat aaaaggaatc ataatgaggg ctttaagttc

120961 gtagaaatga tcaagcagta cttcctcacg attccgatcc agagtatgct tctatccact

121021 gattaaataa atgactgtcg agaacgaagt aatcctttga tttgattttt tagaaacccc

121081 cttctgagtg agaaagaaga acaggaacga aagaaatgga atgcaatagg aaaactgaat

121141 aataagagat ctttgtttat tctttctttc ctcaatccta tccatattta tacggataga

121201 attcttataa tgatttatca actataactc atgaattagt gtctaattct ttttcgtacg

121261 aaaagtatgg gtcgaaatat ctattgaaac aacgagtatt ttattgaagg attaagttat

121321 tactgaacta aaagaattct aagtctaatt agaaaataaa ggatgagatc aattcggaag

121381 cgcttttttt tttttttatt atggcggacg gaattccatt ggtctaattc gggactcttc

121441 gatacatctt tactctaatc tacactacaa acatgccgag gtaataatga accagtcctt

121501 agatttattt gtggccatcg aggagccgta tgaagctgag gtctcatgtg cggttctgga

121561 atagcgatgg gaatagtgat gttatcatcg actatgatta tctaacagtt caagtacagt

121621 tgatatagtt gaggcacagt caaaatatgg gttttggggg tggaatctgt ggcgtcaacc

121681 tatagggttt atagtttttc taatttcttc cctagcggaa tgtgaaagat taccttttga

121741 tttaccagaa gcagaggagg aattagtagc aggttatcaa accgaatatt caggtattaa

121801 atctggttta ttttacgttg cttcttacct aaatctacta gtttcttcat tatttgtaac

121861 agttctttac ttgggcgggt ggaatttctc tattccgtac atattcattt ctgaaccttt

121921 tggaataaat aaaacaggtg gagtctttgg aatgacaatt ggtatcctta ttacattagc

121981 taaagcttat ttgttcctgt tcattcctat cacaacaaga tggactttac ctaggatgag

122041 aatggaccag ctattaaacc ttgggtggaa atttctttta cctatttctc taggtaatct

122101 attattaaca acttcttccc aactcgtttc gctataacaa aatatgatat tctagattca

122161 taacctatct agagcaagag aaagaaacat caaactattc atggatatcc acgatatgtt

122221 ccctatggtg actgggttca tgaattatgg tcaacagaca atacgagctg caaggtatat

122281 tggtcaaagt ttcatgatta ccttatctca cgtgaatcgt ttacctgtga ctattcaata

122341 tccttatgaa aagtcgatca catcggagcg ttttcgtggt cgaatccatt ttgaatttga

122401 taaatgcatt gcttgtgaag tatgtgttcg ggtatgcccc atagatctac ccgttgttca

122461 ttggagattg gaaacggata ttagaaagaa acgattgctt aattatagta ttgattttgg

122521 aatctgtata ttttgtggta attgtgtcga gtattgtcca acaaactgtt tatcaatgac

122581 tgaagaatat gaactttcta cttatgatcg tcacgaattg aattataatc aaattgcttt

122641 gggccggtta ccaatgtcag taattggaga ttacacaatt cgaacaatta cgaattcgac

122701 tccaatcaaa ataatcaggg gtaaacctct tgattcaaaa acgattacca attactaaga

122761 ttccgttttg atttaaagta aaggagtgag gcttctttca ttttgctagg tcagtaaata

122821 actattgatt ggtgagaatc aaggcttgat tttgatttag aatggattca tagatctgtg

122881 atgatttcaa aatatacaat ttcgaactac tctttcagat acagtagcgg gattgatcca

122941 ataactgtat ctatataaat ctacacccct ttaggattca attaggaacg tatcatatac

123001 aagaaaaaaa taaggtaacc tcttttttct tgggtcggtt agtaagttta tgaaatattt

123061 cgatttattt atctcttttt ttacacataa tggatttacc tggaccaata catgatattc

123121 ttttagtatt tctgggatca ggtcttatat tagggggtct gggggtggtc ttacttacca

123181 acccaattta ttctgctttt tcattgggac tggttcttgt ttgtatatcc ttattccata

123241 ttccatctaa ctcctatttt gtagctgctg cacagctcct tatttacgta ggagctgtaa

123301 atgttttaat cctatttgct gtgatgttca tgaatggttc agaatattac aaagatttct

123361 atctttggac cgttggggat ggggtcactt cactggtttg tacaagtatt cttttttcac

123421 taattactac tatctcggat acgtcgtggt acgggattgt ttggactaca agatcaaatc

123481 agattataga gcaggaccta acaagtaacg ttcaacaaat tgggattcat ttatcaacag

123541 atttttacct tccatttgaa ctcatttcta taattctttt agttgctttg ataggtgcaa

123601 ttgctatggc ccggcagtaa agtaattaag taataaatac ttagatccaa aataaaataa

123661 ataaagtctt gttttgttct atgttatcac atccattttc cttcagttcc attttcatat

123721 tctattgttc atatatgaaa ttgaaagggg tttagttcga tccattacta ataccttact

123781 ttgtttcgta cttcatttat attctaatca aatcggtgaa attgttgttc atattgaaat

123841 gaatcaaaat tgatgagggg ttggtcaatg atgaccgaac atgtacttat tttgagtgcc

123901 tatttatttt ctatcggtat ttatggattg atcacaagtc gaaacatggt tagagcactt

123961 atgtgtcttg aacttatact gaatgcggtt aatataaatc tcgtaacatt ttctgatttg

124021 tttgatagtc gtcaattaaa aggagatatt ttctcgattt ttgttatagc tattgcagcc

124081 gctgaagcag ctattgggcc ggctattgtt tcatcgatcc atcgtaacag aaaatcaact

124141 cgtatcaatc aatcgaattt gttgaataaa tagtattaat gatataaata aagacagata

124201 tccacaaaat attcactaat ttagaactag catgtatgat tcgtatgacc atgcttgttg

124261 aaacgtaaga aatcaaagta tcttggccct tgctcatgaa cagatccaga aatagattga

124321 ttatcaaaaa agttctggta aaccactgat tcgtctggcg tctacaacat aatatatata

124381 attcaattac taatgaagcc agatcgaaaa ttcataaagt tcaaaaaatt tatagatcca

124441 atgtcgcatt cagtaaagat ttatgataca tgtatagggt gtactcaatg tgtacgagcc

124501 tgccccacag atgtattgga aatgatacct tgggacggat gtaaagctaa acaaattgct

124561 tctgctccaa gaacagagga ctgtgtaggt tgtaagagat gtgaatccgc ttgtccaacg

124621 gatttcttga gtgttcgggt ttacttatgg catgagacaa ctcgcagcat gggtctagct

124681 tattgatacg ttctagaaaa atccacttga atccatttga ttcctcttta ccgacaaaaa

124741 cccgtactcg agaaattatt ccgagcgcgg gtttttctgg tcaaagtcta tcttgtcttt

124801 accacgagtt attttccttg gttaacaata attgttgttt tgccgatatc cgcgggttcg

124861 tcaattttct ttctccctcg tagagggaat aaaaataagg tggttcggtg gtatactatt

124921 tgtatatgct tattagaact ccttctaacg acctatgcgt tctgttatca tttccaattg

124981 gacgatccat taatccaatt agaagaggct tataaatgga taaatacttt tgattttcac

125041 tggagaccgg gaatcgatgg actttccata ggacccattt tactgacggg attcatcact

125101 actttagcta ctttagcggc tcggccagtt actagagatt cgcgattgtt ccatttcctg

125161 atgttagcaa tgtatagtgg tcaaatagga tcattttcct ctcgagacct tttacttttt

125221 ttcctcatgt gggaattaga attaattcct gtttacctac ttgtatccat atggggaggg

125281 aagaaacgtc tgtactcagc tacaaagttt attttgtaca cagcgggggg ttctattttt

125341 ctcttaatgg gagttccggg tatgggttta tatggctcca atgagccaac attaaatttt

125401 gaaacattag ctaatcaatc gtatcctttg ggattggaaa taatattcta tattggcttc

125461 cttattgctt atgctgtcaa atcgccgatt atacccctac atacatggtt accagatacc

125521 catggagaag cacattacag tacatgtatg cttctagcgg gaatcttatt aaaaatggga

125581 gcgtatggat tggttcggat caatatggaa ttattacccc atgctcattc tatattttct

125641 ccctggttga tgatagtagg agcgattcaa ataatctatg cagcttcaac ttctttcggt

125701 caacgcaatt taaaaaagag aatagcctat tcttccgtat ctcatatggg tttcacactt

125761 ataggaattg gttccataac cgatacggga atcaatggag ccattttaca aataatctct

125821 catggattta ttggtgctgc actttttttc ttggcaggaa cgagctacga tagaatacgt

125881 cttgtttatc tcgacgaaat gggaggaata gctatcccaa tgccaaaaat atttaccatg

125941 ttcagtagct tctcgatggc ttctcttgca ttgccaggaa tgagtggttt tgttgcggaa

126001 tcagtagtat tttttggaat aattactagc ccgaaatatc ttttaatgcc aaaaatacta

126061 ataactttcg taatggcaat tggaatgata ttaactccta tttattcatt atctatgtca

126121 cgtcggatgt tctatggcta caagctattc aacgttccaa actcttattt ttttgattct

126181 ggaccacgag aactatttgt ttcggtctgt atccttctac ctgtaatagg tattggtatt

126241 tatcctgatt tcgttctctc gctatcaatt gacaggatag aagctattct atctatttat

126301 tttcataaat agttttcatc aataagacat tacattaatg taaaagaact gtgtgatttt

126361 taaaagcgtt caatgaaaga aaaaaaaaat gaagtgaatt ataatcagat acatctaaag

126421 ttttttcgaa ccatttgaat caagtagtga ttcaaatggt tcgaaaaaac ttgcacaaac

126481 cttctttata taatatacgg gacgatgttc ctatgtattc ttcaggccct ttcttcaatt

126541 agttgttaat gtgaatgaac cataactatg tagtcctatt cctaatagat tgaccccaaa

126601 atagcatatc caaattataa gaaatcctat agaagccaca attgccgaat ccacaccctg

126661 aaagctctga tttgttctac tgtgtaaata aatcgcgaat atggtccaag taataaaagc

126721 ccaagtttcc ttggggtccc aattccaata agacccccat gcctcattag cccatactgc

126781 tcccgaaaga atacctatgg ttaaaaaagt aaaccctaga ctaatgacac gataactaca

126841 ctgatctaat tgttgagtta attgatacct gtgataattt ctaaatgaaa gaaaagaagt

126901 gttttgtaaa acgcttcttt tttcattcac aaaggaaaat gacccaatta ataaatgatt

126961 gcttttgcga ggaatatcta ggttttttcg aaatgtaatg actaaaagag ctatggataa

127021 taacgatcca cataaaagag ctgcataact caataacatc atacttacgt gcatcattaa

127081 ccactgggat tgtagagcag gtactaatat tgcagattga tgcatttcgg ttgaaagacc

127141 cgaagtggca aagccttggg taaaaatagc acttggcgcg gttattgcgc ttaaataact

127201 tttctggttc cgtcttttag gaaacatatg aataatggag aaactccatg aaagaaacat

127261 taaagattca tataaatcgc ttaacggcaa atgtctcgaa taaatccaac gagtaactaa

127321 taatcctgtt atacagaaaa aggtagccat catggctttt tctgacaaat caaatagtac

127381 tacggtttca tggattaata aggtcatcaa atgaatcgta ataacaattg aaatgataga

127441 aaaagagatg tgagttaata tatgttctaa agtggcaaat atcataattt tttttagggg

127501 ggtatcccca attacggaat ggaaatccgg aattgaattc attataggat ctattgtgcc

127561 ttttttagag gatgccgcca ctcggactcg aaccgagatg ctctagcact gcttcctaag

127621 agcagcgtgt ctaccaattt caccatggcg gcatcatcga aataatcata gtccatatga

127681 tgttcaatcg tcgagattga gggatttaat gcaatctatt ttaggaaatg ttagaatcga

127741 tgaataaaga cccgttcaaa taaatattta aacgctcaat aatccttagt atcgtggcag

127801 ggggtcgttt taaacagcgg gcttttccgt attataagtt cttctggaat agttggaact

127861 agacggttat gccctgagtc caggtataga actaagaatt tttttctcat tttttgacga

127921 ttcatttctc atttatctga tttgatagat ccgaaatgaa aaagattcat ttttcaatga

127981 acaaaataaa acaagatttt ttatatatca caacttcatc actacatcca aaggatttct

128041 atgaaaattt ggatagtttg gtatcaaaga tgtattaatg attgggatct tcattgtata

128101 cagaacataa tttgtgtgag gatttaccaa acccattagg tattggaccg ggcatatcgt

128161 ataacaaaag agtttcaatc tttatcggaa ttctactgta tgtactttaa cttagaaaac

128221 ttagaaaatc aaatttaaac tgataagatc tctttatata tagatttgaa atctaatatt

128281 aatagataaa ataatttaga tattggatct agatatatcg attgatcgat cctccagctc

128341 aaacatggga taggatccat tggggttgga ttacgtaacg taagattgac tcattattta

128401 gtacataaat atcgatctaa atgggatcct ggcagtttta ttattttgtg tttttttttt

128461 ttatctgttc gaaacaagag ggagtccttg tagatatgta gtgattcaga gagctacaaa

128521 tcaaagtttt aaaatgtata ttttaatcaa ttagtttcaa taatccattg actttgacta

128581 tgaatcttac ccccgcctta ctttggaaca aaaaaggggg ctctaacctc gttcatactt

128641 gtttcagatt ttccaaaaat cttaatgatg tataactatt ggagatacat aatccaataa

128701 ggcaatccct tcctaagaaa aaaaggaaga gttttgttat tgtgaaaaat gaatcgatgg

128761 ggaaagttac aatccccatc tcgcgaatta taaaaaccaa tcaatgaaag gtgaaacctt

128821 gtattttgtg tctaactact tctttttctt tctttttttt ttttttttca aacaaaaaaa

128881 gaaatcattt ttagaaccta gtatacagaa tacttcatat tctacatacc acaacagcta

128941 gttctatctc atattgatga gttcaaaaca ttagttaatg tgaattcttc atcttataaa

129001 tttaatcatc caattcatcg agtcatgctg atgcagattc agatcattcc aaggctttat

129061 tacttgtttg tcgcacaaaa aaactttttg aatgcccggt agaaatagat ttagctaaag

129121 ataaagcttt tgccgcggcc tgatatcccc ttcctttcca aatatttcta cgaatatgct

129181 tttttgacag agaagtacgt ttctttggaa ccgccatttc aaaataaaag ggtcactcat

129241 ttttatagtt ggacgtgaaa gacatttatt gttcaattca aaatagattg ttctttctat

129301 taactattca ttatctatct ttattccata gccgatactt atgcttactt cataacatag

129361 aagagtatgg atacagatag atcagcatcg catatggtat cattaaggat aaaaaaagaa

129421 atgaaataga agaaaaaaga aaaaacgatg tgattttcaa gggaattttt tttttttcac

129481 atttccatta catccaatgt tcgaagaaag aataatttgt actgattggg ggcttcatat

129541 ctttattcaa tctatgtcta cgggcgggat ctactaccgt tgaatcggat gccattgata

129601 agaattaaaa aggttccaat tcatatctca gtctatttag ataatatata tatatattat

129661 aaatgttatt tttaataaat cgaaaagctt aagaaccctt tcctaattta ggaaaccaat

129721 aatgaatgta acctttttct attaattgat caagctcgga gatagagtcc acataattta

129781 aattgaaatt aaatctaaag tagttaatcc gttaaacaat acattacttg ataaaataaa

129841 gggaatttga gtaatttctc tttttagttc tatgcgaagt gacaagtatt ctagcatttt

129901 tcataaacac ataaacataa gtttgtttta ttggactaga agccaatcaa ttccagaatt

129961 tgttttagtt aatgactccg aagaaactaa aaaaaaacta ggtttattgg atcgagttat

130021 tggcagatcc tacgatacat atcagaataa agtacttgaa atttgggtat cattcttttt

130081 ccttactata ttgatataaa tatggataga aatcaccgta tcgtatgtat atagtagaat

130141 aattgaaaat ctcgtatttt ttatcttaat aaatatcttc gttaataact aggtagtagc

130201 ttttaactag taacttgatt atttgaattt tttgtttttt tttttcaata gtttggaaag

130261 aatcagaata attaagaatg aaaaatcata tttgagttct tttatatctt gtatatcttg

130321 attttttttt tatttatttg attattgctc cttccggaag aaataagggc tggtgaatgg

130381 gaaacaatta ataagaataa aaaaagaaag tttgattttc ttatggaaca tacatatcaa

130441 tatgcatgga tcataccttt cgctctactt ccagttacta tgtcaatagg gttgggactt

130501 ctgcttgttc cgaccgcaac aaaaaatctg cgtcgtatgt ggacttttcc tagtgtttca

130561 ttgctaagta tagttatggt tttttcgtcc gatctgtcta ttcaacagat aaatggcagt

130621 tctatctatc aacatctatg gtcttggacc atcaatactg atttttcctt agagttcggc

130681 tacttgatcg atccacttac ttctattatg tcaatactaa tcactacggt tggaatcatg

130741 gttcttattt atagtgacaa ttatatgtct catgatcaag gatatttgag attttttgct

130801 tatatgagtt tttccaatac ttccatgttg ggattagtta ctagttccaa tttgatacaa

130861 attcatattt tttgggaact agtgggaatg tgttcgtatt tattaatagg tttttggttc

130921 acacgaccgg ctgccgcaaa tgcttgtcaa aaagcgtttg taactaatcg tgtaggggat

130981 tttgggttat tattaggaat cttaggtttt tattggataa cagggagttt cgaatttcga

131041 gatttgttcg aaatcttcaa taacctgatc cgtaataatg gggtcaactc tttatttgct

131101 actctgtgtg cctccctatt attcgtcggt gcagttgcta aatccgcaca atttcccctt

131161 catgtatggt tacctgatgc catggagggg cctactccta tttcagctct tatccatgct

131221 gctactatgg tagcagcagg catttttctt gtcgctcgat ttcttccgct tttcacagtc

131281 ataccttaca taatgaatct catttctttg ataggtgtaa taacggtact attaggagct

131341 actttagctc ttgctcaaag agacattaag agaagtttag cctattctac aatgtctcaa

131401 ttgggttata ttatgttagc cccagggata ggctcttatc gagctgcttt attccatttg

131461 atcactcatg cctattcgaa agcattattg tttttaggat ccggatcaat tattcattca

131521 atggaaccca ttgttggata ttctccagat aaaagtcaga acatggttct tatgggtggt

131581 ttaacaaaat atgtgccaat tacaaaaaat acttttttat taggtacact ttctctttgt

131641 ggaattccac ctcttgcttg tttttggtct aaagatgaaa ttcttaatga tagttggttg

131701 tattcaccta ttttcgcgat aatagcttgt ttctcggcgg ggttaactgc attttatatg

131761 tttcggatgt atttacttac ttttgatggt catttacatg ctcattttca aaattacagt

131821 ggcactcaaa atagctcgtt ctattcaata tctatatggg ggaaagaagg aaccaaacca

131881 gttaacagaa atttgttttt atcaacaatg aataataatg aaaaggtttc ctttttttcg

131941 aggaagatat acaaaatgaa cggaaatgta agaaatctga tacgctcctg taggatttat

132001 tttgaaaata aagacacttc aacgtatccc catgaatcag acaatactat gcttttgcct

132061 ctacttatat tggtcctatt tactttgttc gttggatcca taggaattcc tttcgatcaa

132121 ggagtaatcg attttgatat attatcgaaa tggttaactc catcaataaa ccttttacat

132181 caaaattcga actattctgt ggattggtat gaatttgtga caaatgcaat ttattcagtc

132241 agtatagcct gttttggaat attcatagcg tctattttat atgggtctgt taattcatct

132301 tttcagaatt tggacttaat caattcattt gttaaaaaaa caggctctaa gaaaatttta

132361 ttggaccgaa taataaatgt gatatacaat tggtcatata atcgtggtta catagatctt

132421 ttttatgcaa catgcttaac tacaagtata agaggattag ctgaagtaac tcatttttta

132481 gatagacggg taattgacgg aattaccaat ggtgttggtg ttgcaagttt ctttgtagga

132541 gaagggatca aatatgtggg gggagggcga atctcttctt atctctttgt atatttatca

132601 tatgtatccg gctttttatt aatttactat atctattatc tattcttttt gttttgaata

132661 gaataagaag tgactagact tggttatttt tatcattata caatctggtc cttttttcaa

132721 gcacatccat agtaagagat cccttgttta gaacttctat tcggtttatg aattcattgc

132781 tcaagctgta ccttttttgt tcattggtat aaacccaatg attatacaga tcttcggggg

132841 atagtttttc ggtcgtacac aaagacatct ttctttgcct tggttatttt tatcattata

132901 caatctggtc cttttttcaa gcacatccat agtaagagat cccttgttta gaacttctat

132961 tcggtttatg aattcattgc tcaagctgta ccttttttgt tcattggtat aaacccaatg

133021 attatacaga tcttcggggg atagtttttc ggtcgtacac aaagacatct ttctttgcat

133081 catttccaaa aaaatcgata aactgggtgg atatgtaaaa gatattattt gttttccatc

133141 aactggacat acgtgaaaaa aatattgtga catttcattt cttacagcat tttgaaataa

133201 attttttttt atatatctca atggacgatt acatcgttta tagtcgaaaa gaagattcac

133261 aagaggtttt tcaaaccaga agaggtcttt atcttctttc tctttaagta tttctaactt

133321 caaaatttct tgcctctttt ttttttcatg tagttttttt ggatcctccc tttcttccga

133381 acaaagggaa gggtcttctt cggtggatcc ctcttgttcc tgtttagtcc ccttcgtttc

133441 ggaagttttt tctatttcta catctgtttc ttcctcactt tccccccttt cttccgtttt

133501 tgaggtttct ttcagtttct tagtgacaat aggcgacggt attctgccta aatagtagac

133561 acaggtgata aataagagaa tagtaaagat tcgagccata gaatttctca attctgacac

133621 aaggtactta ttagatcgaa taagtacatt cgatctaata gaatgatttt gccgtatcca

133681 gaataatacc aatccaaccc atttcatgaa gaaaatgtga ccaattaacc aaccaacaaa

133741 actacttgtt acaaataaca tcttgttgtt gcatcgaaac atataaatgt tgactaatct

133801 gactaacgtt gaacttggta aaatgaaatg gttgaataat tgaaaaatga gattattcag

133861 gaatacacat tgaatgctga gattacgcat tgaatttctg gtagtagatc cataatccaa

133921 aaagtgtttg tgattgttcc agaagaaatg aaacaaaaga tacggtagaa ctaggacagt

133981 tattgtatga ggtctaccca atgctagatg cagaggcgca taatagatcg atatgaacat

134041 catgagctgt cccgtaataa aaccagttgt tgctgatacc tccttctcgg ttccttcttc

134101 cataatccta gctcggagaa ggaaaagata agagggccct atggagaatg tggtcagaaa

134161 tccataatag agtccgacca caacgaccga atttattatc ttcatgtata aggataatag

134221 attacctagt agaaaagatt tcaaaatcat cacaaacctc ccttttttct tttctattgc

134281 aatttctcga ttactatatg atgatttctt cactttccat agatagaaac agatagacta

134341 gaaatgacat cttttatgtc aatgataccc tcttcttcga ttgaatgaca tctcttatgt

134401 caatgacacc aaagggatat taaatgaatg gaattgggat atggatggaa tattgggata

134461 tggatggaat ataatgaaat agagccactt tgaggttccc tatgaaatga ggcatggaac

134521 ggagccacta cgaagaagtt ccgggagtta cgaaggaagc ttcgggctca tattggtcat

134581 gggttgagag cgggagttga actctatgag atcgaatctc ccgttattcc tcagtagctc

134641 agtggtagag cggttggctg ttaactgact ggtcgtaggt tcgaatccta cttggggaga

134701 tttgatgaat tcttaattaa agaattcaga attacagaat taaagggctc gctttgaccg

134761 ttaggagtag gtaacccgtt ccctgtcttt gtttctattg cactctatct catcgcattc

134821 tgttctgcga tatttgagaa tcgccgtcaa tacctcggtg tagatccggg ataatccttt

134881 gttccatagc cctggggcta tttacaacta gccaattcat aattctcaga tgatgtacta

134941 gcagtgcatc aaagatgcag tcatcgattc tcccgagagg ccacaattac cgcgagcaaa

135001 catattaatg acgaggaacg catttttgct atgctactaa tacttgtatt tgctctgcta

135061 ttcttcccaa gcctggctga ggaagagtta cggggagtaa aacacaaata tgctgattcg

135121 ggccaggcat actatatgta tcgatttttt ccttcacgat aaataaaaag aataaggcca

135181 tttcgacaaa agacccacac cccagttcca tagctttgcg tccgctatcc cgatcatgat

135241 tttcctaccc ccagaggtaa aggaaaggtc cttccctttt gggccggttg tgggcgagga

135301 gggattcgaa cccccgacac cgtggttcgt agccacgtgc tctaatcctc tgagctacag

135361 gccccacccc gtctccactg gatctgttcc ctggagtacc ctcaaaaagg aaccttttct

135421 cccctcagcc atttcgggtt aagaagacgt gaaagcgcct ttctctctat aagaacagcg

135481 cgttccgagg tgtgaagtgg gagagaaggg atgtcataat tgggttttga ataagacgac

135541 cttttcattt ttttcttttt gagtaataag aataagaggt gttaagcttt ttatcatcct

135601 ggcgtcgagc tatttttccg caggacctcc cctacagtat cgtcaccgca gtagagttta

135661 accaccaagt tccgggatgg attggtgtgg ttcctctacg cctaggacac cagaatatcg

135721 aaccatgaac gaagaaaggt atgagagaaa tattggctag tgattgtgaa gcccaaattc

135781 ttgactggaa gggacaccaa aggcctctgc ccttccatcc cttggataga tagagaggga

135841 gggcagagct tttggttttt tcatgttgtc aaacagttga acaatgaaaa tagatggcga

135901 gtgcctgatc gaattgatcg ggtcatgtag gaacaaggtt caaatctctc ggtctgttag

135961 gatgcctcag ctgcatacat cactgcactt ccgcttgaca cctatcgtaa tgataaacgg

136021 ctcgtctcgc cgtgacctta tcttggattc tcaagacttc tgtcgctcca tccccgcagg

136081 ggcagagaac ccgtcgctgt ctcggctgtg ctaccggagg ctctggggaa gtcggaatag

136141 gagagcactc atcttggggt gggcttacta cttagatgct ttcagcagtt atccgctccg

136201 cacttggcta cccagcgttt accgtgggca cgataactgg tacaccagag gtgcgtcctt

136261 cccggtcctc tcgtactagg gaaaggtcct ctcaatgccc taacgcccac accggatatg

136321 gaccgaactg tctcacgacg ttctgaaccc agctcacgta ccgctttaat gggcgaacag

136381 cccaaccctt ggaacatact acagccccag gtggcgaaga gccgacatcg aggtgccaaa

136441 ccttcccgtc gatgtgaact cttggggaag atcagcctgt tatccctaga gtaactttta

136501 tccgttgagc gacggccctt ccactcgaca ccgtcggatc actaaggccg actttcgtcc

136561 ctgctcgacg ggcgggtctt gcagtcaagc tcccttctgc ctttgcactc gagggccaat

136621 ctccgtccgg cccgaggaaa cttttgcacg cctccgttac cttttgggag gcctacgccc

136681 catagaaact gtctacctga gactgtccct tggcccgtag gtcctgacac aaggttagaa

136741 ttctagctct tccagagtgg tatctcactg atggctcggg cccccccgga aggaggcctt

136801 cttcgccctc cacctaagct gcgcaggaaa ggcccaaagc caatcccagg gaacagtaaa

136861 gcttcatagg gtctttctgt ccaggtgcag gtagtccgca tcttcacaga catgtctatt

136921 tcaccgagcc tctctccgag acagtgccca gatcgttacg cctttcgtgc gggtcggaac

136981 ttacccgaca aggaatttcg ctaccttagg accgttatag ttacggccgc cgttcaccgg

137041 ggcttcggtc gccggctccc ctgtcatcag gtcaccaact tccttaacct tccggcactg

137101 ggcaggcgtc agcccccata catggtctta cgactttgcg gagacctgtg tttttggtaa

137161 acagtcgccc gggcctggtc actgcgacct cctttgtgag gaggcacccc ttctcccgaa

137221 gttacggggc tattttgccg agttccttag agagagttgt ctcgtgcccc taggtattct

137281 ctacctaccc acctgtgtcg gtttcgggta caggtaccct tttgttgaag gtccttcgag

137341 cttttcctgg gagtatggca tgagttactt cagcgccgta gcgcctggta ctcggacatt

137401 ggctcggggc atttcctcta ccccttctta ccctgcccta aaaaagcaag gtgaccttgc

137461 gtccttgaac cgataaccat ctttcggcta acctagcctc ctccgtacct cgggaccaac

137521 aaggggtagt acaggaatat tcacctgttg tccatcgact acgcctttcg gcctgatctt

137581 aggccctgac tcaccctccg tggacgaacc ttgcggagga acccttaggt tttcggggca

137641 ttggattctc accaatgttt gcgttactca agccgacatt ctcgcttccg cttcgtccac

137701 acctgctcgc gcgggagctt ccctctaagc ggaacgctcc cctaccgatg catttttaca

137761 tcccacagct tcggcagatc gcttagcccc gttcatcttc ggcgcaagag cactcgatca

137821 gtgagctatt acgcactctt tcaagggtgg ctgcttctag gcaaacctcc tggctgtctc

137881 tgcaccccta cctcctttat tactgagcgg tcatttaggg gccttagctg gtgatccggg

137941 ctgtttccct ctcgacgatg aagcttatcc cccatcgtct cactgtccga acttgacccc

138001 tgttattttg gggtcatatc tagtattcag agtttgcctc gatttggtac cgctctcgcg

138061 gcccgcaccg aaacagcgct ttacccctag atgtccagtc aactgctgcg cctcaacgca

138121 tttcggggag aaccagctag ctctgggttc gagtggcatt tcacccctaa ccacaacaag

138181 cttcatcctg gtcatggata gatcacccag gttcgggtcc ataagcagtg acaattaccc

138241 tatgaagacg cgctttcgct acggctccgg tgggttccct taaccaagcc actgcctaag

138301 agtcgccggc tcattcttca acaggcacgc ggtcagagat ttctcctccc actgcttggg

138361 agctcacggt ttcatgttct atttcactcc ccgatggggg ttcttttcac ctttccctca

138421 cggtactact tcgctatcgg tcacccagga gtatttagcc ttgcaagggg gtccttgctg

138481 attcacacgg gattccacgt gccccatgct actcgggtca gagcgtaagc tagtgatgct

138541 ttcggctact ggactctatc catctagggt gcagcactcc accgcttcgc ctagcagcac

138601 gacgcttgta ttgctctccc acaacctcgt tttcacggtt taggctgctc ccatttcgct

138661 cgccgctact acggaaatcg cttttgcttt cttttcctct ggctactaag atgtttcagt

138721 tcgccaggtt gtctcttgcc tgcccatgga ttcagcagca gttcgaaagg ttgacctatt

138781 cgggaatctc cggatctatg cttattttca acttcccgaa gcatttcgtc gcttactacg

138841 cccttcctcc tctctgggtg cctaggtatc caccgtaagc ctttcctcgt ttgaacctcg

138901 ccattaatgt gttggctatg ccatcctaag gtgctgctaa atggaaggat cttatcaacg

138961 tccatgaatg agaaatcata gatcgaactg ccgaatcgga aaaattgggt gctatcatat

139021 acctttgcat cggctaaggt cacgagctgg agataagcgg actcgaaccg ctgacatccg

139081 ccacagggta aaccaccgcc tctcgggcct ccccgactga ttctatcata gaggccaacg

139141 atagacaata actccccccc gaacacagct tacaactttc atcgtactgt gctctccaaa

139201 gagcaactct tctcaaaatc tcaaagggtg ctgagttgga atcccattct aaggattctt

139261 gtggttccgg aggatccagc tacacgagaa ccaggaacgg ggagctcttc ccctttttcc

139321 gcccgactct ttggtcttaa gaatgctggt tttaagaatg agtgattgcc cttctccgac

139381 ccttactgcc caaccggaga gcggacagct aatgcgttcc acttattgaa cagggtctat

139441 ggtcggtctg tgacccctgg atgccgaagg cgtccttggg gtgatctcgt agttcctacg

139501 gggtggagac gatggggtcg gtccatggat tttccttcct tttgccacat ttcgctcaaa

139561 gggttgaagg gagatagtgc atcaagctgt tcgcaagggc caacttgatc ctcttcccca

139621 gggatccaga tgagggaacc ctaggagagc cgccgactcc aactatcgtc catgtacgat

139681 ccatactaga tctgaccaac tgcccatcct acctcctcta cgttcttgac agcccatctt

139741 tgtctcagta gagtctttca gtggcatgtt tcggtcctct tccccattac ttagaaaaag

139801 tgagccaccg gttcaggtac aagatactat cattaccgcc tggacaatta gacatccaac

139861 ccgtaatcgc aacgacccaa ttgcaagagc ggagctctac caactgagct atatcccccc

139921 gagcccagtg gagcatgcat gaaggagtca gatgcttctt cgattctttt ccctggcgca

139981 gctgggccat cctggacttg aaccagagac ctcgcccgtg aagtaaatca tcgcacctac

140041 gatccaacca attgggagag aatcaataga ttctttttcg ggggcgattc atccttcccg

140101 aacgcagcat acaactctcc gttgtactgc gctctccaag tgtgcttgtt cccaccttct

140161 tccttaccat ggcaagtatt tgtgaaataa ctccgatgag aagaaaaaga aggcgttaag

140221 agaccctcct ggcccaaccc tagacactct aagatccttt ttcaaacctg ctcccatttt

140281 gagtcaagag atagataaat agacacatcc cattgcactg atcggggggc gttcgtagtg

140341 actgaggggg tcgaagacca agaagtgagt tatttatacc aagcattctt cttatggcta

140401 gatccaatct cctggtccct gcggaaagga aaaagaattt cacgttcttc ctttcgggaa

140461 gggaggatta gggaaatcct attgattgct gctttctcca gacctccgcg ggaaaagcat

140521 gaaaaaaaaa ggctcgaatg gtacgatccc tccgtcaccc cagaatgaaa ggggtgatct

140581 cgtagttctt ggtctgtgaa gatgcgttgt taggtgctcc attttcccat tgaggccgaa

140641 cctaaacctg tgctcgagag atagctgtcc atacactgat aagggatgta tggattctcg

140701 agaagagagg agccatggtg gtcccccccg gaccgcccgg atcccacgag tgaatagaaa

140761 gttggatcta cattggatct cacctgaatc gccccatcta tcctcctgag gagaagtttg

140821 gtttcaaact ccggttcgaa caggaggagt acgccatgct aatgtgcctt ggatgatcca

140881 catccccggg tcaggcgctg atgagcacat tgaactatcc atgtggctga gagccctcac

140941 agcccaggca caacgacgca attatcaggg gcgcgctcta ccactgagct aatagccctt

141001 gcgggccccc cacggggagg cccgctatgc caaaagcgag agaaagccca tccctctctt

141061 tcctttttgc gcccccatgt cgccacacgg gaggggcatg gggacgtaaa aaaggggatc

141121 ctatcaactt gttccgacct aggataataa gctcatgagc ttgtcttact tcaccgtcga

141181 gaaacgaaag aagacttcca tctccaagct tagctcagac gtagctcgct tctttagctc

141241 tccctgaaaa ggaggtgatc cagccgcacc ttccagtacg gctaccttgt tacgacttca

141301 ctccagtcac tagccccgcc ttcggcatcc ccctccttgc ggttaaggta acgacttcgg

141361 gcatagccag ctcccacagt gtgacgggcg gtgtgtacaa ggcccgggaa cgaattcacc

141421 gccgtatggc tgaccggcga ttactagcga ttccggcttc atgcaggcga gttgcagcct

141481 gcaatccgaa cttaggacgg gtttttggag ttagctcacc ctggcgggat cgcgaccctt

141541 tgtcccggcc attgtaacac gtgtgtcgcc cagggcatag ggggcatgat gacttgacgt

141601 catcctcacc ttcctccggc ttatcaccgg cggtctgttc agggttccaa actcaatggt

141661 ggcaactaaa cacgagggtt gcgctcgttg cgggacttaa cccaacacct tacggcacga

141721 gctgacgaca gccatgcacc acctgtgtcc gcgttcccga aggcacccct ctctttcaag

141781 aggattcacg gcatgtcaag ccctggtaag gttcttcgct ttgcatcgaa ttaaaccaca

141841 tgctccaccg cttgtgcggg cccccgtcaa ttcctttgag tttcattctt gcgaacgtac

141901 tccccaggcg ggatacttaa cgcgttagct acagcactgc acgggtcgag tcgcacagca

141961 cctagtatcc atcgtttacg gctaggacta ctggggtatc taatcccatt cgctccccta

142021 gctttcgtct ctcagtgtca gtgtcggccc agcagagtgc tttcgccgtt ggtgttcttt

142081 ccgatctcaa cgcatttcac cgctccaccg gaaattccct ctgcccctac cgtactccag

142141 cttggtagtt tccaccgcct gtccagggtt gagccctggg atttgacggc ggacttgaaa

142201 agccacctac agacgcttta cgcccaatca ttccggataa cgcttgcatc ctctgtctta

142261 ccgcggctgc tggcacagag ttagccgatg cttattcctc agataccgtc attgcttctt

142321 ctccgagaaa agaagttcac gacccgtggg ccttccacct ccacgcggca ttgctccgtc

142381 aggcggccgt gtctcagtcc cagtgtggct gatcatcctc tcggaccagc tactgatcat

142441 cgccttggta agctattgcc tcaccaacta gctaatcaga cgcaagcccc tcctcgggca

142501 gattcctcct tttgctcctc agcctacggg gtattagcaa ccgtttccag ttgttgttcc

142561 cctcccaagg gcaggttctt acgcgttact cacccgtccg ccactggaaa caccacttcc

142621 cgtccgactt gcatgtgtta agcatgccgc cagcgttcat cctgagccag gatcgaactc

142681 tccatgagat tcatagttgc attacttacc aatcttccgg ttcgtagaca aagctgattc

142741 ggaattgtct ttcattccaa ggcttgtatc catgcgcttc atattagcct ggagttcgct

142801 cccagcaata tagccatccc taccctctca cgtcaatccc acgagcctct tatccattct

142861 cattcgatca cggcggggga gcaagtcaaa atagaaaaac tcacattgcg ttgggtttag

142921 ggataatcag gctcgaactg atgacttcca ccacgtcaag gtgacactct accgctgagt

142981 tatatccctt ccctgccccc atcgagaaat agaactgact aatcctaagg caaagggtcg

143041 agaaactcaa cgccactctt cctgaacaac tcggagccgg gacttctttt cgcactatta

143101 cggatacgaa aagaatggaa aaattggatt caattgtcaa ctgctcctat cggaaatagg

143161 attgactacg gattcgagcc atagcacatg gtttcataaa atccgtacga ttttcccgat

143221 ctaaatcgag caggttttac aggaagaaga ttttgttcag catgttctat tcgatactgg

143281 taggagaaga acccgactcg gtattgttaa aaaaagagag gaagcagaac caagtcaaga

143341 tgatacggat caaccccttc ttcttgcgcc aaagatctta ccatttccga aggaactgga

143401 gctacatctc ttttcaattt ccattcaaga gttcttatgt gtttccacgc ccttttgaga

143461 cctcgaaaaa gggacaaatt ccttttctta ggttcttagg aacacataca agattcgtca

143521 ctacaaaaag gataatggta accccaacat taactacttc atttctgaat ttaatagtaa

143581 tagaaataca tgtcctaccg agacagaatt tgtaacttgc tatcctcttg cctagcaggc

143641 aaagattgac ctccgtggaa agactgattc attcggatcg acatgagggt ccaactacat

143701 tgcattgcca gaatccattt gtatatttga aacaggttga cctccttgct tctctcatgg

143761 tacaatcctc ttcccgctga gccccccttt ctcctcggtc cacagagaca aaatgtagga

143821 ctggtgccaa cagttcatca cggaagaaag gactcaccga gccgggatca ctaactaata

143881 ctaatataat agaaaagaac tgtcttttct gtatactttc ccggctccgt tgctaccgcg

143941 ggccttacgc aatcgatcgg atcatataga tatcccttca acacaacata ggtcatcgaa

144001 aggatctcgg acgacccgcc aaagcacgaa agccaggatc tttcagaaaa tggattccta

144061 ttcgaagagt gcataaccgc atggataagc tcacaccaac ccgtcaattt gggatccaat

144121 tcgggatttt ccttgggagg tatcgggaag gaattggaat gtaataatat cgattcatac

144181 agaagaaaag gttctctatt gattcaaacg ctgtacctat gggatagagg aagaggaaaa

144241 aaccgaagat ttcacatagt acttttgatc gaaaaatcaa tcggatttat ttcgtacccc

144301 tcgttcgatg agaaaatggg tcagattcta caggatcaaa cctatgggac ttaaggaatg

144361 atggaaggga ataaaaagaa aagagaggga aagaaaatcg aaataaagaa tcaaagaaaa

144421 taaataaata aaaaatacaa aaataaataa agattccaaa tgaacaaatt caaactcaaa

144481 aaggatcttt ctgattctcg aagaatgagg gcaaagggat tgatcgagaa agatctcttg

144541 ttcttattat aagatcgtga tttgatccgc atattttggt aaaaagaata atcttctcct

144601 ttgatcataa tcaaaaatgg aaagtgttca attggaacat gagaacgtga ctgaattggt

144661 cctagttact cttcgggacg gagtggaaga agggaggaga ttctcgaacg aggaaaagga

144721 cccaattact tcgaaagaat tgaacgagga gccgtatgag gtgaaaatct catgtacggt

144781 tctgtagagt gacagtaagg gtgacttatc tgtcaacttt tccactatca cccccaaaaa

144841 accaaactct gccttacgta aagttgccag agtacgatta acctctggat ttgaaatcac

144901 tgcttatata cctggtattg gccataattc acaagaacat tctgtagtct tagtaagagg

144961 aggaagggtt aaggatttac ccggtgtgag atatcacatt gttcgaggaa ccttagatgc

145021 tgtcggagta aaggagatga tgccatgtga atcgctagaa acatgtgaag tgtatggcta

145081 acccaatcac gaaagtttcg taaggggact ggagcaggct accatgagac aaaagatctt

145141 ctttctaaag agattcgatt cggaactatt atatgtccaa ggttcaatat tgaaatcatt

145201 tcagaggttt tcccttactt tgtccgtgtc aacaaacaat tcgaaatacc tcgacttttt

145261 cagaacaggt ccgagtcaaa tagcaatgat tcgaagcact tctttttaca ctatttcgga

145321 aacccaagga ctcgatcgta tggatatgta aaatacagga tttccaatcc tagcaggaaa

145381 aggagggaaa cggctactca atttaaagtg agtaaacaga attccatact cgatttcata

145441 gatacatatc aaattctgtg gaaagccgta ttcgatgaaa gtcgtatgta cggcttggag

145501 ggagatcttt catatctttc gagatccacc ctacaatatg gggtcaaaaa gccaaaataa

145561 gtgattcgtt tttagcccgt ataaaaagaa aacggattct tgaacctctt tcacgctcat

145621 gtcacgtcga ggtactgcag aagaaaaaac tgcaaaatcc gatccaattt atcgtaatcg

145681 attagttaac atgttggtta accgtattct gaaacacgga aaaaaatcat tggcttatca

145741 aattatctat caagccgtga aaaagattca acaaaagaca gaaacaaatc cactatctgt

145801 tttacgtcaa gcaatacgtg gagtaactcc cgatatagca gtcaaagcaa gacgtgtagg

145861 tggatcgact catcaagttc ctattgaaat agaatctaca caaggaaaag cacttgccat

145921 tcgttggtta ttaggggcat cccgaaaacg tccgggtcga aatatggctt tcaaattaag

145981 ttccgaatta gtggatgctg ccaaagggag tggcgatgcc atacgcaaaa aggaagagac

146041 tcatagaatg gcagaggcaa atagagcttt tgcacatttt cgttaatcca tgaacaggat

146101 ctatatagac acatagatcc gtggatccat acatctcgat cggaaaagaa tcaatagaaa

146161 aagaaagaat tggaatggat cgatatcttt ctcgaaacaa acgaaaagga aaagaaagat

146221 gaaacataaa tcatggatca actaagccct ctcgggggct ttcttaagaa taagaaggag

146281 gaatctcatg gaaataccat ggaataaggt ttgatcctgt tcatggggat tccgtaaata

146341 tcccattcca aaaatcgaaa gttcgaaaca attgggactt tttcggagat tggatgcagt

146401 tactaattca ggatctggca tgtacagaat gaaaacttca ttctcgattc tacgagaatt

146461 tttatgaaag cgtttcattt gcttctcttc catggaagtt tcattttccc agaatgtatc

146521 ctcatttttg gcctaattct tcttctgatg atcgattcaa cctctgatca aaaagatata

146581 ccttggttat atttcatctc ttcaacaagt ttagtaatga gcataacggc cctattgttc

146641 cgatggagag aagaacctat gattagcttt tcgggaaatt tccaaacgaa caatttcaac

146701 gaaatctttc aatttcttat tttactatgt tcaactctat gtattcctct atccgtagag

146761 tacattgaat gtacagaaat ggctataaca gagtttttgt tattcgtatt aacagctact

146821 ctaggaggaa tgtttttatg tggtgctaac gatttaataa ctatctttgt agctccagaa

146881 tctttcagtt tatgctccta cctattatct ggatatacca agagagatgt acggtctaat

146941 gaggctacta cgaaatattt actcatgggt ggggcaagct cttctattct ggttcatggt

147001 ttctcttggc tatatggttc atccggggga gagatcgagc ttcaagaaat agtgaatggt

147061 cttatcaata cacaaatgta taactcccca ggaatttcca ttgcgcttat atccatcact

147121 gtaggaattg ggttcaagct ttccccagcc ccttctcatc aatggactcc tgacgtatac

147181 gaaggagtgc ggttcgttcg acaaattcct acctctatat ctatctctga gatgtttgga

147241 tttttcaaaa ctccatggac atgcagaaga gaaatgctat ccccactcgg accaagacat

147301 aacttttacc aaaagtttat tgtgatcttt ttgttcaaat aacaattaag gtgaagcagg

147361 gtcaggaaca acgaatctct ttatgataaa cagatccatt ttttcctaca aaggatcgga

147421 ctaatgacgt atacaatact tgaattatcg atgtagatgc tacatagttg gttctcatcc

147481 ttcagagact acgagtgtaa taggagcatc cgtcgacaaa aggatcaccc taagatgatc

147541 atctcatggc tattgagaac gaatcaaatc agatggttct atttctcaat ctttctgact

147601 tgtgctccta cggaaccggg gtcgaaaaga ttgaaaaagt cagtcattca caaccactga

147661 tgaaggattc ctcgaaaagt taaggattag taatcctttt tagaaatcga atggattcgg

147721 tcttatacat acgcgaggaa ggtaatcatt cttttatcac ttaggagccg tgcgagatga

147781 aagtctcatg cacggttttg aatgagagaa agaagtgagg aatcctcttt tcgactctga

147841 ctctcccact ccagtcgttg cttttctttc tgttacttcg aaagtagctg cttcagcttc

147901 agccactcga attttcgata ttccttttta tttctcatca aacgaatggc atcttcttct

147961 ggaaatccta gctattctta gcatgatatt ggggaatctc attgctatta ctcaaacaag

148021 catgaaacgt atgcttgcat attcgtccat aggtcaaatc ggatatgtaa ttattggaat

148081 aattgttgga gactcaaatg atggatatgc aagcatgata acttatatgc tgttctatat

148141 ctccatgaat ctaggaactt ttgctcgcat tgtctcattt ggtctacgta ccggaactga

148201 taacattcga gattatgcag gattatacac gaaagatcct tttttggctc tctcttcagc

148261 cctatgtctc ttatccctag gaggtcttcc tccactagca ggttttttcg gaaaactcca

148321 tctattctgg tgtggatggc aggcaggcct atatttcttg gtttcaatag gactccttac

148381 gagcgttgtt tctatctact attatctaaa aataatcaag ttattaatga ctggacgaaa

148441 ccaagaaata acccctcacg tgcgaaatta tagaagatcc cccttaagat caaacaattc

148501 catcgaattg agtatgattg tatgtgtgat agcatctact ataccaggaa tatcaatgaa

148561 tccaattatt gcaattgctc aggataccct cttttagctt ctagggtcta tttcttagtt

148621 caagatccct tttactaact ggaataaaag aattagtaga tctgttccgc ccaaaatggg

148681 aatgggctgg ggttatgaac ttataatcat ggaatcgact cgatcatcag attataagtt

148741 cattccatac cggaccagac cggaataggg ttatgtacat tctcattatg agaaggggtc

148801 attcgagcat atgtaaatag agactatgtt tacatatgga tccctacgtc gttacattcc

148861 atttaggatt aggaataggc gtaatcggac ctgcttttta catatctatc gttatttggg

148921 taccatatta acttctttgg gcttcgattg aatcgagaaa taggtttgat tgtacatctt

148981 tttgatatat ataaggtatc ctccggataa ttcaaatcga agcaatttga tgtccgactc

149041 gggcctatat gacatgaccg atcgatagaa atactccaaa actccacctt tgtcatatat

149101 tccatatatc acactagata gatatcatat tcatggaata cgattcactt tcaagatgcc

149161 ttgatggtga aatggtagac acgcgagact caaaatctcg tgctaaagag cgtggaggtt

149221 cgagtcctct tcaaggcata atattgaaat gctcattgaa tgagcaattc aataacagat

149281 ctcggatcta atcaatattg atatacatat accaagtatc tgttgatacg aagtattcca

149341 tcgatcccca cgatccgagt ccgggctgtt ggaattggaa taggttcggt tctctatcta

149401 atgaatgagg agtccgtttt gaaatcgtcc gccctgcacc caccccccga gtatatgctt

149461 caacaggaat cacacaaggg taaattgata caatagaaac ctctggtaaa atgcccgccc

149521 gtaacccaac agataaagta catagtccgt tttagcctgt tacatgaatc aaatgtttca

149581 tttcatccgg gaaaagccat ctctttctca acaataactc tcggatggag tattagaacg

149641 gaaagatcca ttagataatg aactattggt tcgaagccat ctctggcgat gaatcaacaa

149701 ttcgaagtgc ttttcttgcg tattcttgat gaaccagcgt ttatatatag atgtaggagg

149761 atttgtttgg gaagtaataa gcccttttga catctcttca tctgcaaaga attctcggcg

149821 tgaaaacaca gagacaaagg gctgatcttt gaataggaaa aagaatggat ctgcagggtc

149881 ccaaatgaat tggcttattc gaaaaaagcc ttgttctttg gaagatctat ctcgtgtctg

149941 gtactgcatg gttccactct gcaagaactc cgaatcattc tcttgaagct catcctcttt

150001 atgataaatg atccgcttgc cccgaaatga cctggcccaa tagggaaatc ccaattcatt

150061 ggtcctttcg atacaatcaa atagattgcc acaagggcgc catattctag gagcccaaac

150121 tatgtgattg aataaatcct cctctatctg ttgcgggtcg agggctcctt ctctttcccc

150181 ttcttcaaac tccgattcgt atttttcata tagaaatctc tgatcaacga tagaacaaga

150241 tccgttttgc atcatatcta actgattcct tggttcggac cgaagaagca atgtcactcg

150301 atcattatca aactgactgc aatctttttc tgtccgtgag gatcccaaca gagcgccttc

150361 gacttctaat aggccatgaa ctagatcaga atcattctca acgaatccat aagaagtgat

150421 ccaatttttt tcatcgggtc cgggtggaga ccaaagatct tgagcgaccg atccggcaga

150481 acaactcaaa agataaagaa gtatcgttaa tttcttcatg ctcgttccaa gttcgaagta

150541 ccatttgtac aaataagaat ccccttcctt acatgatttc ttcttcatat agatagatat

150601 aggatctatg gggcaattac ttagaagtac attttgtgca acagcccttc ctatctgata

150661 gaaaaggatc ccatgatcct gaaccgatct tacctgggat cgcaaatccc aagtttgtct

150721 atgaagagct gatctaattg taattgattt cttctgtgta atactaattg atagggcctc

150781 attggtaagt gctacaagat ctcgtgcatt ggaacccatg gttatggacc cgaatctgtt

150841 actgttagta tggaacattt tcttttccaa gtgaaatccc ctagtatatg aaagaatgaa

150901 aaagtttatt cggagctatt agagtgggat ccactttttg gggaatatga gtcgaagcaa

150961 taacaagaat atttctagtg gaacatcttt cactatctct ggagagatag ttcactaata

151021 gaccgaggga taagtaattc gactcattca catacagatc atgaatgttt ggaatccata

151081 ttatgcaagg ggacattgct tttgctaatt ctaattgaag ggtggtatca aatcggtcta

151141 ttttcggcgt catatacata gttagcacat tcgtcatagt tagcagctcc gtatcaaggt

151201 catcatcaat atcgtcacta tcatcaatat cgatatcgtc actatcatca atatcgatat

151261 cgtcaataag ataaccttta ggcttgtcat ccaggaactt gttcggaaat accgtaatga

151321 aaggaacata ggagtttgtc gctaggtatt tgaccaaata ggatcgtcca gttcctatag

151381 aacctatcac taaaataccc ctagaagggg atagggctaa gcggagcgaa aagggttttc

151441 catgagatgg gaaatgagaa ctattagccc cacacgaggt ttgtgaataa gtgattgtct

151501 gataatgagc aaggaatatc cgtctttctg ctaaacagga tctattgaac tcataattca

151561 ttagatactt tttatgaatg tcaactaagt atcgtaagta aatggatccc ggttgttcaa

151621 tcatttgata accagagtca ttctttgata aacgatcact atgagtcaga ctcaatagaa

151681 tttgatcaat ccttttttcc gtcgttaagg tggagaactg aaccaagaat tctctttctt

151741 catcatcaat cgaatcagga ttctatttta tcatcaatcc aatcaccgtt cacgtttttt

151801 ctttttctta tcaatgaata gatctcttta cttgtacgac ttagatgtct cgtatttctc

151861 gaaaaagtga ttcgattgat gggatttggt atgatactga tgagatcgat gagattgata

151921 ttcaaatatt tcttcttaga acgtattgat ttgaccccat aagcgggacc accacccaat

151981 agcatgttgc cgccagaagc agaatcccgt atttcttcca gagaatctcc taattgttcc

152041 agagcaacta gaaagagatt ctttaaccag aaagaattca gttcagatgt aggataccta

152101 tccagaagtt ttcgcaactc aatcatgtat gatggaatca tcaaagattt gatcttttct

152161 aactctgtct gtaactcact agaggctcgg aaaacaaaga gaagatgtgt acgaacgaga

152221 tatccagcaa caagaagaag gaaaagaatt gaatagagga actcccaagc atttggtgat

152281 ctcagatgtg tccatatcaa tggaatgggt gactcattat ttcgatgaat catttcttcg

152341 gacagaagaa gattctgtaa acacttactc gaactctcac ttatcagatt ccgttgtgga

152401 agaatcgacc accacttttt ctgaggaatt ggccatgata tatctgatcc atgcatcata

152461 tcatgaaaaa cggacacaaa attttgactg ccacttaggg aatattgaaa gggaatattc

152521 aatatcaaat aaatattgtt ttttaaggtg aaataaagat atttacaccc

//
