# Supplementary material for: The complete chloroplast genome of Cinnamomum camphora and its comparison with related Lauraceae species
Source: PeerJ. 2017 Sep 18;5:e3820. doi: 10.7717/peerj.3820 (PMC5609524; doi:10.7717/peerj.3820)
Supplement: Supplemental Information 5 [file peerj-05-3820-s005.pdf]

```
LOCUS       Cinnamomum            152570 bp    DNA     circular     16-MAY-2017
DEFINITION  Cinnamomum camphora chloroplast, complete genome.
ACCESSION
VERSION
KEYWORDS    .
SOURCE      chloroplast Cinnamomum camphora
  ORGANISM  Cinnamomum camphora
            Eukaryota; Viridiplantae; Streptophyta; Embryophyta;
            Tracheophyta;Spermatophyta; Magnoliophyta; Magnoliidae; Laurales;
            Lauraceae;Cinnamomum.
REFERENCE   1  (bases 1 to 152570)
  AUTHORS   Chen,C.
  TITLE     direct submission
  JOURNAL   Unpublished
REFERENCE   2  (bases 1 to 152570)
  AUTHORS   Chen,C.
  TITLE     Direct Submission
  JOURNAL   Submitted (16-MAY-2017) Nanjing Forestry University, Co-Innovation
            Center for Sustainable Forestry in Southern China, Longpan Street
            No.159, Nanjing, Jiangsu 210037, China
FEATURES             Location/Qualifiers
     source          1..152570
                     /organism="Cinnamomum camphora"
                     /organelle="plastid:chloroplast"
                     /mol_type="genomic DNA"
     CDS             complement(join(100931..100957,101494..101724,
                     73307..73420))
                     /gene="rps12"
                     /trans_splicing
                     /codon_start=1
                     /transl_table=11
                     /product="ribosomal protein S12"
                     /translation="MPTIKQLIRNTRQPIRNVTKSPALRGCPQRRGTCTRVYTITPKK
                     PNSALRKVARVRLTSGFEITAYIPGIGHNSQEHSVVLVRGGRVKDLPGVRYHIVRGTL
                     DAVGVKDRQQGRSQYGVKKPK"
     misc_feature    1..93705
                     /note="LSC"
     gene            complement(30..104)
                     /gene="trnH-GUG"
     tRNA            complement(30..104)
                     /gene="trnH-GUG"
                     /product="tRNA-His"
                     /note="anticodon:GUG"
     gene            complement(499..1560)
                     /gene="psbA"
     CDS             complement(499..1560)
                     /gene="psbA"
```

/codon_start=1
/transl_table=11
/product="photosystem II protein D1"
/translation="MTAILERRESTSLWGRFCNWITSTENRLYIGWFGVLMIPTLLTA
TSVFIIAFIAAPPVDIDGIREPVSGSLLYGNNIISGAIIPTSAAIGLHFYPIWEAASV
DEWLYNGGPYELIVLHFLLGVACYMGREWELSFRLGMRPWIAVAYSAPVAAATAVFLI
YPIGQGSFSDGMPLGISGTFNFMIVFQAEHNILMHPFHMLGVAGVFGGSLFSAMHGSL
VTSSLIRETTENESANAGYRFGQEEETYNIVAAHGYFGRLIFQYASFNNSRSLHFFLA
AWPVVGIWFTALGISTMAFNLNGFNFNQSVVDSQGRVINTWADIINRANLGMEVMHER
NAHNFPLDLAAVEVPSTNG"

gene complement(2082..3626)
/gene="matK"

CDS complement(2082..3626)
/gene="matK"
/codon_start=1
/transl_table=11
/product="maturase K"
/translation="MEELQGYLEMDGFRQQYFLYPFLFQEYIYALAHGHALNGSILYE
PVENLDHDNKSSSLIVKRLITRMHQQNRLIISVNDSNQNRFVGHNNHFDSQMISEGFA
VVVEIPFSLRLVSSLEEKEIAKSHNLRSIHSIFPFFEDKLSHLNHVSDILIPHPIHLE
ILVQTLHSWIQDTPSLHLLRFSLYEYWNSNSLITPKNSISLFSKENQRFFLFLSNSHV
YECEFIFIFLRKQPFHLRSKSFGSFLERTHFYAKIEYLVVVLCNDFQKTLWLFKDPFM
HYVRYQGKSILASRGARLLIKKWKSHLVNFWQCHFYLWSQPARIHIKQLYNHPFYFLG
YLSSVRLNSSVIRSQMLENSFRIDTAIKKFETVVPIIPLIGSLAKAKFCNVSGHPISK
PFRADLSDSEILNRFGRICRNLSHYHSGSSKKQSLYRIKYILRLSCARTLSRKHKSTI
RAFLKRLGSEFLEEFFTEEEQALSLIFPTTSSPSHRSHRERIWYLDIIRINDLVSHLM
IGHEVM"

gene complement(4344..4380)
/gene="trnK-UUU"

tRNA complement(4344..4380)
/gene="trnK-UUU"
/product="tRNA-Lys"
/note="anticodon:UUU"

gene complement(5170..6314)
/gene="rps16"

exon complement(5170..5425)
/gene="rps16"
/number=2

CDS complement(join(5197..5425,6274..6314))
/gene="rps16"
/codon_start=1
/transl_table=11
/product="ribosomal protein S16"
/translation="MVKLRLKRCGRKQRVIYRIVAIDVRSRREGRDLRKVGFYDPIKN
QTYSNVSAILYFLEKGAQPTGTVHDISKKAEVFKEFRINQMKLMK"

exon complement(6274..6314)
/gene="rps16"

/number=1
gene complement(8207..8279)
/gene="trnQ-UUG"
tRNA complement(8207..8279)
/gene="trnQ-UUG"
/product="tRNA-Gln"
/note="anticodon:UUG"
gene 8615..8794
/gene="psbK"
CDS 8615..8794
/gene="psbK"
/codon_start=1
/transl_table=11
/product="photosystem II protein K"
/translation="MLNIFSLICLNSALHSSSFFFAKLPEAYAFFNPIVDVMPVIPVL
FFLLALVWQAAVSFR"
gene 9178..9288
/gene="psbI"
CDS 9178..9288
/gene="psbI"
/codon_start=1
/transl_table=11
/product="photosystem II protein I"
/translation="MLTLKLFVYTVVIFFVSLFIFGFLSNDPGRNPGRDE"
gene complement(9427..9514)
/gene="trnS-GCU"
tRNA complement(9427..9514)
/gene="trnS-GCU"
/product="tRNA-Ser"
/note="anticodon:GCU"
gene 11116..11152
/gene="trnG-UCC"
tRNA 11116..11152
/gene="trnG-UCC"
/product="tRNA-Gly"
/note="anticodon:UCC"
gene 11278..11349
/gene="trnR-UCU"
tRNA 11278..11349
/gene="trnR-UCU"
/product="tRNA-Arg"
/note="anticodon:UCU"
gene complement(11462..12985)
/gene="atpA"
CDS complement(11462..12985)
/gene="atpA"
/codon_start=1

/transl_table=11
/product="ATP synthase CF1 alpha subunit"
/translation="MVTIRADEISNIIRERIEQYNREVKIVNTGTVLQVGDGIARIHG
LDEVMAGELVEFEEGTIGIALNLESNNVGVVLMGDGLMIQEGSSVKATGRIAQIPVSE
AYLGRVINALAKPIDGRGEISASESRLIESPAPGIISRRSVYEPLQTGLIAIDSMIPI
GRGQRELIIGDRQTGKTAVATDTILNQKGQNVICVYVAIGQKASSVAQVVTTFQERGA
MEYTIVVAETADSPATLQYLAPYTGAALAEYFMYRERHTSIIYDDLSKQAQAYRQMSL
LLRRPPGREAYPGDVFYLHSRLLERAAKSSSRLGEGSMTALPIVETQSGDVSAYIPTN
VISITDGQIFLSADLFNAGIRPAINVGISVSRVGSAAQIKAMKQVAGKSKLELAQFAE
LEAFAQFASDLDKATQNQLARGQRLRELLKQSQSAPLTVEEQIVTIYTGANGYLDPLE
IGQVKKFLVQLRTYLKTNKPQVQEIISSTKTFTAQAEALLKEAIPEQIELFLLQEQK"

gene complement(13055..14335)
/gene="atpF"

CDS complement(join(13055..13465,14192..14335))
/gene="atpF"
/codon_start=1
/transl_table=11
/product="ATP synthase CF0 subunit I"
/translation="MRDVTDSFVSFGHWPSAGSFGFNTDILATNLINLSVVLGVLIFF
GKGVLSDLLDNRKQRILSTIRNSEELREGAIEQLEEARARLRKVEIEADEFRVNGYSE
IEREKWNLINATYENLERLENYKNETIHFEQQRAINQVRQRVFQQALQGALGTLNSCS
NSELHLRTIGANIGMLGAMKEVTD"

exon complement(13055..13465)
/gene="atpF"
/number=2

exon complement(14192..14335)
/gene="atpF"
/number=1

gene complement(14788..15033)
/gene="atpH"

CDS complement(14788..15033)
/gene="atpH"
/codon_start=1
/transl_table=11
/product="ATP synthase CF0 subunit III"
/translation="MNPLISAASVIAAGLAVGLASIGPGVGQGTAAGQAVEGIARQPE
AEGKIRGTLLLSLAFMEALTIYGLVVALALLFANPFV"

gene complement(15698..16441)
/gene="atpI"

CDS complement(15698..16441)
/gene="atpI"
/codon_start=1
/transl_table=11
/product="ATP synthaseCF0 subunit IV"
/translation="MNVLPCSINSLKALYDISDVEVGQHFYWQIGGFQVHAQVLITSW
VVIAILLGSATIAVRNPQTIPTDGQNFFEYVLEFIRDLSKTQIGEEYGPWVPFIGTMF
LFIFVSNWSGALLPRKIIQLPHGELAAPTNDINTTVALALPTSVAYFYAGLTKKGLGY

FGKYIQPTPILLPINILEDFTKPLSLSFRLFGNILADELVVVVLVSLVPSVVPIPVMF
LGLFTSGIQALIFATLAAAYIGESMEGHH"

gene complement(16649..17368)
/gene="rps2"

CDS complement(16658..17368)
/gene="rps2"
/codon_start=1
/transl_table=11
/product="ribosomal protein S2"
/translation="MPRRYWNMNFEEMVKAGVHFGHGTRKWNPRMAPYISSKRKGIHI
TNLTRTARFLSEACDLVFDAASIGKHFLIVGTKNKAANLVASAAIRARCHYVNKKWLG
GMLTNWSTTEMRLHRFRNLRSEQNTGKLNCLPKRDVAMLKRQLSHLQTYLGGIKYMTG
LPDIVIIVGQQEEYTALRECLTLRIPTICLIDTNCDPDLANIPIPANDDAMASIRWIL
NKLVSAICEGSSSYIRNC"

gene complement(17541..21707)
/gene="rpoC2"

CDS complement(17541..21698)
/gene="rpoC2"
/codon_start=1
/transl_table=11
/product="RNA polymerase beta' subunit"
/translation="MAEWADLFFYNKAIDGSAMKRLISRLIDHFGMAYTSHTLDQVKT
LGFQQATATSISLGIDDLLTVPSKGWLVQDAEQQSFILEKHHHYGNVHTVEKLRQSIE
IWYATSEYLRQEMHPNFRMTDPSNSVHIMSFSGARGNASQVHQLVGMRGLMSDPQGQM
IDLPIQSNLREGLSLTEYIISCYGARKGVVDTAVRTSDAGYLTRRLVEVVQHIVVRRT
DCGTTRGISVSPRNGMTERIWVQTLIGRVLAYNIYMGPRCIAAQNKDIGVGLVTRFIT
FRTQPIYIRTPFLCRSISWICRLCYGGQSPTHGDLVELGEAVGIIAGQSIGEPGTQLTL
RTFHTGGVFTGGTAEHVRAPFKGKIKFNEDLVHPTRTRHGHPAFLCYIDLYVTIESHD
ILHNVNIPPKSFLLVQNDQYVESEQVIAEIRAGTSTFNFNKVKEKVRKHIYSDSEGEM
HWSTDVYHAPEYKYGNVHLLPKTSHLWILSGALCRSSIVPFSLHKDQDQMNVHSVERR
SISDLSVTNDQVRHKLFSSNPYGKKGGVLDYSGPDRIISNGHWNFIYPTILHENSDLL
AKRRRNRFIIPFQSDQEREKELMPRSGISIEIPINGVLPRNSILAYFDDPLYRRSSSG
ITKYGTIGVGSIVKKEDLIEYRRAKEFRPKYQMKVDRFFFIPEEVHILPGSSPIMVRN
NSIIGVDTRIALNTRSQVGGLVRVERKKKRIELKIFSGDIYFPGATDKISRHCGILIP
PGTGKKNSKESKKLKNWIYVQRITPTKKKYFVSVRPVVTYEIADGINLATLFPQDPLQ
ERDNVQLRIVNYILYGNGKPIRGIYHTSLQLVRTCLVLNWNQDRDGSIEEVHASFVEV
RANDLIRDFIRIDLVKSSIFYIGKRNDMASSGLIANNGSDRTNINPFYFKARIQSFTQ
HQGTIRTLLNRNKECPSFLILLSSDCSRIGLFNGSKSHKELIKLIKEDPAIPIRNSLG
PLGIVPQITNFYSFYYFYLITHNQILLKKYFLLDNFKHTFQGLKYYLMDENGRIYNPD
SCSNIIFNPFDLNWCFLPHDYCEETSTIISLGQFICENVCISKCGPHIKSSQVLIVHV
DSLVIRSAKPHLATPGATVHGHCGEILYEGDTLVTFIYEKSRSGDITQGLPKVEQVLE
ARSIDSISMNLEKRVEGWNERITRILGIPWGFLIGAELTIAQSRISLVNKIQKVYRSQ
GVQIHNRHIEIIVRQITSKVLVSEDGMSNVFSPGELIGLLRAERTGRALEEAICYRAI
LLGITRASLNTQSFISEASFQETARVLAKAALRGRIDWLKGLKENVVLGGMMPVGTGF
KGLVHRSRQHSNIPLEIKKKNPFEEEMRDLLFHHRELFGSCIPNNFPDTSERSFTGFN
DRFILFF"

gene complement(21860..24630)

/gene="rpoC1"
CDS complement(join(21860..23479,24199..24630))
/gene="rpoC1"
/codon_start=1
/transl_table=11
/product="RNA polymerase beta"
/translation="MIDRYKHQQLRVGSVSPQQISAWATKILPNGEMVGEVTKPYTFH
YKSNKPEKDGLFCERIFGPIKSGICACGNYRVIGEEKEDPKFCEQCGVEFVDSRIRRY
QMGYIKLACPATHVWYLKRLPSYIANLLDKPLKELEGLVYCDFSFARPIAKKPTFLRL
RGSFESEIQSRKYSIPLFFTTPGFDTFRNREISTGAGAIREQLADPDLRIIIDHSSVE
WKELGEEGFTGNEWEDRKIGRRKDFLVRRMELAKHFIRTNVEPERMVLCLLPVLPPEL
RPIIQIDGGKLMSSDINELYRRVIYRNNTLTDSLTTSRSTPGELVMCQEKLVQEAVDT
LLDNGIRGQPMRDGHNKVYKSFSDVIEGKEGRFRETLLGKRVDYSGRSVIVVGPSLSL
HRCGLPREIAIELFQTFVIRGLIRRHIASNIGIAKSQIREKEPIVWEILQEVMQGHPV
LLNRAPTLHRLGIQAFQPILGGGRAICLHPLVRKGFNADFDGDQMAVHVPLSLEAQAE
ARLLMFSHMNLFSPAIGDPISVPTQDMLIGLYVLTIGNRRGICANRYNPWNRINYQNE
TVNDYKYKYTTKEKEPYFCSSYDVLIVYQQKRINLDSPLWLRWRLDQRVIASREVPVE
VQYESLGTYHEIYGHYLIVRRIKKQTLCIYTRTTVGPISFSREIEEAIQGFCQAYSYG
T"
exon complement(21860..23488)
/gene="rpoC1"
/number=2
exon complement(24184..24630)
/gene="rpoC1"
/number=1
gene complement(24657..27839)
/gene="rpoB"
CDS complement(24657..27839)
/gene="rpoB"
/codon_start=1
/transl_table=11
/product="RNA polymerase beta subunit"
/translation="MYTIPGFSQIQFDGFCRFIDQGLMEELHKFPKIEDTDQEIEFQL
FVATYQLAEPLIKERDAVYESLTYSSELYVSAGLIWKTGRDMQEQTVFIGNIPLMNSL
GTSLVSGIYRIVINQILQSPGIYYRSELDHSGISVYTGTIISDWGGRSELEIDRKARI
WARVSRKQKISILVPSSAMGSNLREILDNVCYPEIFLSFPNHKEKKKIGSRENAILEF
YQQFACVGGDPVFSESLCKELQKKFFQQRCELGRIGRRNMNRRLNLDIPPNNTFLLPQ
DVLAAVDHLIGMKFGMGTLDDMNHLKNKRIRSVADLLQDQFGLALVRLENAVRGTICG
AIRHKLIPTPHNLVTPTPLTTTYESFFGLHPLSQVLDRTNPLTQIVHGRKLSYLGPGG
LTGRTASFRIRDIHPSHYGRICPIDTSEGINVGLIGSLAIHARIGHWGSIESPFYEIS
ERSKKMVYLSPSRDEYYMVAAGNSLALNRGIQEEQVVPAGYRQEFLTIAWEQIHLRSI
FPFQYFSIGASLIPFIEHNDANRALMSSNMQRQAVPLSRSEKCIVGTGLECQAALDSG
VSAIAEHEGKIIYTDTDKIVLSGNGDTISIPLVMYQRSNKNTCMHQKPQAPRGKCIKK
GQILADGAATVGGELTLGKNVLVAHMSWEGYNSEDAVLISERLVYGDIYTSFHIRKYE
IQTHVTSQGPERITNKIPHLEAHLLRNLDKNGIVMLGSWIERGDILVGKLTPQAAKES
SYAPEDRLLRAILGIQVSTAKETCLKLPIGGRGRVIDVRWIQKKGGSSYNPETIRVYI
LQKREIKVGDKIAGRHGNKGIVSKILPRQDMPYLQAGTPVDMVFNPLGVPSRMNVGQI

```
                     FECSLGLAGYLLDRHYRIAPFDERYEQGASRKLVFPELYSASKQTANPWVFEPEYPGK
                     SRILDGRTGDPFEQPVIIGKSYILKLIHQVDDKIHGRSSGHYALVTQQPLRGRAKQGG
                     QRVGEMEVWALEGFGVAHISQEMLTYKSDHIRARQEVLGTTIIGGTILKPEDAPESFR
                     LLVRELRSLALELNHFLVSEKNFQINRKEV"
     gene            28984..29064
                     /gene="trnC-GCA"
     tRNA            28984..29064
                     /gene="trnC-GCA"
                     /product="tRNA-Cys"
                     /note="anticodon:GCA"
     gene            30111..30200
                     /gene="petN"
     CDS             30111..30200
                     /gene="petN"
                     /codon_start=1
                     /transl_table=11
                     /product="cytochrome b6/f complex subunit VIII"
                     /translation="MDIVSLAWAALMVVFTFSLSLVVWGRSGL"
     gene            complement(31265..31369)
                     /gene="psbM"
     CDS             complement(31265..31369)
                     /gene="psbM"
                     /codon_start=1
                     /transl_table=11
                     /product="photosystem II protein M"
                     /translation="MEVNILAFIAIALFILVPTAFLLIIYVKTVSQSD"
     gene            complement(32329..32402)
                     /gene="trnD-GUC"
     tRNA            complement(32329..32402)
                     /gene="trnD-GUC"
                     /product="tRNA-Asp"
                     /note="anticodon:GUC"
     gene            complement(32716..32799)
                     /gene="trnY-GUA"
     tRNA            complement(32716..32799)
                     /gene="trnY-GUA"
                     /product="tRNA-Tyr"
                     /note="anticodon:GUA"
     gene            complement(32854..32926)
                     /gene="trnE-UUC"
     tRNA            complement(32854..32926)
                     /gene="trnE-UUC"
                     /product="tRNA-Glu"
                     /note="anticodon:UUC"
     gene            33650..33721
                     /gene="trnT-GGU"
     tRNA            33650..33721
```

```
                     /gene="trnT-GGU"
                     /product="tRNA-Thr"
                     /note="anticodon:GGU"
gene                 35168..36229
                     /gene="psbD"
CDS                  35168..36229
                     /gene="psbD"
                     /codon_start=1
                     /transl_table=11
                     /product="photosystem II protein D2"
                     /translation="MTIALGRFTKEENDLFDIMDDWLRRDRFVFVGWSGLLLFPCAYF
                     ALGGWFTGTTFVTSWYTHGLASSYLEGCNFLTAAVSTPANSLAHSLLLLWGPEAQGDF
                     TRWCQLGGLWTFVALHGAFGLIGFMLRQFELARSVQLRPYNAIAFSAPIAVFVSVFLI
                     YPLGQSGWFFAPSFGVAAIFRFILFFQGFHNWTLNPFHMMGVAGVLGAALLCAIHGAT
                     VENTLFEDGDGANTFRAFNPTQAEETYSMVTANRFWSQIFGVAFSNKRWLHFFMLFVP
                     VTGLWMSAIGVVGLALNLRAYDFVSQEIRAAEDPEFETFYTKNILLNEGIRAWMAAQD
                     QPHENLIFPEEVLPRGNAL"
gene                 36177..37598
                     /gene="psbC"
CDS                  36177..37598
                     /gene="psbC"
                     /codon_start=1
                     /transl_table=11
                     /product="photosystemII CP43 chlorophyll apoprotein"
                     /translation="MKILYSLRRFYPVETLFNGTLALAGRDQETTGFAWWAGNARLIN
                     LSGKLLGAHVAHAGLIVFWAGAMNLFEVAHFVPEKPMYEQGLILLPHLATLGWGVGPG
                     GEVIDTFPYFVSGVLHLISSAVLGFGGIYHALLGPETLEESFPFFGYVWKDRNKMTTI
                     LGIHLILLGIGAFLLVLKALYFGGVYDTWAPGGGDVRKISNLTLSPSVIFGYLLKSPF
                     GGEGWIVSVDDLEDIIGGHVWLGSICILGGIWHILTKPFAWARRAFVWSGEAYLSYSL
                     GALSVFGFIACCFVWFNNTAYPSEFYGPTGPEASQAQAFTFLVRDQRLGANVGSAQGP
                     TGLGKYLMRSPTGEVIFGGETMRFWDLRAPWLEPLRGPNGLDLSRLKKDIQPWQERRS
                     AEYMTHAPLGSLNSVGGVATEINAVNYVSPRSWLATSHFVLGFFLFVGHLWHAGRARA
                     AAAGFEKGIDRDFEPVLSMTPLN"
gene                 complement(37813..37905)
                     /gene="trnS-UGA"
tRNA                 complement(37813..37905)
                     /gene="trnS-UGA"
                     /product="tRNA-Ser"
                     /note="anticodon:UGA"
gene                 38227..38415
                     /gene="psbZ"
CDS                  38227..38415
                     /gene="psbZ"
                     /codon_start=1
                     /transl_table=11
                     /product="photosystem II protein Z"
                     /translation="MTIAFQLAVFALIATSSILLISVPVVFASSDGWSSNKNVVFSGT
```

SLWIGLVFLVAILNSLIS"
gene 38691..38761
/gene="trnG-UCC"
tRNA 38691..38761
/gene="trnG-UCC"
/product="tRNA-Gly"
/note="anticodon:UCC"
gene complement(38921..38994)
/gene="trnfM-CAU"
tRNA complement(38921..38994)
/gene="trnfM-CAU"
gene complement(39149..39451)
/gene="rps14"
CDS complement(39149..39451)
/gene="rps14"
/codon_start=1
/transl_table=11
/product="ribosomal protein S14"
/translation="MARKSLIQRERKKQKLEQKYHLIRRSSKKEIGKVSSLSDKWEIH
GKLQSPPRNSTPTRLHRRCFLTGRPRASYRDFRLSGHILHEKVQACLLPGATRSSW"
gene complement(39582..41786)
/gene="psaB"
CDS complement(39582..41786)
/gene="psaB"
/codon_start=1
/transl_table=11
/product="photosystem I P700 apoprotein A2"
/translation="MALRFPKFSQGLAQDPTTRRIWFGIATAHDFESHDDITEERLYQ
NIFASHFGQLAIIFLWTSGNLFHVAWQGNFESWVQDPLHVRPIAHAIWDPHFGQPAVE
AFTRGGALGPVNIAYSGVYQWWYTIGLRTNEDLYTGALFLLFLSAISLIAGWLHLQPK
WKPSVSWFKNAESRLNHHLSGLFGVSSLAWTGHLVHVAIPGSRGQYVRWNNFLDVLPH
PQGLGPLFTGQWNLYAQNPDSSRHLFGTSQGAGTAILTLLGGFHPQTQSLWLTDIAHH
HLAIAFIFLVAGHMYRTNFGIGHSMKDLLEAHIPPGGRLGRGHKGLYDTINNSIHFQL
GLALASLGVITSLVAQHMYSLPAYAFIAQDFTTQAALYTHHQYIAGFIMTGAFAHGAI
FFIRDYNPEQNEDNVLARMLDHKEAIKSHLSWASLFLGFHTLGLYVHNDVMLAFGTPE
KQILIEPIFAQWIQSAHGKTSYGFDVLLSSTNGPAFNAGRSIWLPGWLNAVNENSNSL
FLTIGPGDFLVHHAIALGLHTTTLILVKGALDARGSKLMPDKKDFGYSFPCDGPGRGG
TCDISAWDAFYLAVFWMLNTIGWVTFYWHWKHITLWQGNVSQFNESSTYLMGWLRDYL
WLNSSQLINGYNPFGMNSLSVWAWMFLFGHLVWAIGFMFLISWRGYWQELIETLAWAH
ERTPLANLIRWRDKPVALSIVQARLVGLAHFSVGYIFTYAAFLIASTSGKFG"
gene complement(41812..44064)
/gene="psaA"
CDS complement(41812..44064)
/gene="psaA"
/codon_start=1
/transl_table=11
/product="photosystem I P700 apoprotein A1"

/translation="MIIRSPEPEVKILVDRDPIKTSFEEWARPGHFSRTIAKGPDTTT
WIWNLHADAHDFDSHTSDLEEISRKVFSAHFGQLSIIFLWLSGMYFHGARFSNYEAWL
SDPTHIGPSAQVVWPIVGQEILNGDVGGGFRGIQITSGFFQLWRASGITNELQLYCTA
IGALVFAALMLFAGWFHYHKAAPKLAWFQDVESMLNHHLAGLLGLGSLSWAGHQVHVS
LPINQFLDAGVDPKEIPLPHEFILNRDLLAQLYPSFAEGSTPFFTLNWSKYAEFLSFR
GGLDPVTGGLWLTDIAHHHLAIAILFLVAGHMYRTNWGIGHGLKDILEAHKGPFTGQG
HKGLYEILTTSWHAQLSLNLAMLGSSTIVVAHHMYSMPPYPYLAIDYGTQLSLFTHHM
WIGGFLIVGAAAHAAIFMVRDYDPTTRYNDLLDRVLRHRDAIISHLNWVCIFLGFHSF
GLYIHNDTMSALGRPRDMFSDTAIQLQPIFAQWVQNTHALAPGATAPGATTSTSLTWG
GGDLVAVGGKVALLPIPLGTADFLVHHIHAFTIHVTVLILLKGVLFARSSRLIPDKAN
LGFRFPCDGPGRGGTCQVSAWDHVFLGLFWMYNAISVVIFHFSWKMQSDVWGSISDQG
VVTHITGGNFAQSSITINGWLRDFLWAQASQVIQSYGSSLSAYGLFFLGAHFVWAFSL
MFLFSGRGYWQELIESIVWAHNKLKVAPATQPRALSIVQGRAVGVTHYLLGGIATTWA
FFLARIIAVG"

gene complement(44705..46677)
/gene="ycf3"

CDS complement(join(44705..44857,45589..45816,46552..46677))
/gene="ycf3"
/codon_start=1
/transl_table=11
/product="photosystem I assembly protein ycf3"
/translation="MPRSRINGNFIDKTSSIVANILLRIIPTTSGEKEAFTYYRDGMS
AQSEGNYAEALQNYYEATRLEIDPYDRSYILYNIGLIHTSNGEHTKALEYYFRAIERN
PFLPQAFNNMAVICHYRGEQAIRQGDSEIAEAWSDQAAEYWKQAIALTPGNYIEAHNW
LKIARRFE"

exon complement(44708..44857)
/gene="ycf3"
/number=3

exon complement(45589..45816)
/gene="ycf3"
/number=2

exon complement(46552..46677)
/gene="ycf3"
/number=1

gene 47488..47574
/gene="trnS-GGA"

tRNA 47488..47574
/gene="trnS-GGA"
/product="tRNA-Ser"
/note="anticodon:GGA"

gene complement(47854..48459)
/gene="rps4"

CDS complement(47854..48459)
/gene="rps4"
/codon_start=1
/transl_table=11
/product="ribosomal protein S4"

```
                     /translation="MSRYRGPRFKKIRCLGALPGLTSKRPRSGSDLRNQSRFGKRSQY
                     RIRLEEKQKLRFHYGLTERQLLRYVRIAGKAKGSTGQVLLQLLEMRLDNILFRLGMAS
                     TIPGARQLVNHRHILVNGRVVDIPSYRCKPRDIITARDEQRSRALIQNYLDSSPREDL
                     AKHLTFDSSQYKGLVNQIIDIKWIGLKINEFLVVEYYSRQT"
     gene            complement(48799..48871)
                     /gene="trnT-UGU"
     tRNA            complement(48799..48871)
                     /gene="trnT-UGU"
                     /product="tRNA-Thr"
                     /note="anticodon:UGU"
     gene            49420..49454
                     /gene="trnL-UAA"
     tRNA            49420..49454
                     /gene="trnL-UAA"
                     /product="tRNA-Leu"
                     /note="anticodon:UAA"
     gene            49934..49983
                     /gene="trnL-UAA"
     tRNA            49934..49983
                     /gene="trnL-UAA"
                     /product="tRNA-Leu"
                     /note="anticodon:UAA"
     gene            50331..50403
                     /gene="trnF-GAA"
     tRNA            50331..50403
                     /gene="trnF-GAA"
                     /product="tRNA-Phe"
                     /note="anticodon:GAA"
     gene            complement(50935..51411)
                     /gene="ndhJ"
     CDS             complement(50935..51411)
                     /gene="ndhJ"
                     /codon_start=1
                     /transl_table=11
                     /product="NADH-plastoquinone oxidoreductase subunit J"
                     /translation="MQGRSSAWLVKHELVHRSLGFDYQGRETLQIKPEDWYSIAVISY
                     VYGYNYLRFQCAYDVAPGGFLASVYHLTRIQYGVDQPEEVCIKVFVPRRNPRIPSVFW
                     IWKSADFQERESYDMLGISYENHPRLKRILMPESWVGWPLRKDYIAPNFYEIQDAH"
     gene            51511..52368
                     /gene="ndhK"
     CDS             complement(51511..52368)
                     /codon_start=1
                     /transl_table=11
                     /product="NADH-plastoquinone oxidoreductase subunit K"
                     /translation="MGNEFRCIGCICVYRSFNFRAYPNCWFSLCMAKRSIGMVLAPEY
                     SDNQNQKEGKDYIETVMNSIEFPLLDRTAQNSVISTTSNDLSNWSRLSSLWPLLYGTS
                     CCFIEFASLIGSRFDFDRYGLVPRSSPRQADLILTAGTVTMKMAPSLVRLYEQMPEPK
```

YVIAMGACTITGGMFSTDSYSTVRGVDKLIPVDVYLPGCPPKPEAVIDAITKLRKKVS
REIYEDRIGSQQENRYFTTNHKFHVGHSTHTGNYDQGLLYQSPSTSEIPSETFFKYKS
SVSSPKLMN"

```
gene            complement(52248..52610)
                /gene="ndhC"
CDS             complement(52248..52610)
                /gene="ndhC"
                /codon_start=1
                /transl_table=11
                /product="NADH-plastoquinone oxidoreductase subunit 3"
                /translation="MFLLHEYDIFWAFLIISSVIPILAFLISGVLAPISEGPEKLSSY
                ESGIEPMGDAWLQFRIRYYMFALVFVVFDVETVFLYPWAMSFDVLGVSVFIEALIFVL
                IPIVGSVYAWRKGALEWS"
gene            complement(52256..52257)
CDS             complement(52261..52275)
                /gene="ndhC"
                /pseudo
                /codon_start=1
                /transl_table=11
                /product="noproductstringinfile"
gene            complement(54463..54499)
                /gene="trnV-UAC"
tRNA            complement(54463..54499)
                /gene="trnV-UAC"
                /product="tRNA-Val"
                /note="anticodon:UAC"
gene            complement(55087..55125)
                /gene="trnV-UAC"
tRNA            complement(55087..55125)
                /gene="trnV-UAC"
                /product="tRNA-Val"
                /note="anticodon:UAC"
gene            55305..55376
                /gene="trnM-CAU"
tRNA            55305..55376
                /gene="trnM-CAU"
                /product="tRNA-Met"
                /note="anticodon:CAU; tRNA-Met2"
gene            complement(55605..56009)
                /gene="atpE"
CDS             complement(55605..56009)
                /gene="atpE"
                /codon_start=1
                /transl_table=11
                /product="ATP synthase CF1 epsilon subunit"
                /translation="MTLNLCVLTPNRIIWDSEVKEIILSTNSGQIGVLPNHAPIATAV
                DIGILRIRLNDQWLTMAVMGGFARIGNNEITILVNDAEKGSDIDPQEAQRTLEIAEAN
```

LSRAEGKRQAIEANLALRRARTRVEAINVISY"
gene complement(56006..57502)
/gene="atpB"
CDS complement(56006..57502)
/gene="atpB"
/codon_start=1
/transl_table=11
/product="ATP synthase CF1 beta subunit"
/translation="MRINPTNSGPGVSTLEEKNLGRIAQIIGPVLDVAFPPGKMPNIY
NALVVKGRDTVGQQINVTCEVQQLLGNNRVRAVAMSATDGLMRGMEVIDTGAPLSVPV
GGATLGRIFNVLGEPVDNLGPVDTRTTSPIHRSAPAFIQLDTKLSIFETGIKVVDLLA
PYRRGGKIGLFGGAGVGKTVLIMELINNIAKAHGGVSVFGGVGERTREGNDLYMEMKE
SGVINEQNIAESKVALVHGQMNEPPGARMRVGLTALTMAEYFRDVNEQDVLLFIDNIF
RFVQAGSEVSALLGRMPSAVGYQPTLSTEMGSLQERITSTKEGSITSIQAVYVPADDL
TDPAPATTFAHLDATTVLSRGLAAKGIYPAVDPLDSTSTMLQPRIVGEEHYETAQRVK
QTSQRYKELQDIIAILGLDELSEEDRLTVARARKIERFLSQPFFVAEVFTGSPGKYVG
LTETIRGFQLILSGELDGLPEQAFYLVGNIDEATAKAMNLEVESKLKK"
gene 58257..59684
/gene="rbcL"
CDS 58257..59684
/gene="rbcL"
/codon_start=1
/transl_table=11
/product="ribulose-15-bisphosphate carboxylase/oxygenase
large subunit"
/translation="MSPKTETKASVGFKAGVKDYKLTYYTPDYETKDTDILAAFRVTP
QPGVPPEEAGAAVAAESSTGTWTTVWTDGLTSLDRYKGRCYHIEPVPGEETQFIAYVA
YPLDLFEEGSVTNMFTSIVGNVFGFKALRALRLEDLRIPPAYSKTFQGPPHGIQVERD
KLNKYGRPLLGCTIKPKLGLSAKNYGRAVYECLRGGLDFTKDDENVNSQPFMRWRDRF
VFCAEAIYKAQAETGEIKGHYLNATAGTCEEMIKRAVFARELGVPIVMHDYLTGGFTA
NTTLAHYCRDNGLLLHIHRAMHAVIDRQKNHGMHFRVLAKALRMSGGDHVHAGTVVGK
LEGERDITLGFVDLLRDDFIEKDRSRGIYFTQDWVSMPGVLPVASGGIHVWHMPALTE
IFGDDSVLQFGGGTLGHPWGNAPGAVANRVAVEACVQARNEGRDLAREGNEIIREAAK
WSPELAAACEVWKEIKFEFAAMDTL"
gene 60368..61879
/gene="accD"
CDS 60368..61879
/gene="accD"
/codon_start=1
/transl_table=11
/product="Acetyl-CoA carboxylase carboxyltransferase beta
subunit"
/translation="MGKWWFNSMLSNEELEHRCGLGKSMDSLGRPVGNTSGSEDPILN
DTNKNNHNHGWRESNSCSNVDHFFGVRDIWSFISDDTFLVRDSNGNSYSVYFDIENRV
FEIDNDSSFLSELETAFSSYLNSGSKSDNRYYDHYMYDTTYSWNNHINSCIDSYLRSE
ISINKYISSGSDNPIYSYIYSYICSGESVSDSDRGSSSIRTGGNGSDFNIRGRSNDFD
GNKKYRHLWVQCENCYGLNYKKFFRSKMNICEQCGYHLKMGSSDRIELSVDSGTWDPM

DEDMVSIDPIEFHSEEEPYRDRINSYQRKTGLTEAVQTGIGQLNGIPIAIGVMDFQFM
GGSMGSVVGEKITRLIEYAANRSLPVIMVCASGGARMQEGSLSLMQMAKISSALYDYQ
FNKKLFYVSILTSPTTGGVTASFGMLGDIIIAEPNAYIAFAGKRVIEQTLNKTVPDGS
QAAEYLFHKGLFDPIVPRNPLKGVLSELFQLHGFFPLNQNSSRALGSVICSEL"

gene 62465..62575
/gene="psaI"

CDS 62465..62575
/gene="psaI"
/codon_start=1
/transl_table=11
/product="photosystem I subunit VIII"
/translation="MTDFNLPSIFVPLVGLVFPAIAMASLSLHVQKNKIV"

gene 63013..63567
/gene="ycf4"

CDS 63013..63567
/gene="ycf4"
/codon_start=1
/transl_table=11
/product="photosystem I assembly protein ycf4"
/translation="MNYRSERIWIELITGSRKTSNFCWACILFLGSLGFLLVGTSSYL
GRNLISLFPSQQIIFFPQGIVMSFYGIAGLFISSYLWCTISWNVGSGYDRFDRKEGIV
CIFRWGFPGINRRIFLRFLMREIQSIRMEVKEGLYPRRVLYMEIRGQGAIPLTRTDEN
LTPREIEQKAAELAYFLRVPIEVF"

gene 64478..65167
/gene="cemA"

CDS 64478..65167
/gene="cemA"
/codon_start=1
/transl_table=11
/product="chloroplast envelope membrane protein"
/translation="MSKKKALTPLPYLASIVFLPWWISLSFNKSLEPWVTNWWNTGQS
ETFLNDIQEKNVLERFVELEQLFLLDEMIKEYPETQIQKLRIGIHKETMQLVKMHNED
HIHIILDFSTNIICFAILSGYSILGNEELVILNSWVQEFLYNLSDTIKAFSILLLTDL
CIGFHSPRGWELMIGSVYKDFGFAHNDQIISGLVSTFPVILDTILKYWIFHYLNRVSP
SLVVIYHSMNE"

gene 65390..66352
/gene="petA"

CDS 65390..66352
/gene="petA"
/codon_start=1
/transl_table=11
/product="cytochrome f"
/translation="MQNRNTFSWVKKEMTRFISVLIMIYVITRTSISNAYPIFAQQGY
ENPREATGRIVCANCHLANKPVDIEVPQAVLPDTVFEAVVRIPYDMQMKQVLANGKKG
ALNVGAVLILPEGFELAPPDRISPELKEKMGNLSFQSYRPTKRNILVVGPVPGQKYSE
IVFPILSPDPSTKKDVHFLKYPIYVGGNRGRGQIYPDGSKSNNTVYNATAAGIVSRIV
RKEKGGYEISIADASDGHQVVDIIPPGPELLVSEGESIKLDQPLTSNPNVGGFGQGDA

EIVLQDPLRVQGLLFFLASVILAQIFLVLKKKQFEKVQLSEMNF"
gene complement(67516..67638)
/gene="psbJ"
CDS complement(67516..67638)
/gene="psbJ"
/codon_start=1
/transl_table=11
/product="photosystem II protein J"
/translation="MADTTGRIPLWLIGTVTGIPVIGSIGIFFYGSYSGLGSSL"
gene complement(67765..67881)
/gene="psbL"
CDS complement(67765..67881)
/gene="psbL"
/exception="RNA editing"
/codon_start=1
/transl_except=(pos:complement(67879..67881),aa:Met)
/transl_table=11
/product="photosystem II protein L"
/translation="MTQSNPNEQNVELNRTSLYWGLLLIFVLAVLFSNYFFN"
gene complement(67904..68023)
/gene="psbF"
CDS complement(67904..68023)
/gene="psbF"
/codon_start=1
/transl_table=11
/product="photosystem II cytochrome b559 beta subunit"
/translation="MTIDRTYPIFTVRWLAVHGLAVPTVSFLGSISAMQFIQR"
gene complement(68033..68284)
/gene="psbE"
CDS complement(68033..68284)
/gene="psbE"
/codon_start=1
/transl_table=11
/product="photosystem II cytochrome b559 alpha subunit"
/translation="MSGSTGERSFADIITSIRYWVIHSITIPSLFIAGWLFVSTGLAY
DVFGSPRPNEYFTESRQGIPLITGRFDPLAQLDEFSRSF"
gene 69552..69647
/gene="petL"
CDS 69552..69647
/gene="petL"
/codon_start=1
/transl_table=11
/product="cytochrome b6/f complex subunit VI"
/translation="MTTITSYFGFLLAASTITPALLISLSKIRLI"
gene 69822..69935
/gene="petG"
CDS 69822..69935

```
                     /gene="petG"
                     /codon_start=1
                     /transl_table=11
                     /product="cytochrome b6/f complex subunit V"
                     /translation="MIEVFLFGIVLGLIPITLAGLFVTAYLQYRRGDQLDL"
gene                 complement(70055..70128)
                     /gene="trnW-CCA"
tRNA                 complement(70055..70128)
                     /gene="trnW-CCA"
                     /product="tRNA-Trp"
                     /note="anticodon:CCA"
gene                 complement(70282..70355)
                     /gene="trnP-UGG"
tRNA                 complement(70282..70355)
                     /gene="trnP-UGG"
                     /product="tRNA-Pro"
                     /note="anticodon:UGG"
gene                 complement(70284..70354)
                     /gene="trnP-GGG"
tRNA                 complement(70284..70354)
                     /gene="trnP-GGG"
                     /product="tRNA-Pro"
                     /note="anticodon:GGG"
gene                 70731..70865
                     /gene="psaJ"
CDS                  70731..70865
                     /gene="psaJ"
                     /codon_start=1
                     /transl_table=11
                     /product="photosystem I subunit IX"
                     /translation="MRDIKTYLSTAPVLTTLWFGSLAGLLIEINRLFPDALTFPFFSF
                     "
gene                 71292..71492
                     /gene="rpl33"
CDS                  71292..71492
                     /gene="rpl33"
                     /codon_start=1
                     /transl_table=11
                     /product="ribosomal protein L33"
                     /translation="MAKGKDVRVVVILECTSCVRNGLNKESRGISRYITQKNRHNTPS
                     QLDLKKFCPYCYKHTIHGEIKK"
gene                 71633..71938
                     /gene="rps18"
CDS                  71633..71938
                     /gene="rps18"
                     /codon_start=1
                     /transl_table=11
```

```
                     /product="ribosomal protein S18"
                     /translation="MDKSKRPFHKSKRSFHRRLPPIGSGDRIDYRNMSLINQFISEQG
                     KILSRRVNRLTLKQQRLITIAIKQARILSSLPFLNNEKQFERTGSIPRTTGPRTRNK"
gene                 complement(72196..72549)
                     /gene="rpl20"
CDS                  complement(72196..72549)
                     /gene="rpl20"
                     /codon_start=1
                     /transl_table=11
                     /product="ribosomal protein L20"
                     /translation="MTRVRRRYIARRRRTKIRLFAATFRGAHSRLTRTTTQQKMRALV
                     STHRDRGRRKRSFRRLWITRINAVTRENRGSHSYSRLILDLYKRQLLLNRKIPAQIAI
                     SNRNCLDTISNAIIK"
CDS                  join(complement(73307..73420),144824..145035,
                     145534..145564)
                     /trans_splicing
                     /codon_start=1
                     /transl_table=11
                     /product="ribosomal protein S12"
                     /translation="MPTIKQLIRNTRQPIRNVTKSPALRGCPQRRGTCTRVYTITPKK
                     PNSALRKVARVRLTSGFEITAYIPGIGHNSQEHSVVLVRGGRVKDLPGVRYHIVRGTL
                     DAVGVKDNMGSKSQNK"
misc_feature         join(complement(73307..73420),144824..145035,
                     145534..145564)
                     /note="trans-splicing"
gene                 complement(73307..145560)
                     /gene="rps12"
gene                 complement(73557..75592)
                     /gene="clpP"
CDS                  complement(join(73557..73802,74458..74748,75524..75592))
                     /gene="clpP"
                     /codon_start=1
                     /transl_table=11
                     /product="clp protease proteolytic subunit"
                     /translation="MPIGVPKVPFRSPGEEDAVWVDVNRLHRERLLFLGQEVDSEISN
                     QLVGLMVYLSIEDDTRDLYLFINSPGGWVIPGIAIYDTMQFVPPDVHTICMGLAASMG
                     SFILVGGEITKRLAFPHARVMIHQPASSFYEAPTGEFILEAEELLKLRETLTRVYVQR
                     TGNPLWVVSEDMERDVFMSATEAQDYGIVDLVAIENTGDFA"
exon                 complement(73557..73802)
                     /gene="clpP"
                     /number=3
exon                 complement(74458..74748)
                     /gene="clpP"
                     /number=2
exon                 complement(75524..75592)
                     /gene="clpP"
                     /number=1
```

gene 76021..77547
/gene="psbB"
CDS 76021..77547
/gene="psbB"
/codon_start=1
/transl_table=11
/product="photosystem II CP47 chlorophyll apoprotein"
/translation="MGLPWYRVHTVVLNDPGRLLSVHIMHTALVSGWAGSMALYELAV
FDPSDPVLDPMWRQGMFVIPFMTRLGINNSWGGWSITGGTITNPGIWSYEGVAGAHIV
FSGLCFLAAIWHWVYWDLEIFCDERTGKPSLDLPKIFGIHLFLSGVACFGFGAFHVTG
LYGPGIWVSDPYGLTGKVQSVNPAWGAEGFDPFVPGGIASHHIAAGTLGILAGLFHLS
VRPPQRLYKGLRMGNIETVLSSSIAAVFFAAFVVAGTMWYGSATTPIELFGPTRYQWD
QGYFQQEIYRRVGASLAENLSLSEAWSKIPEKLAFYDYIGNNPAKGGLFRAGSMDNGD
GIAVGWLGHPIFRDKEGHELFVRRMPTFFETFPVVLVDGDGIVRADVPFRRAESKYSV
EQVGVTVEFYGGELNGVSYSDPATVKKYARRAQLGEIFELDRATLKSDGVFRSSPRGW
FTFGHATFALLFFFGHIWHGARTLFRDVFAGIDPDLDAQVEFGAFQKLGDPTTRRQVV
"
gene 77725..77832
/gene="psbT"
CDS 77725..77832
/gene="psbT"
/codon_start=1
/transl_table=11
/product="photosystem II protein T"
/translation="MEALVYTFLLVSTLGIIFFAIFFRDPPKVPTKKTK"
gene complement(77891..78022)
/gene="psbN"
CDS complement(77891..78022)
/gene="psbN"
/codon_start=1
/transl_table=11
/product="photosystem II protein N"
/translation="METATLVAISISGSLVSFTGYALYTAFGQPSQQLRDPFEEHGD"
gene 78130..78351
/gene="psbH"
CDS 78130..78351
/gene="psbH"
/codon_start=1
/transl_table=11
/product="photosystemIIphosphoprotein"
/translation="MATQTVEGSARSGPRRTITGDLLKPLNSEYGKVAPGWGTTPFMG
VAMALFAIFLSIILEIYNSSVLLDGISMN"
gene 79257..79904
/gene="petB"
CDS 79257..79904
/gene="petB"
/codon_start=1

/transl_table=11
/product="cytochrome b6"
/translation="MSKVYDWFEERLEIQAIADDITSKYVPPHVNIFYCLGGITLTCF
LVQVATGFAMTFYYRPTVTEAFASVQYIMTEANFGWLIRSVHRWSASMMVLMMILHVF
RVYLTGGFKKPRELTWVTGVVLAVLTASFGVTGYSLPRDQIGYWAVKIVTGVPEAIPV
IGSPLVELLRGSASVGQSTLTRFYSLHTFVLPLLTAVFMLMHFPMIRKQGISGPL"
gene 80102..81300
/gene="petD"
CDS join(80102..80108,80819..81300)
/gene="petD"
/codon_start=1
/transl_table=11
/product="cytochromeb 6/f complex subunit IV"
/translation="MGVPITKKPDLNDPVLRAKLAKGMGHNYYGEPAWPNDLLYIFPV
VILGTIACNVGLAVLEPSMIGEPADPFATPLEILPEWYFFPVFQILRTVPNKLLGVLL
MVLVPTGLLTVPFLENVNKFQNPFRRPVATTVFLIGTAAALWLGIGATLPIDKSLTLG
LF"
exon 80102..80108
/gene="petD"
/number=1
exon 80819..81300
/gene="petD"
/number=2
gene complement(81488..82507)
/gene="rpoA"
CDS complement(81488..82507)
/gene="rpoA"
/codon_start=1
/transl_table=11
/product="RNA polymerase alpha subunit"
/translation="MVREEVAVSTRTLQWKCVESRTDSKRLYYGRFVLSPLMKGQADT
IGIAMRKALLGEIEGTCITRAKSEKVSHEYSTIVGIEESVHEILMNLKEIVLRSNLYG
TRDASICVRGPKYVTAQDIISPPSVELVDTTQHIANLTEPIHLCIEFKIERDRGYRMK
SPNNYQDGSYPIDAVSMPVRNANHSIHSYGSENEKQEILFLEIWTNGSLTPKEALREA
SRTLIDLFIPFLHAEEEDIHFYLEDNQNRFTVSFFTFHDRLANIRKNKKGIALKCIFI
DQSELPSRTYNCLKRSNIHTLLDLLNNSQEDLMRIEHLRIEDVKQILDILQKHFAIYL
PKNKF"
gene complement(82576..82968)
/gene="rps11"
CDS complement(82576..82968)
/gene="rps11"
/codon_start=1
/transl_table=11
/product="ribosomal protein S11"
/translation="MTKAIPRIGSRKNGRRIQKGVIHVQASFNNTIVTVTDVIGRVVS
WSSAGTCGFRGTRRGTPFAAQTAAGNAIRKAVDQGLQRAEVMIKGPGLGRDAALRAIR
RSGILLSFVRDVTPMPHNGCRPPKKRRV"

```
gene            complement(83081..83194)
                /gene="rpl36"
CDS             complement(83081..83194)
                /gene="rpl36"
                /codon_start=1
                /transl_table=11
                /product="ribosomal protein L36"
                /translation="MKIRASIRKICEKCRLIRRRGRIIVICSNPKHKQRQG"
gene            complement(83310..83543)
                /gene="infA"
CDS             complement(83310..83543)
                /gene="infA"
                /codon_start=1
                /transl_table=11
                /product="translational initiation factor 1"
                /translation="MKEQKLIHEGLITESLPNGMFRVRLDNEDLILGYVSGRIRRSFI
                RILPGDRVKIEVSRYDSTRGRIIYRLRNKESND"
gene            complement(83660..84058)
                /gene="rps8"
CDS             complement(83660..84058)
                /gene="rps8"
                /codon_start=1
                /transl_table=11
                /product="ribosomal protein S8"
                /translation="MGRDTIADIITSIRNANMDKKGTVRVASTNIAENIVKILLQEGF
                IENVRKHRENNKYFLVSTLRHRRNRKGTYRNILKRISRPGLRIYSNHQRIPRILGGMG
                VVILSTSRGIMTDREARLERIGGEILYYIW"
gene            complement(84238..84606)
                /gene="rpl14"
CDS             complement(84238..84606)
                /gene="rpl14"
                /codon_start=1
                /transl_table=11
                /product="ribosomal protein L14"
                /translation="MIQPQTHLNVADNSGARELMCIRIIGASNQRYAHIGDVIVAVIK
                EAVPNMPLERSEVIRAVIVRTCKELKRDNGMIIRYDDNAAVVIDQEGNPKGTRVFGAI
                ARELRQLNFTKIVSLAPEVL"
gene            complement(84736..85140)
                /gene="rpl16"
CDS             complement(84736..85140)
                /gene="rpl16"
                /codon_start=1
                /transl_table=11
                /product="ribosomal protein L16"
                /translation="MLSPKRTRFRKQHRGRMKGISYRGNHICFGRYALQALEPAWITS
                RQIEAGRRAMTRYARRGGKIWVRIFPDKPVTVRPTETRMGSGKGSPEYWVSVVKPGRI
                LYEMGGVSETVARAAISIAACKMPIRTQFIIA"
```

gene complement(86264..86926)
/gene="rps3"
CDS complement(86264..86926)
/gene="rps3"
/codon_start=1
/transl_table=11
/product="ribosomal protein S3"
/translation="MGQKINPLGFRLGENQSHRSLWFAQPKSYYIGLQEDEKIRDWIK
IYVQKNIRVSSSFEGIGIAHIEIQKKMDLIQVIIYIGFPNLLIEGQTRGIEELQINVQ
KGLHSVNRRLNIAITRVAKPYGQPNILAEYIALQLKNRVSFRKAMKKAIELTEQADTK
GIQVEIAGRIDGKEIARVEWIREGRVPLQTIRAKIDHCSYTVRTAYGALGIKIWIFVD
EQ"
gene complement(86817..87380)
/gene="rpl22"
CDS complement(86817..87380)
/gene="rpl22"
/codon_start=1
/transl_table=11
/product="ribosomal protein L22"
/translation="MKRSSSTQVQALAQRICMSAHKARRVIDQIRGHSYEKTLMLLEL
MPYRAFYPIFKLVYSAAANASHNKSFNEADSVISKAEVNGGTIVKKLKPRARGRSYPI
ERPACHIIIVLKDSSKKKTDQDIFLETKNVWRDPIIERYIEKEREREKKDGSKNKSTW
FPPWRKPKSSFPLVRTTKKLLHRSPGR"
gene complement(87482..87760)
/gene="rps19"
CDS complement(87482..87760)
/gene="rps19"
/codon_start=1
/transl_table=11
/product="ribosomal protein S19"
/translation="MTRSLKKNPFVANHLLEKIKKLNMREEKEIIVTWSRASTIIPTM
IGNTIAIHNGKAHLPIYITDRMVGQKLGEFAPTLTFQGHARNDTRSLR"
gene complement(87835..89329)
/gene="rpl2"
CDS complement(join(87835..88266,88940..89329))
/gene="rpl2"
/exception="RNA editing"
/codon_start=1
/transl_except=(pos:complement(89327..89329),aa:Met)
/transl_table=11
/product="ribosomal protein L2"
/translation="MIHLYKTSTPSIRKGSIDSQAKSNPRNNLIYGQHRCGKGRNSRG
IITAGHRGGGHKRLYRKIDFRRNEKDISARIVTIEYDPNRNAYICLIHYGDGEKRYIL
HPRGAIIGDTIVSGTEVPISMGNALPLSTHMPLGTAVHNIEITLGKGGQLTRAAGAVA
KLIAKEGKSVTLRFPSGEVRLISKNCSATVGQVGNVGANQKSLGRAGSKCWLGRRPVV
RGVVMNPVDHPHGGGEGRAPIGRKKPTTPWGYPALGRKSRKRNKYSNSFIIRRRK"
exon complement(87835..88266)

/gene="rpl2"
/number=2
exon complement(88940..89329)
/gene="rpl2"
/number=1
gene complement(89361..89588)
/gene="rpl23"
CDS complement(89361..89588)
/gene="rpl23"
/codon_start=1
/transl_table=11
/product="similar to ribosomal protein L23"
/translation="MGKKVESGSTRTEIKHWVELFFGVKVIAINSHQLPGKGRRTGPI
MGHTMHYRRMIITLQPGYSILPLIEKRKEFK"
gene complement(89738..89811)
/gene="trnI-CAU"
tRNA complement(89738..89811)
/gene="trnI-CAU"
/product="tRNA-Ile"
/note="anticodon:CAU"
gene 89895..96866
/gene="ycf2"
CDS 89895..96866
/gene="ycf2"
/codon_start=1
/transl_table=11
/product="hypothetical chloroplast RF2"
/translation="MRGHQFKSWIFELREIKNSHDFLDSWIQPDSVKSFTSFFFHQER
FMKLFDSRIWSVLISRDSQGSIRRHCMIKGVVLLVLVVAVLIYNRNRVERKNIYLMGL
LPKPLRSIGPPNYTLKESFWSSNLNRLIVSLLYLPKGKNIYESCFMDPKESTWVLPIT
KKCIMSESNWGSQRWRNAIVKKRNSSCKISNEIAAGIEISFKEKDIKYLEFFFVSYTN
DPIRKDHDWKLFDRLSPSKKRNIINLNSGQLFEILVKHLICYLMSAFREKRPIDEGGF
FKQQGAEATIQSNEIEHVSHLLSRNKGGIFLKNCAQFHMWQFRQDLFVIGGKNRHKSD
FLRNVSRENLIWLDNAWLVNRNRVFSKVRNVSSNIQYDSIRSIFFQVTDSSQSKGFSD
QSIDPFNSISNEGSEYHTLINQTEIQQLKKRSILLDTSFLQTERTEIKSDRFSKYLSG
YSSMARLFPEREKQMNNHLLPEEIEEFLGNPTRSIRSFFSDRWSELHLGLNPTERSTI
DQKLLKKQQGVSFVPSRRSENKEIVDIFKIITYLQNTSSVHSIAADPGWDMVPKDEPD
MDSSNKISFLNENAFFDLFHLFHDRNKGGYRLHHEFELEETFQEMADLFTLSITEPGL
AYHNKEFGLSIDSYGKLLNEVFNSGDESKKKSLLVLPSIFYDLFLLVLSSIFYDLFLL
VLLSIFYDLFLLVLLSIFYEENESFYRKIKKKSVRISCGNDLEDPKPKIAVFAHNNIM
EAIHQYRLIRNQIQIQYSTYGYIRNVLNRFFLMNRPDCNFAYGIQKHPIGIQKHPIGN
DILNHLTIIIDKINQHLSNLKKIKKKWFDPLISRTERSTNLDPNVYRYKCSNGSKNFQ
EHLEHFVSEQKHRFQVMFDRLRINQYSIDWSEVIDKQDLSKSLRFFLSKSLLLLSKSL
LFLSKSLPFFVVSLGNISIHRAEIHIYELKGLNDQPGNQLLESIGVQIVYLNKLKPFL
LYDHDTSQRSKFLINTGTILPFLFNKIQKCMIDSFRTRKNRKKSFENTDSYFSMISHD
RNNWLNPQKSSLISSFYRANRLQFLNHPHCFWFYCNKGFHFYGEKTRIHNYDFTYAQF
PNILCIRNKKFSLCFGKKKHVLGERETISPIESQVSGIFIPNNVSQSGNKTYNLYKSF

HFSIGSDPSVPIYSIADISGTPVIEEQIVNFERTYCQLLSDMNLSDSEGKNLHHYLRF
NSNMGLIHTPCFEKYVPSGKRKELSLCLKKNVEKGEVGRTLQRDSAFSNLSKWNLFQT
YMPWFLTWTGCKYLYFTLKNNIYLILNIPFQYSLSGSQNFVSVFHDMMHGSDISWPIP
QKKWWSILPQRNLISESSSKCLQNLLLSEEMIHRNNESPIPLIWTHLRSPNAWEFLYS
ILFLLLVAGYLVRTHLLFVFRASSELQTELEKIKSLMIPSYMIELRKLLDRYPTSELN
SFWLKNLFLVALEQLGDSLEEIRDSASGGNMLLGGGPAYGVKSIRSKKKYLNINLIDL
ISIIPNPINRITFSRNTRHLSRTSKEIYSLIRKRKNVNGDWIDDKIESWVANSDSIDD
EEREFLVQFSTLTTEKRIDQILLSLTHSDRLSKNDSGYQMIEQPGSIYLRYLVDIHKK
YLMNYEFNRSCLAERRIFLAHYQTITYSQTSCGANSSHFPSHGKPFSLRLALSPSRGI
LVIGSIGTGRSYLVKYLATNSYVPFITVFPNKFLDDKPKGYLIDDIDIDDSDDIDIDD
SDDIDDDLDTELLTMTNVLTMYMTPKIDRFDTTLQLELAKAMSPCIIWIPNIHDLYVN
ESNYLSLGLLVNYLSRDSERCSTRNILVIASTHIPQKVDPTLIAPNKLKKCMKIRRLL
IPQQRKHFFILSYTRGFNLEKKMFHTNSNRFGSITMGSNARDLVALTNEALSISITQK
KSIIDTNTIRSALHRQTWDLRSQVRSVQDHGILFYQIGRAVAQNVLLSNCPIDPISIY
MKKKSCKEGDSYLYKWYFELGTSMKKLTILLYLLSCSAGSVAQDLWSPPGPDEKNWIT
SYGFVENDSDLVHGLLEVEGALLGSSRTEKDCSQFDNDRVTLLLRSEPRNQLDMMQNG
SCSIVDQRFLYEKYESEFEEGEREGALDPQQIEEDLFNHIVWAPRIWRPCGNLFDCIE
RTNELGFPYWARSFRGKRIIYHKEDELQENDSEFLQSGTMQYQTRDRSSKEQGFFRIS
QFIWDPADPFFFLFKDQPFVSVFSRREFFADEEMSKGLITSQTNPPTSIYKRWFIKNT
QEKHFELLIHRQRWLRTNSSLSNGSFRSNTPSESYQYLSNLFLSNGTLLDQMTKALLR
KRWLFPDEMKHLIHVTG"

repeat_region　　93706..113784
　　/rpt_type=inverted

gene　　complement(97206..97286)
　　/gene="trnL-CAA"

tRNA　　complement(97206..97286)
　　/gene="trnL-CAA"
　　/product="tRNA-Leu"
　　/note="anticodon:CAA"

gene　　complement(97847..100078)
　　/gene="ndhB"

CDS　　complement(join(97847..98602,99305..100078))
　　/gene="ndhB"
　　/codon_start=1
　　/transl_table=11
　　/product="NADH-plastoquinone oxidoreductase subunit 2"
　　/translation="MWHVQNENFILDSTRIFMKAFHLLLFHGSFIFPECILIFGLILL
LMIDSTSDQKDIPWLYFISSTSLVMSITALLFRWREEPMISFSGNFQTNNFNEIFQFL
ILLCSTLCIPLSVEYIECTEMAITEFLLFVLTATLGGMFLCGANDLITIFVAPESFSL
CSYLLSGYTKRDVRSNEATTKYLLMGGASSSILVHGFSWLYGSSGGEIELQEIVNGLI
NTQMYNSPGISIALISITVGIGFKLSPAPSHQWTPDVYEGSPTPVVAFLSVTSKVAAS
ASATRIFDIPFYFSSNEWHLLLEILAILSMILGNLIAITQTSMKRMLAYSSIGQIGYV
IIGIIVGDSNDGYASMITYMLFYISMNLGTFARIVSFGLRTGTDNIRDYAGLYTKDPF
LALSSALCLLSLGGLPPLAGFFGKLHLFWCGWQAGLYFLVSIGLLTSVVSIYYYLKII
KLLMTGRNQEITPHVRNYRRSPLRSNNSIELSMIVCVIASTIPGISMNPIIAIAQDTL
F"

exon　　complement(97847..98602)

```
                     /gene="ndhB"
                     /number=2
     exon            complement(99305..100078)
                     /gene="ndhB"
                     /number=1
     gene            complement(100405..100872)
                     /gene="rps7"
     CDS             complement(100405..100872)
                     /gene="rps7"
                     /codon_start=1
                     /transl_table=11
                     /product="ribosomal protein S7"
                     /translation="MSRRGTAEEKTAKSDPIYRNRLVNMLVNRILKHGKKSLAYQIIY
                     QAVKKIQQKTETNPLSVLRQAIRGVTPDIAVKARRVGGSTHQVPIEIESTQGKALAIR
                     WLLGASRKRPGRNMAFKLSSELVDAAKGSGDAIRKKEETHRMAEANRAFAHFR"
     gene            complement(100927..100956)
                     /gene="rps12"
                     /pseudogene="unknown"
     gene            complement(101482..101724)
                     /gene="rps12"
     gene            103559..103630
                     /gene="trnV-GAC"
     tRNA            103559..103630
                     /gene="trnV-GAC"
                     /product="tRNA-Val"
                     /note="anticodon:GAC"
     gene            103859..105349
                     /gene="rrn16"
     rRNA            103859..105349
                     /gene="rrn16"
                     /product="16S ribosomal RNA"
     gene            105599..105640
                     /gene="trnI-GAU"
     tRNA            105599..105640
                     /gene="trnI-GAU"
                     /product="tRNA-Ile"
                     /note="anticodon:GAU"
     gene            105740..106118
                     /gene="ycf68"
     CDS             join(105740..105781,105810..106118)
                     /gene="ycf68"
                     /codon_start=1
                     /transl_table=11
                     /product="hypothetical chloroplast RF68"
                     /translation="MAYSSCSNRSLKPNSGEIQCRSNFLFTRGIRAVRGGPPWLLSSR
                     ESIHPLSVYGQLSLEHRFRFGLNGKMEHLTTHLHRPRTTRSPLSFWGDGGIVPFEPFF
                     FMLFPRRSGESSNQ"
```

```
exon            105740..105781
                /gene="ycf68"
                /number=1
exon            105810..106118
                /gene="ycf68"
                /number=2
gene            106580..106614
                /gene="trnI-GAU"
tRNA            106580..106614
                /gene="trnI-GAU"
                /product="tRNA-Ile"
                /note="anticodon:GAU"
gene            106679..106716
                /gene="trnA-UGC"
tRNA            106679..106716
                /gene="trnA-UGC"
                /product="tRNA-Ala"
                /note="anticodon:UGC"
gene            107702..110505
                /gene="rrn23"
rRNA            107702..110505
                /gene="rrn23"
                /product="23S ribosomal RNA"
gene            110624..110726
                /gene="rrn4.5"
rRNA            110624..110726
                /gene="rrn4.5"
                /product="4.5S ribosomal RNA"
gene            110947..111067
                /gene="rrn5"
rRNA            110947..111067
                /gene="rrn5"
                /product="5S ribosomal RNA"
gene            111300..111373
                /gene="trnR-ACG"
tRNA            111300..111373
                /gene="trnR-ACG"
                /product="tRNA-Arg"
                /note="anticodon:ACG"
gene            complement(111965..112036)
                /gene="trnN-GUU"
tRNA            complement(111965..112036)
                /gene="trnN-GUU"
                /product="tRNA-Asn"
                /note="anticodon:GUU"
gene            112414..118035
                /gene="ycf1"
```

```
CDS             112414..118035
                /gene="ycf1"
                /codon_start=1
                /transl_table=11
                /product="hypothetical chloroplast RF1"
                /translation="MILKSFLLGNLLSLYMKIINSVVVVGLYYGFLTTFSIGPSYLFL
                LRARIMEEGTEKEVSATTGFITGQLMMFISIYYAPLHLALGRPHTITVLVLPYLLFHF
                FWNNHKHFLDYGSTTRNSMRNLSIQCVFLNNLIFQLFNHFILPSSTLVRLVNIYMFRC
                NNKMLFVTSSFVGWLIGHIFFMKWVGLVLFWIRQNHSIRSNVLIRSNKYLVSELRNSM
                ARIFTILLFITCVYYLGRIPSPIVTKKLKETSKTEERGESEEETDVEIEKTSETKGTK
                QEQEGSTEEDPSLCSEEREDPKKLHEKKKRQEILKLEILKEKEDKDLFWFEKPLVNLL
                FDYKRCNRPLRYIKKNLFQNAVRNEMSQYFFHVCPVDGKQIISFTYPPSLSIFLEMMQ
                RKMSLCTTEKLSPEDLYNHWVYTNEQKRYSLSNEFINRIEVLNKGSLTMDVLEKRTRL
                YNDKNNQEDKNNQEDKNNQEDKNNQEDKNNQECLPRVYDPFLNGPYRGTIKKVYSRSM
                VDDSITSTEDSIGTEDSIGMVWINKIHDRLPTDYQKLEHKTDTFNGEPLSTDIGPFLT
                SISELARKSTTGFSLNFKKLVLISEQRRFDSENKKKCLKFLFDVITTDQNNQTIQNKS
                IGIEEIGKKIPRRSYKLINSFEEREEENEEESEESTENHGIRSRKAKRVVIYTDKADP
                DQNTNTHTSTSTNSDQAEELALVRYSQQSDFRRDIVKGSIRAQRRKMVIWEMFQANAH
                SLLFLDRIDKTFFFSFDISRTMNLIFRNWIDTGPKLKTSDSEEEEAKEEAKKMEDKKN
                ENERIAIAETWDTFIFAQAIRGTMLVTQSILRKYIILPSLIIAKNLGRMLLFQFPEWY
                EDLKEWNREMHVKCTYNGVQLSETEFPKNWLTDGIQIKILFPFCLKPWRKSKLRSHHR
                DPIQKKGKTENFCFLTIWGKETELPFGSARQQPSFFEPIYNEFEKKKIKVKKKCFLVL
                RVFKKKTKQFRKVSKEKTRWIIKTILFLKRKIKEFANVNPIFLFVLKKVYEPNENGKD
                SIIISSNKIVPKSTSTIRIRFMDWANYSLTEKKKKDLSDRTTLIRNQIERGAKDKRKI
                FLTPDINISPNDTSCGDKRSESQKHIWQISKGKSNRFIFIRKWHYFLTFLDERIYIHI
                FLYTVNVSRVNVQLFLESTKKIIDKYIHKEGIDEINQKKMHFISTIKKSISNISKNKS
                KISGDLYSFSQASVFYKLSQIQAINKKYHLRSLLQYREAYLILKDRIRNFFGTRRILD
                SKSRHKKLPNSGMNEWKNWLRGHYQYNLSQARWSKLVPQKWRTRVNWRRTIQNKDSKK
                NSYEKAQFIHYEKKNDYEVNSLTIKKAKLKKNYRYDLFSYKYINYGDRKDSYIYPSSL
                QVNEDREIPYNYNTPKIEPFYVLGDICISDYLGEESIIGTGKSTDRKYLEWKIFDLFL
                RKNIDIESWTDTDTGTNINKMTKTETDYYQMIDKKDLFYLTIHQEINPPNQKKKFFLM
                GMNKEMLYRPILNTKSWFFSEFVPLYDAYKIKPWIIPIKLLLLIFNGNENISENKNIN
                ENQKKDLRISSNQKEYLELKNRNQEEKEQLGHGNIGSDARKRQKDFEKDYTESDIQKR
                EKKGQPESNKKAKQELFLKKYLLFQLRWDDLLNNRIFNNVKVYCFLLRLINAKEIAIS
                SIQGGEMHLDVMLIQTNPTLPELIKKGILILEPVRLSIKWDRQFIMYQTIGISLVHNN
                KCQTNGRYREKRYVDENYFNGSIVQHKKMLVNRDENHYDLLVPENILSPRRRRELRIL
                ICFNSGNRNAMDRNPVFFNDNNVRNWVQFLDEDKHIDTDINKFIQFKLFLWPNYRLED
                LACMNRYWFDTNNGSRFSMSRIHMYPRFGIS"
misc_feature    113785..132877
                /note="SSC"
gene            118362..118628
                /gene="rps15"
CDS             118362..118628
                /gene="rps15"
                /codon_start=1
                /transl_table=11
                /product="ribosomal protein S15"
```

/translation="MVKNSFISVIPKEEKNKGSVEFQVISFTNKIQRLTSHFELHRKD
YLSQIGLRKILGKRQRLLAYLSKKNRVRYKKLIDQLDIREPKTR"
gene 118736..119917
/gene="ndhH"
CDS 118736..119917
/gene="ndhH"
/codon_start=1
/transl_table=11
/product="NADH-plastoquinone oxidoreductase subunit 7"
/translation="MNVPATRKDLMIVNMGPHHPSMHGVLRLIVTLDGEDVIDCEPVL
GYLHRGMEKIAENRTIIQYLPYVTRWDYLATMFTEAITVNAPEQLGNIQVPKRASYIR
VIMLELSRIASHLLWLGPFMADIGSQTPFFYIFRERELLYDLFEAATGMRMMHNYFRI
GGVAADLPHGWIDKCLDFCDYSLTGIVEYQKLITQNPIFLERVEGVGIIGGEEAINWG
LSGPMLRASGIQWDLRKVDHYECYDEFDWEVQWQKEGDSLARYLVRINEMTESIKIIQ
QALEGIPGGPYENLEVRRFDRASDSEWNGFEYRFISKKPSPTFELSKQELYVRVEAPK
GELGIFLIGDNSVFPWRWKIRPPGFINLQILPQLVKRMKLADIMTILGSIDIIMGEVD
R"
gene 119919..122137
/gene="ndhA"
CDS join(119919..120482,121598..122137)
/gene="ndhA"
/codon_start=1
/transl_table=11
/product="NADH-plastoquinone oxidoreductase subunit 1"
/translation="MIIDTTEVQAINSFSRSESLKEVYDLLWLLVPIFTPVSGITIGV
LVIVWLEREISAGIQQRIGPEYAGPLGILQALADGTKLLLKEDLLPSRGDVRLFSMGP
SIAVISILLSYLVIPFGYRLVLADLSIGVFLWIAISSIAPIGLLMSGYGSNNKYSFSG
GLRAAAQSISYEIPLTPCVLSISLRVIRLSNSSSTVDIVEAQSKYGFWGWNLWRQPIG
FIVFLISSLAECERLPFDLPEAEEELVAGYQTEYSGIKSGLFYVASYLNLLVSSLFVT
VLYLGGWNFSIPYIFISEPFGINKTGGVFGMTIGILITLAKAYLFLFIPITTRWTLPR
MRMDQLLNLGWKFLLPISLGNLLLTTSSQLVSL"
exon 119919..120482
/gene="ndhA"
/number=1
exon 121598..122137
/gene="ndhA"
/number=2
gene 122216..122758
/gene="ndhI"
CDS 122216..122758
/gene="ndhI"
/codon_start=1
/transl_table=11
/product="NADH-plastoquinone oxidoreductase subunit I"
/translation="MFPMVTGFMNYGQQTIRAARYIGQSFMITLSHVNRLPVTIQYPY
EKSITSERFRGRIHFEFDKCIACEVCVRVCPIDLPVVHWRLETDIRKKRLLNYSIDFG
ICIFCGNCVEYCPTNCLSMTEEYELSTYDRHELNYNQIALGRLPMSVIGDYTIRTITN

STPIKIIRGKPLDSKTITNY"
gene 123090..123620
/gene="ndhG"
CDS 123090..123620
/gene="ndhG"
/codon_start=1
/transl_table=11
/product="NADH-plastoquinone oxidoreductase subunit 6"
/translation="MDLPGPIHDILLVFLGSGLILGGLGVVLLTNPIYSAFSLGLVLV
CISLFHIPSNSYFVAAAQLLIYVGAVNVLILFAVMFMNGSEYYKDFYLWTVGDGVTSL
VCTSILFSLITTISDTSWYGIVWTTRSNQIIEQDLTSNVQQIGIHLSTDFYLPFELIS
IILLVALIGAIAMARQ"
gene 123868..124173
/gene="ndhE"
CDS 123868..124173
/gene="ndhE"
/codon_start=1
/transl_table=11
/product="NADH-plastoquinone oxidoreductase subunit 4L"
/translation="MMTEHVLILSAYLFSIGIYGLITSRNMVRALMCLELILNAVNIN
LVTFSDLFDSRQLKGDIFSIFVIAIAAAEAAIGPAIVSSIHRNRKSTRINQSNLLNK"
gene 124441..124686
/gene="psaC"
CDS 124441..124686
/gene="psaC"
/codon_start=1
/transl_table=11
/product="photosystem I subunit VII"
/translation="MSHSVKIYDTCIGCTQCVRACPTDVLEMIPWDGCKAKQIASAPR
TEDCVGCKRCESACPTDFLSVRVYLWHETTRSMGLAY"
gene 124807..126312
/gene="ndhD"
CDS 124807..126312
/gene="ndhD"
/exception="alternative start codon"
/codon_start=1
/transl_except=(pos:124807..124809,aa:Met)
/transl_table=11
/product="NADH-plastoquinone oxidoreductase subunit 4"
/translation="MYFPWLTIIVVLPISAGSSIFFLPRRGNKNKVVRWYTICICLLE
LLLTTYAFCYHFQLDDPLIQLEEAYKWINTFDFHWRPGIDGLSIGPILLTGFITTLAT
LAARPVTRDSRLFHFLMLAMYSGQIGSFSSRDLLLFFLMWELELIPVYLLVSIWGGKK
RLYSATKFILYTAGGSIFLLMGVPGMGLYGSNEPTLNFETLANQSYPLGLEIIFYIGF
LIAYAVKSPIIPLHTWLPDTHGEAHYSTCMLLAGILLKMGAYGLVRINMELLPHAHSI
FSPWLMIVGAIQIIYAASTSFGQRNLKKRIAYSSVSHMGFTLIGIGSITDTGINGAIL
QIISHGFIGAALFFLAGTSYDRIRLVYLDEMGGIAIPMPKIFTMFSSFSMASLALPGM
SGFVAESVVFFGIITSPKYLLMPKILITFVMAIGMILTPIYSLSMSRRMFYGYKLFNV

PNSYFFDSGPRELFVSVCILLPVIGIGIYPDFVLSLSIDRIEAILSIYFHK"
gene complement(126535..127485)
/gene="ccsA"
CDS complement(126535..127485)
/gene="ccsA"
/codon_start=1
/transl_table=11
/product="CcsA"
/translation="MIFATLEHILTHISFSIISIVITIHLMTLLIHETVVLFDLSEKA
MMATFFCITGLLVTRWIYSRHLPLSDLYESLMFLSWSFSIIHMFPKRRNQKSYLSAIT
APSAIFTQGFATSGLSTEMHQSAILVPALQSQWLMMHVSMMLLSYAALLCGSLLSIAL
LVITFRKNLDIPRKSNHLLIGSFSFVNEKRSVLQNTSFLSFRNYHRYQLTQQLDQCSY
RVISLGFTFLTIGILSGAVWANEAWGSYWNWDPKETWAFITWTIFAIYLHSRTNQSFQ
GVDSAIVASIGFLIIWICYFGVNLLGIGLHSYGSFTLTTN"
gene complement(127573..127652)
/gene="trnL-UAG"
tRNA complement(127573..127652)
/gene="trnL-UAG"
/product="tRNA-Leu"
/note="anticodon:UAG"
gene complement(129044..129217)
/gene="rpl32"
CDS complement(129044..129217)
/gene="rpl32"
/codon_start=1
/transl_table=11
/product="ribosomal protein L32"
/translation="MAVPKKRTSLSKKHIRRNIWKGRGYQAAAKALSLAKSISTGHSK
SFFVRQTSNKALE"
gene 130423..132657
/gene="ndhF"
CDS 130423..132657
/gene="ndhF"
/codon_start=1
/transl_table=11
/product="NADH-plastoquinonevoxidoreductase subunit 5"
/translation="MEHTYQYAWIIPFALLPVTMSIGLGLLLVPTATKNLRRMWTFPS
VSLLSIVMVFSSDLSIQQINGSSIYQHLWSWTINTDFSLEFGYLIDPLTSIMSILITT
VGIMVLIYSDNYMSHDQGYLRFFAYMSFSNTSMLGLVTSSNLIQIHIFWELVGMCSYL
LIGFWFTRPAAANACQKAFVTNRVGDFGLLLGILGFYWITGSFEFRDLFEIFNNLIRN
NGVNSLFATLCASLLFVGAVAKSAQFPLHVWLPDAMEGPTPISALIHAATMVAAGIFL
VARFLPLFTVIPYIMNLISLIGVITVLLGATLALAQRDIKRSLAYSTMSQLGYIMLAP
GIGSYRAALFHLITHAYSKALLFLGSGSIIHSMEPIVGYSPDKSQNMVLMGGLTKYVP
ITKNTFLLGTLSLCGIPPLACFWSKDEILNDSWLYSPIFAIIACFSAGLTAFYMFRMY
LLTFDGHLHAHFQNYSGTQNSSFYSISIWGKEGTKPVNRNLFLSTMNNNEKVSFFSRK
IYKMNGNVRNLIRSCRIYFENKDTSTYPHESDNTMLLPLLILVLFTLFVGSIGIPFDQ
GVIDFDILSKWLTPSINLLHQNSNYSVDWYEFVTNAIYSVSIACFGIFIASILYGSVN

SSFQNLDLINSFVKKTGSKKILLDRIINVIYNWSYNRGYIDLFYATCLTTSIRGLAEV
THFLDRRVIDGITNGVGVASFFVGEGIKYVGGGRISSYLFVYLSYVSGFLLIYYIYYL
FFLF"

```
gene            complement(132781..134250)
                /gene="ycf1"
CDS             complement(132781..134250)
                /gene="ycf1"
                /codon_start=1
                /transl_table=11
                /product="hypothetical chloroplast RF19"
                /translation="MILKSFLLGNLLSLYMKIINSVVVVGLYYGFLTTFSIGPSYLFL
                LRARIMEEGTEKEVSATTGFITGQLMMFISIYYAPLHLALGRPHTITVLVLPYLLFHF
                FWNNHKHFLDYGSTTRNSMRNLSIQCVFLNNLIFQLFNHFILPSSTLVRLVNIYMFRC
                NNKMLFVTSSFVGWLIGHIFFMKWVGLVLFWIRQNHSIRSNVLIRSNKYLVSELRNSM
                ARIFTILLFITCVYYLGRIPSPIVTKKLKETSKTEERGESEEETDVEIEKTSETKGTK
                QEQEGSTEEDPSLCSEEREDPKKLHEKKKRQEILKLEILKEKEDKDLFWFEKPLVNLL
                FDYKRCNRPLRYIKKNLFQNAVRNEMSQYFFHVCPVDGKQIISFTYPPSLSIFLEMMQ
                RKMSLCTTEKLSPEDLYNHWVYTNEQKRYSLSNEFINRIEVLNKGSLTMDVLEKRTRL
                YNDKNNQGKERCLCVRPKNYPPKICIIIGFIPMNKKGTA"
repeat_region   132878..152570
                /rpt_type=inverted
gene            134628..134699
                /gene="trnN-GUU"
tRNA            134628..134699
                /gene="trnN-GUU"
                /product="tRNA-Asn"
                /note="anticodon:GUU"
gene            complement(135291..135364)
                /gene="trnR-ACG"
tRNA            complement(135291..135364)
                /gene="trnR-ACG"
                /product="tRNA-Arg"
                /note="anticodon:ACG"
gene            complement(135596..135716)
                /gene="rrn5"
rRNA            complement(135596..135716)
                /gene="rrn5"
                /product="5S ribosomal RNA"
gene            135938..136040
                /gene="rrn4.5"
rRNA            135938..136040
                /gene="rrn4.5"
                /product="4.5S ribosomal RNA"
gene            complement(136139..138895)
                /gene="rrn23"
rRNA            complement(136139..138895)
                /gene="rrn23"
```

```
                     /product="23S ribosomal RNA"
gene                 complement(139048..139082)
                     /gene="trnA-UGC"
tRNA                 complement(139048..139082)
                     /gene="trnA-UGC"
                     /product="tRNA-Ala"
                     /note="anticodon:UGC"
gene                 complement(139881..139918)
                     /gene="trnA-UGC"
tRNA                 complement(139881..139918)
                     /gene="trnA-UGC"
                     /product="tRNA-Ala"
                     /note="anticodon:UGC"
gene                 complement(139983..140017)
                     /gene="trnI-GAU"
tRNA                 complement(139983..140017)
                     /gene="trnI-GAU"
                     /product="tRNA-Ile"
                     /note="anticodon:GAU"
gene                 complement(140479..140787)
                     /gene="ycf68"
CDS                  complement(140479..140787)
                     /gene="ycf68"
                     /exception="alternative start codon"
                     /codon_start=1
                     /transl_except=(pos:complement(140785..140787),aa:Met)
                     /transl_table=11
                     /product="hypothetical chloroplast RF68"
                     /translation="MGEIQCRSNFLFTRGIRAVRGGPPWLLSSRESIHPLSVYGQLSL
                     EHRFRFGLNGKMEHLTTHLHRPRTTRSPLSFWGDGGIVPFEPFFFMLFPRRSGESSNQ
                     "
gene                 complement(141248..142689)
                     /gene="rrn16"
rRNA                 complement(141248..142689)
                     /gene="rrn16"
                     /product="16S ribosomal RNA"
gene                 complement(142918..142989)
                     /gene="trnV-GAC"
tRNA                 complement(142918..142989)
                     /gene="trnV-GAC"
                     /product="tRNA-Val"
                     /note="anticodon:GAC"
gene                 145619..146086
                     /gene="rps7"
CDS                  145619..146086
                     /gene="rps7"
                     /codon_start=1
```

```
                     /transl_table=11
                     /product="ribosomal protein S7"
                     /translation="MSRRGTAEEKTAKSDPIYRNRLVNMLVNRILKHGKKSLAYQIIY
                     QAVKKIQQKTETNPLSVLRQAIRGVTPDIAVKARRVGGSTHQVPIEIESTQGKALAIR
                     WLLGASRKRPGRNMAFKLSSELVDAAKGSGDAIRKKEETHRMAEANRAFAHFR"
     gene            146413..148597
                     /gene="ndhB"
     CDS             join(146413..147186,147842..148597)
                     /gene="ndhB"
                     /codon_start=1
                     /transl_table=11
                     /product="NADH-plastoquinone oxidoreductase subunit 2"
                     /translation="MWHVQNENFILDSTRIFMKAFHLLLFHGSFIFPECILIFGLILL
                     LMIDSTSDQKDIPWLYFISSTSLVMSITALLFRWREEPMISFSGNFQTNNFNEIFQFL
                     ILLCSTLCIPLSVEYIECTEMAITEFLLFVLTATLGGMFLCGANDLITIFVAPESFSL
                     CSYLLSGYTKRDVRSNEATTKYLLMGGASSSILVHGFSWLYGSSGGEIELQEIVNGLI
                     NTQMYNSPGISIALISITVGIGFKLSPAPSHQWTPDVYEGSPTPVVAFLSVTSKVAAS
                     ASATRIFDIPFYFSSNEWHLLLEILAILSMILGNLIAITQTSMKRMLAYSSIGQIGYV
                     IIGIIVGDSNDGYASMITYMLFYISMNLGTFARIVSFGLRTGTDNIRDYAGLYTKDPF
                     LALSSALCLLSLGGLPPLAGFFGKLHLFWCGWQAGLYFLVSIGLLTSVVSIYYYLKII
                     KLLMTGRNQEITPHVRNYRRSPLRSNNSIELSMIVCVIASTIPGISMNPIIAIAQDTL
                     F"
     exon            146413..147186
                     /gene="ndhB"
                     /number=1
     exon            147842..148597
                     /gene="ndhB"
                     /number=2
     gene            149158..149238
                     /gene="trnL-CAA"
     tRNA            149158..149238
                     /gene="trnL-CAA"
                     /product="tRNA-Leu"
                     /note="anticodon:CAA"
     gene            complement(149555..152570)
                     /gene="ycf2"
     CDS             complement(149555..152570)
                     /gene="ycf2"
                     /pseudogene="unprocessed"
                     /codon_start=1
                     /transl_table=11
                     /product="hypothetical chloroplast RF21"
BASE COUNT    45810 a  30038 c  29677 g  47045 t
ORIGIN
        1 gtccaagttt atagggatag cgaatgctgg gcgaacgacg ggaattgaac ccgcgcatgg
       61 tggattcaca atccactgcc ttgatccact tggctacatc cgcccctcct ctctcaaaag
      121 gattccattt tcaccattca ttattttttg atttagtctt tattacttca ctctccttcc
```

181 tgctgaaata cagatattgt acataagaca aaatgttgta cgtaaaaaaa aaaaaagaaa

241 aatgctttga ttttttcaaa aaatcaaatt attttgaaga ataagaatat ataaaatgca

301 ggttggtaca gaagaaacta cgatattcga tcatgaaata accagcggtt ttcataagtt

361 gaataaaaga aatgaaaatg aaaaacgatt atgtgaataa aacactactg aaccaaatgg

421 atcaatacca aacttcttaa tagaacaaga agtttggtat tgatccttca acgactcgta

481 tacactaata ccaaagtatt atccgtttgt agatggaact tcgacagcag ctaggtctag

541 agggaagttg tgagcattac gttcatgcat aacttccata ccaaggttag cacgattgat

601 gatatcagcc caagtgttaa taacacgacc ttgactgtca actacagatt ggttgaaatt

661 gaaaccattt aggttgaaag ccatggtgct gatacctaaa gcagtaaacc agatacctac

721 tacaggccaa gctgctagga agaaatgtaa ggaacgggag ttgttaaaac tagcatattg

781 gaagatcaat cggccaaaat aaccatgagc agctacgata ttgtaagttt cttcctcttg

841 accgaatctg taacctgcat tagcagattc attttcagtg gtttccctga tcaaactaga

901 ggttaccaag gaaccatgca tagcactgaa tagggagccg ccgaatacac cagctacgcc

961 taacatgtga aatggatgca taaggatatt gtgctctgcc tggaatacaa tcatgaagtt

1021 gaaagtacca gatattccta aaggcatacc atcagaaaaa cttccttgac caatagggta

1081 gatcaagaaa acagcagtag ctgctgcaac aggagctgaa tatgcaacag caatccaagg

1141 gcgcataccc agacggaaac taagttccca ttcacgaccc atgtaacaag ctacaccaag

1201 taagaagtgt agaacaatta gctcataagg accaccattg tataaccatt catcaacgga

1261 tgctgcttcc catattgggt aaaaatgcaa acctatagct gcagaagtgg gaataatggc

1321 accggaaata atattgtttc cataaagtag agacccagaa acaggttcac gaataccatc

1381 aatatctact gggggagcag caatgaaggc aataataaat acagaagttg cggtcaataa

1441 ggtagggatc atcaaaacac cgaaccatcc aatataaaga cggttttcag tgctggttat

1501 ccagttacag aagcgacccc ataggcttgt gctttcgcgt ctctctaaaa ttgcagtcat

1561 ggtaaaatct tggtttattc aattctcagg gactcccaag cacacagatt atctataaat

1621 agaaatagac aacggaaggc ttgttattca acagtataac atgacttata tgtccgtgtc

1681 aaccaataag agagatatct atctggatag atccatccga acgatttgta aattaaatga

1741 gtagggattt atccaataac aaatattttt tcgtattttc cgtacgattg gtaatgggtt

1801 gcccgggact cgaacccgga actagtcgga tggagtagat aatttccttc ttgcaatata

1861 atagatatag agtaaaaaga ccccccaaaa aagccgtgct tgcatttttc agtgcacagg

1921 gctttaccta tgtatacatc caaaactaag ttccctaaaa ggggacctaa gaaacttgaa

1981 gactcagttg attcaaccac tactgtatga acatttcaga attcaaatga ataaaatgat

2041 tttgtgattt tatctcttca tcatttaggg atcctttcta tttacatgac ctcatgacca

2101 atcattaaat gactgactag gtcattgata cggataatat ccaaatacca aatccgttct

2161 ctatgtgacc tatgagaagg agaagaggtt gttgggaaga tcaaagaaag agcttgttct

2221 tcctccgtaa agaattcttc caagaactcc gaacctaatc ttttcaaaaa agcacgtatc

2281 gtacttttat gtttacgaga caaagttcta gcacatgaaa gtcgaagtat atactttata

2341 cgatacaaac tctgtttttt tgaggatcca ctgtgataat gagaaagatt tctgcatatc

2401 cgcccaaatc gattgagaat ctcagaatct gacaaatcgg cccgaaacgg cttactaatg

2461 ggatgccctg atacattaca aaattttgct ttagccaatg atccaatcag aggaataatt

2521 gggactacgg tctcgaattt cttaatagca gtatctattc gaaacgaatt ctctagcatt

2581 tgactcctta tcaccgaaga gtttagtcgt acacttgaaa gatagcccag aaaatagaag

2641 ggatgattat ataattgctt tatatggatc ctggccggtt gagaccacaa gtaaaaatga

2701 cattgccaaa agttgacaag gtgagatttc catttcttta tcagaagacg agcccccctt

2761 gaagccagaa tcgattttcc ttgatatctg acataatgca taaaagggtc tttgaacaac

2821 catagggttt tctgaaaatc gttacaaagc actactacaa gatattctat ttttgcatag

2881 aaatgtgttc gctcaagaaa ggatccaaaa gattttgatc gtaaatgaaa gggttgttta

2941 cggagaaaaa tgaatatgaa ttcacattca tatacatgag aattagagag gaacaagaag

3001 aatctttgat tctcttttga aaaaagggaa atggaatttt ttggagtaat gagactattt
3061 gaattccaat actcgtagag agagaatcgc aataaatgca acgaaggagt atcttgtatc
3121 caagagtgaa gggtttgaac caagatttcc agatggatgg ggtggggtat tagtatatct
3181 gacacatgat ttaaatgtga taacttgtcc tcgaaaaagg gaaatattga atgaatagat
3241 cgtaaattat gagattttgc tatttctttt tcttctaggg aagataccaa tcgcagcgag
3301 aatggaattt ccacaacgac tgcaaaaccc tccgatatca tttgagaatc aaaatgattg
3361 ttgtgcccaa cgaatcgatt ttgattagaa tcattaaccg aaataatcaa acgattctgt
3421 tgatgcattc gagtaattaa acgtttcaca attagtgaac tggatttatt gtcatgatct
3481 aaattttcca ccggttcata aagaatcgat ccatttaaag catgaccatg agcaagcgcg
3541 tagatatatt cctgaaatag aaacggatat aggaagtatt gttgccgaaa tccatccatt
3601 tctaaatatc cttgtagttc ctccatttcc atttgaaatt acacttgaac caaatggggg
3661 atttcttgag ttatcaaata atacatagta cgatacggtc agaacaaggt atatagtaag
3721 aaaagaatag ataccccgga gccagaaagg acaatcaacg gatcctattt ccatccaatt
3781 tatttatgtt cgttatagtt acaagagatg gttagaaatc ctttattttt acaacccgat
3841 cactcttttg actttggaat aatgaatttt gatcagtata ccgtttcttc tacacattcg
3901 tctccactac ataatagaga acataataga gaatagttag gattcattaa aaaaaaggaa
3961 ttgatgatcc actcacaaga gaaccctttc ccacatcagg cactaatata tttttaacgt
4021 ctaattagat cgggtaatca ttcgaattaa gaacaaacag aagctcgttg cttttggttt
4081 ccctataatt ggagctatag ggctctatcc atttattcac tcgacccaac ttgaattgat
4141 ttgacccctt tccaagaaaa gaatcaaaac aagattttgt atcgatccgt taaggatgaa
4201 gtattctaag agttctccat tgatacgaca tgctgttttt tcctttcatt ccctttcagg
4261 atcagtcgtg gtcttacaaa ctccaccaat ggtatggacg aatccgttgc ttcatcaaaa
4321 tgtgtaaaag accatagccg cacttaaaag ccgagtactc taccgttgag ttagcaaccc
4381 atataaatag ggtgtgtaga tacgatcgga ataaaaaata aataaagaga ttcgattgcc
4441 cgacctcgtc aaaacattga actagcaaca gatcaaaaag aaagatttga tgatcaattg
4501 tgaacataaa aatgaacaga gatcagatga aaatacaaca gattctggga taaattatag
4561 agaaaatcta aatagatgta aaaattgata gactacccat actctatttt ttttttttca
4621 ttatattaac taagatactt cttgtgtcac aacgaaattg acgaacccat cgtttgagtg
4681 aaataaagaa acaaacttat gaaatgtgga taaatagatc tatttatcca cgatcgaatt
4741 atatttgttc gatacaccat tgtcaatatg aattgaatgt tgagaaaacc aattcaataa
4801 ataaaacaag gacttgtgtt ggagtgacac tacaacatag ataagggatg aagtatgagt
4861 ggggaaataa ataaggaatt ccggtaggaa aaaaatgtcg ggtttattca atatttattc
4921 aatagaggta caataagcaa gattgacctt ttgtttgttg gtggagtccc aacgaaaacc
4981 atctgattga tggtaatccc ataatttccc agttatttca tctattcact caatcttttt
5041 tctatctgtc tcatatcaag agaagatatt ttttttttat agttctatga tacggggtca
5101 tgtgagagca acaatgaata gagaatagag aaaaaaaata aggaatggtg gagaaacaat
5161 tagacaaagc tagatactgg ccccccccct ttttttttat ttcattaatt tcatttgatt
5221 aattcgaaat tccttaaata cctccgcctt ctttgaaata tcatgaacag ttcctgtagg
5281 ttgagcgcct ttttcaagga aatatagaat agcggaaaca tttgaataag tttggttctt
5341 tatcggatcg taaaaaccca ctttacgaag atctcttccc tctcttcggg atcgaacatc
5401 aattgcaacg attcgataga tgactcattg ggatagacat aaatgaacaa ccccccctag
5461 aaacgtataa gaggttttct cctcgtacgg ctcgaaaaag aatgattcga atttatgtat
5521 tgtagtggca aatagatcca caaaatcatc aattagacta tgatttgagt catttttttt
5581 tgttcttcct tcctgaaaaa aaaaaaaaaa aaaaataaat ctcattcgta ctcataactc
5641 aagttgggta attctcaaag agctcgaagg gaaatcctta gacatttatt gagccgtctc
5701 taacctcttt tgtttgtctc gcctagaatc gatttgattt cttccccatt ctgatctagt
5761 tgttgagaca attgaaaacg gtgtttcctt gttccggtat cctttatttt atctttgctt

5821 tgaatccttg ggtttagaca ttacttcggt gatccttaat tgtttcaaaa tggtagcaac
5881 ataccttttg ttatttcgtt ctatggaaac gattgattcc cctgtgatac acttttgatc
5941 ggaattggta aaactatttc gacaaattca ttttactttt ttttttact tgttcgaact
6001 tgatcctttc aatttctata ccgaagatat acttacgaag ttgttccaac ttattgattg
6061 gcattaaccc tagatccttc tctctgctaa atgaaccaat tctttatgct cgagctccat
6121 catgtgctat attataatta tattatttta ttacaacccc aaaattgggg tcctagtgga
6181 atagaacaaa ctatgtcgag ccgagagcat cttctttgat atataaaatg gtgggtacaa
6241 gaatccacag ccaataatgt ccttcaagtc gcacgttgct ttctaccaca tcgtttcaaa
6301 cgaagtttta ccataacatt cctctaattt tggaccggta tggaattgat tcaatatgga
6361 atcatgaata gtcattggct caatcggtat atagtatatg aagtcctcca tactttcatt
6421 ttcatatatg gatctggaga agtctcagca agaatataat ttaaccccat attttattag
6481 aagaatgaaa cacatttata aaaaacacga agaaatgcgt tgcttaacac ttctttacat
6541 cttcaacaga ttgttaggga tgatgaatca tgaaagatcc aattctttct caaacaacta
6601 aaactaaaga agggtctaga ttaccgatac cggaacagaa tgagtcatta accaaataag
6661 ctattccaat gaccgggaaa gatcgcaagt gactcgatag gatagattca ccaatgtcac
6721 acttcttcga ggagaaagaa tttataagga atcataaaaa acaatttcaa gaatttccta
6781 cttcgacatc attactggaa ataagttttc cagtaaagac tgaattgaat ctctaaatcc
6841 atcaacaagt cggtacaacg aggatctaaa aatgtttagc agatatctga ttaattaaat
6901 cagacgcgaa tttgatcatc ataagagacc tctatgtttc ctttccgatt gaatcatcaa
6961 taatttaaac cagataaatg ggtcaagaaa caaatccaat tgttttgttt tcttggggat
7021 ggatagaagg ggctcatgaa agaaaagatt aaggtcggta ttctttcagg tattctttca
7081 tctgattgat aaaatcagaa tcgtaggagt ctgattttat cgtattcatc agatacacca
7141 aacaagtgtt accgggggta atcgtgggta tttaagggat cgcagatctt tttcgccaaa
7201 actgaaacac cggtgtcact gatcactgaa atagaacaat aacaatatat ataggcatgg
7261 ttcattttct acagattttt ccttacttca gattcaggaa tctttccata aagtaaagtg
7321 agtgtatgaa tctccccccт cccccaattg atcgctacat tgatttccaa ttattcattg
7381 gagccaaatc aaatacaaaa taaaagaaat taggtccaaa gaaaataatc cgttccgtat
7441 tgaattttct tgtttgttaa taagatccgg atgacaaggg tcttgaaatt gataccttcc
7501 tttcctttgc ggattaactc atatcgacac gaattccatg gggatcatga atcataccca
7561 aagccaattt aaaaggatct tcttatggga tgctatcctg tcttgtataa gtacaaagca
7621 aaatgggttc atatcaattc gttgttacta ttttgtttga ttttgtccca ccccagtctc
7681 aggagcttta attccagcga tggaattaat accaagaacc cccccgtttt ttaggattca
7741 ggatccacat agaattagtc attttgtcta ccctatcttt atttatattt actttaggga
7801 aagtagagac ttctctttta tttcgcattt cgactcagaa tgcatcatgt gagaatccaa
7861 atatcataga tatggggcat aaagtttatc caaatgacta actaaccttc aatatgaata
7921 tgggcgaggg ggcgaacata cgctgaatgg cccacccagt tttttttga atttcacttt
7981 gatctttgtt cccatcctac ctatatcaaa aaagatattt atttccatcc acatgtcatt
8041 gatactcgat ccgtttgttt gtgagaaaca aaatcgaaag ggaagatatg attctataga
8101 agaatcatta gaaatcacaa agaaagattg gatcacattg tatccaacat tacaccctta
8161 aacagaagat tgttaaaaag aacaatcttt gttatgatag aattggtctg ggacggaagg
8221 attcgaacct ccgagtaacg ggaccaaaac ccgttgcctt accacttggc cacgccccat
8281 ttttatttct attcggcacc aataaacact aatatcggta ttggttgttc gtcaattcca
8341 gccccaatat ctattgaatt ggttgttgct atgattctac acatgtagat gtagaatcaa
8401 aatgaattta ttgatcatta catataattc aattaagata ttgtatgtaa ggtatgattc
8461 cttctattct catttgagaa ttgaaggatt tttgattgag ggagttcaaa gaaaaagaaa
8521 gattttgcgg cctactttcc cttctttctt cattttcccc ttatatcaat aacccaataa
8581 taatgaaatt ttctccaaga acaaaatgtt tgttatgctt aatatcttta gtttgatctg

8641 tcttaattct gcccttcatt cgagtagctt tttcttcgcc aaattgcccg aagcttatgc
8701 ttttttcaat ccaatcgtag atgttatgcc agtcatacct gtgctctttt ttctcttagc
8761 ccttgtttgg caagctgctg taagttttcg atgagatctt taatactgtc ttagaaacat
8821 tcatgattta ttcgataaaa aaaaattcta ttcttaagaa ttgataagat cagataatga
8881 accctcgact caaacatgga aattcttttg gataaccgag atgaatcgga atcacctcat
8941 ttcttcattc cttctggggg tcgaagaccc tatgtatggt ccctacaata cctaattgta
9001 ggtatgagag atcattttgt taacgaaaga atcagaatct tattacaaat gcattcctgc
9061 aaattcctta tgttttctag aaaccgcttc ttttcttggt gtcaaaacgg aatatgtggt
9121 acaaaaatgg agaatctatt cccctatttc ccccaaaatg atcttggaga ttgtgtaatg
9181 cttactctca aactcttcgt ttacacagta gtgatattct ttgtttctct cttcatcttc
9241 ggattcctat ctaatgatcc aggacgtaat cctggacgtg atgaataaaa aaatcagggg
9301 tttttccttg ctcgatttct gaattttctt aggattttct ttctccattc catacattta
9361 actatgagaa agggggttag agatttttc gaattcgaaa gggaaatatc aagtgatcag
9421 aagaaacgga gagaggggga ttcgaaccct cggtacaaat aattcgtaca acggattagc
9481 aatccgccgc tttagtccac tcagccatct ctccccattt taaatggata attcatatgt
9541 gacgcgtgaa gtaaaggttg ataaaagttt ttccttatct ttctttattc tataaatata
9601 gacgaatttg atcaatcatc aattcccttt agataatgat tcgaaacaga tatctccaat
9661 agaaagagta cctctttgat ttcgtccgaa aagttctttc ttttattccc ccggcctggc
9721 cagtacctag ccaggccatt ccttgttcca atgaatcata gatcaaatga tttatttgat
9781 ttgaaaacga aaatgcttgt tattgaagca gcaacaaggc tatttccatt cctatgatag
9841 gagtgtcatt tgttattatg tttttccttt tctcgattta cttaaatgga ataaaaaaaa
9901 catatttttt tctattataa tagaactcat atatttcttc aaagaacatg tttgaacctg
9961 aacccttgag tccacaacca aaacaatagg atttttcact cgatccaatc gacccgaatt
10021 cataagattt ggcagttgaa tgaataggaa aaggagtagc ttcgaaaaag aaaaatggag
10081 ctctggattc ttgtacaact caactcattt ttatgttccg acttcaatgg ctctttcggg
10141 ccgggactat cagtaacggc tccccgataa aagcttgtta ttgaaatgaa cctccttctc
10201 ctattttatc aagtctcccc gtcagagcac aacatgtcag caccccaatt ttcatgattc
10261 tgatcctatc ttgattacgt ttcacgccct tgttcgacaa atggcccgct cgtatacaat
10321 aattatattg tagcgggtat agtttagtgg taaaagtgtg attcgttcta ttaacaagtg
10381 aaatagataa gggatctttc gtttgattcc tattctgatc aaaaacttta tttattaaaa
10441 gggcattaat cccttacctc tcaatgccac atttgaggaa gaatatacat tctcgtgatt
10501 tgtatccaaa agtcaagtca attagaaatt gactaacaaa attggattat ggaattgcga
10561 agcataattt tttttttga agttggatca accattccaa ttgaatgagt ataagtaagg
10621 gatccatgta tgaagataca aaagtctatt tctaatcgta actagatctt ccattttttt
10681 tttttagggg gagattgaag ccaaatagct attaaacgat gactttggtt tactagagcc
10741 atcgacatat tgtttcagct cggtggaaca aaaaaattct tttcttcagg attcttgcaa
10801 gtacaaatag ggaacgaagt aactagaaag atttgtgaga atcctcctct ttctagaggg
10861 atcatctaaa aagcaagtca tttggggtgc attcagacga aaaggctgac atagatgtta
10921 tgggccaaat tgatttcttt gaattcagat ttgctatgac tccctttcc catacatcgt
10981 aaattttta gtttttttta tgtcttagat ttgggaatcc cataaaggag ccgaatgaaa
11041 ccaaaatttc atgttcggtt ttgaattaga gacgttaaaa atgatgaatc gacgtctact
11101 ataaccccta gccttccaag ctaacgatgc gggttcgatt cccgctatcc gcttcatatt
11161 aattattata atacatgctt ttgatatgtc cctaaaatct ttctttcaca tacaatccta
11221 ttcctttttt ttataggaga taggaaagtc agaacgtgaa agaaatcgga atgaaaagcg
11281 tccattgtct aatggatagg acagaggtct tctaaacctt tggtataggt tcaaatccta
11341 ttggacgcaa tttatttcca tctatttttg tagattgcta tgtcaagaaa catattttga
11401 atgattcgaa tcggggccat ttctcaacga ttcgtcttgt acttaagagt gatcaatttc

11461 tttatttttg ttcctgaagt agaaacagtt ctatctgttc cggaatagct tccttcaaaa
11521 gggcttccgc ttgcgcggta aatgtcttgg tagaagatat gatttcttgg acctgaggtt
11581 tatttgtttt taagtaggta cgtaactgaa cgagaaattt ctttacctgt ccaatttcta
11641 acggatcaag atacccattc gctccagtat aaatagtaac tatctgttct tccaccgtga
11701 gaggggctga ttgggattgt ttgagcaact cgcgcaatcg ttgacctctt gccaattgat
11761 tctgagtggc tttatcgaga tcagaagcga attgtgcaaa ggcttctaac tctgcgaatt
11821 gagccagttc cagtttcgat ttgccggcta cttgtttcat ggctttaatt tgagctgcag
11881 atcctactct cgagacagaa atacccacat taatggcagg acggattccg gcattgaata
11941 gatcagcgga taagaatatt tgtccatctg taatggaaat cacattagta gggatataag
12001 ccgaaacgtc cccagattga gtctcaacta ttggtaaagc ggtcatactt ccttcaccta
12061 aacgagaact tgatttagcg gctctttcca aaaggcgtga atgcaaataa aaaacatctc
12121 ctggataagc ttcgcggccg ggcggtcttc ttaatagaag agacatttga cgataagctt
12181 gtgcttgttt ggagagatca tcataaatta ttgaagtatg tcgttcacgg tacataaaat
12241 attcagccag agccgctcct gtataaggag cgaggtattg taatgtagca ggtgaatccg
12301 ccgtttcggc taccacaata gtgtattcca ttgcccctcg ttcctggaaa gtggtcacta
12361 cctgggccac ggaggatgct ttttgaccaa tagctacata aacacatatt acattttgcc
12421 ccttttggtt gagaatagta tctgtggcta ctgctgtttt tccggtctgt ctgtccccaa
12481 taattaattc tcgctgaccg cgtcctatag ggatcatcga atcaatagca ataagtcccg
12541 tttgaagagg ctcatatacg gaacgtctcg aaataatacc tggagcagga gattcgatta
12601 accgagattc agaagctgaa atttcacctc tcccatcaat gggtttagcc agagcattta
12661 taacacgacc caaataagcc tcactaacgg gtatctgagc aattcttcct gttgctttta
12721 cagaacttcc ctcttgtatc atcaaaccgt cacccattaa tacaacgcca acattatttg
12781 attccaaatt cagagcaatg cctattgtac cctcttcaaa ttctactaat tcccctgcca
12841 ttacttcatc aagaccatga atacgagcaa tgccgtcgcc tacctgaagt actgtgccgg
12901 tattcacaat cttgacttct ctattatatt gttcaatacg ttcacggata atattactaa
12961 tttcgtcggc tcgaatggtt accattagtg tctcttaatt ctttttcgga aacaaagaaa
13021 aaaaaaaaaa aaaataatgc cttcagtaga agggctaatc agttacttct ttcatggccc
13081 cgagcatgcc aatattagca ccgatggtgc gtaaatgtaa ctcgctgttt gaacaactat
13141 tcagagttcc tagagctcct tgtaaggctt gttggaaaac tcgttgtcgc acctgattaa
13201 ttgctctttg ttgttcaaaa tgaatggttt catttttgta attttctaat cgttccaaat
13261 tctcataagt ggcattaatc aaattccatt tttctcgttc tatctcagag tatccattca
13321 ctcgaaactc atctgcttct atttccactt tccgtaagcg agcccgggct tcttcgagct
13381 gctcaatggc tccttcacgt agttcttctg aatttcgaat agtactcaag atcctctgtt
13441 ttcgattatc taataaatca cttaatgaaa gtagattatc ttcccattca tttcacaact
13501 tcatttccat gatctcttcc cgaaccaaac atgaatcttt cgattcattt ggctctcaca
13561 ctcagttact taatgggtca ttccatctat tttaatgtaa tgagcctacc ctctcttctc
13621 tgttcgtatt ccaagatatc aaaactgata cgagaccaga atattcggag gactcttccg
13681 acccgacaaa aaatctgtca ttgtcagcaa agttgtttct tttttttgt tttcttcaaa
13741 tccaaaaaat tcttcttatt ttagacatag gtcatcgatt caacattgga taaaaaaggg
13801 cgagacacct atttttacag taaatggttc aaatcatttt atcgatatga gtgttctata
13861 tcggataaat tgccaactat tcattttttc gaaaccatct ccgtactaac gtagtggtag
13921 aaagagtacc atgttgtgcc tggacttcaa acggtttcgc tttaaccatg ttaaaggtcc
13981 cacattattg gctgatagag aatcaaagtt gatttaccaa taaattacga aatgctatgg
14041 ttcttacata tgatttctta atttattcag aagtaattcg tcgagatcgt gcacctttct
14101 ttcctattta taactttccc attcaaaaaa aaaaaaaagg aaagtgcagc cggttggatc
14161 cagcctattc ttgaaataca caactcgcac acactcccct tccaaaaaag atcaatacac
14221 caagcactac acttagattt attagatttg ttgctaaaat atcggtatta aacccgaaac

14281 tcccagcgga tggccagtga ccaaaggaaa cgaaagaatc ggttacatct ctcatatgct
14341 tttctcttat agataggacc aacaaaatgg aacagagttc tttttgtatc acttcgcccc
14401 ctttgttttg gattgatttc tttttattaa tttacttatt ataaatatga atagattcat
14461 tttaagaaat atttttcttt attattattt ccatggaaat tctcaataat ctatttattt
14521 agtcccaggt ttcatgtcaa ttacgaaata cctcgtttgt tgcaacactt cctaaaagtc
14581 aaaaagagtt tccattaaga acggaaggga agaaagcaag tgggtctgct atgctaattc
14641 ctcatcctca aatcactcca tccccggggg tattgtctca acgaagaagt gattgtagga
14701 gtgaagtttg gatataattc ggcaaggcaa gcccgcggca ataaaatagg aaaagaaaat
14761 aagtatttat ttttcacatt tataggatta aacaaaagga ttcgcaaata aaagcgctaa
14821 tgccacgacc agtccgtaaa ttgttaaagc ttccataaaa gccagactaa gcaataaagt
14881 acctcgtatt ttaccctctg cttctggctg tctcgcgata ccttctacgg cttggcccgc
14941 agcagtacct tgaccaactc caggtccaat agaagcaagt cccacagcca atccagcagc
15001 aataacggaa gcggcagaaa tcaatggatt catattaagt tcctcgcacc aaaaaaaaga
15061 aatggttaat gatacaatca accgatgaat tattacttca ttattccatc acttaagatc
15121 tatccgaaaa aaaaaaagaa ctaagaactc tgaattgaaa taataatatt actgaatcat
15181 cagagctact tcgatatctc gtttttagtt cctatccgtg gagtctttgt aaatctatac
15241 ggttccagtt cttccatttc tttgttccga accattccat tctttcaatt cttcgctctt
15301 tctcttctat atgcatgctt gacttcattt gtttattcat tcaatccaca gtcacagata
15361 aaacggaagg gcttgcattg gaatccgtct aaattcagtg ggatgggaaa taatatatat
15421 ggatagccca tatataacta gtgaatatct aatatcacat atacattgtt tctttaataa
15481 cgtaaaccat ccgtaccttc tattgaaccg gattctagaa tcattcttcg aaacatatac
15541 agggattggc ttagagccct tacatatacc cagctcgacc ccccttactt ttttttaatt
15601 ctctaatatc atacattttt tttttgctcc tattctagat cgtatatacc ggtatgagtt
15661 gggataagct tttttttaag accatttcgg aagctagtca atgatgaccc tccatggatt
15721 cacctatata agctgcggct aaagttgcaa aaataagagc ctgaatcccg cttgtgaata
15781 atccaaggaa catgacaggt ataggaacca ccgaaggtac taaagaaaca agaacaacaa
15841 ctactaattc atccgctaat atattcccga aaagtcgaaa actaagtgat aagggttttg
15901 tgaaatcttc tagaatgtta attggtaaaa gtattggagt tggttgaatg tatttaccga
15961 aataacccaa tccttttttg gtaagacccg catagaaata tgccactgac gtaggtaaag
16021 ctaaagcaac agtagtattt atatcattcg tgggtgcagc taactctcca tgcggtaact
16081 gtatgatttt ccggggtaaa agggcacctg accagttaga aacaaaaata aataggaaca
16141 tagttccaat aaagggaacc caaggaccat attcttctcc aatctgagtt ttgctcaagt
16201 ctcgaatgaa ttcgaggaca tattcgaaga aattctgacc gtcggttgga atggtttgtg
16261 gattccgaac agctatagtg gctgaaccta ataagatagc aattacaacc caagaagtga
16321 taagtacttg ggcatggact tggaaacccc ctatttgcca ataaaaatgt tggcctactt
16381 ccacatcgga tatatcgtat aacgctttta gtgagttgat ggaacagggt aaaacattca
16441 tattgccctc tgacataaat agaacttaaa aaggaattat tttgattcag ccatctcgta
16501 tctctttctc aactcgtcta ctttgaatca atcgtatatt tcggatccca agtgatcaca
16561 taatatcccc agtgattttg atctcttttt tgagactcag ggatagtaac cgattcaatc
16621 aatttatgga gtttccaaag tgattgactt atcctaatca acaatttctt atatagctag
16681 aactgccctc acaaattgcg gatactaatt tgttaagaat ccatcggatt gaagccatag
16741 cgtcatcgtt cgctggaatc ggaatatttg cgagatccgg gtcacaattt gtatcgatta
16801 aacaaattgt tggaatcctc aaagtgagac attctcgaag agccgtatat tcttcttgct
16861 gaccaacgat gattacaata tcgggtaacc ccgtcatata tttgatcccg cccagatagg
16921 tttgcaagtg agataattgc ctcttcaaca ttgctacatc tcttttcggg agacagttga
16981 gtttccccgt attttgttcc gatctcaagt tcctgaacct atgaagtctc atttctgtag
17041 tggaccaatt cgttaacata ccaccgagcc attttttatt aacataatga caccgagccc

17101 ttattgcagc tgatgctact aaattggctg ctttattttt ggtaccaact attaagaagt
17161 gttttcctat acttgctgca tcaaaaacta aatcacaggc ttctgacaaa aaacgagcag
17221 ttcgagtaag atttgtaata tgaatacctt tacgctttga agagatgtaa ggtgccattc
17281 taggattcca tttcctagta ccatggccaa aatgaactcc tgctttcacc atctcttcaa
17341 aattcatgtt ccaatatctt cttggcattt ctccccacat tttctctctt ttttttatt
17401 taagaggtac ccctgaaata aataattgtt ccgacggaac cttctaccgg agattgaccg
17461 ttaatacccca gtccaagtca ttaattcctt tctattcgtt attatcttta ttaccaaatc
17521 aaatgaccag gaccctatag ttaaaagaaa agaatgaatc tgtcattaaa tcccgtaaat
17581 gatcgttctg atgtatcagg gaaattattt gggatgcaag aaccaaataa ttctctgtgg
17641 tggaacaaaa gatctctcat ttcctcctcg aatggattct tctttttgat ttccaaagga
17701 atgttgctgt gttgccttga gcggtgcact aatcctttga atccggtacc aacaggcatc
17761 atccccccca gaacaacgtt ttctttcagg cctttcaacc aatcaatacg acctcgtaga
17821 gcagcttttg ctaaaactcg agcggtttct tgaaaactcg cttcggatat gaaactttga
17881 gtattcagag atgccctcgt tattcccaat aagatggctc ggtaacagat cgcttcttcc
17941 aaagcacgcc ctgttcgttc cgctcgcaac aatccgatta gttctccagg tgaaaaaaca
18001 ttagacattc catcttctga aaccaacact tttgatgtta tttgacgtac aataatctct
18061 atatgcctat tatggatctg caccccttgg gatcgataaa ccttttggat cttattaacc
18121 aaagagatac gactttgcgc tatggttagt tcagcgccaa tcaagaatcc ccaaggaatt
18181 ccaaggattc ttgttatacg ttcgttccaa ccctcaaccc tcttttctaa gttcatcgat
18241 attgaatcaa tcgaacgcgc ttctaacact tgttccactt ttggaagacc ttgtgttata
18301 tcacccgatc tcgatttttc atatataaat gtaactaatg tatctccttc gtaaaggatt
18361 tctccacaat ggccatgaac agttgcccct ggcgtagcca aatggggctt agcggatctt
18421 atgactaagg agtcaacatg aacaattaga acttgactcg attttatgtg tggtccgcat
18481 ttggatatac atacattttc acaaataaac tgtccaaggc taattattgt ggatgtctcc
18541 tcacaataat cgtgagggag aaagcaccaa ttcaaatcga atggattaaa aatgatgtta
18601 ctgcatgaat cgggattata aattctccca ttttcatcca ttaaataata tttaagccct
18661 tggaaagtgt gtttgaaatt gtcaagtaga aaatatttct ttaacaagat ctgattatga
18721 gttattaaat agaaatagta aaatgaataa aaattcgtaa tttgaggtac aatccctaaa
18781 ggacccaacg aattcctaat gggaatcgcg ggatcctctt taattaattt aattaattct
18841 ttgtgagatt tcgaaccatt gaatagacca attcgagaac aatcggatga taacaaaatt
18901 agaaaagatg gacattcctt atttctattc agcaacgtac gaatagtccc ttgatgttgg
18961 gtaaatgatt gaatcctcgc cttgaaataa aaaggattaa tattggtgcg atctgatcca
19021 ttattggcaa tcaatcctga acttgccata tcattccttt ttccgatata aaaaatagag
19081 gacttcacta aatcaattct tatgaaatct cgaatcagat catttgccct tacttcaaca
19141 aaggaagcat gaacctcttc tatagaacca tctcggtctt ggttccaatt caatactaaa
19201 caagtccgaa ctaattgaag acttgtgtga taaattcccc gaatcggttt gccatttcca
19261 taaaggatat aattgacaat tcgtagttgc acattatccc tttcctgcaa cggatcctgg
19321 gggaaaagcg ttgctaaatt tatcccatca gctatttcat atgtgactac gggtcgaact
19381 gaaacaaaat actttttctt ggtaggtgta atccgttgga cataaatcca atttttcaat
19441 ttttttgatt ccttagaatt tttttttccc gttcctggtg gtatcaagat gccgcagtgc
19501 cgggatatct tatctgtcgc tccaggaaaa tagatatccc cagaaaagat tttgagttca
19561 atcctttttt tttttctctc cacccggacc aatccgccta cttggcttct tgtatttaaa
19621 gcgattcgtg tatctactcc aatgatactg ttgttccgta ccattatggg cgaagaaccg
19681 ggtaagatat gcacttcttc gggaatgaaa aaaaatcgat ctactttcat ttggtatttt
19741 ggcctaaatt cttttgctct tcgatactca atcaaatcct cttttttttac gattgaaccc
19801 acccctatag tcccatattt agtaattcct gaactgcttc ttctgtatag gggatcgtcg
19861 aaataagcaa gaatactatt tctaggtaaa acaccattta tgggtatttc aatcgagata

19921 ccggaacggg gcattagttc tttttctcgt tcttgatcag attggaatgg gatgataaat
19981 ctatttcttc gcctcttagc caataaatca gaattctcgt ggagaatggt aggatatatg
20041 aaattccaat gaccgttgga tatgattcga tcaggtcctg aataatcaag aacccccccc
20101 tttttaccgt aaggattcga actaaacaat ttgtgtctca cttgatcatt agtcacggag
20161 aggtcagaaa tagatctccg ttcaacagaa tgaacattca tttgatcttg atccttgtgg
20221 agcgaaaaag gcactatact ggatctgcac agagctcctg ataatatcca taaatgactt
20281 gttttgggta agagatgaac attaccatat ttatattcag gtgcatggta cacatcggta
20341 ctccagtgca tttctccctc tgagtcagaa taaatatgtt ttcgaacttt ctctttaact
20401 ttattaaaat tgaaagtgga tgttccagcg cgaatctcag caatcacttg ttctgattct
20461 acatattgat cattttgaac taaaagaaaa cttttgggtg gaatattcac attatgtaga
20521 atatcgtgac tctcaatagt tacatacagg tctatataac atagaaaagc aggatgccca
20581 tgacgtgtac gtgtgggatg aaccaaatcc tcattgaatt tgatttttcc cttaaaagga
20641 gctcgtacat gttctgcagt accacctgtg aatactccac cggtatgaaa agttcttaat
20701 gttagttgag tccccggttc gccgattgat tgacccgcaa taatacctac tgcttctcct
20761 aattcgacca ggtcgccatg agtgggactc tgaccataac ataatcgaca gatccaagat
20821 atactcctgc aaagaaaggg ggttcgaata tatattggtt gtgttcgaaa ggttatgaat
20881 cgagtgacaa gtccaacccc aatatcttta ttttgagcgg caatgcaacg tggacccata
20941 tatatattgt atgctaatac acgaccaatt agtgtttgga cccaaattct ttccgtcatc
21001 ccatttctag gactcacgga aatgcctcgg gtagtgccac aatctgttct acgtacaaca
21061 atgtgttgaa ctacttcaac aagtctacgc gtgaggtatc cagcatctga tgttcgtaca
21121 gcagtatcca caactccttt gcgggctccg tagcaggaaa tgatatattc cgttaaagaa
21181 agtccttcgc gtaaattgct ttgaatcggt aaatcaatca tttgtccttg gggatccgac
21241 attaatcctc tcatacctac taattggtgt acctgagatg catttcctct agctcccgaa
21301 aaggacatta tatggactga attagaagga tcagtcatcc taaaattagg atgcatttct
21361 tgtctcaaat attcacttgt agcataccat atctcaatgg attggcgtaa tttttctact
21421 gtgtgtacat tcccataatg atggtgcttt tctaaaatga aactttgttg ttcagcatct
21481 tggactaacc accccttaga aggtactgtt aaaagatcat caattcctaa tgaaatggat
21541 gtagcagtgg cttgctggaa acccagagtc tttacttgat ccagggtgtg cgatgtatat
21601 gccattccga agtgatctat taatctgcta ataagtcgtt tcatggcaga cccatctatc
21661 gctttattgt aaaagaacag atcagcccat tctgccataa gtacttctct attccgctga
21721 gtaggattcg ccaatgggtt tgagtcagtg attcgaagac ctcctttact ggatctcgat
21781 tcatgtagaa attaaggaat tatgattcca gttgaaccgg agagatccaa attcccgcgg
21841 tattacataa ttccttagct taggtaccgt atgagtaggc ctgacaaaac ccctgtatgg
21901 cttcttctat ttctcgagaa aaagaaatag gaccaacagt ggttcgggtg tatatacaaa
21961 gggtttgttt ttttatacgt cttactatta gatagtgccc ataaatttca tgataggtac
22021 ccaaagattc atattgaact tcgacaggaa cttctcttga ggcaatgaca cgttgatcta
22081 gtcgccaccg aagccacaaa ggactatcta aattgattcg tttctgctga taaactataa
22141 gtacatcata ggaactacaa aaatagggct ctttctcttt cgtagtatat ttatacttat
22201 aatcattaac tgtttcattt tgatagttta tgcgattcca tggattatac ctatttgcac
22261 aaatacctcg acgattcccg atcgttaata catagagtcc aataagcata tcttgggttg
22321 gtaccgaaat gggatctcca atagccggag aaaagagatt catatgagaa aacataagta
22381 aacgagcctc cgcttgcgct tccaaagata aaggtacatg aacagccatt tgatccccat
22441 caaagtctgc attgaatcct ttacgaacta atggatgtaa acaaatagca cgtccacccc
22501 ctaaaatggg ctggaacgcc tgtatgccta atctatgcag ggtgggcgct ctattcaaca
22561 atacaggatg cccctgcata acttcttgaa gtatttccca tacaatgggt tctttttccc
22621 gaatttgact tttagcaatt cctatgttag aagcgatatg tcgtctgatt aaaccacgaa
22681 tgacaaatgt ctgaaaaagt tctattgcta tttctcgagg taatccacat cgatgtaatg

22741 aaagcgaagg gcccacgaca atgacggaac gccccgaata atcgacccgt ttaccaagta
22801 gagtctcacg aaatcttcct tctttgcctt caattacatc tgaaaatgac ttgtaaactt
22861 tattatgacc gtctctcatt ggttgtccgc ggatcccatt atcaagaagt gtatccacgg
22921 cttcttgtac caatttctcc tgacacatta ctaattcccc tggcgtagat ctacttgttg
22981 ttaatgaatc ggtaagagta ttgttccgat agataactct tcggtagagt tcattaatat
23041 ccgaactcat tagtttaccc ccatctatct gaatgattgg cctcaactcg ggaggaagaa
23101 ctggtaatag gcacaaaacc atccgttctg gttctacatt tgttcgaata aaatgcttag
23161 ctaattccat gcgtctaacc aaaaaatcct ttcttcttcc aatttttcta tcttcccatt
23221 cattcccagt gaacccttct tcccctaatt ccttccattc taccgatgaa tgatctataa
23281 taattcgcaa atccggatcg gctaattgtt ctctgatagc acctgctcca gtagagattt
23341 ctcgatttcg aaatgtatcg aagcctgggg tagtaaaaaa aagtgggatg ctgtatttcc
23401 gggattggat ttcagattcg aatgaacctc gtaatcgcaa gaaagtcggt tttttagcta
23461 tgggcctggc aaaagaaaaa ttgggatagg ttcctatagg atccccccctt caaaatcgga
23521 cgtgatggtt tcctctcatc cggctcaagt agttacacca aataaagaaa ggggttctcc
23581 actttcaaat tttgttctag aaaatcccag aaagatctac tccttactca agttcccagt
23641 gaggaccaac aaaaatattt catggattca tccttccttt gacttttctg aattacttat
23701 tcaattacga taaaaatgga atgtcaaatt attgagtagt ctacttccct tcgaatgata
23761 aatcccctta aatgaaagga ataccttgga attaataagg gatttacttg tctatgtatt
23821 gtttcattcg atcttttagg tctccacttc acctcgacgg ttatgccata atgtcccttg
23881 aagcatatat gcgatggatg ggctcctgta accgtgtcat atttgtttat ttgaacagaa
23941 tctctttcta aaagaaatag aacgtctaat tccacgaaag aagttttttt tttcacgagg
24001 tacaactgtt atatgttacg gaatcgacca tggatcaatt cccctttcat ttggaagtat
24061 tgaatacaac cataattctg agcttcatat tcctcctccc aagatacatg tcagagtcag
24121 gggcatcccc atcgaattga atgggatgac agtttctcat tccgaatctg taaaatccta
24181 atttcgatca aatcacacat cgcagtatac taggccttct aattccttaa ggggtttatc
24241 taaaagattc gcgatataac taggaagacg tttcaaatac catacatgag tcgctggaca
24301 tgccagtttg atgtatccca tttgatatct tcgtatccga gaatcaacaa attcaactcc
24361 gcattgttca caaaatttcg gatcttcttt ttcctctccg atcactcgat aatttccaca
24421 agcacaaatt ccgcttttta taggtccaaa aattctttca caaaacaatc catccttttc
24481 cggtttattg cttttgtaat gaaaagtata gggttttgtc acctctccaa ccatctctcc
24541 attaggtagg attttggtgg cccaagcact tatttgttga ggagaaactg atccaactcg
24601 aagttgttga tgtttatatc ggtcgatcat agaagaaaaa ttctgattca tttcgatcaa
24661 acttccttcc tgttaatctg gaagttcttc tcagatacaa ggaaatgatt cagttccaga
24721 gccaaagatc gtagttctcg aacgagcaat cgaaaagatt ctggcgcatc ctcgggttta
24781 agtattgttc cgccaatgat tgtagttcca agtacttcct gacgagctct aatatgatcc
24841 gatttataag taagcatctc ttgtgaaata tgagcaacac caaatccctc tagagcccaa
24901 acttccattt ctcctactcg ttgtcccccct tgcttggccc ttcccctaag gggttgttgt
24961 gtaacaagtg cataatgccc gctggaacgt ccatggattt tatcatcaac ttgatgaatt
25021 aatttcagga tataggactt tcctattata accggttgtt caaaaggatc tcctgttctt
25081 ccatccaata ttctgctttt tcccggatac tcgggttcaa atacccacgg atttgctgtt
25141 tgcttactgg ctgaatataa ttcaggaaac actagctttc tcgaagcccc ctgctcatat
25201 ctctcatcaa agggtgctat tctataatgt ctgtccaaca ggtaccccgc taaaccgagc
25261 gaacattcaa atatttgtcc cacattcatt cgcgaaggta ctcctaatgg gttgaagacc
25321 atatcgaccg gtgttccagc ttgcaaataa ggcatatcct gtctaggcaa aattttggaa
25381 acgataccct tattcccatg tcttccagct attttatcac ctactttgat ttcacgtttc
25441 tgtaaaatat atacacgaat cgtttctgga ttataactgg aaccccccctt tttctggatc
25501 catctcacat caataactcg accccttccg cctataggta gttttagaca agtttccttt

25561 gcggtggata cctgaatgcc aagtatggct cgtaataatc tatcttccgg ggcatacgat
25621 gattctttcg ccgcctgagg cgttaattta cccactaaaa tatcacctct ttctatccaa
25681 gatcccagca ttacaattcc atttttgtct aaattgcgga gtaaatgggc ttctaaatgc
25741 ggtattttat tagtgattct ttcggggcct tggcttgtca catgagtctg aatttcatat
25801 ttccgtatgt gaaaagacgt ataaatatct ccatatacca gacgttcgct aatgagtacc
25861 gcgtcttcag aattgtagcc ttcccatgac atatgagcta ctaatacatt ttttcccaaa
25921 gtaagttcac cgccaactgt agccgcgccg tccgctaaaa tttgtccctt tttaatgcat
25981 ttaccccgcg gagcctgagg tttttgatgc atacaagtat ttttgttgga acgttgatac
26041 ataaccaacg gaatgcttat agtgtctcca ttacccgata aaacgatctt gtccgtatcg
26101 gtataaatga tctttccctc gtgttcggct atagccgaaa cccccgaatc tagagccgct
26161 tggcattcca acccagttcc aacaatgcac ttctcggatc gagaaagcgg aactgcttga
26221 cgctgcatat tagaactcat taaagcccga tttgcatcat tatgctcgat aaagggaatg
26281 agggaagctc caatagaaaa atattggaag ggaaaaatgc ttcgaagatg aatctgttcc
26341 catgcaatag tcaagaattc ttgacggtat ccggctggaa caacctgttc ttcctgaata
26401 ccccgattca aagccaaaga atttcctgcc gctaccatat agtattcatc tctacttggt
26461 gataaataaa ccatcttttt tgatctctca gatatttcat aaaacggact ctctatagac
26521 ccccaatgac caatcctcgc atgaatagct aaggatccaa taagtccaac attgattcct
26581 tcggacgtgt caattgggca aatacgtcca tagtgactag gatggatatc tcgtatccga
26641 aaactagcag ttcgccctgt taatcctcca ggacccaaat aactcaattt tcgcccatga
26701 actatttgtg tcaatggatt agttcgatcc aaaacttgag ataaagggtg taggccgaaa
26761 aacgattcat aagtggttgt taatggagtt ggagttacca aattatgagg agtcggtatc
26821 aatttatgcc tgattgctcc acatatagtt cctcgaaccg cattttctaa acgaaccaga
26881 gccaatccga attgatcctg taacagatct gctacagaac gaatacgttt atttttcaag
26941 tgattcatgt cgtcaagtgt gcccattcca aatttcattc cgatcaaatg atccacagca
27001 gccaatacgt cttgtggtaa caagaatgta ttgttcggag gtatatcaag attcagtctc
27061 cggttcatat ttcgtcgacc aatccttcct aattcacatc tttgttgaaa aaatttcttt
27121 tgtaattcct tacataagga ctcagaaaat accggatccc cgcctacaca agcaaattgt
27181 tgataaaact ccaaaatggc attttccctt gacccaatct ttttttctc cttatgattc
27241 gggaaagaca agaaaatttc agggtagcaa acattatcta gaatttctct tagattcgaa
27301 cccatagccg atgatggaac taggatagat attttttgtt tcctactcac acgggcccat
27361 atccttgctt ttctatcaat ctctaattct gatcttcccc cccaatctga tattatggtg
27421 ccggtataga cagaaattcc gctatggtcc aattctgaac ggtaataaat accggggctt
27481 tgcaatattt gattgattac aattctgtat attccactta ctagagaggt tcccagggaa
27541 ttcattagag gaatatttcc aataaatacg gtttgttctt gcatatctct accggttttc
27601 caaattaatc ccgcggatac atataattca gaagagtatg tgagtgattc atacacagca
27661 tctctttctt ttatcaaggg ctctgccaat tgatatgttg ccacaaataa ttgaaattca
27721 atttcttggt ctgtatcttc aatttttgga aacttatgaa gttcttccat caagccttga
27781 tcaatgaacc tacaaaatcc gtcaaattgt atctgactaa accctggtat tgtatacatt
27841 ccctcatttc catcccggaa catcttaagt tttccgttta tcgaaaaaat ccaactattg
27901 gctcactctt cgttgaacca tatagattga tctagcaacg atggaatgta tattttgctc
27961 atttgaacaa catgaaattt tatccaaccc catatacata tatacatgta ctaaatacgt
28021 atgaacggag gaataaaaaa aaatgtgact caaattcgaa tttgcgacag atacaaatgg
28081 aaatgaattg ataaaacatt cctggaaaca aaattctgcc acttagactt atggagtctt
28141 gtatagaata tcaaaataga tccaatttct accttatgat attacgatca gattgggtac
28201 cataaatgga ttcggaattg aatctgttct ctatgagtga gataaagaca gaataatcag
28261 gaaccgtcta gagttgactt tgattttagc acttaattta tttcatcgat tccgttgttc
28321 aaaaaatgat tcgcagagag aaaagatatt tctacccatt tgttaattag tagaatacga

28381 ttgaagtgcg taagagaagt catatttatt aagtacatgc agatataact atctagctat
28441 ccgtatatcc catcttttat cgaagttccc ttgaggcaac ataggtcgtg ctatatccaa
28501 atttctattt tatattcaat atattcaatg aaaaatgcaa gcacgacgat ttcctaatag
28561 gaatatgtag ataagatacc tgactaggta tccgtgtaag aatttctgtt ctggggttta
28621 catatacaca taattgttgt tataattgaa attgaaaagg attaattatg gaaaagaatt
28681 gagactgatt agtcgtatat caatttgatc tccttatgtc attaaggaaa ccaaattgga
28741 gatcaaaacc caagaaccat tcatgaattc acagtcatta atgcttccaa tttgctctga
28801 attttggatt ctgtgactgg aaatccattt ttctcaatag aaaaaggggg gaaagctttt
28861 atttaggtgt tgtgtgtttt gaaatacaat caatctaaga gaacaacagg atccaatcaa
28921 aaaaaagaaa tggttcagca attccccaga atatttccat ctatatctat tttgtatcgt
28981 tttggcggca tggccgagtg gtaaggcggg ggactgcaaa tcctttctcc ccagttcaaa
29041 tccgggtgtc gcctgattaa caaaagactc ggaatttctt accctactaa actaatagaa
29101 ctcacaaaat tcttgcctgg cagaagcaga ggtaaggggc ggggactgtc gatacccaat
29161 tttaagaatc gggggttga ctttcaatta tttcttcaaa aatcggggtg tgacccaaac
29221 ctgtaccata ccaatatgaa ttaccaatat aaataaagaa atactcactt aattacggat
29281 tgctgatgcg ttcaggccat tgatttgatt tatcaatcga atcaaatcca tagtaagatt
29341 caattgtgag attcagaact acgaaagtca gggaccgtaa atccgtgtat ccaaaggaag
29401 gttcctaaga gactgtaaat ccttgctcct aggatccaag aaggggttct aggaaggact
29461 ataaatactt cctaattacc ttaccctact agtgtttact gactgagtct tgaagtagat
29521 tgggtaggct ggtggggaat ccaattagga gttgtggaaa gaactgaaga tactttgtat
29581 ccatacaaac tcatgagagt ctctgagtgc tcaggttttc aattaatatt gtatgggtga
29641 ttggctttta tagaataaaa gtggaaaaaa gtgctttcgt tggggtaacc ccgccaagaa
29701 tgtaataggg tgtcttccaa ttgtttcaca tttcacagaa gtagagacag taaggcttag
29761 atgggaaatt aggagtatgt gatagatagt tatatatctt gatggtatat ttttttttc
29821 tgctttttgt ttatgaaagg caacaatagg tcttactatt cctacatatt ccattagtca
29881 cattcccttg agacttccaa ggggcaactg tgtatcttgc ttgtacttag tgctttccga
29941 ttccaccaga aatcatatag ggacttgtta cgggtgtatt cattggattg gttcatcaaa
30001 aacgttaggt caaaatccca ttttgactct gcaccattga ttccactatt attagtgatc
30061 aagaatggaa taattccttc atattcatag agatagggga cacgattcac atggatatag
30121 taagtctcgc ttgggctgct ttaatggtag tctttacatt ttccctttca ctcgtagtat
30181 ggggaagaag tggactctag gggtactact aattgagttg agtaatcgaa tttatcaatt
30241 gtttaataga tcgttctgca aagcgctttt aaatcaaaat atctccacct cataaattct
30301 actggaatcc aatatgaata agaacctttc gatcaaacaa atatttcaac gacttgattc
30361 ccatattcgt atttcgaaac tcaaagggat acacatgatg ggaaattttt tccaaccgaa
30421 ttctttctaa atattctatt tcgacaaatc ggcccttact agaattatgc atattacaat
30481 gaggagcaac caacccctat tttttttttt tatttgtttc ccttttctct ttgctgttca
30541 aagagggaac cgttcttcta ttacgtacgt ggatatgtac tttctactga ggcgacatag
30601 acatagtcgt tgtccaaaga ggtactacgc ctaataagat cttactttcg ttgggtatgc
30661 gtacttacct ttttatactc ctaggaatct tatttatgct ttatcgactc gtctcatgtc
30721 atggttcaag catgaaaaat cggtggggtc tactacatcc ttttcaaaat ccgaagaagt
30781 tactatagaa ctttttggat catccgttaa cggatcaatc aattacttct tcgtaatgct
30841 aaaaaaaggc ttggttttct tttatataat atatgcccat actagtcttt ctccattgat
30901 tctttcaatg gatccccgga tccatattga aaataatcag aaacccagga attagaaaag
30961 ttgacgttcg attatttcag attgatcggg atcaatacaa attgacgtaa caaagaaata
31021 gaattggagt gctatttaca tgtacatata taaatgtggg tacatattgt ggattgatct
31081 atatcaagct catacctttc tacaataata gatagtgtgg tagaaagaac tataatgaac
31141 ctttctacca tactatctat tatactgctg actccaaccc gaccatttaa gacttggaat

31201 ttgaatccct ttctttcatt tcttcaatcg ttgataagaa ctaataagtc aagtttcagt
31261 caaattaatc actttgactg actgttttta cgtagattat aagtaaaaaa gcagtaggaa
31321 ctagaatgaa caacgcaata gcaataaatg ctagaatatt gacttccata atctcatcgt
31381 ttttttttgc ttcgcaataa ctcgggatct aatcccatag agatgataag tctttctcct
31441 gtaaattcaa taggatagat tgtatcctga tgatacttaa tcgtatcaat atcatgaata
31501 acaatatctg atctatcaaa tcgattcatc gtcgagaatt gaatagtata acataggaag
31561 atcttttatc cataccgaat ccaaaattgg attcctggtc caatcaagaa tcccattgaa
31621 tttctcattt ccactctttc ttttttataa cctgccgtct tccttataca atcatctgac
31681 cggcgttcca ttggtcacaa acccaaacgg tagggatgaa atggaaaaag gaatgagtta
31741 agttctaaac gaagtttttg tgaagatcta ctctttttgg aagacagaga agtgtgataa
31801 agattggtcc ggtagaaaag atctaacaga atattctatt ctgacaaatt catttattta
31861 ttgattttgt tttttcttcg atggggccat taaaatagga agaaaaaaaa aagggggggg
31921 ggtaggtttc atctgaaaag tactctgtcg ctgtcggggt aactagtaac tatactatat
31981 taattaaatt aattgcgtat cgtacaataa acgaatacaa tttgtgtatg tgctcccggg
32041 aaacgtatga gtactctatt acatggacca ggagcaatcg aaaaagacag gcccgtaggg
32101 tctctcttga aatctgaata gggcgatacc gccgatcaat ctacatatgt ctctccccat
32161 caatcggtac tagttgaagt aattgaaagt cccatatttg tacgatgaga aatgcgaaac
32221 gaaaaacacg aaagaaataa ggatccccg gggattaaat cctgctcctt gtccccctc
32281 ttcgcagaaa atggggagat gagttgatgg attcatcgga ttctaggtcg ggactgacgg
32341 ggctcgaacc cgcagcttcc gccttgacag ggcggtgctc tgaccgattg aactacaatc
32401 ccgggaaatg gggtgtacag catacataca tattcttata atttcattcg aaccctttct
32461 ttctattcta tattagattg aaaatcgaca tctttctgtt acaagaaaga cgagtgatat
32521 actgatatac acatggatat ggactatagt gggagtgaca cggattacta gtaatcctgt
32581 ggttatttta ttacccaatc aattgataat ccatttttca atgaaaaaaa aaaaggactc
32641 tttatttcta tctatcaggc atttcattta tagaggacaa actggttata tcatcctcat
32701 ggatcggcga attgttgggc cgagctggat ttgaaccagc gtagacatat cgccaacgaa
32761 tttacagtcc gtccccatta accgctcggg catcgaccca ggaagaatca attctaggct
32821 tattgataat ccatgatcaa cccccttcg tcttaccccc aggggaagtc gaatccccgc
32881 tgcctccttg aaagagagat gtcctgaacc actagacgat aggggcatac ccgcccgatc
32941 gccatcatac tatctatgct catagtatga gcagtttttt gaaattgtca atatacaata
33001 tatatgacta gatccgaaga atctttcttg cttacaagat tccatagaat ggaattttgg
33061 gattgttgat tcatgaacca tcctatatat aagagaggat aggatccttc agggagtgat
33121 ttgtccgaca gaaaaagggc aaaccccatt ccatttcttt cattttcact cgttgattcg
33181 ttcgtcgtta aggtgagata tgcctatctc acactaacac taaactaagc caggaaattc
33241 agaaacgata gaatttcttt ttttgaggat cgacgaataa tcgaaaagat tcttttttt
33301 tttttctaat aatttaattt agggtacgaa tcgaatccct tcatcacatg attcgatgaa
33361 ataccttgga tctatatcgg attggtacat gtatcaatca accaagcgaa tctcgtccgg
33421 atgaatcaat aaaagcaaag caattaggag cgtccctgaa acaattcatt gcattgatat
33481 ttctcaaata tcaataacta aaacttccta ggtaaatcaa atttattgtt cctgaatgag
33541 cccctatgta tacatgtaca ttatatacat atacattgta gtacatacat agatatatgt
33601 agtagactct atagtagcta gtgattaatt catttttga agaaaatggg cccttttaac
33661 tcagtggtag agtaacgcca tggtaaggcg taagtcatcg gttcaaatcc gataaagggc
33721 tttttctacg aagctccagt cttcgtcttc atttttcatt ggagaataga gatattgttg
33781 atatttgtaa taaaagtaac ccataatgag ttatcattct aatgagttat aggtataaag
33841 tgaaacagtt gtttattatg attatgataa gtaatcgtac ttagtaggag gactactatg
33901 taattcacta caagctatac ccctcctcat attattccta tttttggtcc tgggacatag
33961 atattctaga tacccaatcc aaattgtgaa tcgccaaacc aaagtattcc catttctcta

34021 ttgttccaat caaatccctc ggaaaaatta gaaatcaaga aaatcaaaaa gtaagtggac
34081 ctgagccatt gaatcatgac tatatcagct attctgatat tcaaattcga tagagatgaa
34141 attgtagaag cgaacttttt ctttccttgg accacgcaat aatttgtcga tatttcagat
34201 tgaatcttct tgttcctgga tgctccatag gaataaattg ctcttccttt cctccacaga
34261 gatacgttta ttccaagtca caagagcaat ctctttttca atacctttct ttgattccag
34321 aaaaaaagaa gtttctatct atataggatt tagatataga tatcaaatca tggcttcagg
34381 tacaaaatat ttccatattg atgcatcaga tatttttgtt cctccaatgc aacggaaaac
34441 gagtgcgata aaggagggat tttgatttcc agtctcccta tttaatttag gggcaggga
34501 caaaaaatag ggtccttttt tttctgccgg atatagggta ataaaaaaaa gtaaagaggg
34561 aaatattcga agtttatttt ttttggttcg acccgcgaaa agatatactc tggaatttta
34621 gattcattcg aaggaaatat aacaaagaag acaataacaa acaaaaagca atcaaaaaag
34681 gaaggagtaa gaaattatat atatatatag gatactgtag tagtttagta tacacataaa
34741 ttacgagaat ccataaagat atttattgat cttttctcaa taagatccaa gaacaagaat
34801 acgattagct tatggaatgg cgagctagat ctggggagca actgataacg agagaaagga
34861 tcgcttgttt cctcacagtt atttcaaaaa atggatctga ttgatgggtc ataagacaat
34921 tcagggttcg gatggttatt aagaataaga aggaataagg aataggaagg aataattgaa
34981 tcgaactcat ggatttacct aggttggttt ctggcccaat agaaaggaag gatttgtatc
35041 ttcgaaaccc attggatgga aggcgcagtg gacgaggaat cgttcataga tgaccgaacc
35101 atcgtatgcc ctgagaatga tatgaggtgt tcggaaatgg ttgaagtagt tgaataggag
35161 gatcgatatg actatagccc ttggcagatt taccaaagaa gaaaatgatt tatttgatat
35221 tatggatgac tggttaagga gggaccgttt cgtttttgta ggttggtctg gtctattact
35281 ctttccttgt gcttatttcg ctttaggcgg ttggttcaca ggtacaacct ttgtaacttc
35341 atggtatacc catggattgg ccagttccta tttggaaggc tgcaatttct taaccgctgc
35401 agtttctact cctgctaata gtttagcaca ttctttgttg ttactatggg gacctgaagc
35461 acaaggagat tttactcgtt ggtgtcaatt aggcggtctg tggacttttg ttgctctcca
35521 tggagctttc gggctaatag gtttcatgtt acgtcaattt gaacttgctc gatctgttca
35581 attgcgacct tataatgcaa tcgcattttc tgctccaatt gctgtttttg tttctgtatt
35641 cttgatttat ccactaggtc agtctggttg gttcttcgcg cctagttttg gtgtagcagc
35701 tatatttcga ttcatcctct tcttccaagg gtttcataat tggacgttga acccatttca
35761 tatgatgggg gttgctggag tattgggcgc tgccctgcta tgcgctattc atggtgctac
35821 tgtagaaaac actttattcg aggatggtga cggtgcaaat acattccgtg cttttaaccc
35881 aactcaagct gaagagactt attcgatggt cactgctaac cgcttttggt ctcaaatctt
35941 tggggttgct ttttccaata aacgttggtt acatttcttt atgttatttg taccagtaac
36001 cggtttatgg atgagtgcta ttggagtagt cggtctggcc ctgaacctac gcgcctatga
36061 cttcgtttcc caggaaatcc gtgcagcgga agatcctgaa tttgagactt tctacaccaa
36121 aaatattctc ttaaacgaag gtattcgtgc ttggatggcg gctcaggatc agcctcatga
36181 aaatcttata ttccctgagg aggttctacc ccgtggaaac gctctttaat ggaactttag
36241 ctttagccgg tcgtgaccaa gaaaccactg ggttcgcttg gtgggccggg aatgcacgac
36301 ttatcaattt gtccggtaaa ctactcgggg ctcacgtagc ccatgccgga ttaattgtat
36361 tctgggccgg agcaatgaac ctattcgaag tggctcattt cgtaccagag aaacctatgt
36421 atgaacaagg attgatttta cttcccccatc tagctactct aggttgggga gtaggtccgg
36481 gtggggaagt tatagacacc tttccatact ttgtatctgg agtacttcac ttaatttcct
36541 ctgcagtctt aggctttggc ggcatttatc atgcacttct aggacctgag actctcgaag
36601 aatcctttcc attcttcggt tatgtatgga aagatagaaa taaaatgact acaattctgg
36661 gtattcactt aatcttgtta ggtataggtg cttttcttct agtactcaag gctctttatt
36721 ttgggggcgt atatgatacc tgggctcccg gggggggaga tgtaagaaaa attagcaact
36781 tgacccttag cccgagtgtt atatttggtt atttactaaa atcgcctttt gggggagaag

36841 gatggattgt tagtgtggac gatttagaag atataattgg aggacacgta tggttaggtt
36901 ccatttgtat acttggtgga atttggcata tcttaaccaa accctttgca tgggctcgcc
36961 gagcatttgt atggtctgga gaggcctact tatcttatag tttaggtgct ttatctgtct
37021 ttggtttcat tgcttgttgt tttgtttggt tcaataatac cgcttatcct agcgagtttt
37081 acgggcccac cgggccggaa gcttctcaag ctcaagcatt tacttttcta gttagggacc
37141 aacgtcttgg ggctaacgtg ggatccgctc aaggacccac tggtttaggt aaatatctaa
37201 tgcgttcccc gaccggagag gtcatttttg gaggagaaac tatgcgtttt tgggatctcc
37261 gtgctccctg gttggaacct ctaagaggtc ccaatggttt ggacttgagt aggctgaaaa
37321 aagacataca accttggcaa gaacgacgtt cggcagaata tatgactcat gcccctttag
37381 gttctttaaa ttccgtgggt ggcgtagcta ccgagatcaa tgcagttaat tatgtctctc
37441 ctagaagttg gttagctacc tctcattttg ttctaggatt cttcctattc gtgggtcatt
37501 tgtggcatgc gggaagggcc cgtgcagctg cagcaggatt tgaaaaagga atcgatcgtg
37561 atttgaacc tgttctttcc atgacccctc ttaattgaga caggagatca aatgcatgaa
37621 gtaggaatcc atttgattcc attatacata ttaggttaag atcaggtcat atttaaaaag
37681 tattccttgt tttttccttt tcattctatc tatttttttc tggctcggct atcccaccta
37741 gccgagccat ttccctttat gacaccgggc caggccatac caataaagaa acaaatcgat
37801 tcaacgagca aaaggagaga gagggattcg aaccctcgat agttctttgt tcggaactat
37861 accggttttc aagaccagag ctatcaacca ctcagccatc tctccaagag acaatctcca
37921 ttttattcct ccgaatagaa catggccata tgggttgata ctctaactat ctgtagaaac
37981 atcccaagtg cgaatctata tttcgacata tctatctgtc tatagatgca tgatccaaca
38041 tgcccatttg ggaagtcaaa aaaaaattcc ctgattccat gtccgaataa aataaagtgg
38101 gactaagttc gaaaggatca ataaattcat ggtcaaatcc cgtcatgatg cattatttca
38161 attttgactc tgagagaggg atcaaatggt atagttcatt tgttggtagc ttggaggatt
38221 acaagcatga ctattgcttt ccaattggct gtttttgcat taattgctac ttcatcaatc
38281 ttactgatta gtgtacccgt tgtatttgct tcttctgatg gttggtcaag taacaaaaat
38341 gttgtatttt ctggtacatc gttatggatt ggattagtct ttctggtagc tattcttaat
38401 tctctcatct cttgaaccta ttcggtcttt cccggatcaa aaaactgacc cctccccaaa
38461 ttctttcgga ttgtaagaca cattaaaatg aaatatgagt ccaaaaataa aaataaaaaa
38521 attggaggga ggggtcaaaa atcacttctt gaataaaaaa aacgaagaat ctaataataa
38581 ttggaatctt cctaagtatc tgaccctgtc tgtacaaatg ggatccagac acatatatga
38641 tatatcatat atgtgtggac atatacgtgt gtatcaggaa cgaagaaagt gcggatatgg
38701 tcgaatggta aaatttctct ttgccaagga gaagatgcgg gttcgatccc cgctatccgc
38761 ccatggtaaa gtaaggtaat atgataaatg atttaggtat agttgaccac gataggggag
38821 tggttctatt cttcccatcc caaaaccaaa atagccattg gttactaggt aacggaatcg
38881 cacctaaaaa tgttttttttg aaaaaaaaaa aaaagagatg ttgcggagac aggatttgaa
38941 cccgtgacct caaggttatg agccttgcga gctaccaaac tgctctaccc cgcgctgaaa
39001 actaatggac gaacaagaat tggatgtgcc cccataccat attctatata aatagaatag
39061 cccatttata cagaatggta aagggggccc ctctatgatc atagctcata gagataaata
39121 gaaataggaa gaaggggtat ttttatcctt accaacttga tcttgtggcc cccggcaaca
39181 aacatgcctg aaccttttcg tgaagtatgt gtccggatag ccgaaagtct cgatagctag
39241 ctctaggtct tccggtcaaa aaacaacgtc gatgaagacg tgtaggtgta ctattacgtg
39301 gtggggattg caattttcca tgaatttccc atttgtcact caacgatgaa actttgccta
39361 tttctttttt tgaggatcga cgaatcaaat gatatttctg ttccaatttc tgcttctttc
39421 tctccctctg aatcaaactt ttccttgcca taaaggttca gttcctatta ttatcaatga
39481 tacgggtcgg atcctagatg tagaaataga agaaggtgga ttctcccttc tccatcgaat
39541 caaatgaaat tgtcgatgat acagcacatt aaaaaaaaaa attaaccaaa tttgcctgat
39601 gtagaggcaa tcaagaaagc tgcataagtg aatatataac ctacagaaaa gtgggctaat

39661 ccaaccaatc ttgcttgcac aatggaaaga gccactggtt tatccctcca tcgaatcaaa
39721 ttagccaaag gcgtgcgttc atgagcccat gctaaggttt caatcaattc ctgccaatat
39781 ccacgccagg aaattaagaa cataaatcca atagcccaaa caagatgccc aaataggaac
39841 atccatgccc agaccgataa actattcata ccaaaggggt tatagccatt gataagttgt
39901 gaagagttta accatagata atctcttaac catcccatca aataagtgga ggattcgtta
39961 aattgtgaaa cgttaccctg ccataatgtg atgtgcttcc aatgccaata aaaagtaacc
40021 catccgatag tatttaacat ccagaaaacc gccaaataaa atgcgtccca ggccgaaata
40081 tcacaagtac cgcctcgtcc cggaccgtcg caaggaaaac tataaccgaa atcttttta
40141 tctggcatta acttggaacc acgtgcatct aaagcacctt ttactaagat caatgtagtt
40201 gtatgcaaac ctagagcaat agcatgatga accaagaagt ctccaggacc tattgttaag
40261 aatagtgaat tactattctc attaacggca ttcaaccagc ccggtaacca tatacttcga
40321 ccagcattga atgccgggcc attcgttgaa gataaaagta catcgaaccc atatgaagtc
40381 ttaccgtgag cggattgtat ccattgggca aatatgggtt cgatcaagat ttgtttctcc
40441 ggagtaccaa aagcgagcat gacgtcgtta tgaacataga gtcctaaggt atggaaccct
40501 agaaagagac tggcccaact taaatgagat ttgatagctt ctttatggtc taacattctt
40561 gccaatacat tatcctcatt ctgttccgga ttgtaatctc gaatgaagaa tatagctcca
40621 tgagcaaagg cccctgtcat gatgaatcct gcgatgtatt ggtgatgagt atataacgca
40681 gcttgagtag taaagtcttg tgctatgaat gcataagcag gtaaagagta catgtgttga
40741 gctaccaagg aagtaataac ccctaaagag gctagagcaa ggcctaattg aaaatgaatc
40801 gaattattga ttgtgtcata aagaccctta tgcccacgtc ccaatcgacc ccccggagga
40861 atatgtgctt ctaaaagatc tttcatactg tgcccaatcc cgaagttagt tctatacata
40921 tgaccagcaa cgagaaaaat aaatgcaata gctaaatgat gatgagcaat atcggtcagc
40981 cataaacttt gcgtttgtgg atggaatccc ccgagaaggg ttagaatggc agttcccgcc
41041 ccttgggagg taccaaataa atgacgactg gaatcggggt tttgggcata aagattccac
41101 tgacctgtaa aaagtgggcc caacccttga ggatggggta atacatctaa gaaattattc
41161 catctgacgt actgccccct tgatcctgga atagcgacat gaactaaatg ccctgtccaa
41221 gccaaggaac tgactccgaa gagtcctgac aaatgatgat tgagacgaga ttcggcattt
41281 ttgaaccacg aaacgcttgg tttccatttg ggttgtagat gtaaccaacc cgcaattaaa
41341 gatatggcag aaagaaataa tagaaaaaga gctccagtat aaagatcttc attggtgcgt
41401 aagccaattg tgtaccacca ctgataaaca ccggagtaag cgatattcac tgggccgaga
41461 gcccctcctc gagtaaaggc ttctacagcc ggttgaccaa aatggggatc ccaaattgca
41521 tgagcaatag gtcttacatg taaagggtcc tgtacccatg actcaaaatt tccttgccaa
41581 gctacatgaa acagatttcc ggaagtccac agaaagatta ttgctaactg cccgaagtga
41641 gaagcaaaaa tgttctgata aagacgttcc tcagtaatat catcatgact ctcaaaatca
41701 tgtgcggtag caataccaaa ccaaatacga cgagtagtgg ggtcctgagc taagccttgg
41761 ctaaactttg gaaatcttaa tgccataatg cctttcaaat cctcctagcc attatcctac
41821 tgcaataatt cttgctaaga agaatgccca tgttgtggca attccaccca gaaggtaatg
41881 ggttactcct acagcgcgtc cttgtacaat gctcaaggct ctaggctgag tagcaggagc
41941 aacttttaat ttgttatgag cccaaacgat ggattcaatg agttcttgcc aataaccacg
42001 gccgctgaat agaaacatta aactgaaagc ccagacaaaa tgagcaccta ggaaaaaaag
42061 gccatatgcc gataatgaag aaccataaga ctgaattacc tgagatgcct gtgcccataa
42121 aaaatcccgg agccacccat taatagtaat ggaactctgt gcaaagtttc ctcccgtaat
42181 atgagttacc accccttgat cacttatact accccaaaca tctgactgca ttttccaact
42241 gaaatggaat attactaccg aaatagcatt gtacatccag aatagaccta agaagacatg
42301 atcccaggca gatacttgac atgtcccccc tcttccaggt ccatcacaag ggaaacgaaa
42361 accaagattt gctttatcag gtatcaaacg tgagctacga gcaaatagaa cacctttcag
42421 tagtatcaat acggtcacat ggatcgtaaa tgcatgaatg tgatgtacca aaaaatctgc

42481 ggttcctaat ggaataggta acaaagcgac tttgccgcct actgctacta aatcaccacc
42541 cccccaagtc aagctggtgc ttgttgttgc accaggagct gttgcaccag gtgctaaagc
42601 gtgggtgttt tgtacccatt gagcaaagat gggttgtaat tgtatagcgg tatctgaaaa
42661 catatctcgg ggacgcccta aagcgctcat ggtatcatta tgaatataca agccaaaact
42721 gtgaaagcct agaaatatac atacccagtt gagatgtgat atgattgcat ctcggtgtct
42781 aaggacacga tctaatagat cgttgtatcg agtagttgga tcatagtctc ttaccataaa
42841 aatggctgca tgtgcagcag caccaactat gagaaatcca ccgatccaca tgtgatgtgt
42901 gaacaacgaa agttgtgtac catagtcaat agctaggtat ggatagggg gcatggaata
42961 catatggtga gctacaacaa tggttgaaga gcctaacata gctagattaa gagataattg
43021 agcatgccat gacgttgtta ggatctcata gaggccttta tgaccctggc ccgtaaatgg
43081 gcctttatgc gcctctaaaa tatctttcag gccatgacca atgccccagt tggtcctata
43141 catgtgacca gctaccagga aaagaattgc aatagctaaa tgatggtgtg caatatctgt
43201 cagccataga ccccctgtta ctggatctaa tcctccacga aaactaagaa attccgcgta
43261 ttttgaccaa ttcaaggtga aaaatggggt tgatccctcg gcaaaactgg gataaagttg
43321 agccaaaaga tcccgattca agataaattc atgaggaagt ggtatctctt taggatccac
43381 tccagcgtct agaaattggt taatcggtaa agatacatgt acttggtgtc ccgcccaaga
43441 aagagaccca agccctagta accccgctaa gtggtgattc aacatggatt ccacatcttg
43501 gaaccaagcc aattttgggg cagctttgtg ataatggaac caaccggcaa aaagcattaa
43561 ggctgcaaag accaatgcac cgattgcggt acaatagagt tgtaattcat tagttattcc
43621 agatgctcgc caaagctgaa aaaaaccaga ggttatttgt attcctcgga aaccccccgcc
43681 cacatcacca ttcaatattt cttgacctac tattggccaa acaacctggg cgctgggtcc
43741 aatgtgagta ggatcactta gccatgcttc ataattagaa aaacgggcgc catggaaata
43801 cataccactc agccaaagaa aaatgatgga gagttgaccg aaatgagcac taaatacttt
43861 tcgagagatc tcctccaaat cactggtatg gctatcgaaa tcgtgagcat cagcatgaag
43921 gttccagatc caagtggtag tatcagggcc cttagctatt gttcttgaga aatggccggg
43981 tctggcccat tcctcgaaag aagtttttat gggatcccta tccaccaaaa tcttcacttc
44041 tggttccggc gaacgaataa tcattgagtc ctcctctttc cggacaacac atacaaagaa
44101 acccgccaac agtcaagtaa ttagtgaacg atgggtattt atgattagtt ccttatcttt
44161 cctatccccc atctatcttt ttttttttta gttatttact agagcaatta tgatatggaa
44221 gtcgatccgg ggcaagtgtt cggatctatt atgacataac catggggcgc tcaacggacc
44281 tttataatat tttataaccc cctccggcgt gacacaaaaa cggatttttt gatacaagct
44341 agttagtgta ttcatatctc aatgtatagt atctagatgg atctacttca tatcttacac
44401 ggaacatatt acttacaata caaatcaaag gatcattcat tagtcattaa taagagacat
44461 cttgatatct atatttagtc attcgaggtc tgtctttttt actggcttag ctttattagc
44521 atagcagaag ggaatatttt ctgtactgta tccgtatcgt ttatccctat gacagacgaa
44581 atagaacaat cttagactta gaagggatat aatgaaattc cctgattggc tcttcctaga
44641 ggaacgatct attttatttg attgatggat cccatattat aatgaattca aaaagagaat
44701 gttcttattc aaacctcctt gcgatcttca accaattatg tgcttcaata taattccccg
44761 gagtaagcgc tatagcttgt ttccaatatt cagcagcttg atcggaccaa gcctctgcaa
44821 tttcagaatc tccttgtcga atggcctgtt ctccccggtc ggaataggtg ggtcaattcc
44881 ttcccttaga accgtacttg agagtttcct acctcatacg gctcgacatt cttttggtgt
44941 tccatcttaa tctaccatat ctaactgaat gagatttctc ataaatctat cccatttttt
45001 ttttttcggg ttaaccagaa gaggttaatt acacgagttt caaactctaa ttttgatcaa
45061 taatcagttt tctcttttct cccaccttca gaagaaccaa gcataggtat tttcctctat
45121 cgttcgaatt ttctgaaagg taactatctc ggtttcatat agaaattcat atagaatctt
45181 tgaaaaagac tttcctccat aagaaagaaa ggacttacta tctttgggat ctgatgctac
45241 accgctgctc aataccttag tagatcgact ctattacata agttgattcc taacttttat

45301 ctcatatcat gacattaagt aagcagtcct tattgtatcg gtccccgaac ctcactaatt
45361 gatctttacg gtgcttcctc tatcaattag atcctttatc catagaataa agtatatagg
45421 ccatacctat ttcttcatat ttcggctctt atgaagtctc tttctttgct acagctgata
45481 aaaatcgttg ctttggacga tgcatatgta gaaagcctat tttgtttcta gtattgacta
45541 gcggatttgg tctttccttc cttctttcta tagtgtagat agtcgcacgt aatgacagat
45601 cacggccata ttattaaaag cttgtggtaa gaatggattt cgttctattg cccggaaata
45661 atattccaaa gccttcgtgt gttctccgtt acttgtgtgg ataaggccta tgttatagag
45721 tatataactt cgatcatagg gatcaatttc tagtcgcgta gcttcataat aattttgtaa
45781 agcttccgca taatttcctt cggattgagc tgacatccgt tacggtcgtt cattctattc
45841 aaagaatctc cgttccagaa ccgtacgtga gattttcatc tcatacggct cctcccttct
45901 gtgcatagta ataaggggaa taatccatgg aatcaaaaaa gattgaaata ttctcattat
45961 gaactgacag gggctggtgt ttttacaaga aatctctagc cagccttcct gcaagaggtc
46021 tgtcttttct taacaccaag cgcgtttgtg ctagatagaa atggtaactc caacaatttc
46081 tttgtcctca acgcccctg tttccaggaa ttagtcactt caacgacctt tgatggttat
46141 acgggtatcc aaagtacgaa cgagatggat gtttgttgtc ccaaccattc ttgttagttc
46201 cgatcccgat aaggaaaagg gttaatttat aacaaagttt tcgtgttgtt gatttctaga
46261 tgtagtgctt cttcccctat gcggcctatt ggtactagtg gagtaggatt gacccgcaat
46321 acagaaccta taggtgtaac ctttcgctca atactagaat cgacagttga agcatctaag
46381 gctgcatcaa tcggggatac acgacagaag gaattgttct atctccaaac ttcaccttca
46441 tcaagcgtag gtttatttca agaatctttt ttctttgtat cccgaatcat gtctctttct
46501 cgtaagactg agggcggtaa ataaataaat tcaaaaaaaa aagcaaatcg caccatctct
46561 gtaataggta aatgcctctt tttctcctga ggttgtcgga attattcgta ataagatatt
46621 ggctacaatt gaagaggtct tatcaataaa atttccattt atccgagatc taggcatagt
46681 taacaatcca ttctagaatt cttctcatta cccctcaggg gaaaatgatt ccacaaacaa
46741 aggaattgta cagtacgaaa tcacataaaa acagactcat tctaaaaaaa aaaatgtgga
46801 ccttccactc aaattatccc tttttgagag gtatagatag gaaatatttg aatcggattg
46861 gatttcattg aaattgagta gtataccaat gaatggaact cttttatttt atcgaagtta
46921 agaaatccag gaatttttac taccgattct tataatttaa ttcgataaat ttggatttga
46981 ttatgatcca aagaggaaaa agaatcaaat aatcattcca tgatgaaaat agaataacca
47041 tccattttgt gtgcatagtg tggatacacc atccaatcga aagataaaaa tctatagaac
47101 gattcatgaa tttgtaatag atctatggag tagctcatga gaggagttgt tgttgagaaa
47161 tctgaaactg gaagggggga attttgtaat tcctatggaa tcgtagttta aatataaaca
47221 tagtctaaaa tagggtcagt tgactcgttc caattcattg gcttaatccg aaatattaga
47281 ataagatagg atcgtcgtat tgacaaacaa gacatttttg tttttaacaa gaaaaaagtg
47341 tgtttttttt atccctcgag cctcgaagga aaatcgttct ttagcgaaaa gttttctatt
47401 tctaatagat tggtcgtacc tgtattgcaa taatatgaat gactcgctat ttactcggtt
47461 tctggggcat aataataaga ttatgtagga gagatggccg agtggttcaa ggcgtagcat
47521 tggaactgct atgtagactt ttgtttaccg agggttcgaa tccctctctt tccgtacctt
47581 catctaattc accaaccgac cacaatgtat caaatcaaat aacaattgat accattattc
47641 caacagtaag acccttattt gatagagatt ctctattcct aattactgca gtacggaaaa
47701 taccggaaag agtggaaagg aatgaaaatc tcactgctga tccatttgtg atacgtgaat
47761 gggagaaaaa tccggatcaa acccccttctt cggtgaaaaa aaaaaagagg ggggggggca
47821 aaatggtccg aagctttgtt attttagtta ggttcaagtc tgacgggaat aatattctac
47881 gactagaaac tcattgattt tcaaaccgat ccatttaata tctattattt gatttactaa
47941 tcctttatat tgggatgagt caaaagtcaa atgttttgcc aaatcctcgc ggggcgatga
48001 atcaagataa ttttgaatca gagctctgga tctttgttca tcccttgcag taataatatc
48061 tcggggtttg cagcgataac ttgggatatc tactacacga ccattaacta aaatatgtct

48121 atggttaact aattgcctgg ctccaggaat ggtcgaagcc atacctaatc gaaaaaggat
48181 gttatccaaa cgcatctcaa gtagttgtag taaaacctga cctgttgacc ctttggcttt
48241 tccggcaata cgaacatatc taagcaattg tcgctctgtc agaccataat gaaaacgcaa
48301 tttttgtttt tcttctagac gaatacgata ttgagatctt ttcccgaaac gtgattggtt
48361 tctaagatca cttccggatc taggtctttt actagttagt cccggtaaag cccccagaca
48421 gcgtattttt ttgaaacgag gccctcggta acgagacata aagactcctt gttaaaattg
48481 tattttacag aataaactta aattaagact gaactaaacg ataaacgaaa ctaaatctat
48541 tgaagtacta caaaagaaga ctacaaaaga agaatgagat gaattgtatc aatatccgga
48601 ttattttgta tatataggaa gtgaaggacc cctttcttga tttgttctgt agtgtagaga
48661 tttaactgct ccaatcaaat aagtttttta ttcatagttg gaagttgcta cgacataata
48721 gatcggtgac ccgacatttt taaaagaaaa aaagagagga gtcttttcaa tattccttga
48781 gatcaaggaa tattgaaaag ccggctatcg gaatcgaacc gatgaccatc gcattacaaa
48841 tgcgatgctc taacctctga gctaagcggg ctcacataac agaaataagt gcaatagaac
48901 taactaacta tatctatata gaatgttttt tttaattctt aatttatata ttatacttaa
48961 tttatagcag ttagttatag cagattagat taatcatatt agagcagatc ggtactaagg
49021 aaaggataag ataaggatgc aatccagatc ataatgagac atttcgccgg tttcattcag
49081 aaaggggggа ggtagaacga aaaaaaaaaa tgaatatcga ccgttccagt attaaaaatc
49141 gagcgggaaa aatgagaggg ggggagggta tgtatatgtg ggatatctct atccatattg
49201 aattgcagat acatcaatga tagaatcatt tctgatggga ccaaatacgg gtcttccgat
49261 agagaatatg gacaagaaat caaaataaaa taaataaaat aataaaatag gagtagactt
49321 tttttcgata ttaggaatca gtatctaatg aattcaacgg ttccgacata aataaatgaa
49381 agagggggat gggatcacaa tgagatctcg gtctcataag gggatatggc gaaattggta
49441 gacgctacgg acttggttgg attgagcctt ggtatggaaa cctactaagt gataacttcc
49501 aaattcagag aaaccctgga attaaaaatg ggcaatcctg agccaaatcc tgttttcaga
49561 aaacaagggt tcagaaagcg agaaccaaaa aaaggatagg tgcagagact caaaggaagc
49621 tgttctaacg aatggagttg attaacattg gtataggaat ccttctatcg aaattccaga
49681 aaggatgacc ctatcctata tacgtactga aatatcaaac aattaatcac gatccgattc
49741 tgtatttttt ttatatgaaa aatggaagaa ttcttgtgaa tcgattccaa attgaaggaa
49801 gaatcgaata ttcagtgatc aaatcattca ctcctcggat agatcttttg aagaactgat
49861 taatcggacg agaataaaga tagagtccat tctacatgtc aataccgaca acaatgaaat
49921 ttatagtaag gggaaaatcc gtcgacttta gaaatcgtga gggttcaagt ccctctatcc
49981 ccaataaaaa gaaaagagcc cgttttacta cctaacctct ttatttcgtc atcggttcca
50041 aattagttat gtttcttatt cactctactc tttcacaaac ggatccggac agaaaccttt
50101 ctctcttatc acaagtctat agatacgata tacttacaaa tgaacatata taggcaagga
50161 atttccatta ttaaataatt cacagtccat atcattactc ttacactgac aaagtcttct
50221 ttttgaagat ccaagaaact ccaaggccta ggtaagattt tgtaagactt tttgggtttc
50281 tttaattgac atagacccca gtcctctaat agggcgatgc atccggaatg gtcgggatag
50341 ctcagctggt agagcagagg actgaaaatc ctcgtgtcac cagttcaaat ctggttcctg
50401 acacgcggtt aatgtatcga atggatactc atccaaatga atgggtaaag gaaagaagta
50461 gattttgttc cttttttta tactgtaccc cctctcgctc aaaaagaatg ttaatacttc
50521 atacatatcc aaagttaggt ggctgaaacc aaaaagtcta gcctagggga gttgaaggat
50581 aggaatagac aggattcatt tcagatacag tacaaagaaa atacgatccc ttttcatttc
50641 tgaatttcat attttcttgc gtattctatt tcctcactcc ctcttacgcg acttccagga
50701 gcccatccaa gtgatatgcg cggtacaaag ttcatggtac agaactcttt tgattcatac
50761 tattggcttt actcatccga aatagatata tttaaaattg gggaatatca acgaagccta
50821 tttattagct catccataat acgaattaga gcccagttac tctgtttcat ctagaacgta
50881 aaaagattcc ttgaatatct ggagtcgtag aagtgaagat tagtttctta tcattcaatg

50941 agcatcttgt atttcataga aattgggggc aatataatcc ttacgtaggg gccatcccac
51001 ccaactttcg ggcatcaaga tacgtttcag gcgtggatga ttttcataag agatccccaa
51061 catatcataa gattcccgtt cttgaaaatc agcacttttc caaatccaga aaacagacgg
51121 gattctagga ttcctccttg gaacaaatac ttttatgcac acctcttcgg gttgatccac
51181 cccatactgt attctcgtaa gatgatacac actagctaaa aatccgccag gtgctacatc
51241 ataggcacac tggaaacgta gataattgta accatataca tatgaaataa cagcaatgga
51301 gtaccaatcc tcgggcttta tttgtaaagt ctctcttcct tggtaatcga agcccaacga
51361 tctatgaact agctcatgct tgactagcca agcagatgaa cgaccctgca tcttcttgat
51421 ctctcccaca tttgtatgaa tattttacat ttacgatgaa ttttatgaaa attgactcgc
51481 cgtttgttat tccgcacaaa aacaccctgc ctaattcatt aatttggggg aagatactga
51541 acttttgtat ttgaaaaatg tttcagaagg tatctctgaa gtagatggag attggtagag
51601 taatccttga tcgtaatttc cagtatgagt actgtgtcca acatgaaact tgtgattggt
51661 agtaaaatat cgattttcct gttgagaccc aattctatct tcatagattt ctcgagagac
51721 tttcttacga agtttcgtta tagcatctat aactgcctct ggtttagggg ggcagcccgg
51781 caaatagaca tccacaggaa ttagcttatc gactccccga acagtactat aagaatcggt
51841 actgaacatc cctcctgtaa tagtacaggc tcccatagca atgacatatt ttggttcggg
51901 catttgttca tataatctca ctaacgaagg agccattttc attgttactg tgccggctgt
51961 taaaattagg tccgcttgcc taggacttga tcttggtacc agtccataac gatcaaaatc
52021 gaatcgcgag cctattaatg aagcaaactc aatgaagcaa caactggtac cataaagaag
52081 cggccataaa ctagagagtc ttgaccaatt cgaaagatca ttcgatgtag ttgaaataac
52141 tgaattttgg gcggttcggt caagtaacgg aaactcaata gaattcataa ctgtttcaat
52201 gtaatctttt ccttcttttt gattttgatt gtctgaatat tcaggagcta agaccattcc
52261 aatgctcctt ttcgccatgc ataaactgaa ccaacaattg ggataagcac gaaaattaaa
52321 gcttctataa acacagatac acccaataca tcgaaactca ttgcccatgg ataaagaaag
52381 actgtttcaa catcaaaaac aacaaaaact agagcaaaca tgtaatagcg gattcggaat
52441 tgtaaccaag catcccccat cggttctatg cccgattcat aactagagag cttctctggt
52501 ccttcactaa tcggggccaa aactccggaa attagaaatg ccaaaatagg aataacactt
52561 gatattatta gaaatgccca gaaaatatca tattcgtgaa gcagaaacat agaagcactc
52621 ctattaatgt ggaatatacc gaattagttg attcaaattg gaattctcaa ttcatccata
52681 actgcattag tcgaaacaac aattttgatc aaaccacata gtttcgtttg tttacttgtt
52741 gtgggtcatg tatcgtctca agattcatcc aacggaatcc cacttacact tacttcgatt
52801 ctatttagat atggtgtaga catataatgc tattatacaa atcaaactct ctcctacctt
52861 gcctcgggtt ttctatcaaa caaaaaaagg aattaaggaa ttttttttaa agaatatttt
52921 aaataatatg aattgaaatt gaaataatat tcaaacaata ttattcaaat aagattaaat
52981 gaaatattaa acataaataa tttcaatatt ctattaatat aatagagcca aagaggaggt
53041 ctggcccatt ttttctctct ctctcttttt tttttttttt cttagtgatt tagaatatag
53101 tcagtagtca gatgtaatag aatttctagg aatttccatc tcgggattta tggtatattc
53161 tacgtggctg tgttggtgta ttcttttcat taagatccgg atagaggatt attgtttcta
53221 ttgatttgat acggatacga aaacggaatg catcgaccga ttcgattctc tctccctgtc
53281 ccagatttat acttaattga tttgattcaa tccattgaat tgtgaggaac ccttacatat
53341 aaaaactcat gggttcctat acccaaatta ggaaactttg gacctgtact accccggccc
53401 cggctttacc cccgagttag aagtcttgaa agaatcattt cagacccatt tctaggacta
53461 aagatcgtga tttggaatga ctcgaaatac ttatttattt aatgtaataa taacacctag
53521 caggaccaac caacgagtta ggtttcgtga caacaaaaaa cgtttctttt gaagcaaacc
53581 tacaaaatgg ggcatagttt aatggtagag tcggctgaat cgtaaatgat ttacagaaga
53641 tacttcgaat ggaatcatgc gttgtcgaac gattcgatag acaaaatctc cccatcccaa
53701 aaccaactca tagaacatag aaatagaaga gggtgcgttg atacatttag gaccaattca

53761 ttgtctctaa aacaatgaat tgggattgtt gttctgccaa aaggcgatac gtcgggggat
53821 tcctaactca tccaagttca aatgggccct aatcttttta gataaagtct gcattggtag
53881 aattggaatg atgaacaaat tggattgggt agattggaat aaaaaaaaga agttattaag
53941 tattgtacag aaaaatgact acttgctttg ctaagccggt tatacgaaga aaagcctatt
54001 gtacaatgaa acttaccaaa gagcttcgtt tttgaaactc tggcttttct acaaatacaa
54061 gaacaagaat aggttctaga taatgtgact tactattaga ttgaatttgg atttgatttg
54121 gttgggtcag gttggagttt ttcttgagcc aggctcatgt tatgattttg acttcataaa
54181 ttggcttggg tataccaaag caaaggtgta tcactaaatc ttggatcatg gacaaataaa
54241 agaggaaaaa ggccgtatgt cattcacaga cgaagattaa tgaagaagaa tgggtttgtt
54301 tatccgagat ttgaaaatac cgatccgatt ggatccattg gaataaatta ttgttttcaa
54361 gccccgaggg atctccgtga tcctgtggga atgattccat ttctatggaa caatcaaccg
54421 gccggtcaca cgcactaatt aggaaatgaa tacaaaaatg tatagggcta tacggactcg
54481 aaccgtagac cttctcggta aaacagatca aacttattat tatcgaaatg attcgaactg
54541 tttcaaagac ccaacatgcg ttttttttt gcattgggct ctttcattaa ctgataaaaa
54601 gatcggctag tccaccatat tttttcttga caggaagata acgagatggc tccatgcgct
54661 cggattcatt atttgaattc tgatccggga gcaataccaa agtgtttcaa agaagggtta
54721 ccctgacgta ggtctgcctc cggcctagat caacctaagt taaatggagt ctctatcaat
54781 ccgccccaag agtcaaatat gatacttaat acaccttaaa gttcatagga cgaaaagagg
54841 ttattttgag gtccttatcc tcattatgcc tagcattgaa gggactgggt attcacctta
54901 tcaatgatca aaccaatgat gggttctatt tggtacctga attggcacct gaatcggacc
54961 gaacaaaata tttgtcaggc tattgttctc ttgttccctc gaatccatgg agtaagacat
55021 cgatttctca ataagatcaa ttctgttgat tgcatgatgg actcctctga aaaagcattg
55081 gcgcgcgtgt aaacgaggtg ctctacctaa ctgagctata gcccttgtca tagacatatt
55141 aacatctaga taatttcttg tcaagatgga tattccataa tcccacatga taactctccg
55201 atccgtttcc tgccaaggat tggtattgct gagaagtaat attccgtcta taatccccga
55261 tgtgatgggt cccatttttt ctttctcttt gtgatgataa atgacctact taacccagtg
55321 gttagagtat tgctttcata cggcgggagt cattggttca aatccaatag taggtagaac
55381 ttattagata ccggagtcga tggtatctaa taagtttttc tacccacctt cttctctttt
55441 ttttttatgg attttgtacc ctttccctat tatacccccca ctactcatat ttgtatttgt
55501 ttttttttg ttcgttacat cagattacaa ttgattgtat ccaattggcg gaatccaaat
55561 atggtgtata aacagaactt cttttgatta ttctgataca ttgactagta cgaaataaca
55621 ttgatagcct ctactcgtgt cctagctcgt ctaagagcta gattcgcctc aattgcttgt
55681 ctcttgcctt cagctctact caagttagct tcagctattt caagagttcg ctgagcttct
55741 tgtggatcaa tgtcactacc cttctctgca tcatttacta aaatggtgat ctcattattg
55801 cctattctag cgaaaccgcc catcacagcc atcgttaacc attggtcgtt gaggcgtatt
55861 ctcaaaatac ctatatctac ggctgtggca ataggggcgt gatttggtaa tacgccaatt
55921 tggccactat tagtagataa aatgatttct ttcacttccg aatcccaaat aattcgattc
55981 ggagtcagta cacaaagatt taaggtcatt tcttcaattt gctctccact tctaagttca
56041 tagccttcgc agtagcttca tcaatgttac ctaccaaata aaaggcctgc tcgggaagac
56101 catctaattc tccggaaagg atcagttgaa accccctaat tgtttctgta agaccaacat
56161 atttccctgg agaaccagta aatacttctg ctacgaagaa gggttgtgat aagaaacgtt
56221 caatttttcg tgctcttgct acggttaaac gatcctcttc agataattcg tccaacccaa
56281 ggatagctat aatgtcctga agttctttgt aacgttgtga agtttgctta actctttgcg
56341 cagtttcata atgttcctca ccaacgatcc taggttgtag catagttgac gttgaatcta
56401 acggatctac tgctggatag ataccttttgg cagctaatcc tcttgatagt acggtagtag
56461 catctaagtg cgcaaatgtc gtagcaggag cagggtcggt caaatcgtcc gcaggtacat
56521 aaactgcttg aatggaagtt atagacccct ctttggtaga agtaattctt tcttgcaaag

56581 aacccatttc tgtactaagg gtaggttgat aacccacagc ggaaggcatt ctacctaata
56641 aggcggatac ttctgatcct gcttggacga aacggaaaat attgtcgata aatagaagta
56701 cgtcttgttc attaacatcc cgaaaatatt ccgccatggt tagggcagtc aaaccaactc
56761 tcatacgagc tcccggcggt tcattcatct gtccatggac tagagctact ttggattctg
56821 caatattttg ttcattaatc actccggatt ctttcatttc catgtaaaga tcatttcctt
56881 cacgagtacg ttcgcctact ccgccaaata cagatacacc tccatgagct ttggcaatgt
56941 tgttgatcaa ttccatgatg agtactgttt tacccacccc agctcccccg aatagtccga
57001 tttttcctcc acgacgataa ggggctaaaa gatctaccac tttaatccct gtttcaaaga
57061 ttgataattt ggtatctaac tggataaaag caggcgcaga tctatgaata ggagatgttg
57121 tgcgagtatc tacaggacct aaattatcaa caggctctcc aagaacgttg aaaattcgtc
57181 ctagagtagc tccacccact ggaacgctta gaggagctcc cgtgtcaatc acctccattc
57241 ctctcatcag accatctgta gcactcatag ctacagctct aactcgatta tttcctaata
57301 attgctggac ctcacaagtc acattaattt gctgaccgac agtatctcga cccttaacta
57361 ccaaagcgtt gtaaatatta ggcatcttgc cggggggaaa agctacatcc agtaccggac
57421 caatgatttg agcaatacgc cccaggtttt tttcttcaag tgtggaaacc ccaggcccag
57481 aattagtagg attgattctc ataataatga aagtgaaata tgtcaaaatt ttttgcgaat
57541 attaccgaat cgaaaataaa tgtccgatag caagttgatc ggttaattca ataaataaga
57601 aatgggagtt agcgcttgat ttcgttggta ccattcaact gaatccaact caatcgttta
57661 ctcattcact aaatgaattt tcaagttcaa ccaacccttt ttcaaaatat ctatcaaatc
57721 aagtagatga ataagaatca tggggaagtc tttcattttt ctatcattat agacaatccc
57781 atccatatta tctatggaac tcgaacctga actttattta tgattcagaa tttctatctt
57841 attggccgtt gttccttatt tcagcatatt agtttccgcc tattcttgtt tttatttttt
57901 ataccctttc atggatgaat tctgcctatt ttcacatcta ggatttacat atacaacata
57961 tatcactgtc aagggtgaat ttcttattat ttagattcaa aaaaaaagaa ggagatccaa
58021 acttgcaaaa caaggattgg gttgcgccat acatatgaaa gggtatacaa taatgatgta
58081 tttggatgta tttggcaaat caaataccat gataacgaac cattctaatt agttgataat
58141 attagttgag aattttgtga aagattcctg tgaaaggttt cattcattac taatccatgt
58201 cgagtagacc ttgttgttgt gagaattctt aattcatgag ttgtagggag ggacttatgt
58261 caccaaaaac agagactaaa gcaagtgttg gattcaaagc tggtgttaaa gattacaaat
58321 tgacttatta tactcctgac tatgaaacca aagatactga tattttggca gcatttcgag
58381 taactcctca acccggagtt ccacctgagg aagcaggggc tgcggtagct gccgaatctt
58441 ctactggtac atggacaact gtgtggaccg atggacttac cagccttgat cgttacaaag
58501 gacgatgcta ccacatcgag cccgttcctg gggaggaaac tcaatttatt gcctatgtag
58561 cttacccttt agaccttttt gaagaaggtt ctgttacgaa catgtttact tctattgtgg
58621 gtaatgtatt tgggttcaaa gctctacgag ctctacgtct ggaggatctg cgaattcctc
58681 ctgcttattc caaaactttc caaggcccgc cccatggcat ccaagttgag agagataaat
58741 tgaacaagta tggtcgtccc ctattgggat gtactattaa accaaaattg gggttatccg
58801 ccaagaacta cggtagagcg gtttatgaat gtctccgtgg tggacttgat tttaccaagg
58861 atgatgagaa cgtgaactcc caaccattta tgcgttggag agaccgtttc gtattttgtg
58921 ccgaagcaat ttataaagcg caggccgaaa caggtgaaat caaaggacat tacttgaatg
58981 ctactgcagg tacatgcgaa gaaatgatca aaagggccgt atttgccaga gaattgggag
59041 ttcctatcgt aatgcatgac tatttaacgg ggggattcac tgcaaatact accttggctc
59101 attattgccg ggacaacggc ctacttcttc acatccatcg cgcaatgcat gcagttattg
59161 atagacagaa gaatcatggt atgcactttc gcgtactggc taaagcgtta cgtatgtctg
59221 gtggagatca tgttcacgct ggtaccgtag taggtaaact agaaggggaa cgggacatca
59281 ctttgggttt tgttgattta ctacgtgatg attttattga aaaagaccga agtcgcggta
59341 tttatttcac tcaagattgg gtctctatgc caggtgttct gcccgtggct tcagggggta

59401 ttcacgtttg gcatatgcct gccctgaccg agatctttgg ggatgattcc gtactacagt
59461 tcggtggagg aactttagga cacccttggg gaaacgcacc tggtgcagta gctaatcggg
59521 tggctgtaga agcgtgtgta caagctcgta atgagggacg tgatcttgct cgtgaaggta
59581 atgaaattat ccgtgaagct gccaaatgga gccctgagct agctgccgct tgtgaggtat
59641 ggaaggagat caaattcgaa ttcgccgcaa tggatacctt gtaatccagt aattcccgtt
59701 cgttccccta attgtaatta aactcggccc aatcttttac taaaaggatt gagccgaatt
59761 aaagaatgag gatcctatgt atatggatag atatagatct tgtatctatc aatatgtgcc
59821 tactttacct agatatacaa gatctaaata caagataaga tctaagacta aacaactcaa
59881 tgcttctatt gttggatcca taattaatcc tatggatcct taggattggt ggatcctttt
59941 ctatcccgtt gtttcggacc atagatcgag ccaagggtca caacttcttc tactcatcct
60001 gtatattgtc cttttcattc cgtgttgcat tagaaactta ttattatacg agattatacg
60061 aaaatgaatc cttcctagga gggaacaaat atttctcttt tcgatgagag tttgtacaca
60121 acatgggaga aacctatctt ctatttataa taattgaaga aaaggttcca tcatatcata
60181 tatagtgaat tgatactccc gattcccaca aaatcatttc tttcgttcaa tagttactcg
60241 ttattagtta ataatcctag tgattggatc tatatgcgta ttccgatagg aaatgaaata
60301 gtaaaatgat ttttcgtcga atgactattc atttattgta ttttcaaata gggggcagga
60361 aggatctatg ggaaaatggt ggttcaattc aatgttgtct aacgaggagt tagaacacag
60421 gtgtgggcta ggtaaatcaa tggacagtct tggtcgtcct gttggaaata ccagtggaag
60481 tgaagatccc attctaaatg atacgaataa aaacaatcat aatcatggtt ggcgcgaaag
60541 taatagttgc agtaatgttg atcatttttt cggtgtcaga gacatttgga gtttcatctc
60601 tgatgacact tttttagtta gggatagtaa tggtaacagt tattccgtat attttgatat
60661 tgaaaatcgg gtttttgaga ttgacaatga tagttctttt ctgagtgaac tagaaactgc
60721 tttttctagt tatctgaata gcgggtctaa gagtgacaat cgctactatg atcattatat
60781 gtatgatact acgtatagtt ggaataatca cattaatagt tgcattgata gttatcttcg
60841 ttctgaaatc agtattaata agtacatttc gagtggtagc gacaatccca tttacagtta
60901 tatttatagt tacatttgta gtggtgaaag tgtaagtgat agtgacaggg ggagttctag
60961 tataagaact ggcggtaatg gcagtgattt caatataaga ggaagatcta atgatttcga
61021 tggaaataaa aaatacagac atttatgggt tcaatgcgaa aattgttatg gattaaatta
61081 taagaaattt tttaggtcaa aaatgaatat ttgtgaacaa tgtggatatc atttgaaaat
61141 gggtagttca gatagaatcg aactttcggt tgattcgggc acttgggatc ctatggacga
61201 agacatggtc tctattgacc ccattgaatt tcactcggaa gaggaacctt atagagatcg
61261 tatcaattcg tatcaaagaa agacaggttt aactgaggct gttcaaacag gcataggtca
61321 actaaatggg attcccatag ccattggggt tatggatttt cagttcatgg ggggtagtat
61381 gggatccgta gtaggcgaga aaatcacccg gttgatcgaa tatgctgcta atagatctct
61441 acctgttatt atggtgtgtg cttctggagg agcacgcatg caagaaggaa gtttgagttt
61501 gatgcaaatg gctaaaatat cttccgcttt atatgattat caattcaata aaaagttatt
61561 ctatgtatca atccttacat ctcctacaac cggcggagta acggccagtt ttggtatgtt
61621 gggagatatt attattgctg aacccaatgc ctacattgca tttgcgggga aaagagtaat
61681 tgaacaaaca ttgaataaga cagtacctga cggttcacaa gcagctgagt atttattcca
61741 taagggctta tttgatccaa tcgtaccacg taatccttta aaaggtgttc tgagtgaatt
61801 atttcagcta cacggtttct ttcccttgaa tcaaaattca agtagagcgc taggctcagt
61861 tatttgtagc gaactttagt tcatcggaat caaagtcaaa ataagaagag tggagttttc
61921 tttggtaaca taacttctat aggaagtttc ggataattac tttttttgat gcagattttt
61981 tatcctaccc ctattcatga ttagtaatca ggaacccccct atcaggagga aaagagtgaa
62041 ttcttccttc cgcggaatgg aattgggaaa aaaaatcaaa agaatttcat gttcccttct
62101 ttcatattaa tatatatcgt attaatatat atagaataat tcaaatctat aagggaagtg
62161 ttgcatattt ttatatctcc cgaggaccta cacttttgac tatgaattcc tgttggatcg

62221 gattctaaca aatccttagg aaactcgtaa gaaactcttt attagaagat aagggcggta
62281 gaacaagaat aataaagcgg attatcatcc atctatttct tgtagaaagg tgaatagata
62341 cacttattta gctctacatt ccttgcactt attatatact taataatact tataatagat
62401 atacttatca taagataaga tatctttata acaggtacaa atattaaatc gaggcaccca
62461 ttctatgaca gatttcaatt taccctctat ttttgtgcct ttagtgggct tagtgtttcc
62521 agcaattgca atggcttctt tatctcttca tgttcaaaaa aacaagattg tttagacctg
62581 ataggacaaa atttcatcaa tttatttcaa cacttggact tggatcataa tagggatatc
62641 catttagtgg aatatgatac gacatgtggc cccctccggg cacaaataaa aaagtgatta
62701 tacatgcgga tacattatat atggataaat gcatgtatat gggggatag ctgttttaaa
62761 atggatcaga gcggatatct gaaatagaaa gttaacgtat ctatattatg tagatataca
62821 tagtggtggt attatacgaa ggggatgtta ttattttata tctaaccaat tcgatgaatt
62881 actcctaata gttcgcgtca taatagtgct agttgatgag agttacttcg ggagcaaaat
62941 aaaaaaagta aaatcaaatt catttggctt attctcttct ctcaattcca ataggatgca
63001 actgaatcta gtatgaacta tcgatcagaa cgtatatgga tagaacttat aacggggtct
63061 cgaaaaacca gtaatttctg ctgggcctgt atcctttttt taggttcact aggattctta
63121 ttggttggaa cttccagtta tcttggtagg aatctgatat ctttattccc gtctcagcaa
63181 atcatttttt ttccccaagg aatcgtgatg tctttctatg ggatcgcagg tctgttcatt
63241 agttcctatt tgtggtgcac aatttcgtgg aatgtaggta gcggttatga tcgattcgat
63301 agaaaagaag gaatagtgtg tatttttcgt tggggatttc ctggaataaa tcgtcgcatc
63361 ttccttcgat tccttatgag agagattcaa tcgatcagaa tggaagttaa agagggtctt
63421 tatcctcgac gtgtccttta tatggaaatc agaggccagg gcgccattcc cttgacccgt
63481 actgacgaaa atttgactcc acgagaaatt gaacaaaaag ctgctgaatt ggcctatttc
63541 ttgcgcgtgc caattgaagt attttgaaat gaagggaatg aatgctttct cagcatgagg
63601 gaagggaccc aggaaccccc ttttaaatat aactgaagct tcttcggaac gttcattcga
63661 gcaaaacatg ttagattcta tttcccccgt tccgttggta atccttctgt ggcccataga
63721 ataaagcagg cggacgtata cggaacaacc ataataagaa gcaattttga tcgaccaaaa
63781 ctcctttttc tgcatataga actcaattct actagtaaca agtctaataa gtatgtattc
63841 atcacacata tcagagcatt tcggaataca taatttatct ttttaggacc aatactttgg
63901 attaatacat tagatacaga tgtatcatat cccgttaatt atctttcttt tgtcaatcga
63961 tgtttctttt ttgatccttt ctttagctcc tggataacca aacgtttaga tctctcataa
64021 ctatccaatt tctctctcgt tttgtcacct atttcggatt tcatcattaa tattttttcag
64081 aaatatcccc tcaattattc ccgggtcgtg ggtagccagt gaaaatttcg aaaaaataat
64141 tgaggggagt tctttcgtct cgaaatcaaa ataatattca ttatttcaag cggttctttt
64201 gggcattcat cgaaagaaca aatgaagata gaattggttc gaatttgacc aactgagata
64261 tctgggaaaa gtatttgatt atttcttcat tcgaaacggg cccttattct atttctattt
64321 ctatattctt tctagatcca aggactaaac aattcaaaaa aaaaaggaat agatccatag
64381 gttccatacc ttgttataga actcatgctt catagaaata tcggatcaga tagagtcggc
64441 gaatgaagcg ggttcattaa caattcacag atgaaaagtg tcaaaaaaga aagcattgac
64501 tcccctcccg tatcttgcat ctatagtctt tttgccctgg tggatctctc tctcatttaa
64561 taaaagtctg gaaccttggg ttactaattg gtggaatacc ggacaatccg aaactttttt
64621 gaatgatatt caagaaaaga acgttctaga aagattcgta gaattagaac aactattcct
64681 gttggatgaa atgataaaag agtacccgga gacacagata caaaagcttc gtataggaat
64741 ccacaaagag acgatgcaat tggtcaaaat gcacaacgaa gatcatatcc atatcatttt
64801 ggatttctcg acaaatataa tctgttttgc tattctaagt ggttattcta ttctgggtaa
64861 tgaagaactt gtcattctga attcttgggt tcaggaattc ctctataact taagcgacac
64921 aataaaggct ttttctattc ttttattaac tgatttatgt atcggattcc actcaccccg
64981 ggggtgggaa ctaatgattg gttcggtcta caaagatttt ggatttgctc ataacgatca

65041 aattatatct ggtcttgttt ccacttttcc agtcattcta gatacaattt tgaaatattg
65101 gatcttccat tatttaaatc gtgtatctcc ttcacttgta gtgatttatc attcaatgaa
65161 tgaatgaaga actcatttga tctgctgata tcaatcaaat catgatgcta cttcgtacat
65221 aaacaaactg ttttgaagct tactcactct ttatacttct acccgcccag gggttcccta
65281 ctatacttca gtacaattat tccagtacaa tggcagaatc atggataggg aactatgcta
65341 gctacctacc taatttattg tagaaatttc cgggatcaat tattggacca tgcaaaatag
65401 aaataccttt tcctgggtaa agaaagagat gactcgattc atttccgtat tgatcatgat
65461 atatgtaata actcggacat ctatttcaaa tgcatatcct atttttgcgc agcagggtta
65521 tgaaaatcca cgagaagcaa ccggtcgtat tgtatgtgcc aattgccatt tagctaataa
65581 gcccgtggat attgaggttc cacaagctgt gcttcctgat actgtatttg aagcagttgt
65641 tagaatccct tatgatatgc aaatgaaaca agttcttgct aatggtaaaa agggggcttt
65701 gaatgtgggg gctgtcctta ttttacccga gggattcgaa ttagctcccc ccgatcgtat
65761 ttctcccgag ctgaaagaaa agatgggcaa tctgtctttt cagagctatc gccccactaa
65821 aagaaatatt cttgtagtgg gtcctgttcc tggtcagaaa tatagtgaaa tcgtctttcc
65881 cattctttct ccggaccctt ctactaagaa agacgttcac ttcttaaaat atcccatata
65941 cgtaggcggg aacaggggaa ggggtcagat ttatcccgac gggagcaaaa gtaacaatac
66001 agtctataat gctacagcag caggtatagt aagcagaata gtacgtaaag aaaaaggggg
66061 atatgaaata agcatagccg atgcatcgga tggacaccaa gtggttgata ttatacctcc
66121 aggaccagaa cttcttgttt cagagggtga atccatcaag cttgatcagc cattaacgag
66181 taatcccaat gtgggtggat ttggtcaggg agatgcagaa atagtacttc aagatccatt
66241 acgtgtccaa ggtttgttgt tcttcttggc atctgttatc ctagcacaaa tctttttggt
66301 tctcaaaaag aaacagtttg agaaggttca attgtccgaa atgaatttct agatccacgg
66361 attcatcaag ttgataaaaa gggccgaatt attgttgatc aatgcaatta tgtatgatcc
66421 aaaaaaatat ggaaagcccc ttgtcttgct ttgtttatcc tgcttttctg cgagatgccg
66481 ggaattgctt gtatcccatt cccagtaata gtatgtatat tgcgaagaag actacttgac
66541 ccccccctt tttattttt ttttttcaat ccaaattgga gcggtgtgac gcttcttatt
66601 gccagattgt aaatgccatg gaatgcatca atagtttttt ctatctaata gaatcgaatt
66661 ctaatagaca ataggcggca taacattaag taggaagaga atacgcggca agggataaat
66721 gaaagaatga ttctgggagg gattacttgt cttcctaatt ttcgacacaa gaaaaggaat
66781 tttccgccct tttcttgtgt cgaaataata atgattcttg atcttgttcg tcaaagatta
66841 ctgttttctt ttccaggtct atcggaacct ctttctttag attcataaga agtggcggac
66901 aaacaaaaaa gggggatggc ttagtaaaca aatagaactt cttcaacgaa cttataaaat
66961 ttcaagtaaa aaaaaataaa attttaagat gagataataa ataaggattt ggatatgtgc
67021 aaaaatccaa gattttcccc tttcaaccgg aacattaaga gtcttttttt acttgatgaa
67081 attttatttt tatagaattt ttatagaata gagtagagta aggttcaatt aaattagtat
67141 agaaatggtt tgcaggatgt ctcatctgta gaaatcctgt gtcatccaaa aaatcaattg
67201 attccttctt tcttcttgtt tcggaagggg ccctcatact atggcggaac agatactatg
67261 aatcaatcaa gggattccat ttttcaaaaa catcatcaga aacaaagcat ccttttatca
67321 tttctatgaa tctaatatta tgattatgtt gactggatga atttccaact tttttatgt
67381 tatggaatag atcaaacaaa accttacccg aagagtaaga actcaatagg accttacccc
67441 tctttgtctg attcgggggg taaggtccta ttgagttctt actttttcat gtctacaatc
67501 cggctcatcc gattactata gggatgatcc caatccggaa tatgagccgt aaaagaaaat
67561 acctattgaa ccgatcacag gaataccagt tacagtacct ataagccaaa gaggaatcct
67621 tccagtagta tcggccattt acccacttcc ctccacattt catcaagtgg tcgtgctaga
67681 gacataaaca gtcatggata attatgagga tgatatcctt ccgaatggga taagagaatt
67741 cctactcttt ttctttattt tttctcaatt gaagaaataa ttggaaaata aaacagcaag
67801 tacaaaaatg agtaataacc cccagtagag actggtacga ttcaattcaa cattttgttc

67861 gttcgggttt gattgtgtcg tagctctata attcggatta ggtttatcgt tggatgaact
67921 gcattgctga tattgacccc aaaaaagaaa cagtaggtac agctagtccg tgaacagcca
67981 accatcgcac tgtaaaaatt ggataggttc gatctatggt cattgaggcc tcctaaaagg
68041 atctactaaa ttcatcgagt tgtgccaaag gatcaaaacg accagttatt aatggaattc
68101 cttgccggct ctctgtgaaa tattcgtttg gccgagggct tccaaacaca tcgtaagcta
68161 aacctgtgct gacgaataac caacccgcaa tgaataggga aggtatagta atactatgaa
68221 tgacccagta tcgaatactg gtaataatat cagcaaaaga acgttctccc gtgcttccag
68281 acatgctgag ctccacatat tcttgtacag tcaaacagag gatcgattcc gtgaaagatg
68341 ggatcagtaa atggaaaact actgatattt catccttgtg agatcgtcaa tagtgtaccg
68401 aaggtgtatt tagagtatac cgaatcagta tagctatcct tcctctgaca cagcaatgca
68461 ttttcaatca gtatcgaaaa gaaatggaat tcctttcttc ctttcttgtt ccttgtctat
68521 gcaaaaccgc gtgtcattca atagaaaatt cttaaatacc tgtaatatag ggtttccttt
68581 actggcttcg gaatagaaac tgaagatctt ggtaaagtat gagtcgacgg gttctaataa
68641 ttcatgatta ttatatttcc acaattcaat tagatgcaaa atttagaaac cccttttcat
68701 ggttgtcgaa aaggtatttt tttttttcta atcctttcat ttaaagtaat tggttggtcg
68761 tacagtagta gacagtagta ggtagtagta gatggaaaaa acagaacaaa cagtagttgg
68821 aacaattatc aatatttgtg atgcaaccat tgctgcatta gacccaaagg ttccttctta
68881 acccagctac aaggatggga ctgaacttta tgatatggaa agacagagtg cgaaacctaa
68941 actaaaagga taatagcaat tgctagtttt agaatgaagt tgggctcgaa ataaaggttt
69001 tatttcttcg agaaatcatg ggatactttt catttttctt ctcgttcgaa atattatgtg
69061 caattaacca acctactact gaatacaatt aagttaaaaa gtcaagtaaa agccgttatc
69121 cggctgtttg ttccaaaatg gattagataa attgaaaaag aaacgaaatg atcaaaaaaa
69181 tggaattttc aaatccaatt ctttatattc tgatagttac tcaaagagaa tttcattttt
69241 gaattgaatg aagttacaag acacagttct tattattagt actttactca cgggttgctc
69301 actgaatctg ttgattcgga atcatgggat ctgtagatgt tacaggcgac gaatccatct
69361 tttttttcta cccctcttac tctctctttg ttagtgccgt ctataatgga tgatgaatca
69421 agagctttca attggaactg attctgtcaa ttggtatttt ttcttgtcat tgtatctcgc
69481 aaaaatggaa acttaggtaa gtgctttaga accctatgta tgaaaaagaa gatatctcat
69541 ttagctcctc catgactact ataactagtt atttcggttt tctactggct gcttcaacta
69601 taaccccagc tctattgatt agtctgagca agatacgact tatttgaaat taattgaatg
69661 aacaattcat aaaaatgaat atttctgtga gattcccggt attctatagt tccttcccgc
69721 gttaattgcc aattcttggt tattgagatt catgggcgat tcggattaat atttagggac
69781 agatattacc tctcttttc tattctttca aagaaattga aatgattgaa gtttttctat
69841 ttggaattgt gttaggtcta attcccatta ctttggccgg attattcgta actgcatatt
69901 tacaatacag acgcggcgat cagttggacc tttaattgag taacatctct tttttttaat
69961 tgacctcctc cttaatctcc aggaggtcaa attcaggttg cagttcaagt tagtgaagtt
70021 attttattgt gattcaacat taaaagaaca gaatcacgct ctgtaggatt tgaacctacg
70081 acatcgggtt ttggagaccc acgttctacc gaactgaact aagagcgctt tattatgatg
70141 ggagatacgg atgtcaagaa aaggattctt tttgtacccc caatacatct tgtatgtata
70201 gtatcataaa atggtagatt gtgtccaatt ttaatcgatc tcaattgacc cctcgttact
70261 gtccatagga gaagtgataa gtagggatga caggatttga acccgtgaca ttttgtaccc
70321 aaaacaaacg cgctaccaag ctgcgctaca tccctttcat ttgttgtaca gtgccattgt
70381 agggaatcca tgttttgttt tccacatcat aatttcctct atctaaatag aatttctttt
70441 gccatttctt ctttttggtt ttttggtttc attctcataa agaattatat acatacctaa
70501 cgtataaacg tataaaggaa tgaatattta tcagtagtgc tcaggaagga gggttcatct
70561 ttttctgttt tagggacagg tagatttcat ctaccggatc gttgtatata tccattttgg
70621 ttagagattc cccgtacaaa tgatctttaa ctacatatgc atctgatcat atatgtatta

70681 caatatacaa taaagtcaat aaagttaact ttaaagaagg aggattttca atgcgagata
70741 taaaaacata tctctccacg gcacctgtgc taactactct atggttcggg tctttggcgg
70801 gtctattgat agagatcaat cgtttattcc cagatgcgtt gacattcccc tttttttcat
70861 tctagttatt gacatgggaa gggatcaaga agattagaga tacaataaat tctctgtgac
70921 taacccccc ctttttcagt tctttaggat aggaaagaaa gagtaaagaa taaaagtgga
70981 ttgaatctca tcgaaactcg ggttcgggtt aatataggga gaacagaaat ggaaatgtgg
71041 gtcgagggca ggctgttcaa gatcatacaa gatactaaat gaaatactgg gattgggaat
71101 aattgatagt tagaaatatt tgtattactt aataatttga ttactctatt gattgcaacg
71161 aaatctttca taattgaatt ggatttcgag ttagcaactt ctcgtctatt tattttttcat
71221 tcctttcttc ttcgcttcgg ttcgaatcga aaatagaaga attgagtgaa ttcaaaatcc
71281 aaaggaggtt catggctaag ggtaaagatg tcagagtagt agttattttg gaatgtacca
71341 gttgtgtccg aaatggtttg aataaagaat cgcggggcat ttccagatat attactcaaa
71401 agaatcgaca caatacacct agtcaattgg acttgaaaaa attctgccct tattgttaca
71461 aacatacgat tcatggggag ataaagaaat aaatcgaacg gaacgcgtgt gccactcttc
71521 caaggaagag gaagaaatta catatatata tataatatat acaaatccag tcctattttg
71581 gtcggatccg agatgaatga agaaatagga ttttagaaat aagaaataaa ccatggataa
71641 atccaagcgg ccctttcata aatccaagcg atcttttcat aggcgtttgc ccccaattgg
71701 atcgggggat cgaattgatt atagaaacat gagtttaatt aatcaattta ttagtgaaca
71761 aggaaaaata ttatctagac gagtgaatag attaaccttg aaacaacaac gattaattac
71821 tattgctata aaacaagctc gtattttatc ttcgttacct tttcttaata atgagaaaca
71881 gtttgagaga accgggtcga tccctagaac tactggtcct agaaccagaa ataaataagc
71941 ctattcctct caatcgaatc aaaactctaa ttcgaactca gattgaagtt ttgttcgaaa
72001 aagccgagag attgtcgcgc cgtaatgtaa taataaaaaa aagaatgggg gaagaataaa
72061 tctttttttt ttattgaaac gtgttcgttc attcctacta cttatcttat catacgaatt
72121 tctactatac cctcccggag ttcattctcc ggggaactcc gtttaaagta ttccagtgaa
72181 ttccttccaa tctccttatt tgatgatcgc attggaaatc gtgtcaagac aattcctatt
72241 tgatatggct atttgtgcag gtattttacg attaagaagc aactgcctct tgtacagatc
72301 aagtattaat cgactataac tatgggatcc cctattctca cgagttactg catttatccg
72361 agtgatccac aaacgacgaa aacttctctt tcgcctgcct ctatcccgat gagtggaaac
72421 caaagctctc attttctgtt gagtagtagt tcgagtaagt cttgaatgag cccctcgaaa
72481 ggttgctgca aataaacgaa tttttgttct acgtctccga gctatatatc ttcgtctaac
72541 tctggtcatt gaatcaaaag aaactttgat gaataactaa ttgatttctt ttctttcagt
72601 cattcttttc ccctctcctg gtttattaat aacaaaacgg attcttccga tgtataaaat
72661 taaaatacaa attccaatgg cttttgctac tataaccttc ccaaccacga tttttttatt
72721 tcttccaggc atttcacctc aaaaaaaaaa aaaagaaatt gtaccgatat taggtataaa
72781 ataaatcgta aatggacaaa tagtggcttc catcgtttct atggttactt cttaaacggc
72841 gaggtcctct ctatacaccg gagccccttt cctcatttaa tcaatgttat tggtaacttg
72901 tacagttcac gctctttggc tctaccgatg aattatcgag taataggtct tttttcaatg
72961 ggatctatcc atacagtgac ggcatttaat tatgaaggtt gaataggtag ctgaccctgt
73021 tagtccgttc ttgcaagagt aggagcataa tctttctgct tcttaaatat cattcccccg
73081 cttaatggat aaccatttgc taccaatggg aattgctttt catctcaaat cgaggtgatt
73141 ggatttgcac caatggaaac cataaattcc atacaaatag aggtatacga gagatcttta
73201 tttttcgata gtgaatggag ttcttccatt ctattcattg gtacgagtca ttgatactgt
73261 aaaagtcgtc tcatttgttc tagctcatga tctgaacgag tcgcacatac accctagtac
73321 atgttcctcg acgctgagga catcctcgaa gagcggggga tttcgtgaca tttctgattg
73381 gctgtcttgt atttctaata agttgtttaa tagttggcat gctgaatcat atacagaatg
73441 ggctggttta gatcgatcct aaccggatga ttatgaatta cttctttacc caggtaagaa

73501 gataaaagat caaataaggg ttcattcaaa ttatgattcg aaatggaatc aaagatttat
73561 gcgaaatccc cggtattttc gatcgctaca agatcaacaa tgccataatc ttgggcttct
73621 gttgctgaca taaaaacatc cctttccatg tcttcggata caacccataa aggattgccc
73681 gttctttgta cataaaccct tgtgagggtt tcgcggagtt tcagtagttc ttccgcttcc
73741 aggataaatt ctcctgtggg tgcctcataa aaagaactag caggttgatg gatcataacc
73801 ctgatgatat aacataatga acgattcctc tatctcgcat gattgggcga agggaaggga
73861 taaagaataa caagcaggga gaaaaagata gaattgaaca accgtacagg catcttttgt
73921 gcatacggct ctgtaatgga attttttttt ctctttttc atcgaagaaa gagacaagtc
73981 gaatctatca gacccagatc gttgaatgat ccatttacca tccttccttt cggagtaatc
74041 aaaaaatact atgatggttc cgttgcttta tatatttatc tcgtctgtga ttcagcaatc
74101 ccaaagtttc tttctgatcc gatcaaataa aaataagtaa aaaatgatct tttttttttt
74161 tttattttca cactctttca taacataaat attggaaaga gacttctgat gtggaagcaa
74221 aaaggtttgt gacgctgaaa tggaccccga tacataagat caagtcggaa ataacctttc
74281 tttcatacta ctatctcgat acataatctc atattatgaa aaaataataa tagtttgctc
74341 atatcgaact tgaaatgcca tgctattatt acttaatatt attattattt tattcatatt
74401 ccatatgacg aaggcatagt cttttttttct ctcaaataaa aaaactcatt ggcgccaagc
74461 gtgagggaat gctagacgtt tggtaatttc tcctccaacc aggatgaaag atcccattga
74521 agcggctaat cccatgcata ttgtatgcac atctggtggc acaaattgca tagtatcata
74581 aatagctatt cctgggatta cccatccgcc gggagaattt ataaacaaat acagatccct
74641 ggtatcatcc tctatactga gatataccat gagaccaaca agttgattcg agatctcgct
74701 atcaacttct tggcctaaaa aaagtaatct ttctcgatga agtcggttga ttaggacaaa
74761 attctattcc ttaggaaccg tacacgcacc tttgggtgca tacggttcaa aaaatcaaaa
74821 ttgagaaaaa aaaaaagaaa ttgtcgattc cagccctatt tctttttttcg tagcgggctt
74881 tttcttccat tttttaagaa acatgagttt tgacttgctt ccctataaaa tcaaaaaaga
74941 attcactgaa cttatcgagc taacccctca ttgatgtatt gtttcatcga gatctaaatc
75001 acgatgtaat tttcttgttc ccgaatgggc ctcttccact cttttaggtt tatgctctac
75061 tccgggtaaa gatctgcccg aattcgattt gcacatatag gacaaatgat cccagtacca
75121 cttctttttg ctatgacttc tttttttttt tttcaatttg tttcattttc atgccttcca
75181 caaaatattc gatgtattca tcatattatt ccattaattg gcaatttggg atcactcata
75241 tggtataaag gaatcatttc tgatagggtg gtaatcatac atggattacc ttggtatttt
75301 ctgaacggag cctgtatact tcattttatt ggtccaagcc aaccataaat tcttttaatt
75361 gagaatattg atcctccaac caaataaatt gatctaattg cacttcacgc ttcgaattat
75421 tgatggttca atcaatcttt cttgggcgaa acagaggata tctcgatcgg gggagagaac
75481 ggggaaatcc catatgaccc aatatgtctg acaagtcaca ctatacgtca acccaaactg
75541 catcttcctc tccaggactc cgaaaaggta cttttggaac accaatgggc attaaatgaa
75601 agaaaaatga agtattctat ttcactttga tgtggaaacg taacaaacaa tggtttattg
75661 tcttcataat attgtcgttt atcgtatttt atcgatagat tggaagattc atagaggaag
75721 acggaataaa ggaaaattct tacgaacgga tcgttcgaat gagaaacaag tatctataca
75781 ttcgctcaca aaaaatagga ttaatccccc cattgcgtat tggtacttat tgggtataga
75841 atagatctgc ttctctttgt tcctacgaac agaattgttc aattattact aacggaacag
75901 aataaatatt aacccttgtt tcgagataat ccaatgaaaa ggtgaggtcc atagcatagt
75961 tatttccaat gtgataaagt tacatagtat ctattttatc tttgagaaag ggtatttcc
76021 atgggtttgc cttggtatcg tgttcatacc gtcgtattga atgatcccgg tcggctgctt
76081 tctgtccata taatgcatac agctctagtt tccggttggg ccggttcgat ggctctatac
76141 gaattagcag tttttgatcc ttctgacccc gttcttgatc caatgtggag acagggtatg
76201 ttcgttatac ccttcatgac tcgtttagga ataaacaatt catggggcgg ttggagtatt
76261 acaggaggaa ctataacgaa tccgggtatt tggagttacg aaggtgtggc cggggcacat

76321 attgtgtttt ctggcttgtg cttcttagca gctatctggc attgggtgta ttgggaccta
76381 gaaatattct gtgatgaacg tacgggaaaa ccctccttgg atttgcccaa gatttttgga
76441 attcatttat ttctctcagg ggtggcttgc tttgggtttg gcgcatttca tgtaacaggc
76501 ttgtatggtc ctggaatatg ggtatccgat ccttatggcc taaccggaaa agtacaatct
76561 gtaaatccag cgtggggtgc ggaaggtttt gatccctttg ttccgggagg aatagcctct
76621 catcatattg cagcaggtac attgggtata ttagcaggtc tattccatct tagtgtccgc
76681 ccacccccaac gtctatacaa aggattacgt atgggcaata ttgaaactgt cctttccagt
76741 agtatcgctg ctgtcttttt tgcagctttc gttgttgctg gaactatgtg gtatggttca
76801 gcaactaccc cgatcgaatt atttggtccc actcgttatc agtgggatca gggatacttc
76861 cagcaagaaa tatatcgaag agttggcgcc agtctagccg aaaatctgag tttatcggaa
76921 gcttggtcta aaattcccga aaaattagct ttttatgatt acatcggtaa taatccggcg
76981 aaaggtggat tattccgggc aggctcaatg gacaacgggg atgggatagc tgttggatgg
77041 ttaggacacc ctatctttag agataaagaa gggcatgaac tttttgtacg ccgtatgcct
77101 acttttttttg aaacatttcc agtagttttg gtggacggag acggaattgt gagagccgat
77161 gttcctttta gaagggcaga atcgaagtat agtgtcgaac aagtgggtgt aactgttgag
77221 ttctatggtg gcgaactcaa tggagtcagc tatagcgatc ctgctactgt gaaaaaatat
77281 gctagacgtg cccaattggg tgaaattttt gaattagatc gtgctacttt gaaatccgat
77341 ggtgtttttc ggagcagtcc aaggggttgg ttcacttttg gacatgctac gtttgctttg
77401 ctcttctttt tcggacacat ttggcatggc gctcgaacct tgttcagaga tgtttttgct
77461 gggattgacc cagatttgga tgctcaagtg gaatttggag cattccaaaa acttggagat
77521 ccaactacaa ggagacaggt agtctgatac aacattgctc cggtatcttt cgcctctata
77581 tttgattttt ttgatttgac ataaggtacc gtagaaatat tgatttgaat catcgccttt
77641 ctttgctctt gtcctttctt tatctgggaa ataatcctaa atgaacaggt gtggaagcta
77701 taattgtaaa caacgatcga atctatggaa gcattggttt atacattcct cttagtctcg
77761 actttaggga taatcttttt cgctatattt tttcgagacc cgcctaaggt cccgactaaa
77821 aagacgaaat gatttttcat tatcttaatt gaagtaatga gtcccccata tgggggactc
77881 attacttcaa ttagtctccg tgttcctcga atggatctct tagttgttga gagggttgcc
77941 caaaagcggt atataaggcg taccctgtaa agcttacaag tgaaccagat atggagatgg
78001 cgactaaggt tgctgtttcc attattagag aatttcaaga ccacgatgga tctatgctac
78061 gataagatcg tttatttaca acggaatagt atacaaagtc aacagatctc aaccaatgca
78121 atagtattta tggctacaca aaccgttgag ggtagtgcta gatctgggcc aagacgaact
78181 attacagggg atttattgaa accattgaat tcagaatatg gtaaagtggc tcctggatgg
78241 ggaaccaccc catttatggg tgtcgcaatg gctctatttg cgatattcct atctattatt
78301 ttagagattt ataattcttc cgttttactg gatggaattt caatgaatta ggtccataag
78361 aaccagaagc cctagctttt caatcaaaaa tgaatcactt aggactcaga tttatagtcc
78421 attctggtag tttgaccgtg gaattccgtt gtttcggtat ttccggaata tgagtgtgcg
78481 acttgttata attgatccta ttgatagtac agagaatggg tctgtcatct cgacagagat
78541 ggttctgcct cgtcggatat tcatcctagt atctggagca cggaatatat ggaatagatc
78601 aagaaatatt tgaactatga ttcataccta ctattcagac ctcgtgactg gacttccaaa
78661 aattttcaaa caaagaggta tttgataaat tgaacgattt ttcttccttt agaatcatgc
78721 ttattttgac cgaaggacaa atctttctct ggatttttag tcattacatc tatgaataag
78781 tgatgatcaa atagttctta ctcatagaac ccttggtctt agtttttggg ttttattgaa
78841 tcatcgtggt tctagtatga atctgaggtt tcaatcgatt cataggctct caacaagaga
78901 attcctatca aaaaaaaata gtaaacaata gtcaatctgc attacgcaca aacaaaaaca
78961 acaaatcaaa taacaaataa atagggggaat agaagattca agaggcctgt aacgatcaac
79021 ataaagacag atgagctaac ttgatatttt ggcattctca tcacaacaaa gaagagagtt
79081 cggattttgg ttccttcgta tcttcagaga cgattgaatc aagtggataa ataagaaatt

79141 tcaaattttc tattacatat ccattgtaat cagtatttgg gtgtttctgc ttgagccgta
79201 cgagatgaaa ttctcatata cggttctcag agggggagtc cccttggttt acctatatga
79261 gtaaagtata tgattggttc gaggagcgtc tcgagattca ggcgattgca gatgatataa
79321 ctagtaaata tgttcctcct catgtcaata tattttattg tctaggaggg atcacactta
79381 cttgtttttt agtacaagta gctacgggtt ttgctatgac tttttactat cgtccgaccg
79441 ttacggaggc ttttgcctct gttcaataca taatgactga agccaacttt ggttggttaa
79501 tccgatcagt tcatcgatgg tcagcaagta tgatggtcct aatgatgatc ctacacgtat
79561 ttcgtgtgta tctcacgggc ggatttaaaa aacctcgcga attgacttgg gttacgggtg
79621 tggttctggc tgtattgact gcatcgtttg gtgtaactgg ttattcctta ccccgggacc
79681 aaatcggtta ttgggcagta aaaatcgtga caggcgtacc tgaagctatt cccgtaatag
79741 gatcaccttt agtagagtta ttgcgtggaa gtgctagtgt gggtcaatct actttgaccc
79801 gtttttatag tttacacact tttgtattac ctcttcttac tgccgtattt atgttaatgc
79861 attttccaat gatacgtaag caaggtattt caggtccttt atagagaaga cagatcatag
79921 atatttgtaa tcgatcatat ataatttcgg ggaggaacaa tagtgtttta ttgctacaaa
79981 tatggattat tgaaaagaat aagacatctt tttggatatt tctcttcaac taactacgaa
80041 gtattgtatt ctttatttga tacgaatagt tgaagtacat tctccgaaga gaagatggat
80101 tatgggagtg tgtgacttga actattgatt gggccgtgca gatatatgat tttatccgcc
80161 acattggaat tcacaaccaa atgtgtctct gttccaacca ccgcgtaggt ccccctacag
80221 aggataggct ggttcgcttt aggagaatct tttctatgat cagaccaaat catgttatgt
80281 tgtgcatgaa cgggctccgt aagatccaat agaataaaat aaaatgaagt aatgtggcat
80341 gatccagatt atgttttatc tatttactta aagtatggaa atgcattcat ttcctctgca
80401 tcgatcccaa tctatgatac tatcggagtg aaacaaggga tctaaggaag aacataggct
80461 agactttatt agtaacaagg aaatcctttg tattaagaag actcgagata ttgtggggat
80521 aaacactaat cacaaagcat gagaccatcc aaaaagcatt tgatcatgat caaatttgga
80581 agcctacttg ggtattgagc atttacttgt aagaactgaa ttccttgcaa tgggtagttg
80641 caaccccgta aaattgaatc cggtaaatct tttcttacat agagtcatat atgtgtggat
80701 gatatatcta ttttatatgg acccgtttta ttcttttgat tcttgctcga gccggatgat
80761 aaaaaattat catgtccggt tccttcgggg gatggatcta taagaaagaa ttcacctatc
80821 ccaataacaa agaaacctga cttgaatgat cctgtattaa gagctaaatt ggctaaaggg
80881 atggggcata attattacgg agaacccgca tggcccaatg atcttttata tatttttcca
80941 gtagtcattc taggcactat tgcgtgtaac gtaggtctag cggttctaga accgtcaatg
81001 attggtgaac cagcggatcc atttgcaact cctttggaaa tattacccga atggtacttc
81061 tttcccgtat ttcaaatcct ccgtacagta cccaataagt tattgggtgt tctcttaatg
81121 gttttagtac caacgggatt attgacagta ccgtttttgg agaatgttaa taaattccaa
81181 aatccatttc gtcgtccagt agctacaaca gtttttttga tcggtaccgc ggcagccctt
81241 tggttaggta ttggggcaac attacctatt gataaatctc taactttagg cctttttttaa
81301 gttgatttaa ccgtgaaata ctacgcgtat gtatctaggg aatagtcact tctaaagtga
81361 attctcccta gatacatctc ttaaatttca ttatcaatcc attctggata tagagatgat
81421 actaaggatt caaaagccat tttcttcttt tctttctttc aaaaaagatg aaataatacc
81481 aatggattta aaacttattc ttaggtaaat aaattgcaaa atgcttctgt agaatgtcca
81541 atatctgttt tacatcttct atgcgaagat gttcaattct cataagatct tcttgactgt
81601 tattcaaaag gtccaataat gtatgtatat tggacctttt gagacaatta taggtcctgg
81661 agggcaattc tgattggtca ataaaaatac atttcagtgc aattcctttt ttgtttttcc
81721 ttatattagc caatctatca tgaaaagtaa aaaacgatac agtaaacctg ttttgattgt
81781 cttctaaata aaaatgaatg tcctcttcct ccgcatgtag aaaaggaata aataagtcaa
81841 tcaaagtacg ggaagcttcg cgaagtgctt ctttaggagt taaacttcca ttcgtccata
81901 tttcaagaaa aagtatctct tgtttctcat tctcactccc ataagaatga atactatgat

81961 tcgcatttcg aacaggcatg gatacagcat ctataggata acttccatct tgatagttat
82021 tgggggattt catacgatat ccgcgatccc tctcgatttt gaattcaata cacaaatgaa
82081 ttggctccgt cagattagct atatgctgtg tagtatcgac tagttccaca gaaggcggtg
82141 agatgatatc ttgagcagtt acgtatttag gacccctgac gcaaatggat gcgtcacgag
82201 ttccatacag attacttctc aatacaattt ctttcaaatt cattaaaatt tcatgtactg
82261 attcttcaat accaactatc gtagaatatt catgtgatac cttctcagat tttgcacgtg
82321 tgatacatgt tccctctatt tctccgagta aagcctttcg catcgcgata cctatcgtat
82381 ctgcttgacc tttcataagc ggggacagaa cgaaacggcc ataataaaga cgcttactgt
82441 ctgttcttga ttcaacacac ttccactgta gtgttcgagt ggatactgct acttcttctc
82501 gaaccatact aatattattg tttgatcaga tcattgaatc atttatttct attgcaatcc
82561 attccatttt tatttctaca cacgtctttt tttaggaggc ctacatccat tatgtggcat
82621 aggggttacg tcacgtacga aacttaatag tataccactt ctacgaatgg ctcgtaatgc
82681 tgcatctctt ccgagaccag ggccttttat catgacttct gctcgttgca gaccctgatc
82741 cactgcctta cgaatagcat ttcctgctgc ggtttgagca gcaaatggtg tccctcttct
82801 tgtgcctctg aatccacaag taccagcgga ggaccaagaa accacccgac ctattacatc
82861 tgtaacagtc acaatggtat tgttgaaact cgcttgaaca tgaataactc ctttttgtat
82921 tctacgtcca ttcttacgtg aaccaattct tggtatagct tttgtcatat tttatcatct
82981 cataaatatg agtcagagat atacggatat atccatttca tgtcaaaaca gatcctttat
83041 ttgtacatcg gaccgtttag aaagtccctt gttagaaaga ttacccctgt ctctgtttat
83101 gtttcggatt ggaacaaatt actataattc gtccccgcct acggatcagt cgacattttt
83161 cacaaatttt acgaatggaa gcccttattt tcatatttgt tattccttaa ttccaaatat
83221 actccttgga agaaaataag tctcttcaaa ttttgaacct cgaattgtat tcccatgaaa
83281 ggaatgttta aattcaaata aaaagccgcc taatcattcg actctttgtt gcgaagtcta
83341 taaattatac gtcccctagt tgaatcataa cgacttactt cgattttgac tctatctcct
83401 ggtagtatcc ggataaaact ccgtcggatc ctccccgaaa cataacctag aatcagatct
83461 tcattgtcta aacgaactcg gaacatacca ttgggaagtg attcagtaat taaaccttcg
83521 tgaatcaatt tttgttcttt cattccaggt aaccccccttg aagtatcaac taatggagga
83581 ggagtaatag tagacaattc gtctttcctc tctttttccc aaatagcaag ttacggatca
83641 aattcggata ccagaaggat caccagatat aatacaaaat ttctccccca attctttcta
83701 gtcgagcttc tcgatctgtc attatacctc gagaagtaga aagaattacg acccccattc
83761 cacctaaaat cctaggaatt cgttgatggt tggaatagat tcgtagaccg ggacgactga
83821 tacgctttaa aatatttcta tatgttcctt tcctattcct tctatgtcgc agggttgaaa
83881 ccaagaaata tttgttgttt tccctatgtt tcctaacatt ttcaataaac ccttcttgta
83941 gaagtatttt aacaatgttt tcggcgatat tagtagatgc tactcgaacc gttccttttt
84001 tatccatgtt agcatttctt atagaagtta ttatatcggc aatagtgtcc ctacccatga
84061 cgaactaaat ttatgggtgc cttccagttt tgatataatc aacatgttcc tttttttttt
84121 ttcatttttt cttatttatt tatgaattat taaaggtata tgcgtgagac acaatctact
84181 aacgtgatct atttcagaga cctgactata ctctatcacg gtctcatcta ctagtattta
84241 taagacttca ggagctaatg agactatttt agtgaaattc aactgtctca attcccgcgc
84301 gatcgctcca aaaactcgag ttccttttgg atttccttct tgatcaatga caactgctgc
84361 attgtcatca tatcgtatta tcataccgtt gtcgcgtttg agttctttac atgtacgtac
84421 aattacagct ctgatcactt ctgatctttc gagaggcata ttgggcactg cttctttgat
84481 tacagcaaca ataacgtcac caatatgagc atatcgttga ttactagctc ctatgattcg
84541 aatacacatc aattctcgag ccccgctgtt gtccgctaca ttcaaatgag tctgaggttg
84601 aatcatatca tttttttttt tttgaatctg ctctttcaat gcaaaaggca aaggaaaaag
84661 agagaaatat tgtctgccca gaaatccaaa aatctgcgat tgtatttttc atcacaaata
84721 cccttcacat acctatcacg cgataatgaa ttgagttcgt ataggcattt tgcacgcagc

84781 tattgaaata gctgctctgg ctacagtttc tgatactccg cccatttcat aaagtattcg
84841 accgggttta acgacagata cccaatattc gggagatcct ttccccgaac ccatacgtgt
84901 ttcggtaggt cttactgtaa cgggtttgtc gggaaatata cgtacccata tttttccacc
84961 acggcgtgca tatcgtgtca ttgctcgtcg tcctgcttct atttgtctag atgtgatcca
85021 agcgggttca agtgcctgaa gagcgtatct gccgaaacaa atatgattgc ctcgataaga
85081 tattcccttc attcttcctc tatgttgttt acggaatctg gttcttttgg gactaagcat
85141 gatggttgtt tctcaatccc atctctactg cagaaccgga catgagagtt tcttctcatc
85201 cagctcctcg cgaatgaaac gattcaataa gattacgtat atgtatttat tgaatgaata
85261 atacactgaa tcatggaatt tcttgatatt taatctgtca cacgggaagc cgtatagtat
85321 atagtatata cggctagacg gatatttcta ttttatttat atgggataat gcctttcttt
85381 tgaaaatgaa tccttgacct ttaccgaatc tgtcaaaata ctacaatcca ataatggttt
85441 cgcgggcgaa tattgactct ttccatattt gcttcattcg tagggtgaac ccatgaccta
85501 tcagaagaaa ttaattggtt cctggttgat tccgccatcc cacccaatga atcattagga
85561 ttcgttttca atagaatctt ccgcagtcac aggtttcgtc gttcccatag cttttccatt
85621 aatggctagg cctgaactat gcaatggagc tcctaattaa attcgttccc gagccaatct
85681 cctcagtctc tattgactcg gggctcttta ttatttgtat ttttcttatg aaccgtattc
85741 atctaattat ggacgaatca gtattgatgc tttatcacac tgccttttat gatatgatgt
85801 gattgataga ccatacatat tggaatcata tatcatggag attctccttc tctctttctc
85861 tcgcccttcc agttacccac atccctctat ttttctttcc aacctataaa tggatttttc
85921 ctttatggaa aaaaaaagat ttcagttgct acaactatat gatcgataca tcatatggcg
85981 actgcttcct tggatctcga taatacaaag caatgagttg gttactagtt cttatagtta
86041 ttagttaggg gctggtctgt tttttgaatc ccaactttaa ataaaaaacc aacgagtcac
86101 acactaagca tagcagttcc accaaaaggt caatcgaatt tttattcaac cttatagaat
86161 tagaattgct catttttcat tttttttttt attgaagtga aaaggaatag tttgtagttt
86221 ttgttctatc actgaataga atggcaagca aaggaagggt ccattattgc tcgtctacaa
86281 atatccaaat tttgatgccc aatgccccat aggcagttcg aactgtatag gaacaatgat
86341 caattttagc tcgaatggtt tggaggggaa ccctaccttc tctgatccat tcgacacgtg
86401 caatttcttt tccgtcgata cgccctgcaa tttccacttg aattcctttt gtgtctgctt
86461 gttcagttaa ttcaatagct tttttcattg cctttcgaaa cgaaacccta ttctttaatt
86521 gtagagctat atattctgca agaatattag gttgtccata aggttttgca actcttgtaa
86581 tagcaatgtt aagtctccga ttcacagaat gaagcccctt ttgtacattg atctgcaatt
86641 cttcgattcc tcgtgtttgg ccttctatta acaaatttgg gaatccaata tagattatga
86701 cctggatcaa gtccattttt ttttgaatct ctatatgtgc aattccaatt ccttcgaaac
86761 ttgaagatac tcttatattt ttttgtacat atatcttgat ccaatcccgt attttttcat
86821 cttcctggag acctatgtaa taactttttg gttgtgcgaa ccaaagggaa cgatgacttt
86881 ggttttcgcc aaggcggaaa ccaagtggat ttattttttg acccatcttt tttttctctc
86941 tctctctcct tctctatata tctttctatt ataggatctc tccatacatt ttttgtttct
87001 aaaaatatat cctgatctgt tttcttttta gagctatcct tcaatacaat aattatatga
87061 caggcgggtc tttctatcgg ataactacgt cctctagccc tgggttttaa ctttttcacg
87121 atagtacccc cattgacttc ggctttacta atgaccgaat cagcttcgtt gaaactttta
87181 ttgtgactag catttgctgc tgcagaataa accagtttaa aaatgggata aaatgctcga
87241 taaggcatga gttccagtaa cataagtgtt ttctcatagg aatgcccacg aatctgatca
87301 atgactcttc gtgctttgtg agcagacata catatacgtt gagctaaagc ttgtacttgt
87361 gtactcgagc tcctcttcat cttctgcttt ttcaaggtct tcttccactt tctccacttt
87421 gacataagat aaggttcgcc tcccgccaat gaacgatgag cacctatttc actttatttt
87481 attaacggag agatctagta tcgtttctcg cgtgtccctg gaaagtaaga gtaggtgcaa
87541 attctcctaa tttttgaccc accatacgat ccgttatata aataggtaaa tgcgcctttc

87601 cattatgaat ggcaattgta ttgccgatca ttgtgggtat aatggtagat gcccgggacc
87661 aagttactat tatctctttt tcctctctca tgttaagctt ctttattttt tccaataaat
87721 gattagctac aaaaggattt ttttttagtg aacgtgtcac ggcctattat tccccccccc
87781 tttttttttt gaaaagacga gtaaaaaaca atatttattt gattttgaat attcctattt
87841 acggcgacga ataataaaac tattactata tttattcctt ttcctacttt ttcttccaag
87901 cgcaggataa ccccaagggg ttgtgggttt ttttctacca atcggggccc tcccttcacc
87961 acccccgtgg ggatggtcta cagggttcat aactacccct cttactacag gacgcctacc
88021 tagccaacat ttagatccgg ctctacccaa acttttctgg ttcgccccaa cattacccac
88081 ttgtccgact gttgctgagc agtttttgga tatcaaacgg acctccccag atggaaatct
88141 taatgtgacc gatttaccct cttttgcaat cagtttcgct acagcacctg ctgctctagt
88201 taattgtcca ccctttccaa gtgtgatttc tatgttatgt acggccgtgc ctaagggcat
88261 atgggttgaa gtagattttt ctttttgatc aataaaaacc ccttcccaaa ccgtacaagc
88321 ttcttccaaa gcatacggct ttccggatgt atatatatat tctatgatga tatctagaca
88381 gatggatttt atatgaatcg tgtgatgaag taccacatga gtggatatat aggaatacaa
88441 atctgccaaa tcactcatgt tatgatctta tacatcctag gtctctccgt ttcgtcatct
88501 ggcttatgtt cttcatgtag cattcagacc gaatgactct atgaaattac gtcgctactt
88561 ccacatatta cgggtaacgt aggagacatc tctatttttc cccgggggaa tttttagaat
88621 taccaccact tagctttcaa ttcacctctg accatcaaat gaaatgtgaa taacccatcc
88681 tcttctcttt gaaacaaggg tcgcttccgc ttctgtccgt gcttcaaaca attttgtctt
88741 ctccatatta ccatatctgg agtgtcaata gttttctatg aggaactact gaactcaatc
88801 acttgctgcc gttactcttc agttttctgt tgaggtctat cccgtagagg tactcaaatt
88861 ggatcagtga tcaatttcta ggtttcgtcg taaacctaat tggttacttc caattacgta
88921 aatccatagt tcaaaccgca ctcaaaggta gggcatttcc cattgatata ggaacttctg
88981 taccggaaac aatggtatct ccaattatag cccctctggg atgtaaaata tatcttttct
89041 caccatctcc atagtgtatg agacaaatgt atgcatttcg attagggtca tattctatgg
89101 ttacgatcct agcagatatg tctttttcat tccgtcgaaa atcgatttta cggtatagac
89161 gcttatggcc tcctcctcta tgccctgcgg taatgattcc cctggaatta cgacctttac
89221 cgcagcgatg ctgtccatag atcaaattat ttcgcggatt ggatttcgct tgactgtcta
89281 tggatccttt gcgtatgctc ggggtagaag ttttgtataa atgtatcgcc gtgttattta
89341 gtattttttt tatttttttt ttacttaaat tcttttctct tctctataag aggtaaaata
89401 gaataacccg gttgaagcgt aatgatcata cgtctgtaat gcattgtatg tcccataatg
89461 ggtcccgttc ttctaccctt tcccgggagt tgatgactat ttatagctat tactttgacg
89521 ccaaagaaga gttcgaccca atgctttatt tctgtcctag ttgatcctga ttcgactttc
89581 ttccccacga gttccagtat cgataagaat tctagttctt actcttcata tgttatggtt
89641 ggtatgaata taccatacca attcgttatg tatggatgat gagattccat tgatacggag
89701 ccagtggaat tagtcttatt gaatgtcccc gttggcctgc atccagcagg aattgaacct
89761 acgaattcgc caattatgag ttgggtgctt taaccattca gccatggatg cttcactggg
89821 gtcattgtac atcgcgagtg acccaaattc aattcactta gattcttttg gattctttag
89881 gaggaatcaa tgaaatgaga ggacatcaat tcaaatcctg gatcttcgaa ttgagagaaa
89941 tcaagaattc tcacgatttc ttggattcat ggatccaacc cgattcggtg aaatctttca
90001 cttccttttt tttccaccaa gagcgcttta tgaaactttt tgattcccga atttggagtg
90061 tcctaatttc acgtgattca cagggttcaa ttcgtcgaca ttgcatgatc aaaggtgtag
90121 tactgcttgt acttgtagta gcggtcctta tatacaatcg aaatagggtc gaaagaaaaa
90181 atatctattt gatggggctt cttcctaaac ctctgcgttc cattggaccc cccaattata
90241 cattgaaaga atccttttgg tcttccaatc tcaataggtt gattgtttcg ctcctgtatc
90301 ttccaaaagg gaaaaatatc tatgagagtt gtttcatgga tccgaaagaa agtacttggg
90361 ttcttccaat aactaaaaag tgtatcatgt ctgaatctaa ctggggttcg cagcgatgga

90421 ggaatgcgat cgtaaaaaag aggaattcca gctgtaagat atcgaatgaa attgcagctg
90481 gaattgagat ctcattcaaa gagaaagata tcaaatatct ggagtttttt tttgtatcct
90541 atacgaatga tccgatccgc aaggaccatg attggaaatt atttgaccgc ctttctccga
90601 gtaagaagcg aaacataatc aacttgaatt cgggacagct attcgaaatt ttagtgaaac
90661 atttgatttg ttatctcatg tctgcttttc gtgaaaaaag accaattgat gaggggggtt
90721 tcttcaaaca acaaggagct gaggcgacta ttcaatcaaa cgagattgaa catgtttccc
90781 atctcctctc gagaaacaag gggggtattt ttttgaaaaa ttgcgctcaa tttcatatgt
90841 ggcaattccg ccaagatctc ttcgttattg gggggaagaa tcggcacaaa tcggattttt
90901 tgaggaacgt ctcgagagag aatttgattt ggttagacaa tgcgtggttg gtaaacagga
90961 atcgggtttt tagcaaggta cggaatgtat cgtcaaatat tcaatatgat tccataagat
91021 ccattttctt tcaagtaacg gattctagcc aatcgaaagg attttctgat caatccatag
91081 atcctttcaa ttccattagt aatgagggtt cggaatatca cacattgatc aatcaaacgg
91141 agattcagca actaaaaaaa agatcaattc ttttagatac ttcctttctt caaacggaac
91201 gaacagagat aaaatcagat cgattctcaa aatacctttc cggatattcc tcaatggctc
91261 ggctattccc ggaacgtgag aagcagatga ataatcatct gcttccagaa gaaatagaag
91321 aatttcttgg gaatcctaca agatcaattc gttctttttt ctctgataga tggtcagaac
91381 ttcatctggg tttgaatcct accgagaggt cgactataga tcagaaattg ttgaagaaac
91441 aacaaggtgt ttcttttgtc ccttcgaggc gatcggaaaa taaagaaata gttgatatat
91501 tcaagataat tacgtattta caaaatacct cctcagttca ttcgattgca gcagatccgg
91561 gatgggatat ggttccgaag gatgaaccgg atatggacag ttccaataag atttcattct
91621 tgaacgaaaa tgcatttttt gatttatttc atctattcca tgatcggaac aagggggggat
91681 acaggttgca ccacgagttt gaattagaag agacatttca agaaatggca gatctattca
91741 ctctatcaat aaccgagccg ggtttagcct atcataataa ggaatttggc ttgtctattg
91801 attcctacgg aaaattattg aatgaggtat tcaactccgg ggatgagtcg aaaaagaaat
91861 ctttattggt tctaccttcc atttttttatg atttattttt attggttcta tcttctattt
91921 tttatgattt atttttattg gttctacttt ctatttttta tgatttattt ttattggttc
91981 tactttctat tttttatgaa gagaatgaat ctttttatcg aaagataaaa aaaaaatcgg
92041 tccggatctc ctgcgggaat gatttggaag atccaaaacc aaaaatagcg gtatttgctc
92101 acaacaacat aatggaggcg atccatcaat atagattgat ccgaaatcag attcaaatcc
92161 aatatagtac ctatgggtac ataagaaatg tattgaatcg attcttttta atgaatcgac
92221 ccgattgcaa cttcgcatat ggaattcaaa agcatcccat aggaattcaa aagcacccaa
92281 taggaaatga tattctgaat catctaacta taataataga taagatcaac caacatttat
92341 ccaatttgaa aaagattaag aagaagtggt tcgatcctct tatttctcga accgagagat
92401 ccacgaatct ggatcctaat gtatatagat acaaatgttc caatggaagc aagaatttcc
92461 aggaacattt ggagcatttc gtttctgagc agaaacaccg ttttcaagta atgttcgatc
92521 gattacgtat taatcaatat tcgattgatt ggtccgaggt tatcgacaaa caagatttgt
92581 ccaagtcact tcgtttcttt ttgtccaagt cacttctcct tttgtccaag tcgcttctct
92641 ttttatctaa gtcacttcct tttttcgttg tgagtctcgg gaatatctcc attcataggg
92701 ccgaaatcca catctatgaa ttgaaaggtc tgaatgatca acccggcaat cagttgttag
92761 aatcaatagg tgttcaaatc gtttatttga ataaattgaa acccttctta ttgtatgatc
92821 atgatacttc ccaaagatcg aaatttttaa tcaatacagg aacaatatta cctttttttgt
92881 tcaacaagat acaaaagtgc atgattgact cattccgtac tagaaaaaat cgcaagaaat
92941 cctttgagaa cacggattcc tatttctcaa tgatatccca cgatcgaaac aattggttga
93001 atcctcagaa aagttcattg atatcttctt tttatagagc aaacagactt caattcttga
93061 atcatcccca ttgcttctgg ttctattgta acaaaggatt ccatttttat ggggaaaaga
93121 cccgtatcca taattatgat tttacatatg cacaattccc caatatcttg tgcattcgca
93181 acaaaaaatt ttctttgtgt ttcggtaaaa aaaaacatgt tttgggagag agagagacta

93241 tttcaccaat tgagtcacag gtatctggca tattcatacc taacaatgtt tcacaaagtg
93301 gtaacaaaac gtataacttg tacaaatctt tccatttttc aattggatct gatccatccg
93361 ttcctattta ctcgattgca gacatttcgg gaacacctgt aatagaggaa caaatagtca
93421 attttgaaag aacttattgt cagcttcttt cagatatgaa tctatctgat tcagaaggga
93481 aaaacttgca tcactatctc cgtttcaatt caaacatggg tttgattcac actccatgtt
93541 ttgagaaata tgtgccgtcc ggaaagagga aagaactgag tctatgtcta aagaaaaacg
93601 ttgagaaggg ggaagtaggt agaacccttc aacgagatag tgctttttca aatctctcaa
93661 aatggaattt gttccaaacc tatatgccat ggttccttac ttggacgggg tgtaaatatc
93721 tttatttcac cttaaaaaac aatatttatt tgatattgaa tattcccttt caatattccc
93781 taagtggcag tcaaaatttt gtgtccgttt ttcatgatat gatgcatgga tcagatatat
93841 catggccaat tcctcagaaa aagtggtggt cgattcttcc acaacggaat ctgataagtg
93901 agagttcgag taagtgttta cagaatcttc ttctgtccga agaaatgatt catcgaaata
93961 atgagtcacc cattccattg atatggacac atctgagatc accaaatgct tgggagttcc
94021 tctattcaat tcttttcctt cttcttgttg ctggatatct cgttcgtaca catcttctct
94081 ttgttttccg agcctctagt gagttacaga cagagttaga aaagatcaaa tctttgatga
94141 ttccatcata catgattgag ttgcgaaaac ttctggatag gtatcctaca tctgaactga
94201 attctttctg gttaaagaat ctctttctag ttgctctgga acaattagga gattctctgg
94261 aagaaatacg ggattctgct tctggcggca acatgctatt gggtggtggt cccgcttatg
94321 gggtcaaatc aatacgttct aagaagaaat atttgaatat caatctcatc gatctcatca
94381 gtatcatacc aaatcccatc aatcgaatca ctttttcgag aaatacgaga catctaagtc
94441 gtacaagtaa agagatctat tcattgataa gaaaaagaaa aaacgtgaac ggtgattgga
94501 ttgatgataa aatagaatcc tgggtcgcga acagtgattc gattgatgat gaagaaagag
94561 aattcttggt tcagttctcc accttaacga cggaaaaaag gattgatcaa attctattga
94621 gtctgactca tagtgatcgt ttatcaaaga atgactctgg ttatcaaatg attgaacaac
94681 cgggatccat ttacttacga tacttagttg acattcataa aaagtatcta atgaattatg
94741 agttcaatag atcctgttta gcagaaagac ggatattcct tgctcattat cagacaatca
94801 cttattcaca aacctcgtgt ggggctaata gttctcattt cccatctcat ggaaaaccct
94861 tttcgctccg cttagcccta tccccttcta ggggtatttt agtgataggt tctataggaa
94921 ctggacgatc ctatttggtc aaatacctag cgacaaactc ctatgttcct ttcattacgg
94981 tatttccgaa caagttcctg gatgacaagc ctaaaggtta tcttattgac gatatcgata
95041 ttgatgatag tgacgatatc gatattgatg atagtgacga tattgatgat gaccttgata
95101 cggagctgct aactatgacg aatgtgctaa ctatgtatat gacgccgaaa atagaccgat
95161 ttgataccac ccttcaatta gaattagcaa aagcaatgtc cccttgcata atatggattc
95221 caaacattca tgatctgtat gtgaatgagt cgaattactt atccctcggt ctattagtga
95281 actatctctc cagagatagt gaaagatgtt ccactagaaa tattcttgtt attgcttcga
95341 ctcatattcc ccaaaaagtg gatcccactc taatagctcc gaataaatta aagaaatgca
95401 tgaagatacg aaggcttctt attccacaac aacgaaagca ctttttcatt ctttcatata
95461 ctaggggatt taacttggaa aagaaaatgt tccatactaa cagtaacaga ttcgggtcca
95521 taaccatggg ttccaatgca cgagatcttg tagcacttac caatgaggcc ctatcaatta
95581 gtattacaca gaagaaatca attatagaca ctaatacaat tagatcagct cttcatagac
95641 aaacttggga tttgcgatcc caggtaagat cggttcagga tcatgggatc cttttctatc
95701 agataggaag ggctgttgca caaaatgtac ttctaagtaa ttgccccata gatcctatat
95761 ctatctatat gaagaagaaa tcatgtaagg aaggggattc ttatttgtac aaatggtact
95821 tcgaacttgg aacgagcatg aagaaattaa cgatacttct ttatcttttg agttgttctg
95881 ccggatcggt cgctcaagat ctttggtctc cacccggacc cgatgaaaaa aattggatca
95941 cttcttatgg attcgttgag aatgattctg atctagttca tggcctatta gaagtcgaag
96001 gcgctctgtt gggatcctca cggacagaaa aagattgcag tcagtttgat aatgatcgag

96061 tgacattgct tcttcggtcc gaaccaagga atcagttaga tatgatgcaa aacggatctt
96121 gttctatcgt tgatcagaga tttctatatg aaaaatacga atcggagttt gaagaagggg
96181 aaagagaagg agccctcgac ccgcaacaga tagaggagga tttattcaat cacatagttt
96241 gggctcctag aatatggcgc ccttgtggca atctatttga ttgtatcgaa aggaccaatg
96301 aattgggatt tccctattgg gccaggtcat ttcggggcaa gcggatcatt tatcataaag
96361 aggatgagct tcaagagaat gattcggagt tcttgcagag tggaaccatg cagtaccaga
96421 cacgagatag atcttccaaa gaacaaggct tttttcgaat aagccaattc atttgggacc
96481 ctgcagatcc attctttttc ctattcaaag atcagccctt tgtctctgtg ttttcacgcc
96541 gagaattctt tgcagatgaa gagatgtcaa aagggcttat tacttcccaa acaaatcctc
96601 ctacatctat atataaacgc tggttcatca agaatacgca agaaaagcac ttcgaattgt
96661 tgattcatcg ccagagatgg cttcgaacca atagttcatt atctaatgga tctttccgtt
96721 ctaatactcc atccgagagt tatcagtatt tatcaaatct gttcctatct aacggaacgc
96781 tattggatca aatgacaaag gcattgttga gaaagagatg gcttttcccg gatgaaatga
96841 aacatttgat tcatgtaaca ggctaaaacg gactatgtac tttatctgtt gggttacggg
96901 cgggcatttt accagaggtt tctattgtat caatttaccc ttgtgtgatt cctgttgaag
96961 catatactcg gggggtgggt gcagggcgga cgatttcaaa acggactcct cattcattag
97021 atagagaaga tcgccaagat ttcgtgatcc gctgccgaac ctattccaat tccaacagcc
97081 cggactcgga tcgtggggat cgatggaata cttcgtatca acagatactt ggtatatgta
97141 tatcaatatt gattagatcc gagatctgtt attgaattgc tcattcaatg agcatttcaa
97201 tattatgcct tgaagaggac tcgaacctcc acgctcttta gcacgagatt ttgagtctcg
97261 cgtgtctacc atttcaccat caaggcatct tgaaagtgaa tcgtattcca tgaatatgat
97321 atctatctag tgtgatatat ggaatatatg acaaaggtgg agttttggag tatttctatc
97381 gatcggtcag gtcatatagg cccgagtcgg acatcaaatt gcttcgattt gaattatccg
97441 gaggatacct tatatatatc aaaaagatgt acaatcaaac ctatttctcg attcaatcga
97501 agcccaaaga agttaatatg gtacccaaat aacgatagat atgtaaaaag caggtccgat
97561 tacgcctatt cctaatccta aatggaatgt aacgacgtag ggatccatat gtaaacatag
97621 tctctattta catatgctcg aatgacccct tctcataatg agaatgtaca taaccctatt
97681 ccggtctggt ccggtatgga atgaacttat aatctgatga tcgagtcgat tccatgatta
97741 taagttcata accccagccc attcccattt tgggcggaac agatctacta attcttttat
97801 tccagttagt aaaagggatc ttgaactaag aaatagaccc tagaagctaa aagagggtat
97861 cctgagcaat tgcaataatt ggattcattg atattcctgg tatagtagat gctatcacac
97921 atacaatcat actcaattcg atggaattgt ttgatcttaa gggggatctt ctataatttc
97981 gcacgtgagg ggttatttct tggtttcgtc cagtcattaa taacttgatt atttttagat
98041 aatagtagat agaaacaacg ctcgtaagga gtcctattga aaccaagaaa tataggcctg
98101 cctgccatcc acaccagaat agatggagtt ttccgaaaaa acctgctagt ggaggaagac
98161 ctcctaggga taagagacat agggctgaag agagagccaa aaaaggatct ttcgtgtata
98221 atcctgcata atctcgaatg ttatcagttc cggtacgtag accaaatgag acaatgcgag
98281 caaaagttcc tagattcatg gagatataga acagcatata agttatcatg cttgcatatc
98341 catcatttga gtctccaaca attattccaa taattacata tccgatttga cctatggacg
98401 aatatgcaag catacgtttc atgcttgttt gagtaatagc aatgagattc cccaatatca
98461 tgctaagaat agctaggatt tccagaagaa gatgccattc gtttgatgag aaataaaaag
98521 gaatatcgaa aattcgagtg gctgaagctg aagcagctac tttcgaagta acagaaagaa
98581 aagcaacgac tggagtggga gagtcagagt cgaaaagagg attcctcact tctttctctc
98641 attcaaaacc gtgcatgaga ctttcatctc gcacggctcc taagtgataa aagaaagaag
98701 aactcacctt ctttcttttt tgattacctt cctcgcgtat gtataagacc gaatccattc
98761 gatttctaaa aaggattact aatccttaac ttttcgagga atccttcatc agtggttgtg
98821 aatgactgac tttttcaatc ttttcgaccc cggttccgta ggagcacaag tcagaaagat

98881 tgagaaatag aaccatctga tttgattcgt tctcaatagc catgagatga tcatcttagg
98941 gtgatccttt tgtcgacgga tgctcctatt acactcgtag tctctgaagg atgagaacca
99001 actatgtagc atctacatcg ataattcaag tattgtatac gtcattagtc cgatcctttg
99061 taggaactac ccgtaataac gaacttgcaa aatggatctg tttatcataa agagattcgt
99121 tgttcctgac cctgcttcac cttaattgtt atttgaacaa aaagatcaca ataaactttt
99181 ggtaaaagtt atgtcttggt ccgagtgggg atagcatttc tcttctgcat gtccatggag
99241 ttttgaaaaa tccaaacatc tcagagatag atatagaggt aggaatttgt cgaacgaacc
99301 gcactccttc gtatacgtca ggagtccatt gatgagaagg ggctggggaa agcttgaacc
99361 caattcctac agtgatggat ataagcgcaa tggaaattcc tggggagtta tacatttgtg
99421 tattgataag accattcact atttcttgaa gctcgatctc tcccccggat gaaccatata
99481 gccaagagaa accatgaacc agaatagaag agcttgcccc acccatgagt aaatatttcg
99541 tagtagcctc attagaccgt acatctctct tggtatatcc agataatagg taggagcata
99601 aactgaaaga ttctggagct acaaagatag ttattaaatc gttagcacca cataaaaaca
99661 ttcctcctag agtagctgtt aatacgaata acaaaaactc tgttatagcc atttctgtac
99721 attcaatgta ctctacggat agaggaatac atagagttga acatagtaaa ataagaaatt
99781 gaaagatttc gttgaaattg ttcgtttgga aatttcccga aaagctaatc ataggttctt
99841 ctctccatcg gaacaatagg gccgttatgc tcattactaa acttgttgaa gagatgaaat
99901 ataaccaagg tatatctttt tgatcagagg ttgaatcgat catcagaaga agaattaggc
99961 caaaaatgag gatacattct gggaaaatga aacttccatg gaagagaagc aaatgaaacg
100021 ctttcataaa aattctcgta gaatcgagaa tgaagttttc attctgtaca tgccagatcc
100081 tgaattagta actgcatcca atctccgaaa aagtcccaat tgtttcgaac tttcgatttt
100141 tggaatggga tatttacgga atccccatga acaggatcaa accttattcc atggtatttc
100201 catgagattc ctccttctta ttcttaagaa agcccccgag agggcttagt tgatccatga
100261 tttatgtttc atctttcttt tccttttcgt ttgtttcgag aaagatatcg atccattcca
100321 attctttctt tttctattga ttcttttccg atcgagatgt atggatccac ggatctatgt
100381 gtctatatag atcctgttca tggattaacg aaaatgtgca aaagctctat ttgcctctgc
100441 cattctatga gtctcttcct ttttgcgtat ggcatcgcca ctccctttgg cagcatccac
100501 taattcggaa cttaatttga aagccatatt tcgacccgga cgttttcggg atgcccctaa
100561 taaccaacga atggcaagtg cttttccttg tgtagattct atttcaatag gaacttgatg
100621 agtcgatcca cctacacgtc ttgctttgac tgctatatcg ggagttactc cacgtattgc
100681 ttgacgtaaa acagatagtg gatttgtttc tgtcttttgt tgaatctttt tcacggcttg
100741 atagataatt tgataagcca atgatttttt tccgtgtttc agaatacggt taaccaacat
100801 gttaactaat cgattacgat aaattggatc ggattttgca gtttttttctt ctgcagtacc
100861 tcgacgtgac atgagcgtga aagaggttca agaatccgtt ttctttttat acgggctaaa
100921 aacgaatcac ttattttggc tttttgaccc catattgtag ggtggatctc gaaagatatg
100981 aaagatctcc ctccaagccg tacatacgac tttcatcgaa tacggctttc cacagaattt
101041 gatatgtatc tatgaaatcg agtatggaat tctgtttact cactttaaat tgagtagccg
101101 tttccctcct tttcctgcta ggattggaaa tcctgtattt tacatatcca tacgatcgag
101161 tccttgggtt tccgaaatag tgtaaaaaga agtgcttcga atcattgcta tttgactcgg
101221 acctgttctg aaaaagtcga ggtatttcga attgtttgtt gacacggaca aagtaaggga
101281 aaacctctga aatgatttca atattgaacc ttggacatat aatagttccg aatcgaatct
101341 ctttagaaag aagatctttt gtctcatggt agcctgctcc agtcccctta cgaaactttc
101401 gtgattgggt tagccataca cttcacatgt ttctagcgat tcacatggca tcatccaatg
101461 atacaagtct tggataagaa tctacaacgc actagaacgc ccttgttgac gatcctttac
101521 tccgacagca tctaaggttc ctcgaacaat gtgatatctc acaccgggta aatccttaac
101581 ccttcctcct cttactaaga ctacagaatg ttcttgtgaa ttatggccaa taccaggtat
101641 ataagcagtg atttcaaatc cagaggttaa tcgtactctg gcaactttac gtaaggcaga

101701 gtttggtttt ttgggggtga tagtggaaaa gttgacagat aagtcaccct tactgtcact
101761 ctacagaacc gtacatgaga ttttcacctc atacggctcc tcgttcaatt ctttcgaagt
101821 aattgggtcc ttttcctcgt tcgagaatct cctcccttct tccactccgt cccgaagagt
101881 aactaggacc aattcagtca cgttctcatg ttccaattga acactttcca tttttgatta
101941 tgatcaaagg agaagattat tctttttacc aaaatatgcg gatcaaatca cgatcttata
102001 ataagaacaa gagatctttc tcgatcaatc cctttgccct cattcttcga gaatcagaaa
102061 gatccttttt gagtttgaat ttgttcattt ggaatcttta tttatttttg tatttttat
102121 ttatttattt tctttgattc tttatttcga ttttctttcc ctctcttttc tttttattcc
102181 cttccatcat tccttaagtc ccataggttt gatcctgtag aatctgaccc attttctcat
102241 cgaacgaggg gtacgaaata aatccgattg atttttcgat caaaagtact atgtgaaatc
102301 ttcggttttt tcctcttcct ctatcccata ggtacagcgt ttgaatcaat agagaacctt
102361 ttcttctgta tgaatcgata ttattacatt ccaattcctt cccgatacct cccaaggaaa
102421 atcccgaatt ggatcccaaa ttgacgggtt ggtgtgagct tatccatgcg gttatgcact
102481 cttcgaatag gaatccattt tctgaaagat cctggctttc gtgctttggc gggtcgtccg
102541 agatcctttc gatgacctat gttgtgttga agggatatct atatgatccg atcgattgcg
102601 taaggcccgc ggtagcaacg gagccgggaa agtatacaga aaagacagtt cttttctatt
102661 atattagtat tagttagtga tcccggctcg gtgagtcctt tcttccgtga tgaactgttg
102721 gcaccagtcc tacattttgt ctctgtggac cgaggagaaa gggggggctca gcgggaagag
102781 gattgtacca tgagagaagc aaggaggtca acctgtttca aatatacaaa tggattctgg
102841 caatgcaatg tagttggacc ctcatgtcga tccgaatgaa tcagtctttc cacggaggtc
102901 aatctttgcc tgctaggcaa gaggatagca agttacaaat tctgtctcgg taggacatgt
102961 atttctatta ctattaaatt cagaaatgaa gtagttaatg ttggggttac cattatcctt
103021 tttgtagtga cgaatcttgt atgtgttcct aagaacctaa gaaaaggaat ttgtcccttt
103081 ttcgaggtct caaaagggcg tggaaacaca taagaactct tgaatggaaa ttgaaaagag
103141 atgtagctcc agttccttcg gaaatggtaa gatctttggc gcaagaagaa ggggttgatc
103201 cgtatcatct tgacttggtt ctgcttcctc tcttttttta acaataccga gtcgggttct
103261 tctcctacca gtatcgaata gaacatgctg aacaaaatct tcttcctgta aaacctgctc
103321 gatttagatc gggaaaatcg tacggatttt atgaaaccat gtgctatggc tcgaatccgt
103381 agtcaatcct atttccgata ggagcagttg acaattgaat ccaatttttc cattcttttc
103441 gtatccgtaa tagtgcgaaa agaagtcccg gctccgagtt gttcaggaag agtggcgttg
103501 agtttctcga ccctttgcct taggattagt cagttctatt tctcgatggg ggcagggaag
103561 ggatataact cagcggtaga gtgtcacctt gacgtggtgg aagtcatcag ttcgagcctg
103621 attatcccta aacccaacgc aatgtgagtt tttctatttt gacttgctcc cccgccgtga
103681 tcgaatgaga atggataaga ggctcgtggg attgacgtga gagggtaggg atggctatat
103741 tgctgggagc gaactccagg ctaatatgaa gcgcatggat acaagccttg gaatgaaaga
103801 caattccgaa tcagctttgt ctacgaaccg gaagattggt aagtaatgca actatgaatc
103861 tcatggagag ttcgatcccg gctcaggatg aacgctggcg gcatgcctaa cacatgcaag
103921 tcggacggga agtggtgttt ccagtggcgg acgggtgagt aacgcgtaag aacctgccct
103981 tgggaggggga acaacaactg gaaacggctg ctaatacccc gtaggctgag gagcaaaagg
104041 aggaatctgc ccgaggaggg gcttgcgtct gattagctag ttggtgaggc aatagcttac
104101 caaggcgatg atcagtagct ggtccgagag gatgatcagc cacactggga ctgagacacg
104161 gcccagactc ctacgggagg cagcagtggg gaattttccg caatgggcga aagcctgacg
104221 gagcaatgcc gcgtggaggt agaaggccca cgggtcgtga acttcttttc ccggagaaga
104281 agcaatgacg gtatctgagg aataagcatc ggctaactct gtgccagcag ccgcggtaag
104341 acagaggatg caagcgttat ctggaatgat tgggcgtaag gcgtctgtag gtggcttttc
104401 aagtccgccg tccaatccca gggctcaacc ctggacaggc ggtggaaact accaagctgg
104461 agtacggtag gggcagaggg aatttccggt ggagcggtga aatgcgtaga gatcggaaag

104521 aacaccaacg gcgaaagcac cctgctgggc cgacactgac actgagagac gaaagctagg
104581 ggagcgaatg ggattagata ccccagtagt cctagccgta aacgatggat actaggcgct
104641 gtgcgtatcg acccgtgcag tgctgtagct aacgcgttaa gtatcccgcc tggggagtac
104701 gttcgcaaga atgaaactca aaggaattga cgggggcccg cacaagcggt ggagcatgtg
104761 gtttaattcg atgcaaagcg aagaacctta ccagggcttg acatgccgtg aatcctcttg
104821 aaagagaggg gtgccttcgg gaacgcggac acaggtggtg catggctgtc gtcagctcgt
104881 gccgtaaggt gttgggttaa gtcccgcaac gagcgcaacc ctcgtgttta gttgccacca
104941 ttgagtttgg aaccctgaac agaccgccgg tgataagccg gaggaaggtg aggatgacgt
105001 caagtcatca tgccccctat gccctgggcg acacacgtgt tacaatggcc gggacaaagg
105061 gtcgcgatcc cgccagggtg agctaactcc aaaaacccgt cctaagttcg gattgcaggc
105121 tgcaactcgc ctgcatgaag ccggaatcgc tagtaatcgc cggtcagcca tacggcggtg
105181 aattcgttcc cgggccttgt acacaccgcc cgtcacactg tgggagctgg ctatgcccga
105241 agtcgttacc ttaaccgcaa ggagggggat gccgaaggcg gggctagtga ctggagtgaa
105301 gtcgtaacaa ggtagccgta ctggaaggtg cggctggatc acctcctttt cagggagagc
105361 taaagaagcg agctacgtct gagctaagct tggagatgga agtcttcttt cgtttctcga
105421 cggtgaagta agacaagctc atgagcttat tatcctaggt cggaacaagt tgataggatc
105481 ccctttttta cgtccccatg cccctcccgt gtggcgacat gggggcgcaa aaaggaaaga
105541 gagggatggg ctttctctcg cttttggcat agcgggcctc cccgtggggg gcccgcaagg
105601 gctattagct cagtggtaga gcgcgcccct gataattgcg tcgttgtgcc tgggctgtga
105661 gggctctcag ccacatggat agttcaatgt gctcatcagc gcctgacccg gggatgtgga
105721 tcatccaagg cacattagca tggcgtactc ctcctgttcg aaccggagtt tgaaaccaaa
105781 cttctcctca ggaggataga tggggcgatt caggtgagat ccaatgtaga tccaactttc
105841 tattcactcg tgggatccgg gcggtccggg ggggaccacc atggctcctc tcttctcgag
105901 aatccataca tcccttatca gtgtatggac agctatctct cgagcacagg tttaggttcg
105961 gcctcaatgg gaaaatggag cacctaacaa cgcatcttca cagaccaaga actacgagat
106021 cacccctttc attctggggt gacggaggga tcgtaccatt cgagcctttt tttttcatgc
106081 ttttcccgcg gaggtctgga gaaagcagca atcaatagga tttccctaat cctcccttcc
106141 cgaaaggaag aacgtgaaat tctttttcct ttccgcaggg accaggagat tggatctagc
106201 cataagaaga atgcttggta taaataactc acttcttggt cttcgacccc ctcagtcact
106261 acgaacgccc cccgatcagt gcaatgggat gtgtctattt atctatctct tgactcaaaa
106321 tgggagcagg tttgaaaaag gatcttagag tgtctagggt tgggccagga gggtctctta
106381 acgccttctt tttcttctca tcggagttat ttcacaaata cttgccatgg taaggaagaa
106441 ggtgggaaca agcacacttg gagagcgcag tacaacggag agttgtatgc tgcgttcggg
106501 aaggatgaat cgcccccgaa aaagaatcta ttgattctct cccaattggt tggatcgtag
106561 gtgcgatgat ttacttcacg ggcgaggtct ctggttcaag tccaggatgg cccagctgcg
106621 ccagggaaaa gaatcgaaga agcatctgac tccttcatgc atgctccact gggctcgggg
106681 ggatatagct cagttggtag agctccgctc ttgcaattgg gtcgttgcga ttacgggttg
106741 gatgtctaat tgtccaggcg gtaatgatag tatcttgtac ctgaaccggt ggctcacttt
106801 ttctaagtaa tggggaagag gaccgaaaca tgccactgaa agactctact gagacaaaga
106861 tgggctgtca agaacgtaga ggaggtagga tgggcagttg gtcagatcta gtatggatcg
106921 tacatggacg atagttggag tcggcggctc tcctagggtt ccctcatctg gatccctggg
106981 gaagaggatc aagttggccc ttgcgaacag cttgatgcac tatctccctt caaccctttg
107041 agcgaaatgt ggcaaaagga aggaaaatcc atggaccgac cccatcgtct ccaccccgta
107101 ggaactacga gatcacccca aggacgcctt cggcatccag ggtcacaga ccgaccatag
107161 accctgttca ataagtggaa cgcattagct gtccgctctc cggttgggca gtaagggtcg
107221 gagaagggca atcactcatt cttaaaacca gcattcttaa gaccaaagag tcgggcggaa
107281 aaaggggaag agctccccgt tcctggttct cgtgtagctg gatcctccgg aaccacaaga

107341 atccttagaa tgggattcca actcagcacc ctttgagatt ttgagaagag ttgctctttg
107401 gagagcacag tacgatgaaa gttgtaagct gtgttcgggg gggagttatt gtctatcgtt
107461 ggcctctatg atagaatcag tcggggaggc ccgagaggcg gtggtttacc ctgtggcgga
107521 tgtcagcggt tcgagtccgc ttatctccag ctcgtgacct tagccgatgc aaaggtatat
107581 gatagcaccc aatttttccg attcggcagt tcgatctatg atttctcatt catggacgtt
107641 gataagatcc ttccatttag cagcacctta ggatggcata gccaacacat taatggcgag
107701 gttcaaacga ggaaaggctt acggtggata cctaggcacc cagagacgag gaagggcgta
107761 gcaagcgacg aaatgcttcg ggaagttgaa aataagcata gatccggaga ttcccaaata
107821 ggtcaacctt tcgaactgcc tgctgaatcc atgagcaggc aagagacaac ctggcgaact
107881 gaaacatctt agtagccaga ggaaaagaaa gcaaaagcga ttcccgtagt agcggcgagc
107941 gaaatgggag cagcctaaac cgtgaaaacg aggttgtggg agagcaatac aagcgtcgtg
108001 ctgctaggcg aagcggtgga gtgccgcacc ctagatggat aaagtccagt agccgaaagc
108061 atcactagct tacgctctga cccgagtagc atggggcacg tggaatcccg tgtgaatcag
108121 caaggaccac cttgcaaggc taaatactcc tgggtgaccg atagcgaagt agtaccgtga
108181 gggaaaggtg aaaagaaccc ccagcgggga gtgaaataga acatgaaacc gtgctgagct
108241 cccaagcagt gggaggagaa agtgatctct gaccgcgtgc ctgttgaaga atgagccggc
108301 gactcatagg cagtggcttg gttaagggaa cggaacccac cggagccgta gcgaaagcga
108361 gtcttcatag ggcaattgtc actgcttatg gacccgaacc tgggtgatct atccatgacc
108421 aggatgaagc ttggatgaaa ctaagcagag gtccgaaccg actgatgttg aagaatcagc
108481 ggatgagttg tggttagggg tgaaatgcca ctcgaaccca gagctagctg gttctccccg
108541 aaatgcgttg aggcgcagca gttgactgga catctagggg taaagcgctg tttcggtgcg
108601 ggccgcgaga gcggtaccaa atcgaggcaa actctgaata ctagatatga ccccaaaata
108661 acaggggtca agttcggaca gtgagacgat gggggataag cttcatcgtc gagagggaaa
108721 cagcccggat caccagctaa ggcccctaaa tgaccgctca gtaataaagg aggtaggggt
108781 gcagagacag ccaggaggtt tgcctagaag cagccaccct tgaaagagtg cgtaatagct
108841 cactgatcga gtgctcttgc gccgaagatg aacggggcta agcgatctgc cgaagctgtg
108901 ggatgtaaaa atgcatcggt aggggagcgt tccgcttaga gggaagctcc cgcgcgagca
108961 ggtgtggacg aagcggaagc gagaatgtcg gcttgagtaa cgcaaacatt ggtgagaatc
109021 caatgccccg aaaacctaag ggttcctccg caaggttcgt ccacggaggg tgagtcaggg
109081 cctaagatca ggccgaaagg cgtagtcgat ggacaacagg tgaatattcc tgtactaccc
109141 cttgttggtc ccgaggtacg gaggaggcta ggttagccga aagatggtta tcggttcaag
109201 gacgcaaggt caccttgctt ttttagggca gggtaagaag ggtagagga aatgccccga
109261 gccaatgtcc gagtaccagg cgctacggcg ctgaagtaac tcatgccata ctcccaggaa
109321 aagctcgaag gaccttcaac aaaagggtac ctgtacccga aaccgacaca ggtgggtagg
109381 tagagaatac ctaggggcac gagacaactc tctctaagga actcggcaaa atagccccgt
109441 aacttcggga gaaggggtgc ctcctcacaa aggaggtcgc agtgaccagg cccgggcgac
109501 tgtttaccaa aaacacaggt ctccgcaaag tcgtaagacc atgtatgggg gctgacgcct
109561 gcccagtgcc ggaaggtcaa ggaagttggt gaactgatga cagggaagcc ggcgaccgaa
109621 gccccggtga acggcggccg taacaataac ggtcctaagg tagcgaaatt ccttgtcggg
109681 taagttccga cccgcacgaa aggcgtaacg atctgggcac tgtctcggag agagactcgg
109741 tgaaatagac atgtctgtga agatgcggac tacctgcacc tggacagaaa gaccctatga
109801 agctttactg ttccctggga ttggctttgg gcctttcctg cgcagcttag gtggaaggcg
109861 aagaaggccc ccttccgggg gggcccgagc catcagtgag ataccactct ggaagagcta
109921 gaattctaac cttgtgtcag gacccacggg ccaagggaca gtctcaggta gacagtttct
109981 atggggcgta ggcctcccaa aaggtaacgg aggcgtgcaa aagtttcctc gggccagacg
110041 gacattggcc ctcgagtgca aaggcagaag ggagcttgac tgcaagaccc acccgtcgag
110101 cagagacgaa agtcggcctt agtgatccga cggtgccgag tggaagggcc gtcgctcaac

110161 ggataaaagt tactctaggg ataacaggct gatcttcccc aagagtccac atcgacggga
110221 aggtttggca cctcgatgtc ggctcttcgc cacctggagc tgtaggaggt tccaagggtt
110281 gggctgttcg cccattaaag cggtacgtga gctgggttca gaacgtcgtg agacagttcg
110341 gtccatatcc ggtgtgggcg ttagagcatt gagaggacct ttccctagta cgagaggacc
110401 gggaaggacg cacctctggt gtaccagtta tcgtgcccac ggtaaacgct gggtagccaa
110461 gtgcggagag gataactgct gaaagcatat aagtagtaag cccaccccaa gatgagtgct
110521 ctcctattcc gacttcccca gagcctccgg tagcacagcc gagacagcga cgggttctct
110581 gcccctgcgg ggatggagcg acagaagtct tgagaatcca agataaggtc acggcgagac
110641 gagccgttta tcattacgat aggtgtcaag cggaagtgca gtgatgtatg cagctgaggc
110701 atcctaacag accgagagat ttgaaccttg ttcctacatg acccgatcaa ttcgatcagg
110761 cactcgccat ctattttcat tgttcaactg tttgacaaca tgaaaaaacc aaaagctctg
110821 ccctccctct ctatctatcc aagggatgga agggcagagg cctttggtgt cccttccagt
110881 caagaatttg ggcttcacaa tcactagcca atatttctct catacctttc ttcgttcatg
110941 gttcgatatt ctggtgtcct aggcgtagag gaaccacacc aatccatccc ggaacttggt
111001 ggttaaactc tactgcggtg acgatactgt aggggaggtc ctgcggaaaa atagctcgac
111061 gccaggatga taaaaagctt aacacctctt attcttatta ctcaaaaaga aaaaaatgaa
111121 aaggtcgtct tattcaaaac ccaattatga catcccttct ctcccacttc acacctcgga
111181 acgcgctgtt cttatagaga gaaaggcgct ttcacgtctt cttaacccga aatggctgag
111241 gggagaaaag gttccttttt gagggtactc cagggaacag atccagtgga gacggggtgg
111301 ggcctgtagc tcagaggatt agagcacgtg gctacgaacc acggtgtcgg gggttcgaat
111361 ccctcctcgc ccacaaccgg cccaaaaggg aaggaccttt cctttacctc tgggggtagg
111421 aaaatcatga tcgggatagc ggacgcaaag ctatggaact ggggtgtggg tcttttgtcg
111481 aaatggcctt attcttttta tttatcgtga aggaaaaaat cgatacatat agtatgcctg
111541 gcccgaatca gcatatttgt gttttactcc ccgtaactct tcctcagcca ggcttgggaa
111601 gaatagcaga gcaaatacaa gtattagtag catagcaaaa atgcgttcct cgtcattaat
111661 atgtttgctc gcggtaattg tggcctctcg ggagaatcga tgactgcatc tttgatgcac
111721 tgctagtaca tcatctgaga attatgaatt ggctagttgt aaatagcccc agggctatgg
111781 aacaaaggat tatcccggat ctacaccgag gtattgacgg cgattctcaa atatcgcaga
111841 acagaatgcg atgagataga gtgcaataga aacaaagaca gggaacgggt tacctactcc
111901 taacggtcaa agcgagccct ttaattctgt aattctgaat tctttaatta agaattcatc
111961 aaatctcccc aagtaggatt cgaacctacg accagtcagt taacagccaa ccgctctacc
112021 actgagctac tgaggaataa cgggagattc gatctcatag agttcaactc ccgctctcaa
112081 cccatgacca atatgagccc gaagcttcct tcgtaactcc cggaacttct tcgtagtggc
112141 tccgttccat gcctcatttc atagggaacc tcaaagtggc tctatttcat tatattccat
112201 ccatatccca atattccatc catatcccaa ttccattcat ttaatatccc tttggtgtca
112261 ttgacataag agatgtcatt caatcgaaga agagggtatc attgacataa aagatgtcat
112321 ttctagtcta tctgtttcta tctatggaaa gtgaagaaat catcatatag taatcgagaa
112381 attgcaatag aaaagaaaaa agggaggttt gtgatgattt tgaaatcttt tctactaggt
112441 aatctattat ccttatacat gaagataata aattcggtcg ttgtggtcgg actctattat
112501 ggatttctga ccacattctc catagggccc tcttatcttt tccttctccg agctaggatt
112561 atggaagaag gaaccgagaa ggaggtatca gcaacaactg gttttattac gggacagctc
112621 atgatgttca tatcgatcta ttatgcgcct ctgcatctag cattgggtag acctcataca
112681 ataactgtcc tagttctacc gtatcttttg tttcatttct tctggaacaa tcacaaacac
112741 tttttggatt atggatctac taccagaaat tcaatgcgta atctcagcat tcaatgtgta
112801 ttcctgaata atctcatttt tcaattattc aaccatttca ttttaccaag ttcaacgtta
112861 gtcagattag tcaacattta tatgtttcga tgcaacaaca agatgttatt tgtaacaagt
112921 agttttgttg gttggttaat tggtcacatt ttcttcatga aatgggttgg attggtatta

112981 ttctggatac ggcaaaatca ttctattaga tcgaatgtac ttattcgatc taataagtac
113041 cttgtgtcag aattgagaaa ttctatggct cgaatcttta ctattctctt atttatcacc
113101 tgtgtctact atttaggcag aataccgtcg cctattgtca ctaagaaact gaaagaaacc
113161 tcaaaaacgg aagaaagggg ggaaagtgag gaagaaacag atgtagaaat agaaaaaact
113221 tccgaaacga aggggactaa acaggaacaa gagggatcca ccgaagaaga cccttccctt
113281 tgttcggaag aaagggagga tccaaaaaaa ctacatgaaa aaaaaaagag gcaagaaatt
113341 ttgaagttag aaatacttaa agagaaagaa gataaagacc tcttctggtt tgaaaaacct
113401 cttgtgaatc ttcttttcga ctataaacga tgtaatcgtc cattgagata tataaaaaaa
113461 aatttatttc aaaatgctgt aagaaatgaa atgtcacaat atttttttca cgtatgtcca
113521 gttgatggaa aacaaataat atcttttaca tatccaccca gtttatcgat ttttttggaa
113581 atgatgcaaa gaaagatgtc tttgtgtacg accgaaaaac tatcccccga agatctgtat
113641 aatcattggg tttataccaa tgaacaaaaa aggtacagct tgagcaatga attcataaac
113701 cgaatagaag ttctaaacaa gggatctctt actatggatg tgcttgaaaa aaggaccaga
113761 ttgtataatg ataaaaataa ccaagaggat aagaataacc aagaagataa gaataaccaa
113821 gaagataaaa ataaccaaga agataagaat aaccaagaat gcttgcctag agtgtatgat
113881 cctttttttaa acggaccata tcgtggaaca ataaaaaaag tgtattcacg ttcaatggtg
113941 gatgactcaa tcacttcgac agaagattct atagggacag aagattctat aggaatggtt
114001 tggataaata agattcatga taggcttcct actgattacc aaaaacttga acataaaacg
114061 gatacattta atggagaacc attatcgaca gacattggtc ctttcttgac ctctatcagt
114121 gaattagcta ggaaatcaac aactggtttt agtctgaatt ttaaaaagct tgttttaata
114181 tccgaacaaa gaagatttga ttcagaaaat aaaaaaaaat gtttgaaatt tctattcgat
114241 gtaattacaa ctgatcaaaa taatcaaaca attcaaaata aatctattgg aatagaagaa
114301 atcggtaaaa agattcctcg acgatcatac aaattgatca attcttttga agagcgggag
114361 gaggaaaatg aggaagaatc agaagaatca acagaaaatc atgggattcg ttcaagaaaa
114421 gccaaacgtg tggtaattta tactgataag gcggatccgg atcagaatac caatactcat
114481 actagtacca gtactaatag tgatcaagca gaagagttgg ctttggtacg ttactcgcaa
114541 caatcagatt ttcgtcggga tatagtaaaa ggatccatac gcgctcaaag acgtaaaatg
114601 gttatttggg aaatgtttca agcgaatgca cattccctgc tttttttgga cagaatagac
114661 aaaacttttt ttttttcttt tgatatctcc cgaacaatga atctcatttt tagaaattgg
114721 atagatacag gaccgaaact caaaacttcg gattctgagg aggaagaggc aaaagaagag
114781 gcaaaaaaaa tggaagataa aaaaaacgag aatgaacgga tagcaatagc agaaacatgg
114841 gatactttta tatttgctca agcaataaga ggtactatgt tagtaaccca atcgattctt
114901 agaaaataca tcatattgcc ttcattgata atagctaaaa acctcggccg tatgctctta
114961 tttcaattcc ccgagtggta cgaggatttg aaggagtgga atagagaaat gcatgttaaa
115021 tgcacctata atggtgttca attatcagaa acagaatttc cgaaaaactg gttaacagat
115081 ggtattcaga taaaaatcct atttcctttc tgtctgaaac cctggcgcaa atccaaacta
115141 cgatcccatc atagagatcc aatccaaaag aaagggaaaa cagaaaattt ttgtttttta
115201 acaatctggg gaaaggaaac cgaactacct tttggttctg cccgacaaca accttccttt
115261 tttgaaccta tttataatga attcgaaaaa aaaaagataa aagtgaaaaa aaaatgtttt
115321 ctagttctaa gagttttcaa aaaaaaaaca aaacagttta gaaaggtctc aaaagaaaaa
115381 acaagatgga ttatcaaaac gattctattt ttaaaaagaa aaattaaaga gtttgcaaac
115441 gtaaatccaa ttttcttatt tgtattgaag aaagtatatg aaccgaatga aaatggaaaa
115501 gattccataa tcataagcag taataaaatt gttcctaaat cgacatcgac cattcgaatt
115561 agattcatgg attgggcaaa ttattcactg acagaaaaaa aaaagaaaga tctgtccgat
115621 agaacaaccc taatcagaaa tcaaatagaa aggggtgcaa aagacaaaag aaaaatattt
115681 ctaactccgg atataaatat tagtcctaac gatacaagtt gtggtgataa aagatcggaa
115741 tcgcagaaac atatttggca gatatcaaaa ggaaaaagta acagattcat attcatacgc

115801 aaatggcact attttttgac atttctcgac gaaagaatat acatacatat ctttctatat
115861 actgttaatg tttctagagt caacgtacaa cttttccttg aatcaacaaa aaagattatc
115921 gataaataca ttcacaaaga agggattgat gaaataaatc aaaaaaaaat gcactttatt
115981 tcgactataa aaaagtctat ttctaatatt agtaaaaata aatcaaagat ttctggtgac
116041 ctatattcct tttcacaagc atctgtattt tacaaattat cgcaaatcca agctattaat
116101 aagaagtatc atttgagatc tctacttcaa tatcgcgaag catatcttat tcttaaggat
116161 agaatccgga atttttttgg aacacgaaga atattagatt ccaaatcaag gcataaaaaa
116221 cttccgaatt ctggaatgaa tgagtggaaa aactggttaa ggggtcatta tcaatacaat
116281 ttatctcagg ctaggtggtc taaattagta ccgcaaaaat ggcgaactag ggtcaattgg
116341 cgtcgtacga ttcaaaataa agactcaaaa aagaattcat atgaaaaagc ccaattcatt
116401 cattacgaga aaaaaaatga ttatgaagtg aattcattga cgataaaaaa agcaaaatta
116461 aaaaaaaact acagatatga tctttttttca tataaatata ttaattatgg ggataggaaa
116521 gactcatata tttatccatc ctcattacaa gtaaacgagg accgagagat tccatataat
116581 tacaacacac ctaaaattga accattttat gtactggggg atatatgtat tagtgattat
116641 ctaggagaag agtctattat tggtacgggt aaaagtacgg atagaaaata tttggagtgg
116701 aaaattttcg atttatttct tagaaagaat atcgatattg agtcctggac cgatacggat
116761 accgggacca acattaataa aatgactaaa accgagactg attattatca aatgattgat
116821 aagaaagatc ttttctatct cacgattcat caagaaatca acccacccaa tcaaaaaaaa
116881 aagttttttt tgatgggaat gaataaagaa atgctatatc gccccatatt aaatacgaaa
116941 tcttggttct tctcagaatt tgtgccactt tatgatgcat ataagatcaa accgtggatt
117001 ataccaatca aattacttct tttgattttt aatggaaatg aaaacattag tgaaaacaaa
117061 aacattaatg aaaatcaaaa aaaggatctt cgtatatcat ctaatcaaaa agaatatctt
117121 gaattaaaga atcgaaatca agaagaaaaa gaacagctcg gccacggaaa tattggctca
117181 gacgcacgaa aacgacaaaa agattttgaa aaggattaca cggaatcgga cattcaaaaa
117241 cgtgaaaaga aaggacaacc cgagagtaac aagaaagcaa aacaagagtt attcctgaaa
117301 aaatatttgc tttttcaatt gagatgggat gatcttttga ataacagaat tttcaataat
117361 gttaaggtat attgtttcct gcttagacta ataaatgcaa aggaaattgc tatatcctct
117421 attcaaggag gagaaatgca cctggatgta atgttaattc agacgaatcc aactcttcca
117481 gaattgataa aaaagggaat attgattctt gaaccagtac gtctgtctat aaaatgggat
117541 agacaattta ttatgtatca aaccataggt atctcattgg tccataataa taaatgccaa
117601 actaatggaa gatatcgaga aaaaagatat gttgatgaga attatttcaa tggatccatt
117661 gtacaacata aaaagatgct tgtgaataga gacgaaaatc attatgattt gcttgttcct
117721 gaaaatattc tatcccctag gcgtcgtaga gaattgagaa ttctaatttg tttcaattcc
117781 ggaaatagga atgctatgga tagaaatccg gtatttttca atgacaacaa tgtaaggaac
117841 tgggtccaat tttggatga ggacaagcat attgatacag atataaataa attcattcaa
117901 ttcaaattgt ttctttggcc caattatcga ttagaggatt tagcttgtat gaatcgctac
117961 tggtttgata ccaataatgg cagccgtttc agtatgtcaa ggatacatat gtatccacga
118021 ttcggaatta gttgatggtt ggtacatttc ctatatatca ggtgtcggat ttggattgaa
118081 tctagaaatg atttggcaga atcttcttag gtcaaaataa ttttgttttg tgggtacaaa
118141 aattgccctt ctatcgaatc aaattacaaa ttgtacttac aattcatttg aatttaattt
118201 tgatttgatt tgatagaata tagaataaag taatatatga ataaatccag attattgggt
118261 gtatcagatc aaaataactg gcattattat tattcctgat tggtaaaatc catatatgtg
118321 gaaaaaaaaa aaaaaaagag agaaatttta tttttatggt tatggtaaaa aattcattca
118381 tctcagttat tccgaaagaa gaaaaaaaca aagggtctgt tgaatttcaa gtaatcagtt
118441 tcaccaataa gatacagaga cttacttcac attttgaatt gcacagaaaa gattatttat
118501 ctcagatagg tttgcggaaa attctgggaa aacgtcaacg actgctggct tatttgtcaa
118561 agaaaaatag agtgcgttat aaaaaattaa tcgatcaatt agatattcgg gaaccaaaaa

118621 ctcgttaatt tgaagattat ttgaattctt tggtttatta gatcttgaat tttgatgaac
118681 ctcatttatt tccttttcgg caattcatag aataatggat cggagaagaa aacatatgaa
118741 tgtaccggct acaagaaaag acctcatgat agttaatatg ggtcctcacc acccatcaat
118801 gcatggtgtt cttcgactta tcgttactct agatggtgaa gatgttattg actgcgaacc
118861 cgtattgggt tatttacaca gagggatgga aaaaattgcg gaaaaccgaa caattataca
118921 atatcttcct tatgtaacac gttgggatta tttagctact atgttcacag aggcaataac
118981 ggtaaatgca ccagaacaat taggaaatat tcaagtaccc aaaagagcca gctatatcag
119041 agtaattatg ctggagctga gtcgtatagc ttctcattta ttatggcttg gaccttttat
119101 ggcagatatc ggttcacaga ctcccttctt ctatattttc agagaaaggg aattgctata
119161 tgacctattc gaagctgcca caggtatgcg aatgatgcat aattatttcc gtatcggggg
119221 agtcgctgct gatctacctc atggctggat agataaatgt ttggatttct gcgattattc
119281 tttaacagga attgttgaat atcaaaagct tattacgcaa aatcccattt ttttggaacg
119341 agttgaaggg gtgggcatta ttggtgggga ggaagcaata aattggggtt tatcgggacc
119401 aatgctacga gcttccggaa tccaatggga tcttcgtaaa gttgatcatt atgagtgtta
119461 cgatgaattc gattgggaag tccagtggca aaaagaagga gactcattag ctcgttattt
119521 agtacgaatc aatgaaatga cggaatccat aaaaattatt caacaggctc tagaaggaat
119581 tccggggggg ccctatgaga acttagaagt tcgacgcttt gatagagcaa gtgattccga
119641 atggaatggt tttgaatatc gattcattag taaaaagcct tctcccactt ttgaattgtc
119701 gaaacaagaa ctttatgtga gagtcgaagc cccaaaggga gaattgggaa tttttctgat
119761 aggggataat agtgtttttc cctggagatg gaaaattcgt ccacctggtt tcatcaattt
119821 gcaaattctt cctcagctag ttaaaagaat gaaattggcg gatatcatga cgatactagg
119881 tagtatagat atcattatgg gagaagttga tcgttgaaat gataattgat acgacagaag
119941 tacaagctat caattctttt tccagatcgg aatccttaaa agaggtctat gacctcttat
120001 ggctgcttgt ccctattttt actcctgtat cgggaatcac aataggcgta ctcgtgattg
120061 tgtggttaga aagagaaata tctgcaggga tacaacaacg tatcgggcct gaatatgccg
120121 gccccttggg aattcttcaa gctctagcag atgggaccaa actacttttg aaagaggatc
120181 ttctcccatc tagaggagat gttcgtttat tcagtatggg gccatctata gcggtcatat
120241 caattctact aagctattta gtaattcctt ttggctatcg ccttgttcta gccgatctca
120301 gtataggcgt ttttttatgg atcgctattt ccagtattgc tcctattgga cttcttatgt
120361 caggatatgg atcgaataat aaatattcct tttcaggtgg tctacgagct gctgctcaat
120421 ctattagtta tgaaatacca ttaactccgt gtgtgttatc aatatctcta cgtgtgattc
120481 gttggaacat gaacctttac ccctttcctg gaaataaagg aaaggggttg gatgtgttga
120541 atagatacct ttctcttttt attcatcatt cgggtcgatg agttaaacca gatagttata
120601 tgagtgaaac aaaacagctt atgaatttgc agtaagaaga ttgattctca ttccctatgt
120661 acgagagtaa agtggaagta aacataagcg gtcgaaactg tttaccccaa gattggttga
120721 ttagtcatca tgacttgaag cgggtgcaaa agatcaactg tatggagttt ctactattgt
120781 atagtatgtt gttgtatgtt gtgtatgttg tatgtattac cataccgggg atcaatcaaa
120841 aatgagtgga cggttaggaa cacaaaggta cacaaaggat tagtgatgaa gataatgtaa
120901 ggtatccaaa gggatatttc tgcataacat aaaaggaatc ataatgaggg ctttaagttc
120961 gtagaaatga tcaagcagta cttcctcacg attccgatcc agagtatgct tctatccact
121021 gattaaataa atgactgtcg agaacgaagt aatcctttga tttgattttt tagaaacccc
121081 cttctgagtg agaaagaaga acaggaacga aagaaatgga atgcaatagg aaaactgaat
121141 aataagagat ctttgtttat tctttctttc ctcaatccta tccatattta tacggataga
121201 attcttataa tgatttatca actataactc atgaattagt gtctaattct ttttcgtacg
121261 aaaagtatgg gtcgaaatat ctattgaaac aacgagtatt ttattgaagg attaagttat
121321 tactgaacta aaagaattct aagtctaatt agaaaataaa ggatgagatc aattcggaag
121381 cgcttttttt tttttttatt atggcggacg gaattccatt ggtctaattc gggactcttc

121441 gatacatctt tactctaatc tacactacaa acatgccgag gtaataatga accagtcctt
121501 agatttattt gtggccatcg aggagccgta tgaagctgag gtctcatgtg cggttctgga
121561 atagcgatgg gaatagtgat gttatcatcg actatgatta tctaacagtt caagtacagt
121621 tgatatagtt gaggcacagt caaaatatgg gttttggggg tggaatctgt ggcgtcaacc
121681 tatagggttt atagtttttc taatttcttc cctagcggaa tgtgaaagat taccttttga
121741 tttaccagaa gcagaggagg aattagtagc aggttatcaa accgaatatt caggtattaa
121801 atctggttta ttttacgttg cttcttacct aaatctacta gtttcttcat tatttgtaac
121861 agttctttac ttgggcgggt ggaatttctc tattccgtac atattcattt ctgaaccttt
121921 tggaataaat aaaacaggtg gagtctttgg aatgacaatt ggtatcctta ttacattagc
121981 taaagcttat ttgttcctgt tcattcctat cacaacaaga tggactttac ctaggatgag
122041 aatggaccag ctattaaacc ttgggtggaa atttctttta cctatttctc taggtaatct
122101 attattaaca acttcttccc aactcgtttc gctataacaa aatatgatat tctagattca
122161 taacctatct agagcaagag aaagaaacat caaactattc atggatatcc acgatatgtt
122221 ccctatggtg actgggttca tgaattatgg tcaacagaca atacgagctg caaggtatat
122281 tggtcaaagt ttcatgatta ccttatctca cgtgaatcgt ttacctgtga ctattcaata
122341 tccttatgaa aagtcgatca catcggagcg ttttcgtggt cgaatccatt ttgaatttga
122401 taaatgcatt gcttgtgaag tatgtgttcg ggtatgcccc atagatctac ccgttgttca
122461 ttggagattg gaaacggata ttagaaagaa acgattgctt aattatagta ttgattttgg
122521 aatctgtata ttttgtggta attgtgtcga gtattgtcca acaaactgtt tatcaatgac
122581 tgaagaatat gaactttcta cttatgatcg tcacgaattg aattataatc aaattgcttt
122641 gggccggtta ccaatgtcag taattggaga ttacacaatt cgaacaatta cgaattcgac
122701 tccaatcaaa ataatcaggg gtaaacctct tgattcaaaa acgattacca attactaaga
122761 ttccgttttg atttaaagta aaggagtgag gcttctttca ttttgctagg tcagtaaata
122821 actattgatt ggtgagaatc aaggcttgat tttgatttag aatggattca tagatctgtg
122881 atgatttcaa aatatacaat ttcgaactac tctttcagat acagtagcgg gattgatcca
122941 ataactgtat ctatataaat ctacacccct ttaggattca attaggaacg tatcatatac
123001 aagaaaaaaa taaggtaacc tctttttct tgggtcggtt agtaagttta tgaaatattt
123061 cgatttattt atctcttttt ttacacataa tggatttacc tggaccaata catgatattc
123121 ttttagtatt tctgggatca ggtcttatat tagggggtct gggggtggtc ttacttacca
123181 acccaattta ttctgctttt tcattgggac tggttcttgt ttgtatatcc ttattccata
123241 ttccatctaa ctcctatttt gtagctgctg cacagctcct tatttacgta ggagctgtaa
123301 atgttttaat cctatttgct gtgatgttca tgaatggttc agaatattac aaagatttct
123361 atctttggac cgttggggat ggggtcactt cactggtttg tacaagtatt ctttttttcac
123421 taattactac tatctcggat acgtcgtggt acgggattgt ttggactaca agatcaaatc
123481 agattataga gcaggaccta acaagtaacg ttcaacaaat tgggattcat ttatcaacag
123541 atttttacct tccatttgaa ctcatttcta taattctttt agttgctttg ataggtgcaa
123601 ttgctatggc ccggcagtaa agtaattaag taataaatac ttagatccaa aataaaataa
123661 ataaagtctt gttttgttct atgttatcac atccattttc cttcagttcc attttcatat
123721 tctattgttc atatatgaaa ttgaaagggg tttagttcga tccattacta ataccttact
123781 ttgtttcgta cttcatttat attctaatca aatcggtgaa attgttgttc atattgaaat
123841 gaatcaaaat tgatgagggg ttggtcaatg atgaccgaac atgtacttat tttgagtgcc
123901 tatttatttt ctatcggtat ttatggattg atcacaagtc gaaacatggt tagagcactt
123961 atgtgtcttg aacttatact gaatgcggtt aatataaatc tcgtaacatt ttctgatttg
124021 tttgatagtc gtcaattaaa aggagatatt ttctcgattt ttgttatagc tattgcagcc
124081 gctgaagcag ctattgggcc ggctattgtt tcatcgatcc atcgtaacag aaaatcaact
124141 cgtatcaatc aatcgaattt gttgaataaa tagtattaat gatataaata aagacagata
124201 tccacaaaat attcactaat ttagaactag catgtatgat tcgtatgacc atgcttgttg

124261 aaacgtaaga aatcaaagta tcttggccct tgctcatgaa cagatccaga aatagattga
124321 ttatcaaaaa agttctggta aaccactgat tcgtctggcg tctacaacat aatatatata
124381 attcaattac taatgaagcc agatcgaaaa ttcataaagt tcaaaaaatt tatagatcca
124441 atgtcgcatt cagtaaagat ttatgataca tgtatagggt gtactcaatg tgtacgagcc
124501 tgccccacag atgtattgga aatgatacct tgggacggat gtaaagctaa acaaattgct
124561 tctgctccaa gaacagagga ctgtgtaggt tgtaagagat gtgaatccgc ttgtccaacg
124621 gatttcttga gtgttcgggt ttacttatgg catgagacaa ctcgcagcat gggtctagct
124681 tattgatacg ttctagaaaa atccacttga atccatttga ttcctcttta ccgacaaaaa
124741 cccgtactcg agaaattatt ccgagcgcgg gtttttctgg tcaaagtcta tcttgtcttt
124801 accacgagtt attttccttg gttaacaata attgttgttt tgccgatatc cgcgggttcg
124861 tcaattttct ttctccctcg tagagggaat aaaaataagg tggttcggtg gtatactatt
124921 tgtatatgct tattagaact ccttctaacg acctatgcgt tctgttatca tttccaattg
124981 gacgatccat taatccaatt agaagaggct tataaatgga taaatacttt tgattttcac
125041 tggagaccgg gaatcgatgg actttccata ggacccattt tactgacggg attcatcact
125101 actttagcta ctttagcggc tcggccagtt actagagatt cgcgattgtt ccatttcctg
125161 atgttagcaa tgtatagtgg tcaaatagga tcattttcct ctcgagacct tttacttttt
125221 ttcctcatgt gggaattaga attaattcct gtttacctac ttgtatccat atggggaggg
125281 aagaaacgtc tgtactcagc tacaaagttt attttgtaca cagcgggggg ttctattttt
125341 ctcttaatgg gagttccggg tatgggttta tatggctcca atgagccaac attaaatttt
125401 gaaacattag ctaatcaatc gtatcctttg ggattggaaa taatattcta tattggcttc
125461 cttattgctt atgctgtcaa atcgccgatt ataccctac atacatggtt accagatacc
125521 catggagaag cacattacag tacatgtatg cttctagcgg gaatcttatt aaaaatggga
125581 gcgtatggat tggttcggat caatatggaa ttattacccc atgctcattc tatattttct
125641 ccctggttga tgatagtagg agcgattcaa ataatctatg cagcttcaac ttctttcggt
125701 caacgcaatt taaaaaagag aatagcctat tcttccgtat ctcatatggg tttcacactt
125761 ataggaattg gttccataac cgatacggga atcaatggag ccatttaca aataatctct
125821 catggattta ttggtgctgc acttttttc ttggcaggaa cgagctacga tagaatacgt
125881 cttgtttatc tcgacgaaat gggaggaata gctatcccaa tgccaaaaat atttaccatg
125941 ttcagtagct tctcgatggc ttctcttgca ttgccaggaa tgagtggttt tgttgcggaa
126001 tcagtagtat tttttggaat aattactagc ccgaaatatc ttttaatgcc aaaaatacta
126061 ataactttcg taatggcaat tggaatgata ttaactccta tttattcatt atctatgtca
126121 cgtcggatgt tctatggcta caagctattc aacgttccaa actcttattt ttttgattct
126181 ggaccacgag aactatttgt ttcggtctgt atccttctac ctgtaatagg tattggtatt
126241 tatcctgatt tcgttctctc gctatcaatt gacaggatag aagctattct atctatttat
126301 tttcataaat agttttcatc aataagacat tacattaatg taaaagaact gtgtgatttt
126361 taaaagcgtt caatgaaaga aaaaaaaaat gaagtgaatt ataatcagat acatctaaag
126421 ttttttcgaa ccatttgaat caagtagtga ttcaaatggt tcgaaaaaac ttgcacaaac
126481 cttctttata taatatacgg gacgatgttc ctatgtattc ttcaggccct ttcttcaatt
126541 agttgttaat gtgaatgaac cataactatg tagtcctatt cctaatagat tgaccccaaa
126601 atagcatatc caaattataa gaaatcctat agaagccaca attgccgaat ccacaccctg
126661 aaagctctga tttgttctac tgtgtaaata aatcgcgaat atggtccaag taataaaagc
126721 ccaagtttcc ttggggtccc aattccaata agaccccat gcctcattag cccatactgc
126781 tcccgaaaga atacctatgg ttaaaaaagt aaaccctaga ctaatgacac gataactaca
126841 ctgatctaat tgttgagtta attgatacct gtgataattt ctaaatgaaa gaaaagaagt
126901 gttttgtaaa acgcttcttt tttcattcac aaaggaaaat gacccaatta ataaatgatt
126961 gcttttgcga ggaatatcta ggttttttcg aaatgtaatg actaaaagag ctatggataa
127021 taacgatcca cataaaagag ctgcataact caataacatc atacttacgt gcatcattaa

127081 ccactgggat tgtagagcag gtactaatat tgcagattga tgcatttcgg ttgaaagacc
127141 cgaagtggca aagccttggg taaaaatagc acttggcgcg gttattgcgc ttaaataact
127201 tttctggttc cgtcttttag gaaacatatg aataatggag aaactccatg aaagaaacat
127261 taaagattca tataaatcgc ttaacggcaa atgtctcgaa taaatccaac gagtaactaa
127321 taatcctgtt atacagaaaa aggtagccat catggctttt tctgacaaat caaatagtac
127381 tacggtttca tggattaata aggtcatcaa atgaatcgta ataacaattg aaatgataga
127441 aaaagagatg tgagttaata tatgttctaa agtggcaaat atcataattt tttttagggg
127501 ggtatcccca attacggaat ggaaatccgg aattgaattc attataggat ctattgtgcc
127561 ttttttagag gatgccgcca ctcggactcg aaccgagatg ctctagcact gcttcctaag
127621 agcagcgtgt ctaccaattt caccatggcg gcatcatcga aataatcata gtccatatga
127681 tgttcaatcg tcgagattga gggatttaat gcaatctatt ttaggaaatg ttagaatcga
127741 tgaataaaga cccgttcaaa taaatattta aacgctcaat aatccttagt atcgtggcag
127801 ggggtcgttt taaacagcgg gcttttccgt attataagtt cttctggaat agttggaact
127861 agacggttat gccctgagtc caggtataga actaagaatt tttttctcat tttttgacga
127921 ttcatttctc atttatctga tttgatagat ccgaaatgaa aaagattcat ttttcaatga
127981 acaaaataaa acaagatttt ttatatatca caacttcatc actacatcca aaggatttct
128041 atgaaaattt ggatagtttg gtatcaaaga tgtattaatg attgggatct tcattgtata
128101 cagaacataa tttgtgtgag gatttaccaa acccattagg tattggaccg ggcatatcgt
128161 ataacaaaag agtttcaatc tttatcggaa ttctactgta tgtactttaa cttagaaaac
128221 ttagaaaatc aaatttaaac tgataagatc tctttatata tagatttgaa atctaatatt
128281 aatagataaa ataatttaga tattggatct agatatatcg attgatcgat cctccagctc
128341 aaacatggga taggatccat tggggttgga ttacgtaacg taagattgac tcattattta
128401 gtacataaat atcgatctaa atgggatcct ggcagtttta ttatttgtg ttttttttt
128461 ttatctgttc gaaacaagag ggagtccttg tagatatgta gtgattcaga gagctacaaa
128521 tcaaagtttt aaaatgtata ttttaatcaa ttagtttcaa taatccattg actttgacta
128581 tgaatcttac ccccgcctta ctttggaaca aaaaaggggg ctctaacctc gttcatactt
128641 gtttcagatt ttccaaaaat cttaatgatg tataactatt ggagatacat aatccaataa
128701 ggcaatccct tcctaagaaa aaaaggaaga gttttgttat tgtgaaaaat gaatcgatgg
128761 ggaaagttac aatccccatc tcgcgaatta taaaaaccaa tcaatgaaag gtgaaacctt
128821 gtattttgtg tctaactact tctttttctt tcttttttt ttttttttca aacaaaaaaa
128881 gaaatcattt ttagaaccta gtatacagaa tacttcatat tctacatacc acaacagcta
128941 gttctatctc atattgatga gttcaaaaca ttagttaatg tgaattcttc atcttataaa
129001 tttaatcatc caattcatcg agtcatgctg atgcagattc agatcattcc aaggctttat
129061 tacttgtttg tcgcacaaaa aaactttttg aatgcccggt agaaatagat ttagctaaag
129121 ataaagcttt tgccgcggcc tgatatcccc ttcctttcca aatatttcta cgaatatgct
129181 tttttgacag agaagtacgt ttctttggaa ccgccatttc aaaataaaag ggtcactcat
129241 ttttatagtt ggacgtgaaa gacatttatt gttcaattca aaatagattg ttctttctat
129301 taactattca ttatctatct ttattccata gccgatactt atgcttactt cataacatag
129361 aagagtatgg atacagatag atcagcatcg catatggtat cattaaggat aaaaaaagaa
129421 atgaaataga agaaaaaaga aaaaacgatg tgattttcaa gggaattttt tttttttcac
129481 atttccatta catccaatgt tcgaagaaag aataatttgt actgattggg ggcttcatat
129541 ctttattcaa tctatgtcta cgggcgggat ctactaccgt tgaatcggat gccattgata
129601 agaattaaaa aggttccaat tcatatctca gtctatttag ataatatata tatatattat
129661 aaatgttatt tttaataaat cgaaaagctt aagaaccctt tcctaattta ggaaaccaat
129721 aatgaatgta accttttttct attaattgat caagctcgga gatagagtcc acataattta
129781 aattgaaatt aaatctaaag tagttaatcc gttaaacaat acattacttg ataaaataaa
129841 gggaatttga gtaatttctc tttttagttc tatgcgaagt gacaagtatt ctagcatttt

129901 tcataaacac ataaacataa gtttgtttta ttggactaga agccaatcaa ttccagaatt
129961 tgttttagtt aatgactccg aagaaactaa aaaaaaacta ggtttattgg atcgagttat
130021 tggcagatcc tacgatacat atcagaataa agtacttgaa atttgggtat cattcttttt
130081 ccttactata ttgatataaa tatggataga aatcaccgta tcgtatgtat atagtagaat
130141 aattgaaaat ctcgtatttt ttatcttaat aaatatcttc gttaataact aggtagtagc
130201 ttttaactag taacttgatt atttgaattt tttgtttttt tttttcaata gtttggaaag
130261 aatcagaata attaagaatg aaaaatcata tttgagttct tttatatctt gtatatcttg
130321 attttttttt tatttatttg attattgctc cttccggaag aaataagggc tggtgaatgg
130381 gaaacaatta ataagaataa aaaaagaaag tttgattttc ttatggaaca tacatatcaa
130441 tatgcatgga tcataccttt cgctctactt ccagttacta tgtcaatagg gttgggactt
130501 ctgcttgttc cgaccgcaac aaaaaatctg cgtcgtatgt ggacttttcc tagtgtttca
130561 ttgctaagta tagttatggt tttttcgtcc gatctgtcta ttcaacagat aaatggcagt
130621 tctatctatc aacatctatg gtcttggacc atcaatactg atttttcctt agagttcggc
130681 tacttgatcg atccacttac ttctattatg tcaatactaa tcactacggt tggaatcatg
130741 gttcttattt atagtgacaa ttatatgtct catgatcaag gatatttgag atttttttgct
130801 tatatgagtt tttccaatac ttccatgttg ggattagtta ctagttccaa tttgatacaa
130861 attcatattt tttgggaact agtgggaatg tgttcgtatt tattaatagg tttttggttc
130921 acacgaccgg ctgccgcaaa tgcttgtcaa aaagcgtttg taactaatcg tgtaggggat
130981 tttgggttat tattaggaat cttaggtttt tattggataa cagggagttt cgaatttcga
131041 gatttgttcg aaatcttcaa taacctgatc cgtaataatg gggtcaactc tttatttgct
131101 actctgtgtg cctccctatt attcgtcggt gcagttgcta aatccgcaca atttcccctt
131161 catgtatggt tacctgatgc catggagggg cctactccta tttcagctct tatccatgct
131221 gctactatgg tagcagcagg catttttctt gtcgctcgat ttcttccgct tttcacagtc
131281 ataccttaca taatgaatct catttctttg ataggtgtaa taacggtact attaggagct
131341 actttagctc ttgctcaaag agacattaag agaagtttag cctattctac aatgtctcaa
131401 ttgggttata ttatgttagc cccagggata ggctcttatc gagctgcttt attccatttg
131461 atcactcatg cctattcgaa agcattattg tttttaggat ccggatcaat tattcattca
131521 atggaaccca ttgttggata ttctccagat aaaagtcaga acatggttct tatgggtggt
131581 ttaacaaaat atgtgccaat tacaaaaaat actttttat taggtacact ttctctttgt
131641 ggaattccac ctcttgcttg tttttggtct aaagatgaaa ttcttaatga tagttggttg
131701 tattcaccta ttttcgcgat aatagcttgt ttctcggcgg ggttaactgc attttatatg
131761 tttcggatgt atttacttac ttttgatggt catttacatg ctcattttca aaattacagt
131821 ggcactcaaa atagctcgtt ctattcaata tctatatggg ggaaagaagg aaccaaacca
131881 gttaacagaa atttgttttt atcaacaatg aataataatg aaaaggtttc cttttttttcg
131941 aggaagatat acaaaatgaa cggaaatgta agaaatctga tacgctcctg taggatttat
132001 tttgaaaata aagacacttc aacgtatccc catgaatcag acaatactat gcttttgcct
132061 ctacttatat tggtcctatt tactttgttc gttggatcca taggaattcc tttcgatcaa
132121 ggagtaatcg attttgatat attatcgaaa tggttaactc catcaataaa ccttttacat
132181 caaaattcga actattctgt ggattggtat gaatttgtga caaatgcaat ttattcagtc
132241 agtatagcct gttttggaat attcatagcg tctattttat atgggtctgt taattcatct
132301 tttcagaatt tggacttaat caattcattt gttaaaaaaa caggctctaa gaaaatttta
132361 ttggaccgaa taataaatgt gatatacaat tggtcatata atcgtggtta catagatctt
132421 ttttatgcaa catgcttaac tacaagtata agaggattag ctgaagtaac tcatttttta
132481 gatagacggg taattgacgg aattaccaat ggtgttggtg ttgcaagttt ctttgtagga
132541 gaagggatca aatatgtggg gggagggcga atctcttctt atctctttgt atatttatca
132601 tatgtatccg gctttttatt aatttactat atctattatc tattcttttt gttttgaata
132661 gaataagaag tgactagact tggttatttt tatcattata caatctggtc cttttttcaa

132721 gcacatccat agtaagagat cccttgttta gaacttctat tcggtttatg aattcattgc
132781 tcaagctgta cctttttttgt tcattggtat aaacccaatg attatacaga tcttcggggg
132841 atagtttttc ggtcgtacac aaagacatct ttctttgcct tggttatttt tatcattata
132901 caatctggtc ctttttttcaa gcacatccat agtaagagat cccttgttta gaacttctat
132961 tcggtttatg aattcattgc tcaagctgta cctttttttgt tcattggtat aaacccaatg
133021 attatacaga tcttcggggg atagtttttc ggtcgtacac aaagacatct ttctttgcat
133081 catttccaaa aaaatcgata aactgggtgg atatgtaaaa gatattattt gttttccatc
133141 aactggacat acgtgaaaaa aatattgtga catttcattt cttacagcat tttgaaataa
133201 attttttttt atatatctca atggacgatt acatcgttta tagtcgaaaa gaagattcac
133261 aagaggtttt tcaaaccaga agaggtcttt atcttctttc tctttaagta tttctaactt
133321 caaaatttct tgcctctttt tttttcatg tagtttttttt ggatcctccc tttcttccga
133381 acaaagggaa gggtcttctt cggtggatcc ctcttgttcc tgtttagtcc ccttcgtttc
133441 ggaagttttt tctatttcta catctgtttc ttcctcactt tcccccccttt cttccgtttt
133501 tgaggtttct ttcagtttct tagtgacaat aggcgacggt attctgccta aatagtagac
133561 acaggtgata aataagagaa tagtaaagat tcgagccata gaatttctca attctgacac
133621 aaggtactta ttagatcgaa taagtacatt cgatctaata gaatgatttt gccgtatcca
133681 gaataatacc aatccaaccc atttcatgaa gaaaatgtga ccaattaacc aaccaacaaa
133741 actacttgtt acaaataaca tcttgttgtt gcatcgaaac atataaatgt tgactaatct
133801 gactaacgtt gaacttggta aaatgaaatg gttgaataat tgaaaaatga gattattcag
133861 gaatacacat tgaatgctga gattacgcat tgaatttctg gtagtagatc cataatccaa
133921 aaagtgtttg tgattgttcc agaagaaatg aaacaaaaga tacggtagaa ctaggacagt
133981 tattgtatga ggtctaccca atgctagatg cagaggcgca taatagatcg atatgaacat
134041 catgagctgt cccgtaataa aaccagttgt tgctgatacc tccttctcgg ttccttcttc
134101 cataatccta gctcggagaa ggaaaagata agagggccct atggagaatg tggtcagaaa
134161 tccataatag agtccgacca caacgaccga atttattatc ttcatgtata aggataatag
134221 attacctagt agaaaagatt tcaaaatcat cacaaacctc cctttttttct tttctattgc
134281 aatttctcga ttactatatg atgatttctt cactttccat agatagaaac agatagacta
134341 gaaatgacat cttttatgtc aatgataccc tcttcttcga ttgaatgaca tctcttatgt
134401 caatgacacc aaagggatat taaatgaatg gaattgggat atggatggaa tattgggata
134461 tggatggaat ataatgaaat agagccactt tgaggttccc tatgaaatga ggcatggaac
134521 ggagccacta cgaagaagtt ccgggagtta cgaaggaagc ttcgggctca tattggtcat
134581 gggttgagag cgggagttga actctatgag atcgaatctc ccgttattcc tcagtagctc
134641 agtggtagag cggttggctg ttaactgact ggtcgtaggt tcgaatccta cttggggaga
134701 tttgatgaat tcttaattaa agaattcaga attacagaat taaagggctc gctttgaccg
134761 ttaggagtag gtaacccgtt ccctgtcttt gtttctattg cactctatct catcgcattc
134821 tgttctgcga tatttgagaa tcgccgtcaa tacctcggtg tagatccggg ataatccttt
134881 gttccatagc cctggggcta tttacaacta gccaattcat aattctcaga tgatgtacta
134941 gcagtgcatc aaagatgcag tcatcgattc tcccgagagg ccacaattac cgcgagcaaa
135001 catattaatg acgaggaacg catttttgct atgctactaa tacttgtatt tgctctgcta
135061 ttcttcccaa gcctggctga ggaagagtta cggggagtaa aacacaaata tgctgattcg
135121 ggccaggcat actatatgta tcgatttttt ccttcacgat aaataaaaag aataaggcca
135181 tttcgacaaa agacccacac cccagttcca tagctttgcg tccgctatcc cgatcatgat
135241 tttcctaccc ccagaggtaa aggaaaggtc cttccctttt gggccggttg tgggcgagga
135301 gggattcgaa cccccgacac cgtggttcgt agccacgtgc tctaatcctc tgagctacag
135361 gccccacccc gtctccactg gatctgttcc ctggagtacc ctcaaaaagg aaccttttct
135421 cccctcagcc atttcgggtt aagaagacgt gaaagcgcct ttctctctat aagaacagcg
135481 cgttccgagg tgtgaagtgg gagagaaggg atgtcataat tgggttttga ataagacgac

135541 cttttcattt ttttcttttt gagtaataag aataagaggt gttaagcttt ttatcatcct
135601 ggcgtcgagc tatttttccg caggacctcc cctacagtat cgtcaccgca gtagagttta
135661 accaccaagt tccgggatgg attggtgtgg ttcctctacg cctaggacac cagaatatcg
135721 aaccatgaac gaagaaaggt atgagagaaa tattggctag tgattgtgaa gcccaaattc
135781 ttgactggaa gggacaccaa aggcctctgc ccttccatcc cttggataga tagagaggga
135841 gggcagagct tttggttttt tcatgttgtc aaacagttga acaatgaaaa tagatggcga
135901 gtgcctgatc gaattgatcg ggtcatgtag gaacaaggtt caaatctctc ggtctgttag
135961 gatgcctcag ctgcatacat cactgcactt ccgcttgaca cctatcgtaa tgataaacgg
136021 ctcgtctcgc cgtgacctta tcttggattc tcaagacttc tgtcgctcca tccccgcagg
136081 ggcagagaac ccgtcgctgt ctcggctgtg ctaccggagg ctctggggaa gtcggaatag
136141 gagagcactc atcttggggt gggcttacta cttagatgct ttcagcagtt atccgctccg
136201 cacttggcta cccagcgttt accgtgggca cgataactgg tacaccagag gtgcgtcctt
136261 cccggtcctc tcgtactagg gaaaggtcct ctcaatgccc taacgcccac accggatatg
136321 gaccgaactg tctcacgacg ttctgaaccc agctcacgta ccgctttaat gggcgaacag
136381 cccaaccctt ggaacatact acagccccag gtggcgaaga gccgacatcg aggtgccaaa
136441 ccttcccgtc gatgtgaact cttggggaag atcagcctgt tatccctaga gtaactttta
136501 tccgttgagc gacggccctt ccactcgaca ccgtcggatc actaaggccg actttcgtcc
136561 ctgctcgacg ggcgggtctt gcagtcaagc tcccttctgc ctttgcactc gagggccaat
136621 ctccgtccgg cccgaggaaa cttttgcacg cctccgttac cttttgggag gcctacgccc
136681 catagaaact gtctacctga gactgtccct tggcccgtag gtcctgacac aaggttagaa
136741 ttctagctct tccagagtgg tatctcactg atggctcggg cccccccgga aggaggcctt
136801 cttcgccctc cacctaagct gcgcaggaaa ggcccaaagc caatcccagg gaacagtaaa
136861 gcttcatagg gtctttctgt ccaggtgcag gtagtccgca tcttcacaga catgtctatt
136921 tcaccgagcc tctctccgag acagtgccca gatcgttacg cctttcgtgc gggtcggaac
136981 ttacccgaca aggaatttcg ctaccttagg accgttatag ttacggccgc cgttcaccgg
137041 ggcttcggtc gccggctccc ctgtcatcag gtcaccaact tccttaacct tccggcactg
137101 ggcaggcgtc agcccccata catggtctta cgactttgcg gagacctgtg tttttggtaa
137161 acagtcgccc gggcctggtc actgcgacct cctttgtgag gaggcacccc ttctcccgaa
137221 gttacggggc tattttgccg agttccttag agagagttgt ctcgtgcccc taggtattct
137281 ctacctaccc acctgtgtcg gtttcgggta caggtaccct tttgttgaag gtccttcgag
137341 cttttcctgg gagtatggca tgagttactt cagcgccgta gcgcctggta ctcggacatt
137401 ggctcggggc atttcctcta ccccttctta ccctgcccta aaaaagcaag gtgaccttgc
137461 gtccttgaac cgataaccat ctttcggcta acctagcctc ctccgtacct cgggaccaac
137521 aaggggtagt acaggaatat tcacctgttg tccatcgact acgcctttcg gcctgatctt
137581 aggccctgac tcaccctccg tggacgaacc ttgcggagga acccttaggt tttcggggca
137641 ttggattctc accaatgttt gcgttactca agccgacatt ctcgcttccg cttcgtccac
137701 acctgctcgc gcgggagctt ccctctaagc ggaacgctcc cctaccgatg catttttaca
137761 tcccacagct tcggcagatc gcttagcccc gttcatcttc ggcgcaagag cactcgatca
137821 gtgagctatt acgcactctt tcaagggtgg ctgcttctag gcaaacctcc tggctgtctc
137881 tgcaccccta cctcctttat tactgagcgg tcatttaggg gccttagctg gtgatccggg
137941 ctgtttccct ctcgacgatg aagcttatcc cccatcgtct cactgtccga acttgacccc
138001 tgttattttg gggtcatatc tagtattcag agtttgcctc gatttggtac cgctctcgcg
138061 gcccgcaccg aaacagcgct ttacccctag atgtccagtc aactgctgcg cctcaacgca
138121 tttcggggag aaccagctag ctctgggttc gagtggcatt tcacccctaa ccacaacaag
138181 cttcatcctg gtcatggata gatcacccag gttcgggtcc ataagcagtg acaattaccc
138241 tatgaagacg cgctttcgct acggctccgg tgggttccct taaccaagcc actgcctaag
138301 agtcgccggc tcattcttca acaggcacgc ggtcagagat ttctcctccc actgcttggg

138361 agctcacggt ttcatgttct atttcactcc ccgatggggg ttcttttcac ctttccctca
138421 cggtactact tcgctatcgg tcacccagga gtatttagcc ttgcaagggg gtccttgctg
138481 attcacacgg gattccacgt gccccatgct actcgggtca gagcgtaagc tagtgatgct
138541 ttcggctact ggactctatc catctagggt gcagcactcc accgcttcgc ctagcagcac
138601 gacgcttgta ttgctctccc acaacctcgt tttcacggtt taggctgctc ccatttcgct
138661 cgccgctact acggaaatcg cttttgcttt cttttcctct ggctactaag atgtttcagt
138721 tcgccaggtt gtctcttgcc tgcccatgga ttcagcagca gttcgaaagg ttgacctatt
138781 cgggaatctc cggatctatg cttattttca acttcccgaa gcatttcgtc gcttactacg
138841 cccttcctcc tctctgggtg cctaggtatc caccgtaagc ctttcctcgt ttgaacctcg
138901 ccattaatgt gttggctatg ccatcctaag gtgctgctaa atggaaggat cttatcaacg
138961 tccatgaatg agaaatcata gatcgaactg ccgaatcgga aaaattgggt gctatcatat
139021 acctttgcat cggctaaggt cacgagctgg agataagcgg actcgaaccg ctgacatccg
139081 ccacagggta aaccaccgcc tctcgggcct ccccgactga ttctatcata gaggccaacg
139141 atagacaata actccccccc gaacacagct tacaactttc atcgtactgt gctctccaaa
139201 gagcaactct tctcaaaatc tcaaagggtg ctgagttgga atcccattct aaggattctt
139261 gtggttccgg aggatccagc tacacgagaa ccaggaacgg ggagctcttc cccttttttcc
139321 gcccgactct ttggtcttaa gaatgctggt tttaagaatg agtgattgcc cttctccgac
139381 ccttactgcc caaccggaga gcggacagct aatgcgttcc acttattgaa cagggtctat
139441 ggtcggtctg tgacccctgg atgccgaagg cgtccttggg gtgatctcgt agttcctacg
139501 gggtggagac gatggggtcg gtccatggat tttccttcct tttgccacat ttcgctcaaa
139561 gggttgaagg gagatagtgc atcaagctgt tcgcaagggc caacttgatc ctcttcccca
139621 gggatccaga tgagggaacc ctaggagagc cgccgactcc aactatcgtc catgtacgat
139681 ccatactaga tctgaccaac tgcccatcct acctcctcta cgttcttgac agcccatctt
139741 tgtctcagta gagtctttca gtggcatgtt tcggtcctct tccccattac ttagaaaaag
139801 tgagccaccg gttcaggtac aagatactat cattaccgcc tggacaatta gacatccaac
139861 ccgtaatcgc aacgacccaa ttgcaagagc ggagctctac caactgagct atatcccccc
139921 gagcccagtg gagcatgcat gaaggagtca gatgcttctt cgattctttt ccctggcgca
139981 gctgggccat cctggacttg aaccagagac ctcgcccgtg aagtaaatca tcgcacctac
140041 gatccaacca attgggagag aatcaataga ttctttttcg ggggcgattc atccttcccg
140101 aacgcagcat acaactctcc gttgtactgc gctctccaag tgtgcttgtt cccaccttct
140161 tccttaccat ggcaagtatt tgtgaaataa ctccgatgag aagaaaaaga aggcgttaag
140221 agaccctcct ggcccaaccc tagacactct aagatccttt ttcaaacctg ctcccatttt
140281 gagtcaagag atagataaat agacacatcc cattgcactg atcggggggc gttcgtagtg
140341 actgaggggg tcgaagacca agaagtgagt tatttatacc aagcattctt cttatggcta
140401 gatccaatct cctggtccct gcggaaagga aaaagaattt cacgttcttc ctttcgggaa
140461 gggaggatta gggaaatcct attgattgct gctttctcca gacctccgcg ggaaaagcat
140521 gaaaaaaaaa ggctcgaatg gtacgatccc tccgtcaccc cagaatgaaa ggggtgatct
140581 cgtagttctt ggtctgtgaa gatgcgttgt taggtgctcc attttcccat tgaggccgaa
140641 cctaaacctg tgctcgagag atagctgtcc atacactgat aagggatgta tggattctcg
140701 agaagagagg agccatggtg gtcccccccg gaccgcccgg atcccacgag tgaatagaaa
140761 gttggatcta cattggatct cacctgaatc gccccatcta tcctcctgag gagaagtttg
140821 gtttcaaact ccggttcgaa caggaggagt acgccatgct aatgtgcctt ggatgatcca
140881 catccccggg tcaggcgctg atgagcacat tgaactatcc atgtggctga gagccctcac
140941 agcccaggca caacgacgca attatcaggg gcgcgctcta ccactgagct aatagccctt
141001 gcgggccccc cacggggagg cccgctatgc caaaagcgag agaaagccca tccctctctt
141061 tcctttttgc gcccccatgt cgccacacgg gaggggcatg gggacgtaaa aaagggggatc
141121 ctatcaactt gttccgacct aggataataa gctcatgagc ttgtcttact tcaccgtcga

141181 gaaacgaaag aagacttcca tctccaagct tagctcagac gtagctcgct tctttagctc
141241 tccctgaaaa ggaggtgatc cagccgcacc ttccagtacg gctaccttgt tacgacttca
141301 ctccagtcac tagccccgcc ttcggcatcc ccctccttgc ggttaaggta acgacttcgg
141361 gcatagccag ctcccacagt gtgacgggcg gtgtgtacaa ggcccgggaa cgaattcacc
141421 gccgtatggc tgaccggcga ttactagcga ttccggcttc atgcaggcga gttgcagcct
141481 gcaatccgaa cttaggacgg gtttttggag ttagctcacc ctggcgggat cgcgaccctt
141541 tgtcccggcc attgtaacac gtgtgtcgcc cagggcatag gggcatgat gacttgacgt
141601 catcctcacc ttcctccggc ttatcaccgg cggtctgttc agggttccaa actcaatggt
141661 ggcaactaaa cacgagggtt gcgctcgttg cgggacttaa cccaacacct tacggcacga
141721 gctgacgaca gccatgcacc acctgtgtcc gcgttcccga aggcacccct ctctttcaag
141781 aggattcacg gcatgtcaag ccctggtaag gttcttcgct ttgcatcgaa ttaaaccaca
141841 tgctccaccg cttgtgcggg cccccgtcaa ttcctttgag tttcattctt gcgaacgtac
141901 tccccaggcg ggatacttaa cgcgttagct acagcactgc acgggtcgag tcgcacagca
141961 cctagtatcc atcgtttacg gctaggacta ctggggtatc taatcccatt cgctccccta
142021 gctttcgtct ctcagtgtca gtgtcggccc agcagagtgc tttcgccgtt ggtgttcttt
142081 ccgatctcaa cgcatttcac cgctccaccg gaaattccct ctgcccctac cgtactccag
142141 cttggtagtt tccaccgcct gtccagggtt gagccctggg atttgacggc ggacttgaaa
142201 agccacctac agacgcttta cgcccaatca ttccggataa cgcttgcatc ctctgtctta
142261 ccgcggctgc tggcacagag ttagccgatg cttattcctc agataccgtc attgcttctt
142321 ctccgagaaa agaagttcac gacccgtggg ccttccacct ccacgcggca ttgctccgtc
142381 aggcggccgt gtctcagtcc cagtgtggct gatcatcctc tcggaccagc tactgatcat
142441 cgccttggta agctattgcc tcaccaacta gctaatcaga cgcaagcccc tcctcgggca
142501 gattcctcct tttgctcctc agcctacggg gtattagcaa ccgtttccag ttgttgttcc
142561 cctcccaagg gcaggttctt acgcgttact cacccgtccg ccactggaaa caccacttcc
142621 cgtccgactt gcatgtgtta agcatgccgc cagcgttcat cctgagccag gatcgaactc
142681 tccatgagat tcatagttgc attacttacc aatcttccgg ttcgtagaca aagctgattc
142741 ggaattgtct ttcattccaa ggcttgtatc catgcgcttc atattagcct ggagttcgct
142801 cccagcaata tagccatccc taccctctca cgtcaatccc acgagcctct tatccattct
142861 cattcgatca cggcgggggа gcaagtcaaa atagaaaaac tcacattgcg ttgggtttag
142921 ggataatcag gctcgaactg atgacttcca ccacgtcaag gtgacactct accgctgagt
142981 tatatccctt ccctgccccc atcgagaaat agaactgact aatcctaagg caaagggtcg
143041 agaaactcaa cgccactctt cctgaacaac tcggagccgg gacttctttt cgcactatta
143101 cggatacgaa aagaatggaa aaattggatt caattgtcaa ctgctcctat cggaaatagg
143161 attgactacg gattcgagcc atagcacatg gtttcataaa atccgtacga ttttcccgat
143221 ctaaatcgag caggttttac aggaagaaga ttttgttcag catgttctat tcgatactgg
143281 taggagaaga acccgactcg gtattgttaa aaaaagagag gaagcagaac caagtcaaga
143341 tgatacggat caaccccttc ttcttgcgcc aaagatctta ccatttccga aggaactgga
143401 gctacatctc ttttcaattt ccattcaaga gttcttatgt gtttccacgc ccttttgaga
143461 cctcgaaaaa gggacaaatt ccttttctta ggttcttagg aacacataca agattcgtca
143521 ctacaaaaag gataatggta accccaacat taactacttc atttctgaat ttaatagtaa
143581 tagaaataca tgtcctaccg agacagaatt tgtaacttgc tatcctcttg cctagcaggc
143641 aaagattgac ctccgtggaa agactgattc attcggatcg acatgagggt ccaactacat
143701 tgcattgcca gaatccattt gtatatttga aacaggttga cctccttgct tctctcatgg
143761 tacaatcctc ttcccgctga gcccccсttt ctcctcggtc cacagagaca aaatgtagga
143821 ctggtgccaa cagttcatca cggaagaaag gactcaccga gccgggatca ctaactaata
143881 ctaatataat agaaaagaac tgtcttttct gtatactttc ccggctccgt tgctaccgcg
143941 ggccttacgc aatcgatcgg atcatataga tatcccttca acacaacata ggtcatcgaa

144001 aggatctcgg acgacccgcc aaagcacgaa agccaggatc tttcagaaaa tggattccta
144061 ttcgaagagt gcataaccgc atggataagc tcacaccaac ccgtcaattt gggatccaat
144121 tcgggatttt ccttgggagg tatcgggaag gaattggaat gtaataatat cgattcatac
144181 agaagaaaag gttctctatt gattcaaacg ctgtacctat gggatagagg aagaggaaaa
144241 aaccgaagat ttcacatagt acttttgatc gaaaaatcaa tcggatttat ttcgtacccc
144301 tcgttcgatg agaaaatggg tcagattcta caggatcaaa cctatgggac ttaaggaatg
144361 atggaaggga ataaaaagaa aagagaggga aagaaaatcg aaataaagaa tcaaagaaaa
144421 taaataaata aaaaatacaa aaataaataa agattccaaa tgaacaaatt caaactcaaa
144481 aaggatcttt ctgattctcg aagaatgagg gcaaagggat tgatcgagaa agatctcttg
144541 ttcttattat aagatcgtga tttgatccgc atattttggt aaaaagaata atcttctcct
144601 ttgatcataa tcaaaaatgg aaagtgttca attggaacat gagaacgtga ctgaattggt
144661 cctagttact cttcgggacg gagtggaaga agggaggaga ttctcgaacg aggaaaagga
144721 cccaattact tcgaaagaat tgaacgagga gccgtatgag gtgaaaatct catgtacggt
144781 tctgtagagt gacagtaagg gtgacttatc tgtcaacttt tccactatca cccccaaaaa
144841 accaaactct gccttacgta aagttgccag agtacgatta acctctggat ttgaaatcac
144901 tgcttatata cctggtattg gccataattc acaagaacat tctgtagtct tagtaagagg
144961 aggaagggtt aaggatttac ccggtgtgag atatcacatt gttcgaggaa ccttagatgc
145021 tgtcggagta aaggagatga tgccatgtga atcgctagaa acatgtgaag tgtatggcta
145081 acccaatcac gaaagtttcg taaggggact ggagcaggct accatgagac aaaagatctt
145141 ctttctaaag agattcgatt cggaactatt atatgtccaa ggttcaatat tgaaatcatt
145201 tcagaggttt tcccttactt tgtccgtgtc aacaaacaat tcgaaatacc tcgacttttt
145261 cagaacaggt ccgagtcaaa tagcaatgat tcgaagcact tctttttaca ctatttcgga
145321 aacccaagga ctcgatcgta tggatatgta aaatacagga tttccaatcc tagcaggaaa
145381 aggagggaaa cggctactca atttaaagtg agtaaacaga attccatact cgatttcata
145441 gatacatatc aaattctgtg gaaagccgta ttcgatgaaa gtcgtatgta cggcttggag
145501 ggagatcttt catatctttc gagatccacc ctacaatatg gggtcaaaaa gccaaaataa
145561 gtgattcgtt tttagcccgt ataaaaagaa aacggattct tgaacctctt tcacgctcat
145621 gtcacgtcga ggtactgcag aagaaaaaac tgcaaaatcc gatccaattt atcgtaatcg
145681 attagttaac atgttggtta accgtattct gaaacacgga aaaaaatcat tggcttatca
145741 aattatctat caagccgtga aaaagattca acaaaagaca gaaacaaatc cactatctgt
145801 tttacgtcaa gcaatacgtg gagtaactcc cgatatagca gtcaaagcaa gacgtgtagg
145861 tggatcgact catcaagttc ctattgaaat agaatctaca caaggaaaag cacttgccat
145921 tcgttggtta ttaggggcat cccgaaaacg tccgggtcga aatatggctt tcaaattaag
145981 ttccgaatta gtggatgctg ccaaagggag tggcgatgcc atacgcaaaa aggaagagac
146041 tcatagaatg gcagaggcaa atagagcttt tgcacatttt cgttaatcca tgaacaggat
146101 ctatatagac acatagatcc gtggatccat acatctcgat cggaaaagaa tcaatagaaa
146161 aagaaagaat tggaatggat cgatatcttt ctcgaaacaa acgaaaagga aaagaaagat
146221 gaaacataaa tcatggatca actaagccct ctcgggggct ttcttaagaa taagaaggag
146281 gaatctcatg gaaataccat ggaataaggt ttgatcctgt tcatggggat tccgtaaata
146341 tcccattcca aaaatcgaaa gttcgaaaca attgggactt tttcggagat tggatgcagt
146401 tactaattca ggatctggca tgtacagaat gaaaacttca ttctcgattc tacgagaatt
146461 tttatgaaag cgtttcattt gcttctcttc catggaagtt tcattttccc agaatgtatc
146521 ctcatttttg gcctaattct tcttctgatg atcgattcaa cctctgatca aaaagatata
146581 ccttggttat atttcatctc ttcaacaagt ttagtaatga gcataacggc cctattgttc
146641 cgatggagag aagaacctat gattagcttt tcgggaaatt tccaaacgaa caatttcaac
146701 gaaatctttc aatttcttat tttactatgt tcaactctat gtattcctct atccgtagag
146761 tacattgaat gtacagaaat ggctataaca gagtttttgt tattcgtatt aacagctact

146821 ctaggaggaa tgtttttatg tggtgctaac gatttaataa ctatctttgt agctccagaa
146881 tctttcagtt tatgctccta cctattatct ggatatacca agagagatgt acggtctaat
146941 gaggctacta cgaaatattt actcatgggt ggggcaagct cttctattct ggttcatggt
147001 ttctcttggc tatatggttc atccggggga gagatcgagc ttcaagaaat agtgaatggt
147061 cttatcaata cacaaatgta taactcccca ggaatttcca ttgcgcttat atccatcact
147121 gtaggaattg ggttcaagct ttccccagcc ccttctcatc aatggactcc tgacgtatac
147181 gaaggagtgc ggttcgttcg acaaattcct acctctatat ctatctctga gatgtttgga
147241 tttttcaaaa ctccatggac atgcagaaga gaaatgctat ccccactcgg accaagacat
147301 aacttttacc aaaagtttat tgtgatcttt ttgttcaaat aacaattaag gtgaagcagg
147361 gtcaggaaca acgaatctct ttatgataaa cagatccatt ttttcctaca aaggatcgga
147421 ctaatgacgt atacaatact tgaattatcg atgtagatgc tacatagttg gttctcatcc
147481 ttcagagact acgagtgtaa taggagcatc cgtcgacaaa aggatcaccc taagatgatc
147541 atctcatggc tattgagaac gaatcaaatc agatggttct atttctcaat ctttctgact
147601 tgtgctccta cggaaccggg gtcgaaaaga ttgaaaaagt cagtcattca caaccactga
147661 tgaaggattc ctcgaaaagt taaggattag taatcctttt tagaaatcga atggattcgg
147721 tcttatacat acgcgaggaa ggtaatcatt cttttatcac ttaggagccg tgcgagatga
147781 aagtctcatg cacggttttg aatgagagaa agaagtgagg aatcctcttt tcgactctga
147841 ctctcccact ccagtcgttg cttttctttc tgttacttcg aaagtagctg cttcagcttc
147901 agccactcga attttcgata ttccttttta tttctcatca aacgaatggc atcttcttct
147961 ggaaatccta gctattctta gcatgatatt ggggaatctc attgctatta ctcaaacaag
148021 catgaaacgt atgcttgcat attcgtccat aggtcaaatc ggatatgtaa ttattggaat
148081 aattgttgga gactcaaatg atggatatgc aagcatgata acttatatgc tgttctatat
148141 ctccatgaat ctaggaactt ttgctcgcat tgtctcattt ggtctacgta ccggaactga
148201 taacattcga gattatgcag gattatacac gaaagatcct tttttggctc tctcttcagc
148261 cctatgtctc ttatccctag gaggtcttcc tccactagca ggttttttcg gaaaactcca
148321 tctattctgg tgtggatggc aggcaggcct atatttcttg gtttcaatag gactccttac
148381 gagcgttgtt tctatctact attatctaaa aataatcaag ttattaatga ctggacgaaa
148441 ccaagaaata acccctcacg tgcgaaatta tagaagatcc cccttaagat caaacaattc
148501 catcgaattg agtatgattg tatgtgtgat agcatctact ataccaggaa tatcaatgaa
148561 tccaattatt gcaattgctc aggataccct cttttagctt ctagggtcta tttcttagtt
148621 caagatccct tttactaact ggaataaaag aattagtaga tctgttccgc ccaaaatggg
148681 aatgggctgg ggttatgaac ttataatcat ggaatcgact cgatcatcag attataagtt
148741 cattccatac cggaccagac cggaataggg ttatgtacat tctcattatg agaaggggtc
148801 attcgagcat atgtaaatag agactatgtt tacatatgga tccctacgtc gttacattcc
148861 atttaggatt aggaataggc gtaatcggac ctgcttttta catatctatc gttatttggg
148921 taccatatta acttctttgg gcttcgattg aatcgagaaa taggtttgat tgtacatctt
148981 tttgatatat ataaggtatc ctccggataa ttcaaatcga agcaatttga tgtccgactc
149041 gggcctatat gacatgaccg atcgatagaa atactccaaa actccacctt tgtcatatat
149101 tccatatatc acactagata gatatcatat tcatggaata cgattcactt tcaagatgcc
149161 ttgatggtga aatggtagac acgcgagact caaaatctcg tgctaaagag cgtggaggtt
149221 cgagtcctct tcaaggcata atattgaaat gctcattgaa tgagcaattc aataacagat
149281 ctcggatcta atcaatattg atatacatat accaagtatc tgttgatacg aagtattcca
149341 tcgatcccca cgatccgagt ccgggctgtt ggaattggaa taggttcggt tctctatcta
149401 atgaatgagg agtccgtttt gaaatcgtcc gccctgcacc cacccccgga gtatatgctt
149461 caacaggaat cacacaaggg taaattgata caatagaaac ctctggtaaa atgcccgccc
149521 gtaacccaac agataaagta catagtccgt tttagcctgt tacatgaatc aaatgtttca
149581 tttcatccgg gaaaagccat ctctttctca acaataactc tcggatggag tattagaacg

149641 gaaagatcca ttagataatg aactattggt tcgaagccat ctctggcgat gaatcaacaa
149701 ttcgaagtgc ttttcttgcg tattcttgat gaaccagcgt ttatatatag atgtaggagg
149761 atttgtttgg gaagtaataa gccctttttga catctcttca tctgcaaaga attctcggcg
149821 tgaaaacaca gagacaaagg gctgatcttt gaataggaaa aagaatggat ctgcagggtc
149881 ccaaatgaat tggcttattc gaaaaaagcc ttgttctttg gaagatctat ctcgtgtctg
149941 gtactgcatg gttccactct gcaagaactc cgaatcattc tcttgaagct catcctcttt
150001 atgataaatg atccgcttgc cccgaaatga cctggcccaa tagggaaatc ccaattcatt
150061 ggtcctttcg atacaatcaa atagattgcc acaagggcgc catattctag gagcccaaac
150121 tatgtgattg aataaatcct cctctatctg ttgcgggtcg agggctcctt ctctttcccc
150181 ttcttcaaac tccgattcgt atttttcata tagaaatctc tgatcaacga tagaacaaga
150241 tccgttttgc atcatatcta actgattcct tggttcggac cgaagaagca atgtcactcg
150301 atcattatca aactgactgc aatctttttc tgtccgtgag gatcccaaca gagcgccttc
150361 gacttctaat aggccatgaa ctagatcaga atcattctca acgaatccat aagaagtgat
150421 ccaatttttt tcatcgggtc cgggtggaga ccaaagatct tgagcgaccg atccggcaga
150481 acaactcaaa agataaagaa gtatcgttaa tttcttcatg ctcgttccaa gttcgaagta
150541 ccatttgtac aaataagaat ccccttcctt acatgatttc ttcttcatat agatagatat
150601 aggatctatg gggcaattac ttagaagtac attttgtgca acagcccttc ctatctgata
150661 gaaaaggatc ccatgatcct gaaccgatct tacctgggat cgcaaatccc aagtttgtct
150721 atgaagagct gatctaattg taattgattt cttctgtgta atactaattg atagggcctc
150781 attggtaagt gctacaagat ctcgtgcatt ggaacccatg gttatggacc cgaatctgtt
150841 actgttagta tggaacattt tcttttccaa gtgaaatccc ctagtatatg aaagaatgaa
150901 aaagtttatt cggagctatt agagtgggat ccactttttg gggaatatga gtcgaagcaa
150961 taacaagaat atttctagtg gaacatcttt cactatctct ggagagatag ttcactaata
151021 gaccgaggga taagtaattc gactcattca catacagatc atgaatgttt ggaatccata
151081 ttatgcaagg ggacattgct tttgctaatt ctaattgaag ggtggtatca aatcggtcta
151141 ttttcggcgt catatacata gttagcacat tcgtcatagt tagcagctcc gtatcaaggt
151201 catcatcaat atcgtcacta tcatcaatat cgatatcgtc actatcatca atatcgatat
151261 cgtcaataag ataaccttta ggcttgtcat ccaggaactt gttcggaaat accgtaatga
151321 aaggaacata ggagtttgtc gctaggtatt tgaccaaata ggatcgtcca gttcctatag
151381 aacctatcac taaaataccc ctagaagggg atagggctaa gcggagcgaa aagggttttc
151441 catgagatgg gaaatgagaa ctattagccc cacacgaggt ttgtgaataa gtgattgtct
151501 gataatgagc aaggaatatc cgtctttctg ctaaacagga tctattgaac tcataattca
151561 ttagatactt tttatgaatg tcaactaagt atcgtaagta aatggatccc ggttgttcaa
151621 tcatttgata accagagtca ttctttgata aacgatcact atgagtcaga ctcaatagaa
151681 tttgatcaat ccttttttcc gtcgttaagg tggagaactg aaccaagaat tctctttctt
151741 catcatcaat cgaatcagga ttctatttta tcatcaatcc aatcaccgtt cacgtttttt
151801 ctttttctta tcaatgaata gatctcttta cttgtacgac ttagatgtct cgtatttctc
151861 gaaaaagtga ttcgattgat gggatttggt atgatactga tgagatcgat gagattgata
151921 ttcaaatatt tcttcttaga acgtattgat ttgaccccat aagcgggacc accacccaat
151981 agcatgttgc cgccagaagc agaatcccgt atttcttcca gagaatctcc taattgttcc
152041 agagcaacta gaaagagatt ctttaaccag aaagaattca gttcagatgt aggataccta
152101 tccagaagtt ttcgcaactc aatcatgtat gatggaatca tcaaagattt gatcttttct
152161 aactctgtct gtaactcact agaggctcgg aaaacaaaga gaagatgtgt acgaacgaga
152221 tatccagcaa caagaagaag gaaaagaatt gaatagagga actcccaagc atttggtgat
152281 ctcagatgtg tccatatcaa tggaatgggt gactcattat ttcgatgaat catttcttcg
152341 gacagaagaa gattctgtaa acacttactc gaactctcac ttatcagatt ccgttgtgga
152401 agaatcgacc accacttttt ctgaggaatt ggccatgata tatctgatcc atgcatcata

```
  152461 tcatgaaaaa cggacacaaa attttgactg ccacttaggg aatattgaaa gggaatattc
  152521 aatatcaaat aaatattgtt ttttaaggtg aaataaagat atttacaccc
//
```
